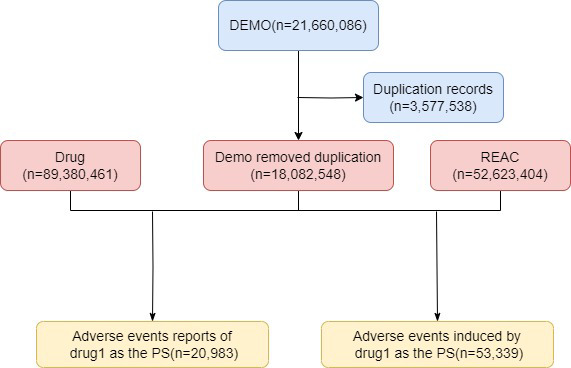# Evaluating adverse reaction signals of vancomycin in pediatric patients: A FAERS database analysis

**DOI:** 10.1097/MD.0000000000049064

**Published:** 2026-06-05

**Authors:** Xue Li, Chaojie Zhang, Jianghong Hou

**Affiliations:** a Graduate Student of Henan University of Traditional Chinese Medicine, Zhengzhou, Henan, China; b Henan Hospital of Traditional Chinese Medicine, Zhengzhou, Henan, China; c Department of Intensive Care, Chengde County Traditional Chinese Medicine Hospital, Chengde, Hebei, China.

**Keywords:** adverse events, FAERS, nephrotoxicity, pediatric patients, vancomycin

## Abstract

Based on the US FDA Adverse Event Reporting System, the adverse reaction (AE) signals of vancomycin in pediatric patients were mined, and its safety characteristics and risk association were evaluated. Data from 2004 to 2024 were extracted. The AE reports (n = 20,983) with vancomycin as the primary suspected drug were screened and standardized by MedDRA (version 26.1). Multi-algorithm fusion strategies were used to detect the signal intensity, and the effects of gender, age, and weight on AE distribution were analyzed. Male, 12 to 18 years old, weight ≥45 kg; the US reported the most, and the main outcome was hospitalization. Skin and subcutaneous tissue disorders reported the most, while renal and urinary disorders have the strongest signal. Acute kidney injury is the most common, and linear immunoglobulin A disease has the highest signal intensity. Acute kidney injury is the main factor for both men and women. The <1 year-old-group has the highest risk of renal and urinary disorders. The signal of renal and urinary disorders was significant in the group <10 kg. Vancomycin is related to nephrotoxicity, skin reaction, and rare immune diseases in pediatric patients, especially in low-birth-weight infants and adolescents. Strengthening therapeutic drug monitoring and individualized medication strategies and paying attention to the influence of gender and geographical differences on AE reports is necessary. This study provides data support for optimizing the safety management of vancomycin in children; however, prospective research is necessary to verify the clinical significance of the FDA Adverse Event Reporting System signal.

## 1. Introduction

Vancomycin is a broad-spectrum antibiotic primarily used to treat severe infections caused by methicillin-resistant *Staphylococcus aureus* and other drug-resistant bacteria.^[1]^ However, recent research has raised significant concerns regarding the efficacy and application of vancomycin. Firstly, a matched cohort study evaluating the clinical effectiveness of vancomycin versus daptomycin in the treatment of methicillin-\resistant *Staphylococcus aureus* bacteremia demonstrated that when the minimum inhibitory concentration of vancomycin exceeded 1 μg/mL, daptomycin exhibited markedly superior therapeutic outcomes. This study revealed a lower clinical failure rate within 30 days with daptomycin treatment, encompassing reductions in both mortality and persistent bacteremia.^[2]^ Moreover, another systematic review and meta-analysis corroborated these findings, indicating that daptomycin is associated with substantially reduced mortality and higher treatment success rates compared with those of vancomycin, particularly in patients classified as being at medium to high risk for infection. In pediatric populations, the need for dose adjustment of vancomycin has also garnered considerable attention.^[3]^ Research indicates that the pharmacokinetics of vancomycin vary in children with obesity and patients with renal insufficiency. Therefore, it is imperative to adjust the dosage based on body weight and creatinine clearance to ensure both effective and safe drug exposure.^[4]^

In summary, vancomycin remains a crucial option for the treatment of drug-resistant bacterial infections. However, its efficacy and safety are influenced by multiple factors, including drug concentration and individual patient characteristics. Therefore, it is essential to implement individualized treatment plans tailored to the specific clinical context. In this study, we analyzed adverse event (AE) data related to vancomycin use in patients under 18 years of age from the FDA Adverse Event Reporting System (FAERS). This analysis aims to provide reference data to support safe clinical use of vancomycin in pediatric populations.

## 2. Materials and methods

### 2.1. Data sources

The data for this study were sourced from the FAERS in the United States, encompassing AE reports from the 1st quarter of 2004 to the 4th quarter of 2024. These reports were retrieved via the official data platform (https://data.nber.org/fda/faers/).^[5]^ Using “vancomycin” as the keyword, we screened for case reports where vancomycin was identified as the primary suspect. We established the following filter parameters in accordance with the research requirements: patient reporting date, gender, age, weight, reporting individual, country, and patient outcome.^[6]^ A systematic data cleaning process was employed to eliminate missing information, logical inconsistencies, duplicate records, and reports not related to AEs (Figure S1, Supplemental Digital Content).

### 2.2. Data processing

All AE reports in this study were standardized and coded based on the terminology system of MedDRA (version 26.1; ICH MedDRA Maintenance and Support Services Organization).

Terminology mapping: The original descriptions of AEs were systematically classified into corresponding preferred terms (PTs).

System classification: Organ systems were categorized according to the system organ classes.

Subgroup analysis: Stratified statistical analyses were performed by gender, age (with age subsections of <1, 1–6, 6–12, and 12–18 years), and weight (<10, 10–45, and >45 kg).

During the data processing phase, we standardized the age data across all records, ensuring that the unit was uniformly converted to “years.” For records in which age was recorded in alternative units – such as “months” or “days” – precise conversions were performed according to established conversion rules, ensuring that all age data are presented in years. Subsequently, we conducted data screening, retaining only reports from individuals aged <18 years. During this screening process, records with missing age fields or with implausible age values (e.g., negative numbers or ages significantly exceeding the typical human lifespan) were marked as “age unknown.” Although these individuals, categorized as “age unknown,” were excluded from further subgroup analyses of the pediatric population, they were preserved in the overall population analysis to maintain the integrity and comprehensiveness of the dataset. For the pediatric population that met the screening criteria (i.e., those aged <18 years and not classified as “age unknown”), we further categorized individuals into 4 distinct subgroups based on age ranges: <1 year, between 1 and 6 years (inclusive), between 6 and 12 years (inclusive), and between 12 and 18 years (inclusive). This stratification enables a more precise and comprehensive analysis of pediatric-related data and research.

For cases with missing key variables, the following processing strategies were implemented:

1. Reports lacking age and weight data were retained in the overall population analysis but were categorized as “unknown” in the subgroup analysis.
2. To evaluate the impact of missing data on the study’s conclusions, a sensitivity analysis was conducted. This involved comparing the consistency of the reporting odds ratio (ROR) values for the top 10 key signals (such as acute kidney injury [AKI]) between information-complete subgroups (where both age and weight were available) and the overall population (which included cases with unknown values).
3. The time-to-onset (TTO) analysis was restricted to reports with complete date information, and entries with logical date errors (i.e., where the onset occurred prior to medication administration) were excluded.

### 2.3. Signal detection method

A multi-algorithm fusion strategy was employed to assess the strength of the association between vancomycin and AE. This evaluation included the calculation of the ROR,^[7]^ proportional reporting ratio (PRR),^[8]^ and empirical Bayes geometric mean (EBGM).^[9]^ Only AE signals that met the criteria established by these 3 algorithms were included in the study; a higher value indicates a stronger potential correlation between the target drug and the specific AE (Tables S1 and S2, Supplemental Digital Content).^[10]^ In the realm of pharmacovigilance signal detection, while each individual disproportionality analysis method possesses its own utility, they inevitably exhibit certain limitations. Specifically, the ROR and the PRR tend to be excessively sensitive to events with few reports, increasing the risk of false positives and compromising signal detection accuracy. In contrast, although the EBGM demonstrates commendable stability, it may be overly conservative in capturing signals, thereby posing a risk of missing true signals.

To enhance the robustness and reliability of signal detection, this study introduces an innovative multi-algorithm fusion strategy. The fundamental principle underlying this strategy is that a target signal is classified as a “positive signal” only when it demonstrates statistical significance across all 3 analytical methods: ROR, PRR, and EBGM. This multi-algorithm fusion strategy offers several notable advantages:

1. Reduction of false positive risk: By cross-verifying detection results across the 3 methods, the strategy effectively filters out signals that may arise incidentally within a single method. This mitigates the potential for erroneous conclusions stemming from the limitations inherent to any single approach, thereby enhancing overall signal-detection accuracy.
2. Improvement of result credibility: A signal is recognized only when it reaches significance across multiple analytically distinct approaches. This ensures that the identified signals exhibit not only a robust statistical association but also methodological consistency, thereby strengthening the credibility of the research findings.
3. Alignment with best practices in pharmacovigilance: The international pharmacovigilance community increasingly favors multi-criteria strategies for signal detection.^[11]^ The multi-algorithm fusion strategy used in this study aligns with these evolving practices, thereby enhancing the credibility of signal detection and strengthening the recognition and impact of the research findings within the global academic community.

TTO was calculated as the duration between the date of the AE (EVENT_DT in the DEMO file) and the start date of vancomycin treatment (START_DT in the THER file). Only reports with available TTO data were included in the analysis. Reports containing input errors (e.g., EVENT_DT occurring before START_DT) were excluded beforehand to ensure the accuracy of the calculations.

### 2.4. Pediatric recommended dose and administration method of vancomycin

According to clinical guidelines, the recommended dose of vancomycin for pediatric patients is adjusted based on factors such as age, weight, and renal function. The typical administration regimen suggests a dosage of 10 to 15 mg/kg per dose, given every 6 hours for children with normal renal function, aiming to maintain a target steady-state trough concentration of 10 to 20 mg/L to effectively manage severe infections. For newborns and infants, due to their immature renal function, the administration interval is generally extended to 8 to 12 hours. Vancomycin is administered via intravenous infusion, and the infusion duration should be at least 60 minutes to minimize the risk of infusion-related reactions, such as erythema syndrome. Therapeutic drug monitoring (TDM) is essential for optimizing the administration regimen, ensuring an appropriate balance between efficacy and the risk of renal toxicity, particularly in children with renal insufficiency, obesity, or critical conditions.^[12]^

## 3. Results

### 3.1. Basic characteristics of vancomycin-related AE

**Figure 1.**
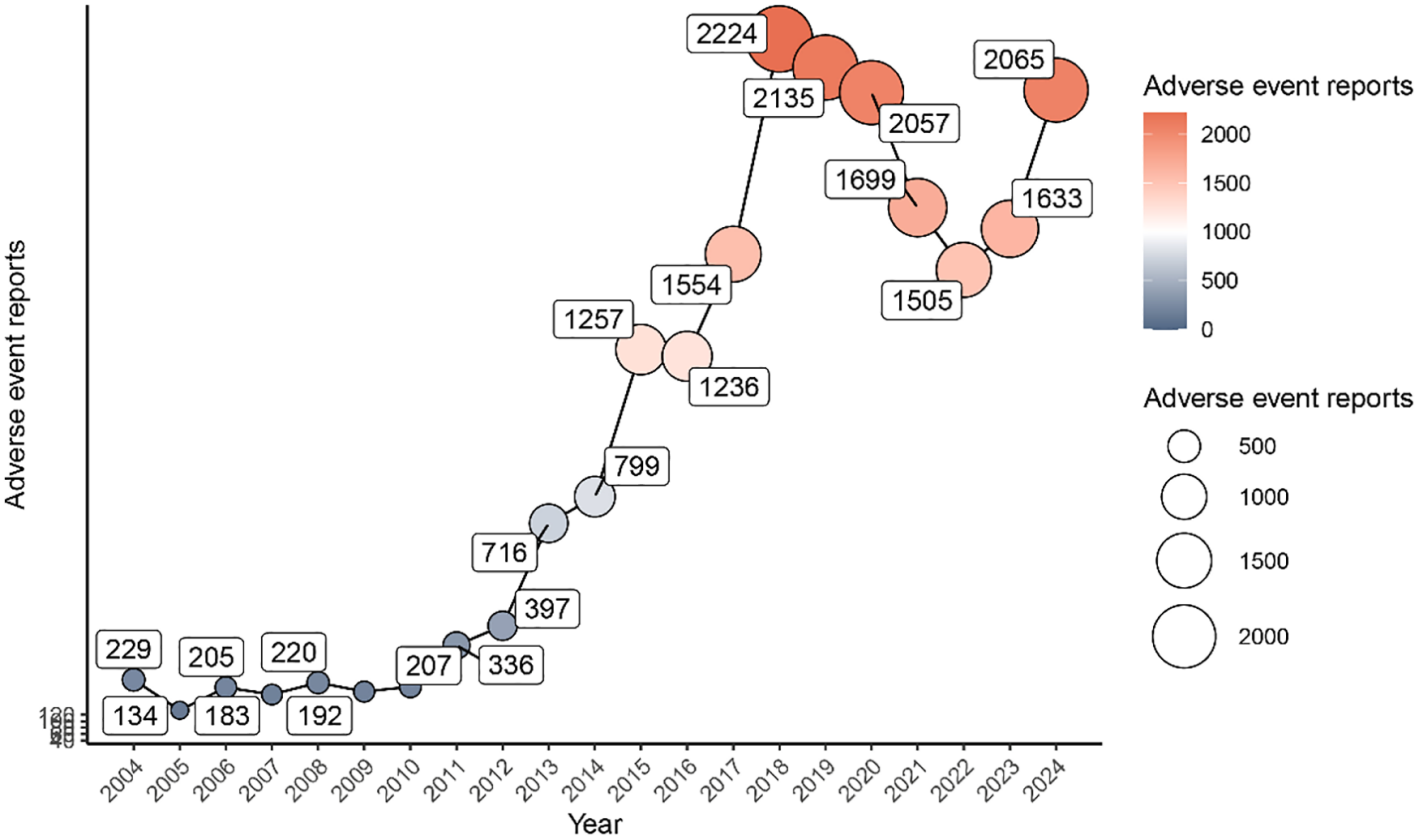
Trend of AE reported by vancomycin from 2004 to 2024. AE = adverse event.

**Figure 2.**
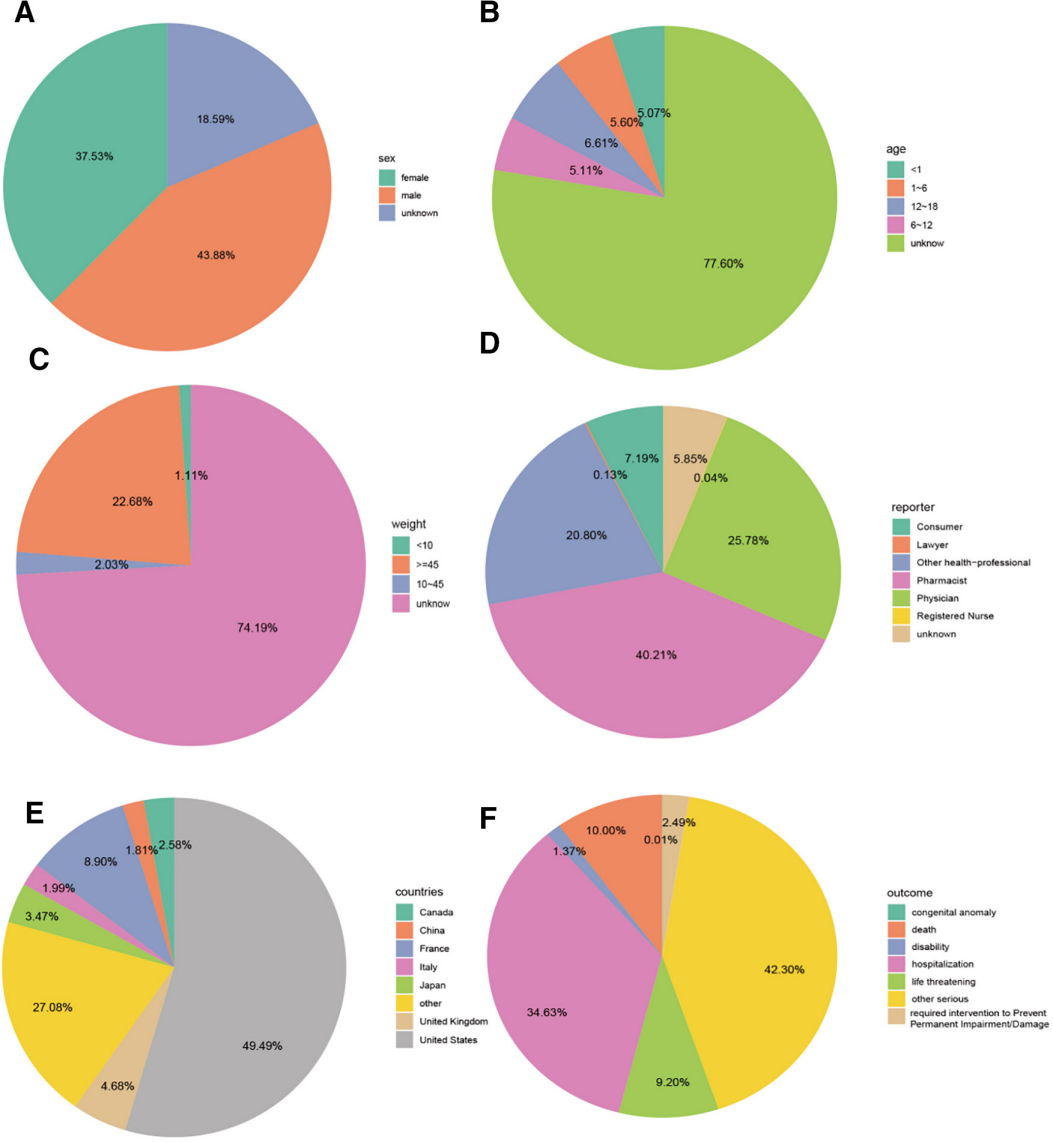
Overview of AE reports for vancomycin from 2004 to 2024. (A) Gender distribution of reported vancomycin AE cases. (B) Proportion of reported ages in vancomycin AE cases. (C) Proportion of reported weight information related to vancomycin AE. (D) Distribution of reporters for vancomycin AE cases. (E) Distribution of reporting countries for vancomycin AE cases. (F) Proportion of outcomes reported in association with vancomycin AE. AE = adverse event.

This study extracted 63,382 AE reports in which vancomycin was the primary suspected drug from the FAERS database, covering the period from January 2004 through the 4th quarter of 2024. Of these reports, 20,983 pertained to pediatric patients (aged <18 years), representing 33.1% of all vancomycin-related reports. As a retrospective real-world pharmacovigilance study, this analysis utilized all available data from the database to ensure the comprehensiveness and representativeness of signal detection. The overall reporting trend shows an increasing pattern, as illustrated in Figure 1. In the reported AE cases, males comprised a significant proportion (n = 9208, 43.88%), as shown in Figure 2A. Among the pediatric population, the majority of cases were reported in the age group of 12 to 18 years (n = 471, 6.61%), as depicted in Figure 2B. Additionally, in terms of weight, the highest number of reports were for individuals weighing ≥45 kg (n = 471, 6.61%), as shown in Figure 2C. Pharmacist is the most reported personas (n = 8438, 40.21%), as shown in Figure 2D. The country with the largest number of reports was the United States (n = 10,385, 49.49%), as shown in Figure 2E. Among the known outcomes, hospitalization was the most frequently reported (n = 8615, 34.63%), as shown in Figure 2F.

### 3.2. AE signal analysis

**Figure 3.**
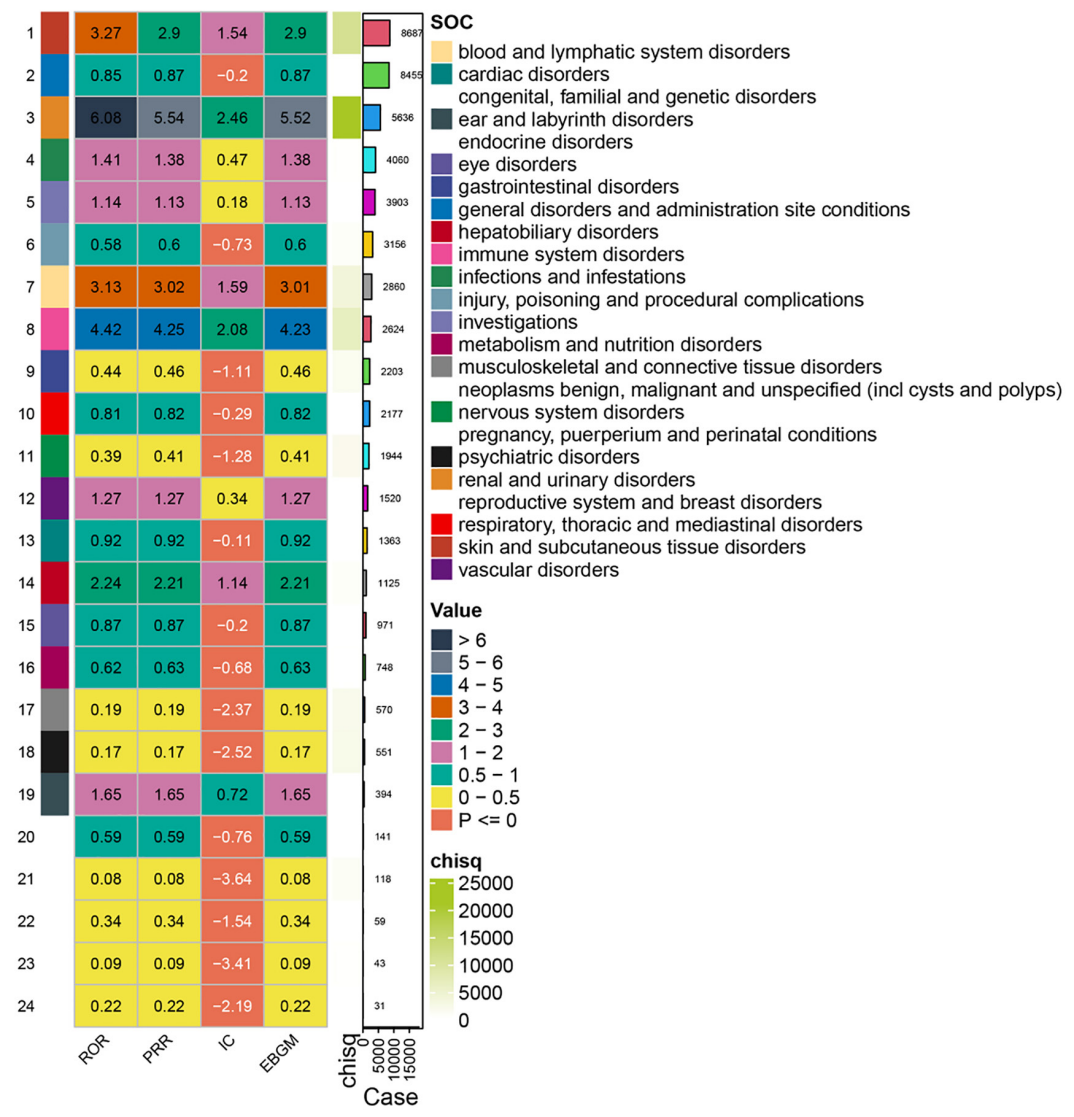
Signal strength of adverse events associated with vancomycin at the SOC level in the FAERS database. EBGM = empirical Bayes geometric mean, FAERS = FDA Adverse Event Reporting System, IC = intensive care, PRR = proportional reporting ratio, ROR = reporting odds ratio, SOCs = system organ classes.

According to the risk signals identified by the MedDRA system, the results indicate that 25 system organ classes are involved. The most frequently reported category is skin and subcutaneous tissue disorders (n = 8687, ROR = 3.27, PRR = 2.90, EBGM = 2.90). The highest number of AE signals was observed in renal and urinary disorders (n = 5636, ROR = 6.08, PRR = 5.54, EBGM = 5.52), as illustrated in Figure 3.

In the analysis of the 3 subgroups of vancomycin at the SOC level, the predominant AEs for both females and males were skin and subcutaneous tissue disorders (females: n = 3684, ROR = 3.24, PRR = 2.85, EBGM = 1.51; males: n = 4037, ROR = 3.84, PRR = 3.37, EBGM = 3.36). Additionally, the highest number of AE signals was recorded in renal and urinary disorders (n = 1841, ROR = 6.38, PRR = 5.91, EBGM = 5.89), as shown in Table S3, Supplemental Digital Content.

In terms of age distribution, the largest number of patients fell into the unknown age group (n = 11,286). The majority of AEs were reported under general disorders and administration site conditions (n = 2304, ROR = 0.97, PRR = 0.98, EBGM = 0.98), with the highest number of AE signals observed in the group aged <1 year for renal and urinary disorders (n = 105, ROR = 8.73, PRR = 7.91, EBGM = 7.68), as summarized in Table S4, Supplemental Digital Content.

Regarding weight categories, the largest number of patients with unknown weight was identified (n = 37,282), with the most cases reported in the general disorders and administration site conditions category (n = 6561, ROR = 0.89, PRR = 0.91, EBGM = 0.91). The highest number of AE signals within the weight segment of <10 kg was also reported for renal and urinary disorders (n = 65, ROR = 7.27, PRR = 6.69, EBGM = 6.57), as indicated in Table S5, Supplemental Digital Content.

A total of 179 PTs were obtained in this study, and the effective signals were arranged in descending order of the reported number of AE, among which the number of AE was AKI (n = 2454, ROR = 19.16, PRR = 18.33, EBGM = 18.01), and the number of AE signals was linear immunoglobulin A (IgA) disease (n = 575, ROR = 60), as summarized in Table S6, Supplemental Digital Content.

In the analysis of the 3 subgroups of vandosing recommendationscomycin at the PT level, the most commonly reported AE for both females and males was AKI (females: n = 812, ROR = 23.33, PRR = 22.47, EBGM = 22.13; males: n = 1178, ROR = 15.30, PRR = 15.30). In the female group, an exceptionally high signal value for Weissella infection was recorded (ROR = 2808.46); however, it is important to note that the number of reports for this event was very low (n = 6). The ROR value derived from rare events is inherently unstable, and therefore, this signal should be considered an exploratory finding, as summarized in Table S7, Supplemental Digital Content. The largest number of patients fell into the unknown age group (n = 3672), with the most reported cases related to pupillary reflex impairment (n = 602, ROR = 14.04, PRR = 13.34, EBGM = 13.24). Additionally, the highest number of AE signals in the pediatric population was recorded in the 12 to 18 age group (n = 12), as summarized in Table S8, Supplemental Digital Content. In terms of weight categories, the largest number of patients was also in the unknown weight segment (n = 17,628). The highest number of reported cases was associated with individuals weighing between 10 and 45 kg (n = 1676, ROR = 19.76, PRR = 18.91, EBGM = 18.59), as summarized in Table S9, Supplemental Digital Content.

### 3.3. AE TTO analysis

**Figure 4.**
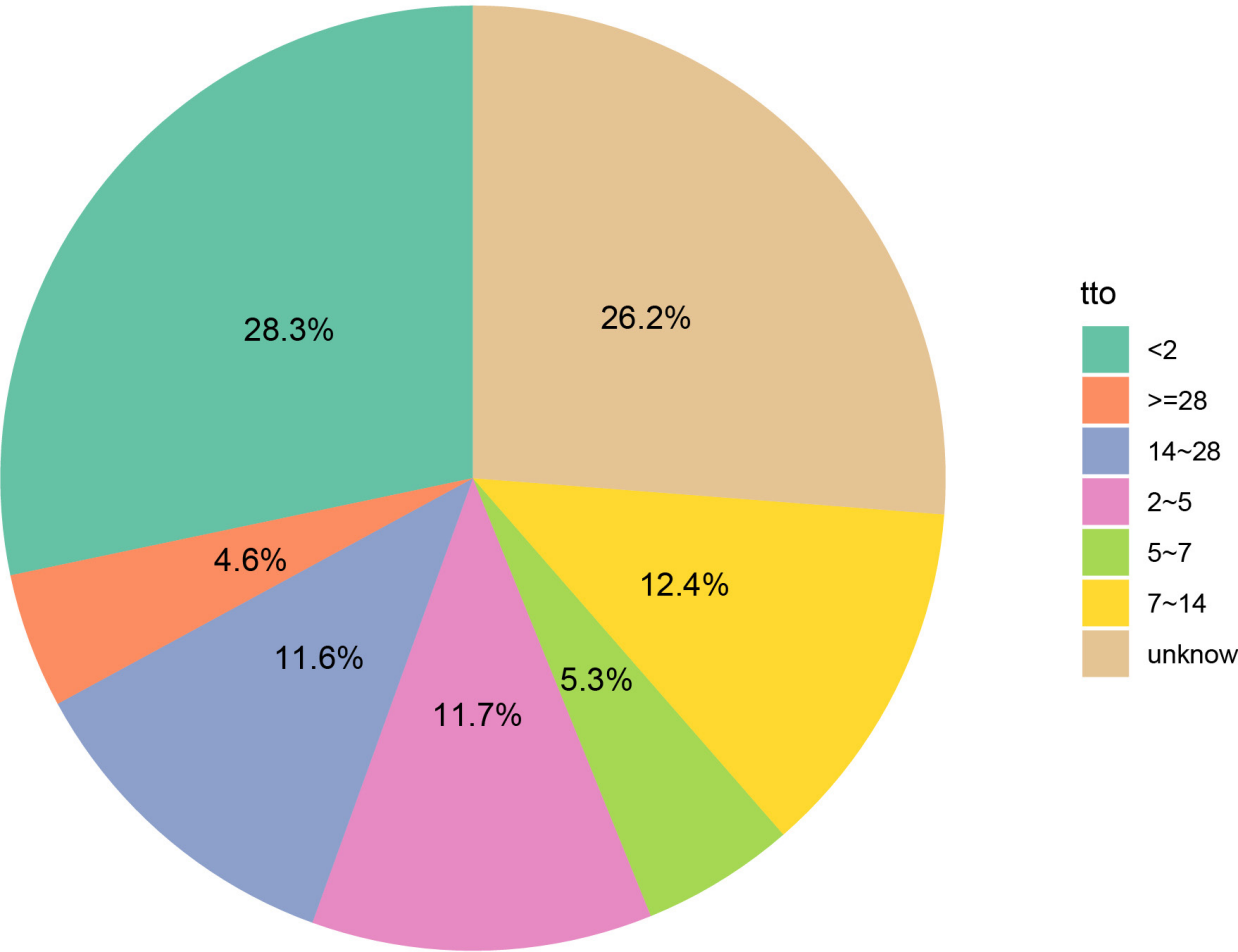
Time-to-onset analysis for the signals of vancomycin. TTO = time-to-onset.

A total of 7509 reports with complete dates were collected in this study. Within the known TTO occurrence time, most AE occurred within 2 days after using vancomycin (Fig. 4).

## 4. Discussion

This study observed that the number of vancomycin reports exhibited an upward trend, potentially linked to increased drug usage, heightened public awareness regarding reporting, or improvements in reporting systems. Although reports from male patients outnumber those from female patients, the overall difference is minimal, suggesting no significant gender-related discrepancies in vancomycin use. Among the age groups analyzed, patients aged 12 to 18 years comprised a substantial proportion, which may stem from certain studies categorizing this age bracket as pediatric. However, the size and pharmacokinetics of patients in this group are often comparable to those of adults, leading clinicians to prescribe vancomycin based on adult dosing strategies. Consequently, this may contribute to a higher frequency of documented medication records. Among patients with known weights, those weighing ≥45 kg were significantly more prevalent, potentially due to the higher incidence of adolescent sports injuries, orthopedic surgeries, or posttraumatic infections, such as skin and soft tissue infections and osteomyelitis, particularly those caused by community-acquired methicillin-resistant *S. aureus*, which necessitate vancomycin treatment.^[13]^ Additionally, the exacerbation of chronic conditions, such as cystic fibrosis, inflammatory bowel disease, or congenital heart disease, often worsens during adolescence, increasing the risk of infections by drug-resistant bacteria.^[14–16]^ Pharmacists filed the majority of reports, largely due to their comprehensive professional responsibilities – including prescription review and TDM, as well as their integral role in systematic monitoring processes and sensitivity to pediatric medication safety. The proportion of reports from the United States was notably higher, reflecting the outcome of an advanced monitoring system, the burden of highly drug-resistant bacteria, and a collaboration model driven by legal and insurance frameworks. This trend not only underscores the United States’ leadership in drug safety management but also highlights the need for ongoing optimization of vancomycin use in pediatrics, including alternative drug development and precise dosing strategies. In terms of reported outcomes, hospitalization may indicate that patients have critical underlying conditions, with vancomycin prescribed for severe infections that warrant inpatient care. The combination of multi-drug regimens and intensive monitoring in hospital settings enhances the detection rates of nephrotoxicity and infusion reactions, while longer treatment courses and necessary dosage adjustments cumulatively raise the risk of toxicity. Additionally, reporting bias may be present, with adverse drug reactions during hospitalization more likely to be systematically recorded. Notably, mortality also constituted a significant proportion of outcomes, attributed to the high fatality rates associated with severe infections and underlying diseases, as well as the direct impacts of drug toxicity (e.g., renal injury and allergic reactions) and potential medication errors. In the future, it is crucial to promote the refinement of pediatric vancomycin medication guidelines to mitigate such risks and prevent tragic outcomes.

Our study not only confirmed the nephrotoxicity and ototoxicity associated with vancomycin as indicated in its prescribing information but also revealed a significant correlation between vancomycin use and immune system disorders. The nephrotoxicity of vancomycin is primarily manifested as AKI, with mechanisms involving direct oxidative damage to renal tubular epithelial cells and mitochondrial dysfunction. Pediatric patients are particularly susceptible to drug accumulation due to immature renal function, especially in newborns with low glomerular filtration rates, as well as considerable individual variability in pharmacokinetics influenced by significant differences in body weight and body surface area. Although the incidence of ototoxicity, including high-frequency hearing loss and tinnitus, is low (<1%), it may be overlooked in children due to their immature auditory systems and the often-subtle symptoms. Research indicates that continuous infusion of vancomycin is more effective than intermittent infusion in achieving therapeutic levels, which may be linked to a reduced incidence of AKI. This finding holds particular significance for children with immune system disorders, as these patients may be more vulnerable to drug toxicity.^[17]^ Additionally, another study examined the pharmacokinetics of vancomycin in children and adolescents with varying levels of obesity and renal function. The results demonstrated that body weight and creatinine clearance significantly influence the clearance rate and volume of distribution of vancomycin. Consequently, it is recommended to adjust dosing based on body weight and renal function to ensure effective and safe drug exposure, particularly for children with immune system disorders, who may exhibit distinct drug metabolism profiles.^[4]^ Regarding the impact of augmented renal clearance on vancomycin treatment, it was observed that in pediatric patients with severe trauma, augmented renal clearance may lead to blood concentrations of vancomycin falling below the therapeutic range. For these patients, continuous infusion of vancomycin can effectively maintain therapeutic levels without AEs. This strategy may also be applicable for children with immune system disorders, who are likely to experience similar alterations in drug metabolism.^[18]^

For events with a very limited number of reported cases, such as Weissella infection, the extremely high ROR values are predominantly a result of the small denominator effect. This occurs because there are either very few or no such events associated with other drugs in the database, leading to a significant amplification of the odds ratio. Consequently, these signals are methodologically unstable and should not be interpreted as indicative of a strong causal relationship between vancomycin and the event in question. Instead, they should be viewed as preliminary clues that necessitate further accumulation of case data, mechanism studies, or targeted monitoring for validation.

In this study, we found that the AEs associated with vancomycin in pediatric patients predominantly occur within the 1st 2 days of treatment. This finding challenges the traditional perception that “toxic reactions are primarily linked to cumulative dosage,” as suggested by earlier studies. The AEs occurring in the initial phase (<2 days) may largely be attributed to direct drug toxicity or immune-mediated acute reactions. The unique pharmacokinetic characteristics of pediatric patients, particularly newborns, may lead to the earlier manifestation of toxicity. In cases of severe infections, such as sepsis, clinicians often administer a loading dose (e.g., 25–30 mg/kg) to promptly achieve therapeutic concentrations, which concurrently increases the risk of early toxicity. Moreover, some institutions have not adhered strictly to the recommendation of “infusion time ≥60 minutes,” resulting in elevated peak concentrations that could precipitate red man syndrome or AKI. The high incidence of early AEs (<2 days) in pediatric patients underscores the potential risk of acute exposure, extending beyond the traditional understanding of cumulative toxicity. Routine TDM is typically conducted after 24 to 48 hours of drug administration, potentially leading to nephrotoxicity or ototoxicity due to elevated concentrations during this period. These findings suggest that the clinical safety management strategies during the early stages of vancomycin treatment should be reevaluated. It is essential to minimize risks through individualized dosing regimens, controlled infusion rates, and early TDM. Future research should focus on identifying biomarkers and genetic polymorphisms that could serve as indicators of high risk in pediatric patients, thereby facilitating the advancement of precise medication practices in this population.

In conclusion, when administering vancomycin to pediatric patients with immune system disorders, it is essential to consider individualized dose adjustments and vigilant monitoring to ensure treatment efficacy and safety. This study utilized the FAERS database to analyze AE signals associated with vancomycin in pediatric populations, examining reactions related to nephrotoxicity and ototoxicity, and identifying other potential risk signals, such as allergic reactions and hematologic abnormalities. Our findings aim to provide data-driven support for safe medication practices in pediatric clinical settings.

While this study, utilizing the FAERS database, identified several significant AE signals associated with vancomycin in the pediatric population, it is crucial to carefully consider its inherent methodological limitations, particularly regarding the potential impacts of missing data, indication confounding, and concurrent medication on the interpretation of these signals.

Firstly, a substantial proportion of key variables in this study were missing (e.g., 77.60% of cases had unknown age, and 74.19% had unknown weight). This issue largely stems from the voluntary and nonmandatory nature of the self-reporting system. Although we included the “unknown” groups in our stratified analysis and conducted sensitivity analysis (comparing the consistency of signals between complete-information subgroups and the overall population, as presented in Fig. 2), we found that key signals, such as AKI, remained stable across both groups. This suggests that the main conclusions are relatively robust despite missing data. However, the high rate of missing information undeniably compromises the accuracy and statistical power of risk quantification for specific subgroups (e.g., different age and weight ranges). To mitigate this limitation, future prospective studies should systematically gather relevant demographic and clinical data. Secondly, the heterogeneity of indications poses a fundamental bias that cannot be entirely eliminated in retrospective analyses. Vancomycin is typically used to treat life-threatening drug-resistant bacterial infections (e.g., sepsis, complex skin, and soft tissue infections), and these severe conditions – along with the systemic inflammatory responses and hemodynamic instability they induce – are independent risk factors for AEs, including AKI.^[19,20]^ Consequently, the strong signal associations detected in this study (e.g., between vancomycin and AKI) may reflect, to a considerable extent, the high-risk clinical status of the patient population requiring vancomycin, rather than being solely attributed to the drug’s toxicity. This suggests that clinical interpretations of drug risk should be contextualized within the complex background of the underlying disease. Our findings serve as a crucial reminder: in critically ill pediatric patients requiring vancomycin, kidney injury is a clinical event that necessitates close monitoring and proactive management. This study utilized the FAERS database to systematically identify AE signals associated with vancomycin in pediatric populations; however, several limitations should be acknowledged: Firstly, as a voluntary reporting system, FAERS is prone to underreporting and reporting bias, where known AEs may be overreported, leading to stimulating reporting effects. This limitation precludes the accurate calculation of the true incidence rates of AEs. Secondly, controlling for confounding factors poses challenges. With regard to indication confounding, vancomycin is primarily used for severe infections, which themselves can contribute to kidney damage.^[21]^ Additionally, concomitant medication confounding is an issue, as pediatric patients often receive other nephrotoxic agents simultaneously, potentially resulting in an overestimation of the signal strength related to vancomycin. Furthermore, the study encountered high rates of missing data for key variables, specifically 53.8% for age and 64.4% for weight. Although sensitivity analyses indicated that the main signals were robust, subgroup analyses were less accurate. Moreover, the study lacked comprehensive clinical information, such as dosage and treatment duration. Additionally, it is important to note that disproportionality analyses performed in this study can indicate statistical associations but do not establish causality. Lastly, the majority of the data originated from the United States (49.49%), which necessitates caution when extrapolating findings to a global population. In conclusion, we acknowledge the limitations discussed above, which are primarily related to the inherent characteristics of the data sources used in this study. As such, our conclusions are more inclined toward generating hypotheses than confirming causal relationships. However, by employing a multi-algorithm fusion signal detection strategy and integrating subgroup analyses with exploratory sensitivity assessments, we aim to enhance the reliability of the identified signals. The safety signal spectrum provided by this study offers an important reference based on real-world data for optimizing clinical strategies related to vancomycin use in pediatrics, with a focus on critical monitoring for high-risk populations and timeframes. Ultimately, the clinical significance and causal relationships of these signals require further validation through rigorous prospective observational studies or targeted clinical trials.

It is essential to emphasize that this study is based on the FAERS, a spontaneous reporting database. The core value of such analyses lies in identifying drug-event combinations that exceed the expected frequency within the reports, thereby generating safety signals or hypotheses. However, these findings do not confirm causality or estimate the incidence of AEs. In this study, all statements regarding the “correlation,” “strong association,” or “high risk” of vancomycin with specific AEs (such as AKI and linear IgA disease) are interpreted precisely. Specifically, when vancomycin is documented as a suspected drug in the FAERS database, the proportion of reports corresponding to these AEs is significantly higher than the background reporting proportion of the event across all other drug reports. This “signal” may be influenced by various factors, including, but not limited to, the true toxicity of the drug, the underlying indications for its use, and confounding effects from co-administered medications (as previously discussed). Additionally, reporting bias may stem from heightened awareness among reporters (leading to overreporting of known adverse reactions, such as kidney injury) or from surges in reporting triggered by media or scholarly attention. Consequently, all conclusions drawn from this study should be interpreted with caution. Specifically, in pediatric patients, the use of vancomycin is associated with notable signals related to AEs, such as nephrotoxicity, cutaneous reactions, and rare immune disorders (e.g., linear IgA disease). These signals strongly indicate potential risk areas warranting prioritized investigation. For example, the finding that “renal toxicity represents the strongest signal in very low birth weight infants” should be understood as indicating the highest statistical association in this subgroup, thereby providing a critical basis for focused monitoring and targeted pharmacological research within this vulnerable population.

This study conducted subgroup analyses stratified by age, body weight, sex, and country, revealing variations in the distribution of reporting. Country differences: nearly half of the reports (49.49%) originated from the United States, reflecting its well-established pharmacovigilance system and reporting culture rather than a higher underlying risk. Reporter differences: pharmacists submitted the highest proportion of reports (40.21%), likely due to their professional responsibilities, such as TDM, which may result in an increased documentation of events such as nephrotoxicity and infusion reactions. Age and weight: the highest number of reports was observed in the 12 to 18 years and ≥45 kg subgroups, typically associated with clinical contexts such as surgeries, trauma, or acute exacerbations of chronic conditions in this age group. Conversely, the <1-year and <10-kg subgroups exhibited the strongest signal intensity for nephrotoxicity. This observation may indicate heightened pharmacokinetic vulnerability (e.g., immature renal function) rather than merely reflecting higher reporting frequencies. Sex: overall signal profiles were similar between males and females, with no clinically significant differences identified. Furthermore, variations in baseline disease severity across subgroups (e.g., low-weight infants in the intensive care unit compared to adolescents in general wards) are important confounding factors when interpreting observed signal variations. While these subgroup analyses illuminate populations and scenarios that may warrant closer attention, they do not facilitate causal comparisons. Differences in signal strength across subgroups should not be directly interpreted as differences in risk levels and require further validation through subsequent studies that control for confounding factors, such as disease severity.

This study is subject to several potential sources of bias. Reporting bias: the FAERS database relies on voluntary reporting, which can result in underreporting, delayed reporting, and selective reporting, such as the overreporting of known adverse reactions. Information bias: the high rate of missing data for key variables, such as age and weight, may impact the accuracy of the subgroup analyses. Monitoring bias: the intensity of monitoring for hospitalized patients, particularly in intensive care units, is typically higher, leading to a greater likelihood of detecting and recording events such as nephrotoxicity. Indication confounding: the underlying diseases that necessitate vancomycin treatment (e.g., sepsis) may themselves serve as independent risk factors for AEs. Concomitant medication confounding: pediatric patients frequently receive other nephrotoxic medications concurrently, complicating the attribution of AEs to a specific drug. These biases may lead to overestimation or underestimation of signal strength; thus, caution should be exercised when interpreting the findings.

In summary, FAERS signals function as “radar alerts” in drug safety monitoring, rather than as definitive “diagnoses.” They suggest directions for potential risks; however, their clinical significance, causal relationships, and actual incidence in real-world settings must be further confirmed or refuted through well-designed prospective epidemiological studies, cohort studies in specific populations, or mechanistic investigations. This study aims to provide data-driven, priority-focused insights to optimize the safety management of pediatric vancomycin, urging clinical practitioners to remain vigilant about the risks indicated by these signals throughout the medication process.

## 5. Conclusions

Vancomycin is significantly associated with nephrotoxicity, skin reactions, and rare immune-related conditions (such as linear IgA disease) in pediatric patients, particularly in low-birth-weight infants and adolescents. There is a critical need to enhance TDM and adopt individualized medication strategies, while also considering the impact of gender and geographic differences on AE reporting. This study provides valuable data to support the optimization of safety management for vancomycin use in children; however, it is essential to complement these findings with prospective research to further validate the clinical significance of the signals identified in the FAERS database.

## 6. Patents

### 6.1. Supplementary materials

The relevant materials mentioned below can be found in the attachment: Figure S1: The flow diagram of selecting vancomycin AEs from FAES database; Table S1: Four-quadrant analysis of vancomycin-associated adverse event signals based on disproportionality metrics; Table S2: Signal detection algorithms: formulae and thresholds; Table S3: Signal strength of adverse events associated with vancomycin at the SOC level by gender; Table S4: Signal strength of adverse events associated with vancomycin at the SOC level by age; Table S5: Signal strength of adverse events associated with vancomycin at the SOC level by weight classification; Table S6: Signal strength of adverse events associated with vancomycin at the PT level in the FAERS database; Table S7: Signal strength of adverse events associated with vancomycin at the PT level by gender; Table S8: Signal strength of adverse events associated with vancomycin at the PT level by age; Table S9: Signal strength of adverse events associated with vancomycin at the PT level by weight.

## Author Contributions

**Conceptualization:** Xue Li.

**Formal analysis:** Chaojie Zhang.

**Data curation:** Jianghong Hou.

**Figure:**