# Supplementary material for: Evaluating adverse reaction signals of vancomycin in pediatric patients: A FAERS database analysis
Source: Medicine (Baltimore). 2026 Jun 5;105(23):e49064. doi: 10.1097/MD.0000000000049064 (PMC13246103; doi:10.1097/MD.0000000000049064)
Supplement: Supplementary file 1 [file medi-105-e49064-s002.docx]

**Table S1** Four-Quadrant Analysis of Vancomycin-Associated Adverse Event Signals Based on Disproportionality Metrics

| Dimension | Target AE report number | Other AE reports | Total |
| --- | --- | --- | --- |
| Target drug | a | b | a+b |
| Other drugs | c | d | c+d |
| Total | a+c | b+d | N=a+b+c+d |
