# Supplementary material for: Evaluating adverse reaction signals of vancomycin in pediatric patients: A FAERS database analysis
Source: Medicine (Baltimore). 2026 Jun 5;105(23):e49064. doi: 10.1097/MD.0000000000049064 (PMC13246103; doi:10.1097/MD.0000000000049064)
Supplement: Supplementary file 3 [file medi-105-e49064-s004.docx]

**Table S4:**Signal Strength of Adverse Events Associated with Vancomycin at the SOC Level by Age.

| **SOC** | **Case Reports** | **ROR(95% CI)** | **PRR(95% CI)** | **chisq** | **IC(IC025)** | **EBGM(EBGM05)** | **group** |
| --- | --- | --- | --- | --- | --- | --- | --- |
| general disorders and administration site conditions | 2304 | 0.97(0.93, 1.01) | 0.98(0.94, 1.02) | 1.76 | -0.04(-0.1) | 0.98(0.94) | unknow |
| skin and subcutaneous tissue disorders | 1497 | 2.68(2.53, 2.83) | 2.45(2.36, 2.55) | 1360.81 | 1.29(1.22) | 2.45(2.34) | unknow |
| renal and urinary disorders | 1183 | 6.41(6.04, 6.81) | 5.85(5.52, 6.2) | 4822.1 | 2.54(2.46) | 5.83(5.54) | unknow |
| injury, poisoning and procedural complications | 842 | 0.65(0.6, 0.69) | 0.67(0.63, 0.71) | 151.51 | -0.57(-0.67) | 0.67(0.63) | unknow |
| infections and infestations | 867 | 1.62(1.51, 1.74) | 1.58(1.49, 1.68) | 191.42 | 0.66(0.56) | 1.57(1.49) | unknow |
| investigations | 676 | 1.05(0.97, 1.13) | 1.04(0.96, 1.12) | 1.24 | 0.06(-0.05) | 1.04(0.98) | unknow |
| blood and lymphatic system disorders | 480 | 3.3(3.02, 3.62) | 3.21(2.97, 3.47) | 737 | 1.68(1.55) | 3.2(2.97) | unknow |
| immune system disorders | 862 | 6.6(6.16, 7.08) | 6.17(5.82, 6.54) | 3769.87 | 2.62(2.52) | 6.15(5.81) | unknow |
| respiratory, thoracic and mediastinal disorders | 343 | 0.71(0.63, 0.79) | 0.72(0.65, 0.79) | 40.59 | -0.48(-0.64) | 0.72(0.65) | unknow |
| gastrointestinal disorders | 421 | 0.43(0.39, 0.48) | 0.45(0.41, 0.5) | 301.67 | -1.14(-1.28) | 0.45(0.42) | unknow |
| vascular disorders | 267 | 1.16(1.03, 1.31) | 1.16(1.03, 1.3) | 5.7 | 0.21(0.03) | 1.16(1.04) | unknow |
| nervous system disorders | 331 | 0.33(0.3, 0.37) | 0.35(0.32, 0.39) | 433.33 | -1.51(-1.67) | 0.35(0.32) | unknow |
| cardiac disorders | 204 | 0.79(0.69, 0.91) | 0.79(0.69, 0.91) | 11.05 | -0.33(-0.53) | 0.79(0.71) | unknow |
| metabolism and nutrition disorders | 116 | 0.55(0.46, 0.66) | 0.55(0.46, 0.66) | 42.34 | -0.85(-1.11) | 0.55(0.48) | unknow |
| hepatobiliary disorders | 182 | 2.44(2.11, 2.82) | 2.41(2.1, 2.76) | 151.6 | 1.27(1.06) | 2.41(2.13) | unknow |
| eye disorders | 318 | 1.25(1.12, 1.4) | 1.25(1.11, 1.41) | 15.74 | 0.32(0.16) | 1.25(1.13) | unknow |
| psychiatric disorders | 101 | 0.13(0.11, 0.16) | 0.14(0.12, 0.17) | 588.05 | -2.87(-3.15) | 0.14(0.12) | unknow |
| musculoskeletal and connective tissue disorders | 97 | 0.15(0.13, 0.19) | 0.16(0.13, 0.19) | 447.83 | -2.63(-2.92) | 0.16(0.14) | unknow |
| pregnancy, puerperium and perinatal conditions | 88 | 1.31(1.06, 1.62) | 1.31(1.06, 1.63) | 6.49 | 0.39(0.09) | 1.31(1.1) | unknow |
| ear and labyrinth disorders | 63 | 1.23(0.96, 1.57) | 1.22(0.95, 1.57) | 2.59 | 0.29(-0.06) | 1.22(0.99) | unknow |
| neoplasms benign, malignant and unspecified (incl cysts and polyps) | 24 | 0.07(0.04, 0.1) | 0.07(0.05, 0.1) | 310.76 | -3.86(-4.42) | 0.07(0.05) | unknow |
| congenital, familial and genetic disorders | 14 | 0.24(0.14, 0.41) | 0.24(0.14, 0.41) | 33.31 | -2.04(-2.77) | 0.24(0.16) | unknow |
| reproductive system and breast disorders | 6 | 0.06(0.03, 0.13) | 0.06(0.03, 0.13) | 94.54 | -4.13(-5.2) | 0.06(0.03) | unknow |
| general disorders and administration site conditions | 163 | 1.8(1.52, 2.14) | 1.67(1.46, 1.92) | 48.51 | 0.74(0.5) | 1.67(1.45) | <1 |
| skin and subcutaneous tissue disorders | 74 | 3.41(2.69, 4.33) | 3.23(2.6, 4.01) | 115.24 | 1.68(1.34) | 3.2(2.62) | <1 |
| renal and urinary disorders | 105 | 8.73(7.11, 10.72) | 7.91(6.63, 9.44) | 621.26 | 2.94(2.65) | 7.68(6.47) | <1 |
| injury, poisoning and procedural complications | 115 | 0.74(0.61, 0.9) | 0.77(0.65, 0.92) | 8.89 | -0.37(-0.65) | 0.77(0.66) | <1 |
| infections and infestations | 100 | 1.44(1.17, 1.77) | 1.39(1.14, 1.69) | 11.8 | 0.47(0.18) | 1.39(1.17) | <1 |
| investigations | 90 | 1.54(1.24, 1.91) | 1.49(1.22, 1.81) | 15.42 | 0.57(0.26) | 1.49(1.24) | <1 |
| blood and lymphatic system disorders | 42 | 1.89(1.39, 2.58) | 1.85(1.38, 2.48) | 16.79 | 0.89(0.44) | 1.85(1.43) | <1 |
| immune system disorders | 28 | 3.89(2.67, 5.69) | 3.81(2.63, 5.53) | 57.55 | 1.91(1.38) | 3.77(2.74) | <1 |
| respiratory, thoracic and mediastinal disorders | 53 | 0.59(0.45, 0.78) | 0.62(0.48, 0.8) | 13.81 | -0.7(-1.09) | 0.62(0.49) | <1 |
| gastrointestinal disorders | 35 | 0.66(0.47, 0.93) | 0.68(0.49, 0.95) | 5.74 | -0.56(-1.04) | 0.68(0.51) | <1 |
| vascular disorders | 42 | 2.08(1.53, 2.84) | 2.04(1.52, 2.74) | 22.5 | 1.02(0.58) | 2.03(1.57) | <1 |
| nervous system disorders | 34 | 0.48(0.34, 0.68) | 0.5(0.36, 0.7) | 18.07 | -0.99(-1.48) | 0.5(0.38) | <1 |
| cardiac disorders | 20 | 0.53(0.34, 0.82) | 0.54(0.35, 0.83) | 8.18 | -0.89(-1.51) | 0.54(0.37) | <1 |
| metabolism and nutrition disorders | 29 | 0.97(0.67, 1.4) | 0.97(0.68, 1.38) | 0.04 | -0.05(-0.57) | 0.97(0.71) | <1 |
| hepatobiliary disorders | 11 | 0.83(0.45, 1.5) | 0.83(0.46, 1.49) | 0.4 | -0.27(-1.1) | 0.83(0.5) | <1 |
| eye disorders | 21 | 1.94(1.26, 3) | 1.92(1.25, 2.96) | 9.31 | 0.94(0.32) | 1.91(1.33) | <1 |
| psychiatric disorders | 6 | 0.24(0.11, 0.53) | 0.24(0.11, 0.54) | 14.58 | -2.04(-3.11) | 0.24(0.12) | <1 |
| musculoskeletal and connective tissue disorders | 6 | 0.34(0.15, 0.77) | 0.35(0.16, 0.78) | 7.51 | -1.52(-2.6) | 0.35(0.18) | <1 |
| pregnancy, puerperium and perinatal conditions | 6 | 0.08(0.04, 0.18) | 0.09(0.04, 0.2) | 63.57 | -3.55(-4.62) | 0.09(0.04) | <1 |
| ear and labyrinth disorders | 6 | 2.3(1.03, 5.16) | 2.29(1.03, 5.11) | 4.35 | 1.19(0.11) | 2.28(1.16) | <1 |
| congenital, familial and genetic disorders | 4 | 0.04(0.01, 0.09) | 0.04(0.02, 0.11) | 104.77 | -4.66(-5.93) | 0.04(0.02) | <1 |
| general disorders and administration site conditions | 203 | 1.5(1.29, 1.75) | 1.41(1.25, 1.59) | 27.51 | 0.49(0.27) | 1.4(1.24) | 1~6 |
| skin and subcutaneous tissue disorders | 169 | 2.86(2.43, 3.38) | 2.57(2.24, 2.95) | 171.06 | 1.35(1.12) | 2.55(2.22) | 1~6 |
| renal and urinary disorders | 107 | 7.65(6.25, 9.35) | 6.97(5.84, 8.31) | 544.59 | 2.78(2.49) | 6.85(5.79) | 1~6 |
| injury, poisoning and procedural complications | 87 | 0.55(0.44, 0.68) | 0.58(0.48, 0.71) | 30.18 | -0.78(-1.09) | 0.58(0.49) | 1~6 |
| infections and infestations | 77 | 0.79(0.63, 0.99) | 0.8(0.64, 0.99) | 4.03 | -0.31(-0.65) | 0.8(0.66) | 1~6 |
| investigations | 74 | 1.01(0.8, 1.28) | 1.01(0.81, 1.25) | 0.01 | 0.01(-0.32) | 1.01(0.83) | 1~6 |
| blood and lymphatic system disorders | 71 | 2.07(1.63, 2.64) | 2(1.61, 2.48) | 36.47 | 1(0.65) | 1.99(1.63) | 1~6 |
| immune system disorders | 36 | 1.99(1.43, 2.78) | 1.96(1.43, 2.68) | 17.01 | 0.96(0.49) | 1.95(1.48) | 1~6 |
| respiratory, thoracic and mediastinal disorders | 45 | 0.69(0.51, 0.93) | 0.71(0.53, 0.95) | 5.83 | -0.5(-0.93) | 0.71(0.55) | 1~6 |
| gastrointestinal disorders | 34 | 0.41(0.29, 0.58) | 0.43(0.31, 0.6) | 27.5 | -1.21(-1.7) | 0.43(0.32) | 1~6 |
| vascular disorders | 22 | 1.02(0.67, 1.56) | 1.02(0.68, 1.54) | 0.01 | 0.03(-0.56) | 1.02(0.72) | 1~6 |
| nervous system disorders | 20 | 0.2(0.13, 0.31) | 0.21(0.14, 0.32) | 63.13 | -2.22(-2.84) | 0.21(0.15) | 1~6 |
| cardiac disorders | 39 | 1.79(1.3, 2.47) | 1.77(1.29, 2.42) | 13.15 | 0.82(0.36) | 1.76(1.35) | 1~6 |
| metabolism and nutrition disorders | 22 | 0.79(0.52, 1.2) | 0.79(0.52, 1.19) | 1.22 | -0.33(-0.93) | 0.79(0.56) | 1~6 |
| hepatobiliary disorders | 20 | 1.4(0.9, 2.19) | 1.4(0.91, 2.15) | 2.27 | 0.48(-0.14) | 1.39(0.96) | 1~6 |
| eye disorders | 13 | 0.62(0.36, 1.07) | 0.63(0.36, 1.09) | 2.96 | -0.67(-1.44) | 0.63(0.4) | 1~6 |
| psychiatric disorders | 6 | 0.09(0.04, 0.19) | 0.09(0.04, 0.2) | 58.18 | -3.46(-4.53) | 0.09(0.05) | 1~6 |
| general disorders and administration site conditions | 179 | 1.2(1.02, 1.41) | 1.16(1.01, 1.33) | 4.72 | 0.22(-0.01) | 1.16(1.01) | 6~12 |
| skin and subcutaneous tissue disorders | 149 | 2.44(2.05, 2.9) | 2.23(1.91, 2.61) | 107.38 | 1.15(0.9) | 2.22(1.92) | 6~12 |
| renal and urinary disorders | 111 | 8.18(6.7, 9.97) | 7.39(6.19, 8.82) | 613.05 | 2.87(2.58) | 7.29(6.18) | 6~12 |
| injury, poisoning and procedural complications | 121 | 0.87(0.72, 1.05) | 0.88(0.74, 1.05) | 2.2 | -0.18(-0.45) | 0.88(0.75) | 6~12 |
| infections and infestations | 81 | 1.27(1.01, 1.59) | 1.25(1.01, 1.55) | 4.19 | 0.32(-0.01) | 1.25(1.03) | 6~12 |
| investigations | 58 | 0.89(0.68, 1.16) | 0.89(0.69, 1.15) | 0.78 | -0.16(-0.54) | 0.89(0.72) | 6~12 |
| blood and lymphatic system disorders | 53 | 1.93(1.46, 2.54) | 1.88(1.46, 2.43) | 22.33 | 0.91(0.51) | 1.88(1.49) | 6~12 |
| immune system disorders | 37 | 2.18(1.57, 3.03) | 2.13(1.56, 2.91) | 22.59 | 1.09(0.62) | 2.13(1.62) | 6~12 |
| respiratory, thoracic and mediastinal disorders | 40 | 0.8(0.59, 1.1) | 0.81(0.59, 1.11) | 1.81 | -0.3(-0.75) | 0.81(0.62) | 6~12 |
| gastrointestinal disorders | 42 | 0.51(0.38, 0.7) | 0.53(0.39, 0.71) | 18.75 | -0.91(-1.35) | 0.53(0.41) | 6~12 |
| vascular disorders | 25 | 1.41(0.94, 2.09) | 1.4(0.95, 2.07) | 2.84 | 0.48(-0.08) | 1.39(1) | 6~12 |
| nervous system disorders | 35 | 0.36(0.26, 0.51) | 0.38(0.27, 0.53) | 38.12 | -1.38(-1.86) | 0.38(0.29) | 6~12 |
| cardiac disorders | 39 | 2.49(1.81, 3.44) | 2.44(1.78, 3.34) | 33.36 | 1.28(0.82) | 2.43(1.86) | 6~12 |
| metabolism and nutrition disorders | 14 | 0.54(0.32, 0.91) | 0.54(0.32, 0.92) | 5.48 | -0.88(-1.61) | 0.54(0.35) | 6~12 |
| hepatobiliary disorders | 12 | 1.1(0.62, 1.95) | 1.1(0.62, 1.94) | 0.11 | 0.14(-0.65) | 1.1(0.68) | 6~12 |
| eye disorders | 6 | 0.28(0.13, 0.63) | 0.29(0.13, 0.65) | 10.92 | -1.8(-2.88) | 0.29(0.15) | 6~12 |
| musculoskeletal and connective tissue disorders | 6 | 0.2(0.09, 0.46) | 0.21(0.09, 0.47) | 18.43 | -2.25(-3.33) | 0.21(0.11) | 6~12 |
| ear and labyrinth disorders | 4 | 1.45(0.54, 3.87) | 1.44(0.54, 3.84) | 0.55 | 0.53(-0.74) | 1.44(0.63) | 6~12 |
| general disorders and administration site conditions | 183 | 0.98(0.84, 1.15) | 0.99(0.86, 1.14) | 0.05 | -0.02(-0.24) | 0.99(0.86) | 12~18 |
| skin and subcutaneous tissue disorders | 280 | 1.71(1.5, 1.95) | 1.56(1.41, 1.72) | 65.29 | 0.64(0.46) | 1.56(1.4) | 12~18 |
| renal and urinary disorders | 102 | 5.74(4.69, 7.03) | 5.38(4.42, 6.54) | 366.06 | 2.42(2.13) | 5.35(4.51) | 12~18 |
| injury, poisoning and procedural complications | 79 | 0.39(0.31, 0.48) | 0.42(0.34, 0.52) | 72.69 | -1.25(-1.57) | 0.42(0.35) | 12~18 |
| infections and infestations | 96 | 1.57(1.27, 1.93) | 1.53(1.26, 1.86) | 18.38 | 0.61(0.31) | 1.53(1.28) | 12~18 |
| investigations | 112 | 1.46(1.21, 1.78) | 1.43(1.2, 1.71) | 15.13 | 0.51(0.23) | 1.43(1.21) | 12~18 |
| blood and lymphatic system disorders | 42 | 1.46(1.07, 1.98) | 1.44(1.07, 1.93) | 5.79 | 0.53(0.09) | 1.44(1.11) | 12~18 |
| immune system disorders | 88 | 5.05(4.06, 6.27) | 4.79(3.94, 5.83) | 265.23 | 2.25(1.94) | 4.76(3.97) | 12~18 |
| respiratory, thoracic and mediastinal disorders | 54 | 1.02(0.78, 1.34) | 1.02(0.79, 1.32) | 0.03 | 0.03(-0.36) | 1.02(0.81) | 12~18 |
| gastrointestinal disorders | 69 | 0.61(0.48, 0.78) | 0.63(0.5, 0.8) | 15.9 | -0.66(-1) | 0.63(0.52) | 12~18 |
| vascular disorders | 69 | 2.81(2.21, 3.58) | 2.72(2.15, 3.44) | 76.12 | 1.44(1.09) | 2.71(2.21) | 12~18 |
| nervous system disorders | 38 | 0.31(0.22, 0.42) | 0.33(0.24, 0.45) | 57.83 | -1.61(-2.07) | 0.33(0.25) | 12~18 |
| cardiac disorders | 38 | 1.44(1.04, 1.99) | 1.43(1.05, 1.96) | 4.98 | 0.51(0.05) | 1.43(1.09) | 12~18 |
| metabolism and nutrition disorders | 20 | 0.7(0.45, 1.08) | 0.7(0.45, 1.08) | 2.61 | -0.51(-1.13) | 0.7(0.48) | 12~18 |
| hepatobiliary disorders | 58 | 3.81(2.93, 4.96) | 3.69(2.86, 4.76) | 114.42 | 1.88(1.5) | 3.67(2.95) | 12~18 |
| eye disorders | 11 | 0.44(0.24, 0.79) | 0.44(0.24, 0.79) | 7.94 | -1.18(-2) | 0.44(0.27) | 12~18 |
| musculoskeletal and connective tissue disorders | 9 | 0.22(0.11, 0.43) | 0.23(0.12, 0.44) | 24.59 | -2.14(-3.04) | 0.23(0.13) | 12~18 |
| ear and labyrinth disorders | 7 | 1.93(0.92, 4.06) | 1.93(0.92, 4.06) | 3.12 | 0.94(-0.06) | 1.92(1.03) | 12~18 |

**Abbreviations:** SOC = system organ classe , ROR = Reporting Odds Ratio, PRR = Proportional Reporting Ratio,EBGM = Empirical Bayes Geometric Mean ,IC=Information Component
