# Supplementary material for: Evaluating adverse reaction signals of vancomycin in pediatric patients: A FAERS database analysis
Source: Medicine (Baltimore). 2026 Jun 5;105(23):e49064. doi: 10.1097/MD.0000000000049064 (PMC13246103; doi:10.1097/MD.0000000000049064)
Supplement: Supplementary file 5 [file medi-105-e49064-s006.docx]

**Table S6:**Signal Strength of Adverse Events Associated with Vancomycin at the PT Level in the FAERS Database.

| **SOC** | **PT** | **Case Reports** | **ROR(95% CI)** | **PRR(95% CI)** | **chisq** | **IC(IC025)** | **EBGM(EBGM05)** |
| --- | --- | --- | --- | --- | --- | --- | --- |
| renal and urinary disorders | acute kidney injury | 2454 | 19.16(18.39, 19.96) | 18.33(17.63, 19.06) | 39565.41 | 4.17(4.11) | 18.01(17.4) |
| immune system disorders | drug hypersensitivity | 1388 | 7.85(7.44, 8.28) | 7.67(7.23, 8.13) | 8017.06 | 2.93(2.85) | 7.62(7.29) |
| skin and subcutaneous tissue disorders | drug reaction with eosinophilia and systemic symptoms | 1242 | 59.87(56.5, 63.44) | 58.5(55.16, 62.04) | 66288.26 | 5.79(5.71) | 55.28(52.66) |
| renal and urinary disorders | nephropathy toxic | 655 | 72.8(67.22, 78.85) | 71.92(66.5, 77.79) | 42699.47 | 6.07(5.95) | 67.1(62.76) |
| skin and subcutaneous tissue disorders | linear iga disease | 575 | 651.74(586.5, 724.23) | 644.72(584.53, 711.1) | 223423.4 | 8.61(8.47) | 390.16(357.2) |
| skin and subcutaneous tissue disorders | erythema | 563 | 3.02(2.78, 3.28) | 3(2.77, 3.24) | 750.02 | 1.58(1.46) | 2.99(2.79) |
| renal and urinary disorders | renal tubular necrosis | 512 | 60.71(55.51, 66.4) | 60.14(54.53, 66.33) | 28067.65 | 5.83(5.7) | 56.74(52.64) |
| investigations | blood creatinine increased | 511 | 8.45(7.74, 9.22) | 8.38(7.75, 9.06) | 3294.57 | 3.06(2.93) | 8.31(7.73) |
| blood and lymphatic system disorders | thrombocytopenia | 493 | 4.88(4.47, 5.34) | 4.85(4.48, 5.25) | 1501.52 | 2.27(2.14) | 4.83(4.48) |
| general disorders and administration site conditions | treatment failure | 482 | 6.76(6.18, 7.4) | 6.71(6.08, 7.4) | 2329.57 | 2.74(2.61) | 6.67(6.19) |
| immune system disorders | anaphylactic reaction | 429 | 8.99(8.17, 9.89) | 8.93(8.1, 9.85) | 2995.8 | 3.15(3.01) | 8.86(8.18) |
| investigations | drug level increased | 417 | 27.94(25.34, 30.81) | 27.73(25.14, 30.59) | 10454.24 | 4.75(4.61) | 27(24.88) |
| renal and urinary disorders | renal failure | 398 | 3.14(2.84, 3.46) | 3.12(2.83, 3.44) | 572.9 | 1.64(1.5) | 3.11(2.87) |
| renal and urinary disorders | renal impairment | 396 | 5.28(4.78, 5.83) | 5.25(4.76, 5.79) | 1357.49 | 2.39(2.24) | 5.23(4.81) |
| blood and lymphatic system disorders | eosinophilia | 352 | 22.93(20.62, 25.49) | 22.78(20.65, 25.13) | 7167.24 | 4.48(4.33) | 22.29(20.4) |
| renal and urinary disorders | tubulointerstitial nephritis | 316 | 18.98(16.98, 21.22) | 18.87(16.78, 21.22) | 5250.18 | 4.21(4.05) | 18.54(16.89) |
| skin and subcutaneous tissue disorders | rash maculo-papular | 303 | 15.45(13.78, 17.31) | 15.36(13.66, 17.28) | 4007.86 | 3.92(3.76) | 15.14(13.77) |
| blood and lymphatic system disorders | leukopenia | 302 | 6.65(5.93, 7.44) | 6.61(5.88, 7.43) | 1430.58 | 2.72(2.55) | 6.58(5.98) |
| infections and infestations | clostridium difficile infection | 284 | 15.04(13.37, 16.91) | 14.96(13.3, 16.83) | 3646.57 | 3.88(3.71) | 14.75(13.37) |
| general disorders and administration site conditions | drug ineffective for unapproved indication | 266 | 5.48(4.85, 6.18) | 5.46(4.85, 6.14) | 963.37 | 2.44(2.27) | 5.43(4.91) |
| skin and subcutaneous tissue disorders | rash erythematous | 254 | 6.47(5.72, 7.32) | 6.44(5.73, 7.24) | 1161.64 | 2.68(2.5) | 6.41(5.78) |
| injury, poisoning and procedural complications | infusion related reaction | 246 | 4.24(3.74, 4.81) | 4.23(3.76, 4.76) | 604.39 | 2.08(1.89) | 4.21(3.79) |
| infections and infestations | septic shock | 239 | 6.2(5.46, 7.05) | 6.18(5.49, 6.95) | 1032.19 | 2.62(2.44) | 6.15(5.53) |
| general disorders and administration site conditions | multiple organ dysfunction syndrome | 239 | 10.37(9.13, 11.78) | 10.33(9.18, 11.62) | 1993.14 | 3.35(3.17) | 10.23(9.19) |
| skin and subcutaneous tissue disorders | toxic epidermal necrolysis | 234 | 17.49(15.36, 19.9) | 17.41(15.18, 19.97) | 3558.29 | 4.1(3.91) | 17.13(15.37) |
| skin and subcutaneous tissue disorders | acute generalised exanthematous pustulosis | 221 | 32.53(28.45, 37.21) | 32.4(28.25, 37.16) | 6512.69 | 4.97(4.78) | 31.4(28.07) |
| blood and lymphatic system disorders | pancytopenia | 214 | 4.25(3.71, 4.86) | 4.24(3.7, 4.86) | 527.42 | 2.08(1.88) | 4.22(3.77) |
| general disorders and administration site conditions | drug resistance | 208 | 9.06(7.9, 10.39) | 9.03(7.87, 10.36) | 1472.21 | 3.16(2.97) | 8.96(7.99) |
| skin and subcutaneous tissue disorders | drug eruption | 206 | 13.2(11.5, 15.15) | 13.16(11.47, 15.1) | 2283.91 | 3.7(3.5) | 13(11.58) |
| eye disorders | retinal vasculitis | 196 | 224.4(192.12, 262.1) | 223.58(191.13, 261.54) | 35400.96 | 7.51(7.29) | 182.42(160.2) |
| hepatobiliary disorders | drug-induced liver injury | 194 | 7.74(6.72, 8.92) | 7.72(6.73, 8.86) | 1125.89 | 2.94(2.73) | 7.66(6.81) |
| infections and infestations | staphylococcal infection | 191 | 6.18(5.36, 7.13) | 6.17(5.38, 7.08) | 822.01 | 2.62(2.41) | 6.13(5.44) |
| skin and subcutaneous tissue disorders | stevens-johnson syndrome | 184 | 8.93(7.73, 10.33) | 8.91(7.77, 10.22) | 1280.49 | 3.14(2.93) | 8.84(7.82) |
| skin and subcutaneous tissue disorders | rash pruritic | 173 | 3.62(3.12, 4.2) | 3.61(3.09, 4.22) | 325.46 | 1.85(1.63) | 3.6(3.18) |
| skin and subcutaneous tissue disorders | vancomycin infusion reaction | 155 | 1355.84(1063.78, 1728.1) | 1351.91(1068.57, 1710.38) | 88222.98 | 9.16(8.87) | 570.6(465.77) |
| infections and infestations | pathogen resistance | 153 | 18.78(16, 22.04) | 18.73(16.01, 21.91) | 2519.66 | 4.2(3.97) | 18.39(16.09) |
| skin and subcutaneous tissue disorders | blister | 153 | 3.04(2.6, 3.57) | 3.04(2.6, 3.56) | 208.54 | 1.6(1.37) | 3.03(2.65) |
| investigations | antibiotic level above therapeutic | 143 | 807.53(647.34, 1007.35) | 805.36(649.17, 999.13) | 63221.1 | 8.79(8.51) | 443.65(368.72) |
| skin and subcutaneous tissue disorders | toxic skin eruption | 141 | 15.43(13.06, 18.23) | 15.39(13.16, 18) | 1868.71 | 3.92(3.68) | 15.17(13.2) |
| skin and subcutaneous tissue disorders | angioedema | 133 | 3.32(2.8, 3.93) | 3.31(2.77, 3.95) | 213.98 | 1.72(1.48) | 3.3(2.86) |
| infections and infestations | enterococcal infection | 126 | 29.66(24.84, 35.41) | 29.59(24.8, 35.3) | 3379.26 | 4.85(4.59) | 28.76(24.79) |
| immune system disorders | anaphylactic shock | 125 | 5.52(4.63, 6.58) | 5.51(4.62, 6.57) | 458.51 | 2.45(2.2) | 5.48(4.73) |
| respiratory, thoracic and mediastinal disorders | hypoxia | 118 | 3.71(3.1, 4.45) | 3.71(3.11, 4.43) | 232.67 | 1.89(1.63) | 3.7(3.18) |
| general disorders and administration site conditions | face oedema | 112 | 7.11(5.9, 8.56) | 7.09(5.94, 8.46) | 582.22 | 2.82(2.55) | 7.05(6.03) |
| vascular disorders | shock | 109 | 5.34(4.42, 6.44) | 5.33(4.38, 6.48) | 381.24 | 2.41(2.14) | 5.3(4.53) |
| blood and lymphatic system disorders | leukocytosis | 108 | 6.57(5.44, 7.95) | 6.56(5.39, 7.98) | 506.08 | 2.71(2.43) | 6.53(5.57) |
| hepatobiliary disorders | cholestasis | 108 | 6.28(5.2, 7.59) | 6.27(5.15, 7.63) | 475.5 | 2.64(2.37) | 6.24(5.32) |
| blood and lymphatic system disorders | agranulocytosis | 107 | 6.59(5.45, 7.97) | 6.58(5.41, 8) | 502.65 | 2.71(2.44) | 6.54(5.58) |
| blood and lymphatic system disorders | lymphadenopathy | 103 | 3.16(2.6, 3.84) | 3.16(2.6, 3.84) | 151.35 | 1.66(1.38) | 3.15(2.68) |
| skin and subcutaneous tissue disorders | erythema multiforme | 102 | 11.96(9.83, 14.54) | 11.94(9.81, 14.53) | 1009.87 | 3.56(3.28) | 11.8(10.02) |
| infections and infestations | candida infection | 102 | 7.06(5.81, 8.58) | 7.05(5.8, 8.58) | 525.73 | 2.81(2.53) | 7(5.95) |
| investigations | transaminases increased | 101 | 4.95(4.07, 6.02) | 4.95(4.07, 6.02) | 316.46 | 2.3(2.02) | 4.93(4.18) |
| renal and urinary disorders | nephropathy | 101 | 11.42(9.38, 13.9) | 11.4(9.37, 13.87) | 947.45 | 3.5(3.21) | 11.28(9.57) |
| skin and subcutaneous tissue disorders | rash morbilliform | 100 | 41.86(34.26, 51.14) | 41.78(34.34, 50.83) | 3818.68 | 5.33(5.04) | 40.12(33.93) |
| nervous system disorders | encephalopathy | 96 | 4.32(3.54, 5.28) | 4.32(3.55, 5.26) | 243.64 | 2.1(1.82) | 4.3(3.64) |
| cardiac disorders | kounis syndrome | 89 | 47.93(38.74, 59.31) | 47.85(38.57, 59.36) | 3893.97 | 5.51(5.21) | 45.68(38.23) |
| investigations | blood urea increased | 88 | 5.45(4.42, 6.72) | 5.44(4.38, 6.75) | 317.62 | 2.44(2.14) | 5.42(4.55) |
| respiratory, thoracic and mediastinal disorders | respiratory distress | 88 | 3.46(2.8, 4.26) | 3.45(2.78, 4.28) | 152.95 | 1.78(1.48) | 3.45(2.89) |
| investigations | drug level above therapeutic | 87 | 22.01(17.8, 27.23) | 21.98(17.72, 27.27) | 1704.38 | 4.43(4.12) | 21.52(18.01) |
| infections and infestations | clostridium difficile colitis | 86 | 8.89(7.19, 11) | 8.88(7.16, 11.02) | 595.95 | 3.14(2.83) | 8.81(7.37) |
| blood and lymphatic system disorders | immune thrombocytopenia | 84 | 24.89(20.04, 30.91) | 24.85(20.03, 30.83) | 1875.94 | 4.6(4.29) | 24.27(20.24) |
| respiratory, thoracic and mediastinal disorders | acute respiratory distress syndrome | 83 | 5.11(4.12, 6.34) | 5.1(4.11, 6.33) | 272.27 | 2.34(2.04) | 5.08(4.24) |
| vascular disorders | haemodynamic instability | 83 | 12.66(10.19, 15.72) | 12.64(10.19, 15.68) | 878.34 | 3.64(3.33) | 12.49(10.42) |
| nervous system disorders | unresponsive to stimuli | 83 | 3.55(2.86, 4.41) | 3.55(2.86, 4.4) | 151.41 | 1.82(1.51) | 3.54(2.95) |
| hepatobiliary disorders | hepatocellular injury | 81 | 6.02(4.84, 7.49) | 6.01(4.84, 7.46) | 336.37 | 2.58(2.27) | 5.98(4.98) |
| renal and urinary disorders | oliguria | 80 | 13.98(11.21, 17.44) | 13.96(11.25, 17.32) | 949.39 | 3.78(3.47) | 13.78(11.46) |
| blood and lymphatic system disorders | disseminated intravascular coagulation | 74 | 5.57(4.43, 7) | 5.57(4.4, 7.05) | 275.64 | 2.47(2.14) | 5.54(4.58) |
| injury, poisoning and procedural complications | incorrect drug administration rate | 73 | 20.26(16.07, 25.55) | 20.24(16, 25.61) | 1308.24 | 4.31(3.98) | 19.85(16.35) |
| hepatobiliary disorders | hepatitis | 73 | 3.13(2.49, 3.94) | 3.13(2.47, 3.96) | 105.28 | 1.64(1.31) | 3.12(2.57) |
| hepatobiliary disorders | liver injury | 72 | 3.67(2.91, 4.62) | 3.66(2.89, 4.63) | 139.05 | 1.87(1.54) | 3.65(3.01) |
| vascular disorders | circulatory collapse | 68 | 4.22(3.33, 5.36) | 4.22(3.34, 5.34) | 166.33 | 2.07(1.73) | 4.21(3.44) |
| eye disorders | retinal vascular occlusion | 68 | 110.73(86.17, 142.3) | 110.59(85.72, 142.68) | 6639.99 | 6.64(6.28) | 99.54(80.69) |
| renal and urinary disorders | anuria | 66 | 8.12(6.37, 10.34) | 8.11(6.41, 10.26) | 408.02 | 3.01(2.66) | 8.05(6.57) |
| skin and subcutaneous tissue disorders | dermatitis bullous | 64 | 9.85(7.7, 12.6) | 9.84(7.63, 12.7) | 503.14 | 3.29(2.93) | 9.75(7.93) |
| infections and infestations | bacteraemia | 64 | 6.14(4.8, 7.85) | 6.14(4.76, 7.92) | 273.48 | 2.61(2.26) | 6.1(4.97) |
| skin and subcutaneous tissue disorders | purpura | 64 | 7.76(6.07, 9.92) | 7.75(6.01, 10) | 373.44 | 2.94(2.59) | 7.7(6.27) |
| hepatobiliary disorders | hepatotoxicity | 64 | 3.23(2.52, 4.12) | 3.22(2.5, 4.15) | 97.82 | 1.68(1.33) | 3.22(2.62) |
| infections and infestations | staphylococcal bacteraemia | 63 | 16.71(13.02, 21.43) | 16.69(12.94, 21.53) | 913.65 | 4.04(3.68) | 16.43(13.33) |
| infections and infestations | endocarditis | 61 | 13.05(10.13, 16.8) | 13.03(10.1, 16.81) | 668.9 | 3.69(3.32) | 12.88(10.42) |
| skin and subcutaneous tissue disorders | petechiae | 61 | 6.43(5, 8.27) | 6.42(4.98, 8.28) | 277.55 | 2.68(2.31) | 6.39(5.17) |
| immune system disorders | type iv hypersensitivity reaction | 60 | 24.98(19.33, 32.28) | 24.95(19.34, 32.19) | 1345.49 | 4.61(4.24) | 24.36(19.66) |
| respiratory, thoracic and mediastinal disorders | bronchospasm | 56 | 4.2(3.23, 5.46) | 4.19(3.25, 5.41) | 135.57 | 2.06(1.69) | 4.18(3.35) |
| eye disorders | glaucoma | 56 | 3.16(2.43, 4.11) | 3.16(2.45, 4.08) | 82.45 | 1.66(1.28) | 3.15(2.53) |
| ear and labyrinth disorders | ototoxicity | 53 | 32.34(24.59, 42.52) | 32.3(24.55, 42.5) | 1556.79 | 4.97(4.58) | 31.31(24.9) |
| infections and infestations | systemic candida | 53 | 25.77(19.62, 33.85) | 25.74(19.56, 33.87) | 1228.49 | 4.65(4.26) | 25.11(19.99) |
| blood and lymphatic system disorders | haemolytic anaemia | 52 | 6.28(4.78, 8.25) | 6.27(4.77, 8.25) | 229.02 | 2.64(2.25) | 6.24(4.97) |
| eye disorders | retinal haemorrhage | 51 | 7.86(5.96, 10.35) | 7.85(5.97, 10.33) | 302.55 | 2.96(2.57) | 7.8(6.19) |
| skin and subcutaneous tissue disorders | dermatitis exfoliative | 50 | 10.24(7.75, 13.54) | 10.23(7.78, 13.46) | 412.37 | 3.34(2.94) | 10.14(8.03) |
| infections and infestations | klebsiella infection | 50 | 11.3(8.55, 14.94) | 11.29(8.58, 14.85) | 463.76 | 3.48(3.08) | 11.18(8.85) |
| skin and subcutaneous tissue disorders | henoch-schonlein purpura | 50 | 27.33(20.63, 36.2) | 27.3(20.75, 35.92) | 1232.8 | 4.73(4.33) | 26.59(21.02) |
| vascular disorders | haemorrhagic vasculitis | 49 | 721.46(499.08, 1042.94) | 720.8(496.69, 1046.03) | 20343.37 | 8.7(8.23) | 416.75(306.16) |
| investigations | eosinophil count increased | 48 | 6.33(4.76, 8.41) | 6.32(4.8, 8.32) | 213.75 | 2.65(2.25) | 6.29(4.96) |
| skin and subcutaneous tissue disorders | hypersensitivity vasculitis | 44 | 17.24(12.79, 23.22) | 17.22(12.83, 23.11) | 660.82 | 4.08(3.66) | 16.94(13.2) |
| investigations | drug level below therapeutic | 43 | 8.72(6.46, 11.78) | 8.72(6.5, 11.7) | 291.27 | 3.11(2.68) | 8.65(6.73) |
| skin and subcutaneous tissue disorders | cutaneous vasculitis | 43 | 15.8(11.69, 21.36) | 15.79(11.77, 21.19) | 586.33 | 3.96(3.53) | 15.56(12.09) |
| immune system disorders | haemophagocytic lymphohistiocytosis | 41 | 7.43(5.47, 10.11) | 7.43(5.43, 10.17) | 226.39 | 2.88(2.45) | 7.38(5.71) |
| general disorders and administration site conditions | generalised oedema | 40 | 3.59(2.63, 4.89) | 3.59(2.62, 4.91) | 74.31 | 1.84(1.4) | 3.58(2.76) |
| immune system disorders | anaphylactoid reaction | 40 | 10.58(7.75, 14.45) | 10.57(7.72, 14.46) | 343.12 | 3.39(2.94) | 10.47(8.07) |
| renal and urinary disorders | nephritis | 39 | 13.31(9.71, 18.26) | 13.3(9.72, 18.2) | 437.93 | 3.72(3.27) | 13.14(10.09) |
| renal and urinary disorders | renal tubular disorder | 39 | 14.6(10.64, 20.03) | 14.59(10.66, 19.96) | 486.41 | 3.85(3.4) | 14.39(11.04) |
| general disorders and administration site conditions | extravasation | 36 | 9.42(6.79, 13.08) | 9.42(6.75, 13.14) | 268.27 | 3.22(2.76) | 9.34(7.09) |
| skin and subcutaneous tissue disorders | dermatitis exfoliative generalised | 36 | 12.84(9.24, 17.83) | 12.83(9.19, 17.9) | 387.58 | 3.66(3.2) | 12.68(9.63) |
| skin and subcutaneous tissue disorders | skin necrosis | 34 | 7.02(5.01, 9.84) | 7.02(5.03, 9.8) | 174.18 | 2.8(2.32) | 6.97(5.26) |
| respiratory, thoracic and mediastinal disorders | pulmonary haemorrhage | 34 | 4.58(3.27, 6.42) | 4.58(3.28, 6.39) | 94.79 | 2.19(1.71) | 4.57(3.44) |
| investigations | urine output decreased | 33 | 4(2.84, 5.64) | 4(2.87, 5.58) | 74.05 | 2(1.51) | 3.99(3) |
| general disorders and administration site conditions | multiple-drug resistance | 33 | 12.55(8.9, 17.69) | 12.54(8.81, 17.85) | 346.17 | 3.63(3.14) | 12.4(9.3) |
| skin and subcutaneous tissue disorders | skin plaque | 32 | 4.1(2.9, 5.81) | 4.1(2.88, 5.83) | 74.77 | 2.03(1.54) | 4.09(3.06) |
| cardiac disorders | pulseless electrical activity | 32 | 8.73(6.16, 12.36) | 8.72(6.13, 12.41) | 216.94 | 3.11(2.62) | 8.66(6.47) |
| infections and infestations | pseudomonas infection | 31 | 4.15(2.92, 5.91) | 4.15(2.92, 5.91) | 73.76 | 2.05(1.55) | 4.13(3.08) |
| infections and infestations | acinetobacter infection | 30 | 31.47(21.88, 45.27) | 31.45(21.67, 45.64) | 857.26 | 4.93(4.42) | 30.51(22.51) |
| investigations | drug level decreased | 30 | 3.22(2.25, 4.61) | 3.22(2.26, 4.58) | 45.78 | 1.68(1.18) | 3.21(2.38) |
| investigations | pulse absent | 30 | 6.66(4.65, 9.53) | 6.65(4.67, 9.46) | 143.11 | 2.73(2.22) | 6.61(4.9) |
| blood and lymphatic system disorders | thrombotic microangiopathy | 30 | 3.56(2.49, 5.1) | 3.56(2.5, 5.07) | 55 | 1.83(1.32) | 3.55(2.63) |
| nervous system disorders | posterior reversible encephalopathy syndrome | 30 | 3.58(2.5, 5.13) | 3.58(2.52, 5.09) | 55.67 | 1.84(1.33) | 3.57(2.65) |
| gastrointestinal disorders | noninfectious peritonitis | 29 | 146.65(99.27, 216.64) | 146.57(99.04, 216.91) | 3650.04 | 7(6.45) | 127.73(92.15) |
| hepatobiliary disorders | venoocclusive liver disease | 29 | 6.58(4.57, 9.49) | 6.58(4.53, 9.55) | 136.32 | 2.71(2.19) | 6.54(4.82) |
| general disorders and administration site conditions | infusion site extravasation | 28 | 4.88(3.37, 7.07) | 4.88(3.36, 7.08) | 85.89 | 2.28(1.75) | 4.86(3.56) |
| investigations | antibiotic level below therapeutic | 28 | 394.44(254.45, 611.44) | 394.23(256.14, 606.76) | 7844.74 | 8.14(7.54) | 281.88(195.33) |
| renal and urinary disorders | renal tubular injury | 28 | 31.85(21.86, 46.4) | 31.83(21.93, 46.19) | 809.97 | 4.95(4.41) | 30.87(22.53) |
| respiratory, thoracic and mediastinal disorders | eosinophilic pneumonia | 27 | 9.91(6.78, 14.48) | 9.91(6.83, 14.38) | 214.08 | 3.3(2.76) | 9.82(7.15) |
| general disorders and administration site conditions | hyperthermia | 27 | 3.85(2.64, 5.62) | 3.85(2.65, 5.59) | 56.68 | 1.94(1.4) | 3.84(2.8) |
| investigations | inflammatory marker increased | 27 | 6.41(4.39, 9.35) | 6.4(4.41, 9.29) | 122.31 | 2.67(2.13) | 6.37(4.64) |
| skin and subcutaneous tissue disorders | symmetrical drug-related intertriginous and flexural exanthema | 27 | 41.93(28.52, 61.63) | 41.91(28.32, 62.02) | 1034.17 | 5.33(4.78) | 40.24(29.15) |
| eye disorders | periorbital oedema | 26 | 5.48(3.73, 8.06) | 5.48(3.7, 8.11) | 94.77 | 2.45(1.9) | 5.46(3.95) |
| infections and infestations | aspergillus infection | 26 | 4.59(3.12, 6.75) | 4.59(3.1, 6.79) | 72.59 | 2.19(1.65) | 4.57(3.31) |
| hepatobiliary disorders | hypertransaminasaemia | 26 | 4.9(3.34, 7.21) | 4.9(3.31, 7.25) | 80.38 | 2.29(1.74) | 4.88(3.54) |
| hepatobiliary disorders | hepatic cytolysis | 26 | 3.12(2.12, 4.58) | 3.12(2.11, 4.62) | 37.27 | 1.64(1.09) | 3.11(2.25) |
| general disorders and administration site conditions | systemic inflammatory response syndrome | 25 | 7.11(4.79, 10.53) | 7.1(4.8, 10.51) | 130.16 | 2.82(2.26) | 7.06(5.08) |
| infections and infestations | rash pustular | 25 | 3.57(2.41, 5.29) | 3.57(2.41, 5.28) | 46.17 | 1.83(1.28) | 3.56(2.57) |
| blood and lymphatic system disorders | haemolysis | 25 | 3.52(2.37, 5.21) | 3.51(2.37, 5.19) | 44.82 | 1.81(1.25) | 3.51(2.52) |
| immune system disorders | type i hypersensitivity | 24 | 8.27(5.54, 12.37) | 8.27(5.59, 12.24) | 152.13 | 3.04(2.47) | 8.21(5.87) |
| infections and infestations | escherichia infection | 24 | 3.31(2.22, 4.94) | 3.31(2.24, 4.9) | 38.54 | 1.72(1.16) | 3.3(2.36) |
| gastrointestinal disorders | megacolon | 24 | 17.31(11.56, 25.92) | 17.3(11.46, 26.11) | 362.32 | 4.09(3.52) | 17.02(12.14) |
| investigations | blood creatine increased | 24 | 5.87(3.93, 8.77) | 5.87(3.97, 8.69) | 96.36 | 2.55(1.98) | 5.84(4.17) |
| infections and infestations | stenotrophomonas infection | 24 | 20.94(13.98, 31.38) | 20.93(13.87, 31.59) | 446.07 | 4.36(3.79) | 20.52(14.63) |
| renal and urinary disorders | myeloma cast nephropathy | 24 | 236.65(151.56, 369.5) | 236.54(150.7, 371.27) | 4539.58 | 7.58(6.96) | 190.95(131.52) |
| ear and labyrinth disorders | deafness neurosensory | 23 | 7.95(5.27, 11.98) | 7.95(5.27, 12) | 138.53 | 2.98(2.4) | 7.89(5.6) |
| infections and infestations | enterococcal bacteraemia | 23 | 25.28(16.71, 38.25) | 25.27(16.74, 38.14) | 522.76 | 4.62(4.04) | 24.66(17.44) |
| renal and urinary disorders | azotaemia | 23 | 6.43(4.27, 9.69) | 6.43(4.26, 9.7) | 104.73 | 2.68(2.1) | 6.39(4.54) |
| general disorders and administration site conditions | localised oedema | 22 | 5.1(3.35, 7.75) | 5.09(3.37, 7.68) | 72.01 | 2.34(1.75) | 5.07(3.57) |
| gastrointestinal disorders | lip oedema | 22 | 5.6(3.68, 8.52) | 5.6(3.71, 8.45) | 82.69 | 2.48(1.89) | 5.58(3.93) |
| infections and infestations | mucormycosis | 22 | 9.59(6.3, 14.6) | 9.59(6.35, 14.47) | 167.65 | 3.25(2.66) | 9.51(6.69) |
| gastrointestinal disorders | enterocolitis haemorrhagic | 22 | 15.59(10.23, 23.76) | 15.59(10.33, 23.53) | 295.68 | 3.94(3.35) | 15.36(10.8) |
| immune system disorders | type iii immune complex mediated reaction | 22 | 46.65(30.41, 71.55) | 46.63(30.3, 71.77) | 937.96 | 5.48(4.87) | 44.57(31.16) |
| gastrointestinal disorders | dysbiosis | 22 | 38.39(25.07, 58.78) | 38.38(24.94, 59.07) | 770.85 | 5.21(4.61) | 36.98(25.89) |
| infections and infestations | clostridial infection | 21 | 6.28(4.09, 9.65) | 6.28(4.08, 9.67) | 92.67 | 2.64(2.04) | 6.25(4.36) |
| infections and infestations | septic embolus | 21 | 27.42(17.77, 42.31) | 27.41(17.81, 42.19) | 520 | 4.74(4.13) | 26.7(18.57) |
| ear and labyrinth disorders | vestibular disorder | 21 | 18.62(12.09, 28.67) | 18.61(12.09, 28.64) | 343.51 | 4.19(3.58) | 18.29(12.74) |
| blood and lymphatic system disorders | haemorrhagic disorder | 21 | 20.81(13.51, 32.06) | 20.8(13.51, 32.01) | 387.66 | 4.35(3.74) | 20.39(14.2) |
| infections and infestations | pseudomembranous colitis | 21 | 9.99(6.5, 15.35) | 9.98(6.48, 15.36) | 168.08 | 3.31(2.7) | 9.89(6.9) |
| injury, poisoning and procedural complications | product label confusion | 21 | 4.25(2.77, 6.52) | 4.24(2.75, 6.53) | 51.85 | 2.08(1.48) | 4.23(2.95) |
| infections and infestations | cardiac valve vegetation | 20 | 45.54(29.09, 71.3) | 45.52(29, 71.45) | 832.47 | 5.44(4.81) | 43.56(29.93) |
| infections and infestations | enterobacter infection | 20 | 13.07(8.41, 20.31) | 13.06(8.32, 20.5) | 219.88 | 3.69(3.07) | 12.9(8.92) |
| general disorders and administration site conditions | necrosis | 20 | 3.84(2.48, 5.96) | 3.84(2.49, 5.91) | 41.85 | 1.94(1.32) | 3.83(2.65) |
| infections and infestations | human herpesvirus 6 infection | 20 | 11.36(7.31, 17.65) | 11.35(7.37, 17.47) | 186.71 | 3.49(2.87) | 11.24(7.77) |
| infections and infestations | staphylococcal sepsis | 19 | 4.03(2.57, 6.32) | 4.03(2.57, 6.33) | 43.08 | 2.01(1.37) | 4.02(2.75) |
| skin and subcutaneous tissue disorders | skin erosion | 19 | 6.8(4.33, 10.68) | 6.8(4.33, 10.67) | 93.39 | 2.76(2.12) | 6.76(4.64) |
| blood and lymphatic system disorders | neutrophilia | 19 | 3.77(2.4, 5.92) | 3.77(2.4, 5.92) | 38.55 | 1.91(1.28) | 3.76(2.58) |
| eye disorders | retinal ischaemia | 19 | 35.89(22.7, 56.72) | 35.87(22.85, 56.3) | 621.52 | 5.11(4.47) | 34.65(23.62) |
| infections and infestations | pneumonia staphylococcal | 18 | 9.9(6.22, 15.75) | 9.89(6.18, 15.83) | 142.49 | 3.29(2.64) | 9.81(6.65) |
| respiratory, thoracic and mediastinal disorders | laryngeal oedema | 18 | 3.17(2, 5.03) | 3.17(1.98, 5.07) | 26.63 | 1.66(1.01) | 3.16(2.15) |
| infections and infestations | fungaemia | 18 | 13.1(8.23, 20.85) | 13.09(8.18, 20.95) | 198.41 | 3.69(3.04) | 12.93(8.76) |
| blood and lymphatic system disorders | autoimmune haemolytic anaemia | 18 | 6.21(3.91, 9.87) | 6.21(3.88, 9.94) | 78.14 | 2.63(1.98) | 6.17(4.19) |
| ear and labyrinth disorders | deafness unilateral | 17 | 3.39(2.11, 5.46) | 3.39(2.12, 5.43) | 28.53 | 1.76(1.09) | 3.38(2.27) |
| renal and urinary disorders | renal tubular atrophy | 17 | 21.06(13.02, 34.04) | 21.05(12.9, 34.36) | 317.86 | 4.37(3.69) | 20.63(13.8) |
| infections and infestations | fungal peritonitis | 17 | 11.89(7.37, 19.19) | 11.89(7.43, 19.03) | 167.57 | 3.56(2.89) | 11.76(7.88) |
| vascular disorders | hyperaemia | 17 | 9.46(5.87, 15.26) | 9.46(5.91, 15.14) | 127.41 | 3.23(2.56) | 9.38(6.29) |
| ear and labyrinth disorders | deafness bilateral | 17 | 11.28(6.99, 18.19) | 11.28(7.05, 18.06) | 157.39 | 3.48(2.81) | 11.16(7.48) |
| metabolism and nutrition disorders | hypernatraemia | 16 | 3.53(2.16, 5.77) | 3.53(2.16, 5.76) | 28.91 | 1.82(1.13) | 3.52(2.33) |
| skin and subcutaneous tissue disorders | rash vesicular | 16 | 3.78(2.32, 6.18) | 3.78(2.32, 6.17) | 32.63 | 1.92(1.23) | 3.77(2.5) |
| general disorders and administration site conditions | potentiating drug interaction | 16 | 5.49(3.36, 8.97) | 5.49(3.36, 8.96) | 58.41 | 2.45(1.76) | 5.46(3.62) |
| infections and infestations | liver abscess | 16 | 5.57(3.4, 9.1) | 5.56(3.41, 9.08) | 59.57 | 2.47(1.78) | 5.54(3.67) |
| infections and infestations | arthritis bacterial | 16 | 4.26(2.61, 6.97) | 4.26(2.61, 6.95) | 39.78 | 2.09(1.4) | 4.25(2.82) |
| injury, poisoning and procedural complications | drug monitoring procedure not performed | 16 | 10.28(6.28, 16.82) | 10.27(6.29, 16.76) | 132.55 | 3.35(2.66) | 10.18(6.74) |
| renal and urinary disorders | kidney fibrosis | 16 | 9.96(6.09, 16.31) | 9.96(6.1, 16.26) | 127.71 | 3.3(2.61) | 9.87(6.54) |
| nervous system disorders | metabolic encephalopathy | 15 | 5.45(3.28, 9.06) | 5.45(3.27, 9.07) | 54.22 | 2.44(1.73) | 5.43(3.55) |
| skin and subcutaneous tissue disorders | exfoliative rash | 15 | 6.31(3.8, 10.49) | 6.31(3.79, 10.5) | 66.6 | 2.65(1.94) | 6.28(4.1) |
| investigations | creatinine renal clearance decreased | 15 | 3.83(2.31, 6.36) | 3.83(2.3, 6.38) | 31.26 | 1.93(1.23) | 3.82(2.5) |
| infections and infestations | sepsis neonatal | 15 | 20.01(12, 33.36) | 20.01(12.02, 33.31) | 265.44 | 4.29(3.58) | 19.63(12.8) |

**Abbreviations:** SOC = system organ classe , ROR = Reporting Odds Ratio, PRR = Proportional Reporting Ratio,EBGM = Empirical Bayes Geometric Mean ,IC=Information Component
