# Supplementary material for: Evaluating adverse reaction signals of vancomycin in pediatric patients: A FAERS database analysis
Source: Medicine (Baltimore). 2026 Jun 5;105(23):e49064. doi: 10.1097/MD.0000000000049064 (PMC13246103; doi:10.1097/MD.0000000000049064)
Supplement: Supplementary file 6 [file medi-105-e49064-s007.docx]

**Table S7:**Signal Strength of Adverse Events Associated with Vancomycin at the PT Level by Gender.

| **SOC** | **PT** | **Case Reports** | **ROR(95% CI)** | **PRR(95% CI)** | **chisq** | **IC(IC025)** | **EBGM(EBGM05)** | **group** |
| --- | --- | --- | --- | --- | --- | --- | --- | --- |
| renal and urinary disorders | acute kidney injury | 812 | 23.33(21.74, 25.04) | 22.47(21.19, 23.83) | 16423.01 | 4.47(4.37) | 22.13(20.86) | female |
| immune system disorders | drug hypersensitivity | 784 | 9.48(8.83, 10.19) | 9.17(8.48, 9.92) | 5691.39 | 3.19(3.09) | 9.11(8.59) | female |
| skin and subcutaneous tissue disorders | drug reaction with eosinophilia and systemic symptoms | 421 | 61.73(55.94, 68.12) | 60.52(54.87, 66.75) | 23632.5 | 5.86(5.72) | 58.06(53.46) | female |
| skin and subcutaneous tissue disorders | linear iga disease | 192 | 618.2(521.52, 732.81) | 612.58(513.52, 730.76) | 81618.89 | 8.74(8.5) | 426.78(370.18) | female |
| renal and urinary disorders | nephropathy toxic | 153 | 74.68(63.43, 87.91) | 74.14(63.38, 86.73) | 10486.91 | 6.14(5.9) | 70.47(61.48) | female |
| investigations | blood creatinine increased | 160 | 9.24(7.91, 10.8) | 9.18(7.85, 10.74) | 1159.77 | 3.19(2.97) | 9.13(8.01) | female |
| blood and lymphatic system disorders | thrombocytopenia | 139 | 4.75(4.02, 5.62) | 4.73(4.04, 5.53) | 407.97 | 2.24(2) | 4.72(4.1) | female |
| renal and urinary disorders | renal tubular necrosis | 177 | 70.7(60.75, 82.27) | 70.11(59.94, 82.01) | 11486.26 | 6.06(5.84) | 66.82(58.87) | female |
| investigations | drug level increased | 160 | 33.69(28.79, 39.43) | 33.44(28.59, 39.12) | 4919.86 | 5.03(4.8) | 32.69(28.66) | female |
| immune system disorders | anaphylactic reaction | 198 | 10.55(9.17, 12.15) | 10.46(9.12, 12) | 1683.88 | 3.38(3.18) | 10.39(9.24) | female |
| renal and urinary disorders | renal impairment | 130 | 5.92(4.98, 7.04) | 5.89(4.94, 7.03) | 526.56 | 2.55(2.31) | 5.87(5.08) | female |
| blood and lymphatic system disorders | eosinophilia | 134 | 28.74(24.21, 34.12) | 28.56(23.94, 34.07) | 3493.81 | 4.81(4.56) | 28.01(24.27) | female |
| general disorders and administration site conditions | treatment failure | 87 | 3.24(2.62, 4) | 3.23(2.6, 4.01) | 133.66 | 1.69(1.39) | 3.22(2.7) | female |
| blood and lymphatic system disorders | leukopenia | 104 | 6.59(5.43, 7.99) | 6.56(5.39, 7.98) | 488.15 | 2.71(2.43) | 6.53(5.56) | female |
| renal and urinary disorders | tubulointerstitial nephritis | 94 | 20.66(16.85, 25.34) | 20.58(16.92, 25.04) | 1725.89 | 4.34(4.05) | 20.29(17.11) | female |
| infections and infestations | clostridium difficile infection | 132 | 17.94(15.1, 21.31) | 17.83(14.95, 21.27) | 2071.82 | 4.14(3.89) | 17.62(15.26) | female |
| skin and subcutaneous tissue disorders | rash maculo-papular | 95 | 13.82(11.28, 16.92) | 13.76(11.31, 16.74) | 1113.58 | 3.77(3.48) | 13.64(11.51) | female |
| skin and subcutaneous tissue disorders | rash erythematous | 113 | 6.8(5.65, 8.19) | 6.77(5.68, 8.08) | 553.43 | 2.75(2.49) | 6.74(5.77) | female |
| infections and infestations | septic shock | 79 | 6.9(5.53, 8.61) | 6.88(5.55, 8.54) | 395.17 | 2.78(2.46) | 6.85(5.69) | female |
| general disorders and administration site conditions | multiple organ dysfunction syndrome | 86 | 13.43(10.86, 16.62) | 13.38(10.79, 16.6) | 976.12 | 3.73(3.42) | 13.26(11.1) | female |
| skin and subcutaneous tissue disorders | toxic epidermal necrolysis | 97 | 20.85(17.05, 25.49) | 20.76(17.06, 25.26) | 1798.01 | 4.36(4.07) | 20.47(17.3) | female |
| skin and subcutaneous tissue disorders | drug eruption | 61 | 11.14(8.65, 14.33) | 11.11(8.61, 14.33) | 556.84 | 3.46(3.1) | 11.03(8.93) | female |
| skin and subcutaneous tissue disorders | acute generalised exanthematous pustulosis | 105 | 40.33(33.2, 48.98) | 40.13(32.99, 48.82) | 3895.55 | 5.29(5.01) | 39.04(33.18) | female |
| blood and lymphatic system disorders | pancytopenia | 73 | 4.47(3.55, 5.62) | 4.45(3.52, 5.63) | 195.04 | 2.15(1.82) | 4.44(3.66) | female |
| general disorders and administration site conditions | drug resistance | 58 | 9.65(7.45, 12.5) | 9.63(7.46, 12.42) | 445.57 | 3.26(2.89) | 9.57(7.71) | female |
| infections and infestations | staphylococcal infection | 61 | 5.35(4.16, 6.88) | 5.33(4.13, 6.88) | 214.08 | 2.41(2.05) | 5.32(4.31) | female |
| eye disorders | retinal vasculitis | 91 | 280.74(224.09, 351.72) | 279.54(225.33, 346.8) | 21062.63 | 7.87(7.55) | 233.28(193.19) | female |
| hepatobiliary disorders | drug-induced liver injury | 35 | 4.19(3.01, 5.84) | 4.19(3, 5.85) | 84.65 | 2.06(1.59) | 4.18(3.16) | female |
| skin and subcutaneous tissue disorders | stevens-johnson syndrome | 77 | 10.41(8.31, 13.03) | 10.37(8.36, 12.87) | 647.43 | 3.36(3.04) | 10.3(8.54) | female |
| infections and infestations | pathogen resistance | 51 | 31.56(23.9, 41.67) | 31.48(23.93, 41.42) | 1472.42 | 4.95(4.55) | 30.82(24.42) | female |
| skin and subcutaneous tissue disorders | vancomycin infusion reaction | 86 | 2473.95(1741.49, 3514.48) | 2463.87(1731.41, 3506.2) | 76847.12 | 9.81(9.42) | 894.93(667.13) | female |
| investigations | antibiotic level above therapeutic | 59 | 1092.87(777.6, 1535.97) | 1089.82(780.99, 1520.76) | 36131.83 | 9.26(8.82) | 613.96(461.81) | female |
| infections and infestations | enterococcal infection | 49 | 39.51(29.73, 52.5) | 39.42(29.96, 51.87) | 1784.81 | 5.26(4.86) | 38.37(30.25) | female |
| skin and subcutaneous tissue disorders | toxic skin eruption | 69 | 21.16(16.68, 26.85) | 21.09(16.67, 26.68) | 1301.42 | 4.38(4.04) | 20.8(17.04) | female |
| respiratory, thoracic and mediastinal disorders | hypoxia | 40 | 3.82(2.8, 5.21) | 3.81(2.78, 5.21) | 82.79 | 1.93(1.48) | 3.8(2.93) | female |
| general disorders and administration site conditions | face oedema | 49 | 7.27(5.49, 9.62) | 7.25(5.51, 9.54) | 262.81 | 2.85(2.45) | 7.22(5.71) | female |
| blood and lymphatic system disorders | leukocytosis | 44 | 8.25(6.13, 11.1) | 8.24(6.14, 11.06) | 278.25 | 3.03(2.61) | 8.2(6.39) | female |
| vascular disorders | shock | 39 | 5.86(4.28, 8.03) | 5.85(4.28, 8) | 156.35 | 2.54(2.1) | 5.83(4.48) | female |
| skin and subcutaneous tissue disorders | erythema multiforme | 42 | 13.77(10.16, 18.66) | 13.74(10.24, 18.44) | 491.56 | 3.77(3.33) | 13.62(10.56) | female |
| infections and infestations | candida infection | 33 | 5.52(3.92, 7.77) | 5.51(3.95, 7.69) | 121.52 | 2.46(1.97) | 5.5(4.13) | female |
| skin and subcutaneous tissue disorders | rash morbilliform | 29 | 34.14(23.62, 49.36) | 34.1(23.5, 49.49) | 909.59 | 5.06(4.54) | 33.31(24.47) | female |
| investigations | blood urea increased | 28 | 5.64(3.89, 8.18) | 5.64(3.89, 8.18) | 106.42 | 2.49(1.96) | 5.62(4.12) | female |
| infections and infestations | clostridium difficile colitis | 42 | 12.21(9.01, 16.54) | 12.18(9.08, 16.34) | 427.56 | 3.6(3.16) | 12.09(9.37) | female |
| investigations | drug level above therapeutic | 53 | 49.66(37.75, 65.33) | 49.54(37.65, 65.18) | 2434.7 | 5.58(5.19) | 47.88(38.06) | female |
| vascular disorders | haemodynamic instability | 27 | 13.29(9.09, 19.41) | 13.27(9.14, 19.26) | 303.53 | 3.72(3.18) | 13.16(9.58) | female |
| renal and urinary disorders | oliguria | 30 | 15.36(10.72, 22.02) | 15.34(10.78, 21.83) | 397.92 | 3.92(3.41) | 15.19(11.24) | female |
| cardiac disorders | kounis syndrome | 11 | 24.56(13.53, 44.59) | 24.55(13.64, 44.2) | 244.24 | 4.59(3.77) | 24.15(14.66) | female |
| injury, poisoning and procedural complications | incorrect drug administration rate | 32 | 24.37(17.18, 34.57) | 24.34(17.1, 34.64) | 703.88 | 4.58(4.08) | 23.94(17.86) | female |
| blood and lymphatic system disorders | immune thrombocytopenia | 12 | 11.71(6.64, 20.68) | 11.71(6.63, 20.67) | 116.54 | 3.54(2.75) | 11.62(7.22) | female |
| vascular disorders | circulatory collapse | 22 | 3.97(2.61, 6.04) | 3.97(2.63, 5.99) | 48.78 | 1.99(1.4) | 3.96(2.79) | female |
| renal and urinary disorders | anuria | 27 | 10.17(6.96, 14.85) | 10.16(7, 14.74) | 221.38 | 3.34(2.8) | 10.09(7.35) | female |
| infections and infestations | bacteraemia | 27 | 9.07(6.21, 13.24) | 9.05(6.24, 13.13) | 192.25 | 3.17(2.63) | 9(6.56) | female |
| skin and subcutaneous tissue disorders | purpura | 17 | 6.11(3.8, 9.84) | 6.11(3.82, 9.78) | 72.32 | 2.61(1.94) | 6.09(4.08) | female |
| infections and infestations | staphylococcal bacteraemia | 14 | 13.93(8.23, 23.58) | 13.92(8.2, 23.63) | 166.23 | 3.79(3.05) | 13.79(8.88) | female |
| infections and infestations | endocarditis | 20 | 15.48(9.96, 24.06) | 15.47(10.05, 23.81) | 267.74 | 3.94(3.32) | 15.31(10.59) | female |
| immune system disorders | type iv hypersensitivity reaction | 29 | 31.38(21.71, 45.35) | 31.34(21.6, 45.48) | 833.23 | 4.94(4.42) | 30.68(22.54) | female |
| eye disorders | retinal vascular occlusion | 15 | 62.71(37.38, 105.22) | 62.67(37.65, 104.32) | 871.41 | 5.91(5.18) | 60.04(38.94) | female |
| infections and infestations | systemic candida | 16 | 27.38(16.69, 44.91) | 27.36(16.76, 44.66) | 398.56 | 4.75(4.05) | 26.85(17.75) | female |
| ear and labyrinth disorders | ototoxicity | 13 | 27.58(15.93, 47.77) | 27.57(15.93, 47.73) | 326.45 | 4.76(3.99) | 27.06(17.09) | female |
| eye disorders | retinal haemorrhage | 24 | 11.5(7.7, 17.19) | 11.49(7.76, 17) | 228.03 | 3.51(2.94) | 11.41(8.15) | female |
| infections and infestations | klebsiella infection | 15 | 10.84(6.52, 18.03) | 10.84(6.51, 18.04) | 132.93 | 3.43(2.72) | 10.76(7.03) | female |
| skin and subcutaneous tissue disorders | dermatitis exfoliative | 23 | 15.84(10.5, 23.89) | 15.82(10.48, 23.88) | 315.77 | 3.97(3.39) | 15.65(11.1) | female |
| skin and subcutaneous tissue disorders | henoch-schonlein purpura | 13 | 26.43(15.26, 45.75) | 26.41(15.26, 45.72) | 311.97 | 4.7(3.93) | 25.94(16.39) | female |
| vascular disorders | haemorrhagic vasculitis | 26 | 913.62(557.5, 1497.2) | 912.49(559.02, 1489.47) | 14347.18 | 9.11(8.47) | 553.42(366.07) | female |
| investigations | eosinophil count increased | 9 | 3.64(1.89, 7) | 3.64(1.91, 6.95) | 17.16 | 1.86(0.96) | 3.63(2.1) | female |
| skin and subcutaneous tissue disorders | hypersensitivity vasculitis | 17 | 19.42(12.03, 31.34) | 19.4(12.12, 31.05) | 292.69 | 4.26(3.59) | 19.15(12.83) | female |
| investigations | drug level below therapeutic | 10 | 8.83(4.74, 16.44) | 8.82(4.71, 16.51) | 68.94 | 3.13(2.28) | 8.77(5.21) | female |
| skin and subcutaneous tissue disorders | cutaneous vasculitis | 15 | 15.57(9.36, 25.91) | 15.56(9.35, 25.9) | 202.18 | 3.95(3.23) | 15.4(10.06) | female |
| immune system disorders | anaphylactoid reaction | 19 | 13.14(8.36, 20.64) | 13.13(8.37, 20.61) | 210.89 | 3.7(3.07) | 13.01(8.92) | female |
| renal and urinary disorders | renal tubular disorder | 6 | 8.75(3.92, 19.52) | 8.75(3.92, 19.54) | 40.91 | 3.12(2.05) | 8.7(4.44) | female |
| respiratory, thoracic and mediastinal disorders | pulmonary haemorrhage | 11 | 5.64(3.12, 10.19) | 5.64(3.13, 10.15) | 41.78 | 2.49(1.67) | 5.62(3.42) | female |
| general disorders and administration site conditions | multiple-drug resistance | 6 | 7.51(3.37, 16.75) | 7.51(3.36, 16.77) | 33.66 | 2.9(1.83) | 7.47(3.82) | female |
| skin and subcutaneous tissue disorders | skin plaque | 12 | 3.89(2.21, 6.86) | 3.89(2.2, 6.87) | 25.68 | 1.96(1.17) | 3.88(2.42) | female |
| cardiac disorders | pulseless electrical activity | 14 | 11.83(6.99, 20.03) | 11.83(6.97, 20.08) | 137.59 | 3.55(2.82) | 11.73(7.56) | female |
| infections and infestations | pseudomonas infection | 9 | 3.87(2.01, 7.45) | 3.87(2.03, 7.39) | 19.12 | 1.95(1.05) | 3.86(2.23) | female |
| infections and infestations | acinetobacter infection | 15 | 54.73(32.67, 91.7) | 54.69(32.85, 91.04) | 761.05 | 5.72(5) | 52.68(34.21) | female |
| gastrointestinal disorders | noninfectious peritonitis | 17 | 284.34(168.8, 478.95) | 284.11(167.36, 482.3) | 3988.69 | 7.89(7.16) | 236.46(152.85) | female |
| investigations | antibiotic level below therapeutic | 11 | 671.75(327.42, 1378.19) | 671.4(325.11, 1386.54) | 4981.12 | 8.83(7.88) | 454.5(249.11) | female |
| hepatobiliary disorders | hypertransaminasaemia | 9 | 4.95(2.57, 9.53) | 4.95(2.59, 9.45) | 28.28 | 2.3(1.41) | 4.94(2.85) | female |
| general disorders and administration site conditions | systemic inflammatory response syndrome | 11 | 10.05(5.55, 18.19) | 10.05(5.58, 18.09) | 88.98 | 3.32(2.5) | 9.98(6.08) | female |
| gastrointestinal disorders | megacolon | 3 | 7.08(2.28, 22.01) | 7.08(2.27, 22.07) | 15.58 | 2.82(1.4) | 7.05(2.73) | female |
| infections and infestations | stenotrophomonas infection | 11 | 38.24(21.01, 69.62) | 38.22(20.82, 70.17) | 388.18 | 5.22(4.39) | 37.24(22.56) | female |
| renal and urinary disorders | azotaemia | 5 | 4.91(2.04, 11.82) | 4.91(2.03, 11.86) | 15.52 | 2.29(1.14) | 4.9(2.35) | female |
| general disorders and administration site conditions | localised oedema | 11 | 6.37(3.52, 11.52) | 6.37(3.54, 11.47) | 49.53 | 2.66(1.85) | 6.34(3.86) | female |
| infections and infestations | mucormycosis | 9 | 18.7(9.69, 36.1) | 18.69(9.6, 36.39) | 148.72 | 4.21(3.31) | 18.46(10.64) | female |
| gastrointestinal disorders | enterocolitis haemorrhagic | 9 | 17.24(8.93, 33.28) | 17.24(8.85, 33.57) | 135.99 | 4.09(3.19) | 17.04(9.83) | female |
| infections and infestations | septic embolus | 5 | 22.79(9.42, 55.16) | 22.79(9.43, 55.05) | 102.5 | 4.49(3.32) | 22.44(10.71) | female |
| infections and infestations | enterobacter infection | 6 | 14.3(6.4, 31.97) | 14.3(6.4, 31.94) | 73.48 | 3.82(2.75) | 14.17(7.23) | female |
| infections and infestations | intervertebral discitis | 4 | 7.64(2.86, 20.42) | 7.64(2.87, 20.36) | 22.96 | 2.93(1.66) | 7.6(3.34) | female |
| investigations | clostridium test positive | 4 | 6.17(2.31, 16.48) | 6.17(2.32, 16.44) | 17.26 | 2.62(1.35) | 6.15(2.7) | female |
| infections and infestations | meningitis bacterial | 4 | 9.27(3.47, 24.78) | 9.27(3.48, 24.7) | 29.3 | 3.2(1.93) | 9.21(4.05) | female |
| skin and subcutaneous tissue disorders | urticaria | 228 | 3.39(2.97, 3.86) | 3.36(2.93, 3.85) | 378.67 | 1.75(1.56) | 3.36(3.01) | female |
| skin and subcutaneous tissue disorders | rash | 482 | 3.08(2.81, 3.37) | 3.03(2.75, 3.34) | 658.44 | 1.6(1.47) | 3.02(2.8) | female |
| blood and lymphatic system disorders | neutropenia | 117 | 3.01(2.51, 3.61) | 3(2.51, 3.58) | 155.58 | 1.58(1.32) | 2.99(2.57) | female |
| blood and lymphatic system disorders | disseminated intravascular coagulation | 38 | 9.19(6.68, 12.65) | 9.18(6.71, 12.56) | 275.18 | 3.19(2.74) | 9.13(6.99) | female |
| metabolism and nutrition disorders | hyperkalaemia | 35 | 4.14(2.97, 5.77) | 4.14(2.97, 5.78) | 82.98 | 2.04(1.57) | 4.13(3.13) | female |
| renal and urinary disorders | renal failure | 152 | 4.13(3.52, 4.85) | 4.11(3.51, 4.81) | 357.34 | 2.04(1.81) | 4.1(3.59) | female |
| skin and subcutaneous tissue disorders | erythema | 297 | 3.58(3.19, 4.01) | 3.54(3.15, 3.98) | 542.03 | 1.82(1.66) | 3.53(3.21) | female |
| skin and subcutaneous tissue disorders | pruritus | 454 | 3.26(2.97, 3.57) | 3.21(2.91, 3.54) | 693.16 | 1.68(1.55) | 3.2(2.96) | female |
| respiratory, thoracic and mediastinal disorders | apnoea | 9 | 3.69(1.92, 7.11) | 3.69(1.93, 7.05) | 17.62 | 1.88(0.99) | 3.69(2.13) | female |
| respiratory, thoracic and mediastinal disorders | respiratory distress | 26 | 3.11(2.11, 4.56) | 3.1(2.09, 4.59) | 37 | 1.63(1.09) | 3.1(2.25) | female |
| skin and subcutaneous tissue disorders | rash pruritic | 92 | 4.41(3.59, 5.42) | 4.4(3.62, 5.35) | 240.85 | 2.13(1.84) | 4.39(3.69) | female |
| infections and infestations | cardiac valve vegetation | 5 | 46.5(19.07, 113.34) | 46.48(19.24, 112.28) | 215.4 | 5.49(4.32) | 45.03(21.36) | female |
| blood and lymphatic system disorders | agranulocytosis | 47 | 8.61(6.46, 11.48) | 8.59(6.4, 11.53) | 313.57 | 3.1(2.69) | 8.55(6.72) | female |
| skin and subcutaneous tissue disorders | blister | 80 | 3.63(2.92, 4.53) | 3.62(2.92, 4.49) | 151.7 | 1.85(1.54) | 3.62(3.01) | female |
| eye disorders | conjunctival oedema | 4 | 11.1(4.15, 29.69) | 11.1(4.17, 29.58) | 36.46 | 3.46(2.19) | 11.02(4.84) | female |
| immune system disorders | anaphylactic shock | 61 | 6.78(5.27, 8.72) | 6.76(5.24, 8.72) | 298.04 | 2.75(2.39) | 6.73(5.45) | female |
| cardiac disorders | cardiac arrest | 93 | 3.94(3.21, 4.83) | 3.93(3.23, 4.78) | 202.58 | 1.97(1.68) | 3.92(3.3) | female |
| injury, poisoning and procedural complications | infusion related reaction | 117 | 5.1(4.25, 6.11) | 5.07(4.25, 6.05) | 381.71 | 2.34(2.08) | 5.06(4.34) | female |
| investigations | lymphocyte stimulation test positive | 3 | 11.48(3.68, 35.75) | 11.48(3.68, 35.78) | 28.46 | 3.51(2.09) | 11.39(4.4) | female |
| hepatobiliary disorders | cholestasis | 52 | 8.95(6.81, 11.76) | 8.93(6.79, 11.75) | 364.11 | 3.15(2.76) | 8.88(7.07) | female |
| skin and subcutaneous tissue disorders | petechiae | 19 | 5.29(3.37, 8.3) | 5.28(3.36, 8.29) | 65.75 | 2.4(1.76) | 5.27(3.61) | female |
| investigations | prothrombin time prolonged | 9 | 4.54(2.36, 8.74) | 4.54(2.38, 8.67) | 24.76 | 2.18(1.28) | 4.53(2.62) | female |
| infections and infestations | clostridial infection | 7 | 5.59(2.66, 11.74) | 5.59(2.65, 11.77) | 26.26 | 2.48(1.48) | 5.57(2.99) | female |
| respiratory, thoracic and mediastinal disorders | acute respiratory distress syndrome | 17 | 3.47(2.15, 5.58) | 3.47(2.17, 5.55) | 29.76 | 1.79(1.12) | 3.46(2.32) | female |
| nervous system disorders | encephalopathy | 41 | 5.88(4.33, 8) | 5.87(4.29, 8.03) | 165.1 | 2.55(2.11) | 5.85(4.53) | female |
| nervous system disorders | metabolic encephalopathy | 6 | 6.04(2.71, 13.47) | 6.04(2.7, 13.49) | 25.11 | 2.59(1.52) | 6.02(3.08) | female |
| investigations | gamma-glutamyltransferase increased | 23 | 3.09(2.06, 4.66) | 3.09(2.05, 4.66) | 32.5 | 1.63(1.05) | 3.09(2.19) | female |
| respiratory, thoracic and mediastinal disorders | laryngeal oedema | 9 | 3.98(2.07, 7.65) | 3.98(2.08, 7.6) | 19.99 | 1.99(1.09) | 3.97(2.29) | female |
| immune system disorders | type i hypersensitivity | 10 | 9.28(4.98, 17.28) | 9.27(4.95, 17.36) | 73.32 | 3.2(2.35) | 9.22(5.48) | female |
| renal and urinary disorders | renal injury | 25 | 3.27(2.21, 4.84) | 3.27(2.21, 4.84) | 39.23 | 1.71(1.15) | 3.26(2.35) | female |
| gastrointestinal disorders | lip swelling | 47 | 3.51(2.63, 4.67) | 3.5(2.61, 4.7) | 83.88 | 1.81(1.4) | 3.5(2.75) | female |
| general disorders and administration site conditions | infusion site oedema | 4 | 11.63(4.35, 31.11) | 11.63(4.36, 30.99) | 38.53 | 3.53(2.26) | 11.54(5.06) | female |
| skin and subcutaneous tissue disorders | dermatitis bullous | 20 | 8.68(5.59, 13.48) | 8.67(5.63, 13.34) | 134.96 | 3.11(2.49) | 8.63(5.97) | female |
| investigations | anti-platelet antibody positive | 3 | 65.81(20.68, 209.5) | 65.8(20.7, 209.14) | 182.89 | 5.98(4.52) | 62.9(23.87) | female |
| renal and urinary disorders | nephrocalcinosis | 3 | 7.6(2.44, 23.65) | 7.6(2.44, 23.69) | 17.11 | 2.92(1.5) | 7.57(2.93) | female |
| skin and subcutaneous tissue disorders | angioedema | 54 | 4.12(3.16, 5.39) | 4.12(3.13, 5.42) | 127.05 | 2.04(1.66) | 4.11(3.28) | female |
| nervous system disorders | unresponsive to stimuli | 30 | 3.69(2.58, 5.29) | 3.69(2.59, 5.25) | 58.67 | 1.88(1.37) | 3.68(2.73) | female |
| general disorders and administration site conditions | infusion site rash | 5 | 9.26(3.84, 22.32) | 9.26(3.83, 22.37) | 36.6 | 3.2(2.04) | 9.21(4.41) | female |
| investigations | eosinophils urine present | 3 | 234.01(68.92, 794.48) | 233.97(69.41, 788.71) | 596.51 | 7.65(6.11) | 200.69(72.17) | female |
| metabolism and nutrition disorders | metabolic acidosis | 37 | 3.8(2.75, 5.24) | 3.79(2.77, 5.19) | 75.85 | 1.92(1.46) | 3.78(2.89) | female |
| investigations | pulse absent | 9 | 5.95(3.09, 11.46) | 5.95(3.12, 11.36) | 36.92 | 2.57(1.67) | 5.93(3.43) | female |
| general disorders and administration site conditions | necrosis | 7 | 3.88(1.85, 8.15) | 3.88(1.84, 8.17) | 14.93 | 1.95(0.95) | 3.87(2.08) | female |
| skin and subcutaneous tissue disorders | exfoliative rash | 7 | 8.02(3.81, 16.86) | 8.02(3.81, 16.89) | 42.74 | 3(1.99) | 7.98(4.28) | female |
| investigations | transaminases increased | 44 | 6.41(4.76, 8.62) | 6.39(4.76, 8.57) | 199.41 | 2.67(2.25) | 6.37(4.97) | female |
| injury, poisoning and procedural complications | procedural hypotension | 4 | 12.97(4.85, 34.72) | 12.97(4.87, 34.56) | 43.78 | 3.68(2.41) | 12.86(5.64) | female |
| investigations | blood creatine increased | 9 | 7.37(3.83, 14.18) | 7.36(3.85, 14.05) | 49.23 | 2.87(1.98) | 7.33(4.24) | female |
| general disorders and administration site conditions | infusion site extravasation | 10 | 5.14(2.76, 9.57) | 5.14(2.75, 9.62) | 33.21 | 2.36(1.5) | 5.12(3.05) | female |
| injury, poisoning and procedural complications | toxic anterior segment syndrome | 7 | 23.02(10.91, 48.59) | 23.01(10.93, 48.46) | 145.02 | 4.5(3.49) | 22.66(12.13) | female |
| respiratory, thoracic and mediastinal disorders | bronchospasm | 30 | 5.7(3.98, 8.16) | 5.69(4, 8.1) | 115.66 | 2.5(2) | 5.68(4.2) | female |
| blood and lymphatic system disorders | haemolysis | 12 | 5.94(3.37, 10.48) | 5.94(3.36, 10.49) | 49.1 | 2.57(1.78) | 5.92(3.68) | female |
| hepatobiliary disorders | hyperbilirubinaemia | 10 | 4.29(2.31, 7.98) | 4.29(2.29, 8.03) | 25.14 | 2.1(1.24) | 4.28(2.54) | female |
| general disorders and administration site conditions | catheter site pruritus | 3 | 6.59(2.12, 20.49) | 6.59(2.11, 20.54) | 14.16 | 2.71(1.3) | 6.56(2.54) | female |
| infections and infestations | cytomegalovirus colitis | 5 | 9.99(4.14, 24.07) | 9.98(4.13, 24.11) | 40.14 | 3.31(2.15) | 9.92(4.75) | female |
| infections and infestations | pneumonia klebsiella | 6 | 13.5(6.04, 30.17) | 13.5(6.04, 30.15) | 68.77 | 3.74(2.67) | 13.38(6.83) | female |
| infections and infestations | peritonitis bacterial | 11 | 3.99(2.21, 7.22) | 3.99(2.22, 7.18) | 24.59 | 1.99(1.18) | 3.98(2.43) | female |
| psychiatric disorders | mental status changes | 30 | 3.59(2.51, 5.14) | 3.59(2.52, 5.11) | 55.83 | 1.84(1.33) | 3.58(2.65) | female |
| infections and infestations | abdominal sepsis | 3 | 11.51(3.69, 35.85) | 11.51(3.69, 35.87) | 28.55 | 3.51(2.09) | 11.42(4.41) | female |
| infections and infestations | brain abscess | 4 | 6.34(2.37, 16.93) | 6.34(2.38, 16.89) | 17.9 | 2.66(1.39) | 6.31(2.78) | female |
| renal and urinary disorders | nephropathy | 19 | 7.01(4.46, 11) | 7(4.46, 10.99) | 97.26 | 2.8(2.17) | 6.97(4.78) | female |
| infections and infestations | staphylococcal sepsis | 7 | 5.02(2.39, 10.55) | 5.02(2.38, 10.57) | 22.45 | 2.32(1.32) | 5(2.69) | female |
| infections and infestations | lung abscess | 7 | 13.79(6.55, 29.03) | 13.78(6.54, 29.02) | 82.18 | 3.77(2.77) | 13.66(7.33) | female |
| gastrointestinal disorders | oedema mouth | 6 | 3.96(1.78, 8.83) | 3.96(1.77, 8.84) | 13.24 | 1.98(0.91) | 3.95(2.02) | female |
| gastrointestinal disorders | gastric perforation | 4 | 5.19(1.94, 13.86) | 5.19(1.95, 13.83) | 13.48 | 2.37(1.1) | 5.17(2.28) | female |
| general disorders and administration site conditions | extravasation | 10 | 8.77(4.71, 16.33) | 8.76(4.68, 16.4) | 68.35 | 3.12(2.27) | 8.71(5.18) | female |
| general disorders and administration site conditions | infusion site necrosis | 6 | 110.86(48.28, 254.56) | 110.83(48.66, 252.45) | 605.25 | 6.68(5.57) | 102.79(51.27) | female |
| general disorders and administration site conditions | hyperthermia | 17 | 7.76(4.81, 12.5) | 7.75(4.84, 12.4) | 99.42 | 2.95(2.28) | 7.71(5.18) | female |
| investigations | urine output decreased | 15 | 4.96(2.99, 8.24) | 4.96(2.98, 8.26) | 47.27 | 2.31(1.6) | 4.95(3.24) | female |
| hepatobiliary disorders | hepatitis | 32 | 3.72(2.63, 5.26) | 3.71(2.61, 5.28) | 63.32 | 1.89(1.4) | 3.71(2.77) | female |
| infections and infestations | urinary tract infection enterococcal | 3 | 7.43(2.39, 23.1) | 7.43(2.38, 23.16) | 16.6 | 2.89(1.47) | 7.39(2.86) | female |
| injury, poisoning and procedural complications | maternal exposure during delivery | 4 | 8.94(3.35, 23.91) | 8.94(3.36, 23.82) | 28.04 | 3.15(1.88) | 8.89(3.91) | female |
| cardiac disorders | myocarditis | 15 | 6.08(3.66, 10.09) | 6.07(3.65, 10.1) | 63.31 | 2.6(1.89) | 6.05(3.96) | female |
| infections and infestations | nosocomial infection | 5 | 6.63(2.75, 15.96) | 6.63(2.74, 16.02) | 23.78 | 2.72(1.57) | 6.6(3.16) | female |
| hepatobiliary disorders | hepatocellular injury | 24 | 4.98(3.34, 7.44) | 4.98(3.37, 7.37) | 76.09 | 2.31(1.75) | 4.97(3.55) | female |
| general disorders and administration site conditions | potentiating drug interaction | 5 | 5.49(2.28, 13.22) | 5.49(2.27, 13.26) | 18.3 | 2.45(1.3) | 5.47(2.63) | female |
| hepatobiliary disorders | jaundice cholestatic | 5 | 5.22(2.17, 12.55) | 5.21(2.16, 12.59) | 16.97 | 2.38(1.22) | 5.2(2.49) | female |
| skin and subcutaneous tissue disorders | rash vesicular | 7 | 3.99(1.9, 8.38) | 3.99(1.89, 8.4) | 15.63 | 1.99(0.99) | 3.98(2.14) | female |
| nervous system disorders | toxic encephalopathy | 6 | 4.4(1.97, 9.81) | 4.4(1.97, 9.83) | 15.72 | 2.13(1.06) | 4.39(2.24) | female |
| hepatobiliary disorders | liver injury | 24 | 3.37(2.26, 5.03) | 3.37(2.28, 4.99) | 39.91 | 1.75(1.18) | 3.36(2.41) | female |
| skin and subcutaneous tissue disorders | nikolsky's sign | 6 | 30.75(13.69, 69.05) | 30.74(13.76, 68.66) | 168.94 | 4.91(3.83) | 30.1(15.3) | female |
| investigations | creatinine renal clearance decreased | 8 | 5.32(2.66, 10.66) | 5.32(2.68, 10.56) | 27.98 | 2.41(1.46) | 5.31(2.97) | female |
| skin and subcutaneous tissue disorders | dermatitis allergic | 18 | 3.93(2.47, 6.24) | 3.92(2.45, 6.27) | 39.11 | 1.97(1.32) | 3.92(2.66) | female |
| infections and infestations | pseudomembranous colitis | 14 | 19.82(11.7, 33.6) | 19.81(11.67, 33.63) | 246.6 | 4.29(3.55) | 19.55(12.57) | female |
| blood and lymphatic system disorders | pseudolymphoma | 5 | 28.66(11.82, 69.47) | 28.65(11.86, 69.21) | 130.76 | 4.81(3.64) | 28.1(13.39) | female |
| blood and lymphatic system disorders | lymphocytic infiltration | 3 | 9.51(3.05, 29.6) | 9.51(3.05, 29.64) | 22.68 | 3.24(1.82) | 9.45(3.65) | female |
| infections and infestations | rash pustular | 10 | 3.71(1.99, 6.9) | 3.71(1.98, 6.95) | 19.73 | 1.89(1.03) | 3.7(2.2) | female |
| general disorders and administration site conditions | hyperpyrexia | 7 | 6.01(2.86, 12.63) | 6.01(2.85, 12.66) | 29.11 | 2.58(1.58) | 5.99(3.22) | female |
| cardiac disorders | tachyarrhythmia | 8 | 9.98(4.98, 20) | 9.97(5.02, 19.8) | 64.14 | 3.31(2.36) | 9.91(5.54) | female |
| investigations | pancreatic enzymes increased | 3 | 6.61(2.13, 20.56) | 6.61(2.12, 20.6) | 14.22 | 2.72(1.3) | 6.59(2.55) | female |
| investigations | enterococcus test positive | 4 | 21.27(7.92, 57.11) | 21.27(7.98, 56.67) | 76.12 | 4.39(3.11) | 20.97(9.18) | female |
| respiratory, thoracic and mediastinal disorders | eosinophilic pneumonia | 8 | 11.05(5.51, 22.15) | 11.04(5.56, 21.92) | 72.5 | 3.45(2.51) | 10.96(6.13) | female |
| blood and lymphatic system disorders | allergic eosinophilia | 3 | 382.92(106.82, 1372.67) | 382.86(107.09, 1368.77) | 897.76 | 8.23(6.64) | 301.04(103.44) | female |
| hepatobiliary disorders | hepatitis fulminant | 8 | 9.59(4.78, 19.22) | 9.58(4.82, 19.02) | 61.08 | 3.25(2.31) | 9.52(5.32) | female |
| blood and lymphatic system disorders | haemolytic anaemia | 25 | 9.41(6.35, 13.95) | 9.4(6.35, 13.91) | 186.41 | 3.22(2.67) | 9.34(6.72) | female |
| general disorders and administration site conditions | catheter site related reaction | 3 | 11.93(3.83, 37.18) | 11.93(3.83, 37.18) | 29.79 | 3.57(2.14) | 11.84(4.57) | female |
| investigations | drug clearance decreased | 6 | 18.52(8.27, 41.44) | 18.51(8.29, 41.34) | 98.1 | 4.19(3.12) | 18.28(9.32) | female |
| general disorders and administration site conditions | foaming at mouth | 8 | 11.8(5.88, 23.67) | 11.8(5.94, 23.43) | 78.4 | 3.55(2.6) | 11.71(6.54) | female |
| nervous system disorders | generalised tonic-clonic seizure | 16 | 3.23(1.98, 5.28) | 3.23(1.98, 5.27) | 24.58 | 1.69(1) | 3.22(2.14) | female |
| nervous system disorders | tongue biting | 4 | 5.5(2.06, 14.69) | 5.5(2.06, 14.65) | 14.67 | 2.45(1.19) | 5.48(2.41) | female |
| renal and urinary disorders | renal failure neonatal | 3 | 56.16(17.71, 178.1) | 56.15(17.67, 178.47) | 156.26 | 5.76(4.31) | 54.03(20.57) | female |
| investigations | venous pressure jugular increased | 3 | 28.27(9.01, 88.66) | 28.27(9.07, 88.11) | 77.34 | 4.79(3.36) | 27.73(10.65) | female |
| gastrointestinal disorders | lip oedema | 9 | 5.93(3.08, 11.41) | 5.92(3.1, 11.3) | 36.68 | 2.56(1.66) | 5.9(3.41) | female |
| metabolism and nutrition disorders | hypernatraemia | 7 | 5.12(2.44, 10.77) | 5.12(2.43, 10.78) | 23.15 | 2.35(1.35) | 5.11(2.75) | female |
| nervous system disorders | change in seizure presentation | 3 | 14.42(4.62, 44.99) | 14.42(4.63, 44.94) | 37.1 | 3.84(2.41) | 14.29(5.52) | female |
| metabolism and nutrition disorders | hyperamylasaemia | 3 | 20.25(6.48, 63.31) | 20.25(6.5, 63.11) | 54.11 | 4.32(2.89) | 19.97(7.7) | female |
| metabolism and nutrition disorders | hyperlipasaemia | 3 | 22.29(7.12, 69.73) | 22.28(7.15, 69.44) | 60.03 | 4.46(3.03) | 21.95(8.45) | female |
| general disorders and administration site conditions | administration site extravasation | 6 | 22.96(10.25, 51.44) | 22.95(10.28, 51.26) | 123.94 | 4.5(3.42) | 22.6(11.5) | female |
| eye disorders | vitreous haemorrhage | 5 | 7.46(3.1, 17.97) | 7.46(3.09, 18.02) | 27.82 | 2.89(1.73) | 7.43(3.56) | female |
| endocrine disorders | diabetes insipidus | 4 | 5.72(2.14, 15.27) | 5.72(2.15, 15.24) | 15.51 | 2.51(1.24) | 5.7(2.51) | female |
| immune system disorders | graft versus host disease | 6 | 4.3(1.93, 9.58) | 4.3(1.93, 9.6) | 15.12 | 2.1(1.03) | 4.29(2.19) | female |
| immune system disorders | graft versus host disease in gastrointestinal tract | 4 | 8.72(3.26, 23.31) | 8.72(3.27, 23.23) | 27.17 | 3.12(1.85) | 8.67(3.81) | female |
| immune system disorders | graft versus host disease in skin | 3 | 6.1(1.96, 18.98) | 6.1(1.96, 19.01) | 12.75 | 2.6(1.19) | 6.08(2.35) | female |
| infections and infestations | leuconostoc infection | 5 | 1170.14(357.08, 3834.49) | 1169.86(353.91, 3867.02) | 3185.08 | 9.32(7.9) | 638.56(236.53) | female |
| investigations | laboratory test interference | 4 | 16.87(6.29, 45.21) | 16.86(6.33, 44.92) | 58.98 | 4.06(2.79) | 16.67(7.31) | female |
| injury, poisoning and procedural complications | drug monitoring procedure incorrectly performed | 7 | 36.01(17, 76.26) | 36(17.09, 75.82) | 232.21 | 5.13(4.12) | 35.12(18.75) | female |
| congenital, familial and genetic disorders | left ventricle outflow tract obstruction | 3 | 25.84(8.25, 80.96) | 25.84(8.29, 80.54) | 70.33 | 4.67(3.24) | 25.39(9.76) | female |
| gastrointestinal disorders | necrotising enterocolitis neonatal | 5 | 73.9(30.06, 181.67) | 73.89(29.99, 182.03) | 341.52 | 6.13(4.95) | 70.24(33.09) | female |
| metabolism and nutrition disorders | acidosis | 11 | 4.82(2.66, 8.71) | 4.81(2.67, 8.66) | 33.13 | 2.26(1.45) | 4.8(2.92) | female |
| blood and lymphatic system disorders | neutrophilia | 8 | 5.27(2.63, 10.55) | 5.27(2.65, 10.46) | 27.56 | 2.39(1.45) | 5.25(2.94) | female |
| nervous system disorders | posterior reversible encephalopathy syndrome | 16 | 4.82(2.95, 7.88) | 4.82(2.95, 7.87) | 48.25 | 2.26(1.58) | 4.8(3.19) | female |
| neoplasms benign, malignant and unspecified (incl cysts and polyps) | neuroendocrine tumour of the lung metastatic | 4 | 351.02(117.34, 1050.07) | 350.96(117.11, 1051.81) | 1116.68 | 8.13(6.73) | 280.97(112.32) | female |
| eye disorders | optic atrophy | 7 | 13.48(6.4, 28.39) | 13.48(6.4, 28.39) | 80.11 | 3.74(2.73) | 13.36(7.17) | female |
| general disorders and administration site conditions | administration site rash | 3 | 55.42(17.48, 175.7) | 55.41(17.43, 176.12) | 154.21 | 5.74(4.29) | 53.35(20.32) | female |
| injury, poisoning and procedural complications | seroma | 6 | 11.98(5.37, 26.77) | 11.98(5.36, 26.76) | 59.88 | 3.57(2.5) | 11.89(6.07) | female |
| respiratory, thoracic and mediastinal disorders | infantile apnoea | 3 | 17.7(5.67, 55.27) | 17.7(5.68, 55.17) | 46.67 | 4.13(2.7) | 17.49(6.74) | female |
| injury, poisoning and procedural complications | dose calculation error | 5 | 31.34(12.92, 76.05) | 31.34(12.97, 75.71) | 143.63 | 4.94(3.77) | 30.67(14.61) | female |
| congenital, familial and genetic disorders | macroglossia | 4 | 22.56(8.4, 60.58) | 22.55(8.46, 60.08) | 81.08 | 4.47(3.2) | 22.21(9.72) | female |
| infections and infestations | enterococcal bacteraemia | 3 | 12.65(4.06, 39.42) | 12.65(4.06, 39.43) | 31.89 | 3.65(2.23) | 12.54(4.85) | female |
| renal and urinary disorders | myeloma cast nephropathy | 8 | 295.66(137.92, 633.8) | 295.54(137.61, 634.73) | 1939.97 | 7.93(6.9) | 244.32(129.08) | female |
| infections and infestations | fungal sepsis | 3 | 12.39(3.98, 38.61) | 12.39(3.98, 38.62) | 31.13 | 3.62(2.2) | 12.29(4.75) | female |
| general disorders and administration site conditions | drug ineffective for unapproved indication | 96 | 5.44(4.45, 6.65) | 5.42(4.46, 6.59) | 345.21 | 2.43(2.15) | 5.41(4.57) | female |
| eye disorders | retinal ischaemia | 10 | 56.63(30.09, 106.58) | 56.61(30.23, 105.99) | 525.07 | 5.77(4.9) | 54.45(32.08) | female |
| skin and subcutaneous tissue disorders | skin necrosis | 10 | 5.38(2.89, 10.02) | 5.38(2.87, 10.07) | 35.55 | 2.42(1.57) | 5.37(3.19) | female |
| blood and lymphatic system disorders | thrombotic thrombocytopenic purpura | 11 | 9.97(5.51, 18.04) | 9.96(5.53, 17.93) | 88.07 | 3.31(2.49) | 9.9(6.03) | female |
| vascular disorders | hyperaemia | 14 | 20.05(11.83, 33.98) | 20.03(11.8, 34) | 249.63 | 4.3(3.57) | 19.77(12.71) | female |
| eye disorders | retinal vascular disorder | 3 | 17.85(5.71, 55.74) | 17.85(5.73, 55.63) | 47.11 | 4.14(2.71) | 17.63(6.8) | female |
| infections and infestations | cerebral aspergillosis | 3 | 25.68(8.2, 80.47) | 25.68(8.24, 80.04) | 69.88 | 4.66(3.23) | 25.24(9.71) | female |
| skin and subcutaneous tissue disorders | dermatitis exfoliative generalised | 14 | 17.85(10.53, 30.24) | 17.83(10.5, 30.27) | 219.69 | 4.14(3.4) | 17.62(11.34) | female |
| gastrointestinal disorders | lip erythema | 4 | 12.94(4.83, 34.64) | 12.94(4.86, 34.48) | 43.66 | 3.68(2.41) | 12.83(5.63) | female |
| hepatobiliary disorders | mixed liver injury | 7 | 8.56(4.07, 17.99) | 8.55(4.06, 18.01) | 46.41 | 3.09(2.09) | 8.51(4.57) | female |
| nervous system disorders | tonic clonic movements | 6 | 14.81(6.62, 33.1) | 14.8(6.63, 33.06) | 76.42 | 3.87(2.8) | 14.66(7.48) | female |
| infections and infestations | candida endophthalmitis | 4 | 208.01(72.78, 594.54) | 207.98(72.17, 599.35) | 717.61 | 7.5(6.14) | 181.27(75.28) | female |
| renal and urinary disorders | neonatal anuria | 3 | 120.35(37.01, 391.33) | 120.33(37.12, 390.04) | 326.98 | 6.79(5.31) | 110.91(41.35) | female |
| nervous system disorders | myasthenia gravis crisis | 10 | 35.38(18.88, 66.27) | 35.36(18.89, 66.21) | 325.69 | 5.11(4.25) | 34.52(20.41) | female |
| neoplasms benign, malignant and unspecified (incl cysts and polyps) | langerhans' cell histiocytosis | 6 | 58.11(25.68, 131.49) | 58.09(25.5, 132.32) | 323.27 | 5.8(4.71) | 55.82(28.19) | female |
| respiratory, thoracic and mediastinal disorders | pulmonary necrosis | 3 | 35.1(11.16, 110.38) | 35.1(11.26, 109.4) | 96.95 | 5.1(3.66) | 34.26(13.14) | female |
| skin and subcutaneous tissue disorders | symmetrical drug-related intertriginous and flexural exanthema | 23 | 115.03(75.18, 176) | 114.9(74.65, 176.84) | 2400.54 | 6.73(6.13) | 106.29(74.46) | female |
| injury, poisoning and procedural complications | therapeutic drug monitoring analysis not performed | 4 | 32.84(12.19, 88.52) | 32.84(12.09, 89.23) | 120.65 | 5(3.72) | 32.11(14.01) | female |
| infections and infestations | empyema | 5 | 11.57(4.8, 27.89) | 11.56(4.79, 27.93) | 47.86 | 3.52(2.36) | 11.48(5.5) | female |
| general disorders and administration site conditions | administration site erythema | 3 | 25.84(8.25, 80.96) | 25.84(8.29, 80.54) | 70.33 | 4.67(3.24) | 25.39(9.76) | female |
| general disorders and administration site conditions | administration site pain | 4 | 9.94(3.72, 26.58) | 9.94(3.73, 26.48) | 31.93 | 3.3(2.03) | 9.88(4.34) | female |
| ear and labyrinth disorders | deafness bilateral | 8 | 14.92(7.43, 29.95) | 14.91(7.36, 30.19) | 102.76 | 3.88(2.94) | 14.77(8.24) | female |
| infections and infestations | amniotic cavity infection | 3 | 8.08(2.6, 25.15) | 8.08(2.59, 25.18) | 18.52 | 3.01(1.59) | 8.04(3.11) | female |
| neoplasms benign, malignant and unspecified (incl cysts and polyps) | differentiation syndrome | 3 | 20.35(6.51, 63.61) | 20.35(6.53, 63.43) | 54.4 | 4.33(2.9) | 20.07(7.73) | female |
| infections and infestations | systemic infection | 7 | 14.5(6.88, 30.53) | 14.49(6.88, 30.52) | 87.04 | 3.84(2.84) | 14.36(7.7) | female |
| investigations | haematocrit abnormal | 7 | 22.75(10.78, 48.02) | 22.75(10.8, 47.91) | 143.22 | 4.49(3.48) | 22.4(11.99) | female |
| investigations | haemoglobin abnormal | 7 | 3.98(1.9, 8.37) | 3.98(1.89, 8.38) | 15.6 | 1.99(0.99) | 3.97(2.14) | female |
| investigations | prothrombin time abnormal | 4 | 19.37(7.22, 51.96) | 19.36(7.27, 51.58) | 68.71 | 4.26(2.98) | 19.11(8.37) | female |
| investigations | red blood cell count abnormal | 4 | 8.72(3.26, 23.31) | 8.72(3.27, 23.23) | 27.17 | 3.12(1.85) | 8.67(3.81) | female |
| investigations | red cell distribution width abnormal | 7 | 112.99(52.31, 244.07) | 112.95(52.59, 242.58) | 718.89 | 6.71(5.67) | 104.62(54.92) | female |
| blood and lymphatic system disorders | bicytopenia | 4 | 7.27(2.72, 19.41) | 7.26(2.72, 19.34) | 21.5 | 2.85(1.59) | 7.23(3.18) | female |
| investigations | anti factor xa activity decreased | 4 | 936.07(264.13, 3317.38) | 935.89(261.78, 3345.92) | 2241.33 | 9.13(7.61) | 561.93(194.94) | female |
| general disorders and administration site conditions | drug chemical incompatibility | 4 | 200.59(70.35, 571.92) | 200.55(70.97, 566.72) | 694.93 | 7.46(6.1) | 175.6(73.08) | female |
| infections and infestations | subperiosteal abscess | 4 | 510.58(162.56, 1603.63) | 510.48(163.78, 1591.05) | 1491.56 | 8.55(7.1) | 374.62(143.78) | female |
| respiratory, thoracic and mediastinal disorders | laryngeal discomfort | 3 | 12.17(3.91, 37.93) | 12.17(3.9, 37.93) | 30.5 | 3.59(2.17) | 12.08(4.67) | female |
| injury, poisoning and procedural complications | product administration error | 19 | 3.14(2, 4.93) | 3.14(2, 4.93) | 27.65 | 1.65(1.01) | 3.13(2.15) | female |
| blood and lymphatic system disorders | anisocytosis | 7 | 31.81(15.04, 67.3) | 31.8(15.1, 66.97) | 204.21 | 4.96(3.95) | 31.12(16.62) | female |
| injury, poisoning and procedural complications | central nervous system injury | 7 | 468.1(198.97, 1101.25) | 467.94(197.54, 1108.47) | 2446.22 | 8.46(7.33) | 351.21(171.66) | female |
| metabolism and nutrition disorders | hyperchloraemia | 7 | 33.66(15.91, 71.25) | 33.65(15.98, 70.87) | 216.59 | 5.04(4.03) | 32.89(17.56) | female |
| blood and lymphatic system disorders | hypochromasia | 7 | 108.02(50.07, 233.05) | 107.99(50.28, 231.93) | 688.98 | 6.65(5.61) | 100.35(52.73) | female |
| blood and lymphatic system disorders | macrocytosis | 7 | 15.68(7.44, 33.03) | 15.67(7.44, 33) | 95.1 | 3.96(2.95) | 15.51(8.32) | female |
| injury, poisoning and procedural complications | splenic injury | 7 | 89.36(41.62, 191.88) | 89.33(41.59, 191.85) | 574.84 | 6.39(5.36) | 84.05(44.35) | female |
| hepatobiliary disorders | venoocclusive liver disease | 8 | 8.87(4.42, 17.77) | 8.86(4.46, 17.59) | 55.47 | 3.14(2.19) | 8.81(4.93) | female |
| infections and infestations | hypopyon | 3 | 11.73(3.77, 36.55) | 11.73(3.76, 36.56) | 29.21 | 3.54(2.12) | 11.64(4.5) | female |
| general disorders and administration site conditions | vessel puncture site inflammation | 4 | 624.04(192.16, 2026.59) | 623.93(192.49, 2022.4) | 1722.26 | 8.76(7.28) | 432.26(161.33) | female |
| infections and infestations | rotavirus infection | 3 | 18.56(5.94, 57.97) | 18.55(5.95, 57.82) | 49.17 | 4.2(2.77) | 18.32(7.06) | female |
| immune system disorders | anaphylactoid shock | 3 | 20.15(6.45, 63) | 20.15(6.47, 62.8) | 53.83 | 4.31(2.89) | 19.88(7.66) | female |
| investigations | bacterial test | 5 | 34.42(14.17, 83.58) | 34.41(14.24, 83.13) | 158.3 | 5.07(3.9) | 33.61(16) | female |
| injury, poisoning and procedural complications | therapeutic drug monitoring analysis incorrectly performed | 5 | 280.83(107.5, 733.67) | 280.77(107.46, 733.58) | 1161.54 | 7.87(6.61) | 234.14(104.84) | female |
| infections and infestations | liver abscess | 5 | 6.61(2.75, 15.92) | 6.61(2.74, 15.97) | 23.69 | 2.72(1.56) | 6.58(3.16) | female |
| renal and urinary disorders | crystalluria | 4 | 26.74(9.94, 71.93) | 26.74(10.04, 71.25) | 97.26 | 4.71(3.44) | 26.26(11.47) | female |
| nervous system disorders | areflexia | 5 | 5.48(2.28, 13.18) | 5.48(2.27, 13.24) | 18.22 | 2.45(1.29) | 5.46(2.62) | female |
| gastrointestinal disorders | gastrointestinal wall thickening | 5 | 11.74(4.87, 28.31) | 11.74(4.86, 28.36) | 48.71 | 3.54(2.38) | 11.65(5.58) | female |
| nervous system disorders | quadriplegia | 5 | 7.65(3.18, 18.42) | 7.65(3.17, 18.48) | 28.73 | 2.93(1.77) | 7.61(3.65) | female |
| general disorders and administration site conditions | infusion site thrombosis | 6 | 66.34(29.25, 150.47) | 66.32(29.12, 151.06) | 368.61 | 5.99(4.89) | 63.38(31.94) | female |
| skin and subcutaneous tissue disorders | vascular purpura | 4 | 10.12(3.78, 27.06) | 10.12(3.8, 26.96) | 32.63 | 3.33(2.06) | 10.05(4.41) | female |
| skin and subcutaneous tissue disorders | epidermal necrosis | 4 | 12.94(4.83, 34.64) | 12.94(4.86, 34.48) | 43.66 | 3.68(2.41) | 12.83(5.63) | female |
| congenital, familial and genetic disorders | porphyria acute | 6 | 19.73(8.81, 44.17) | 19.73(8.83, 44.07) | 105.18 | 4.28(3.21) | 19.47(9.92) | female |
| infections and infestations | clostridium colitis | 3 | 6.62(2.13, 20.59) | 6.62(2.12, 20.63) | 14.25 | 2.72(1.3) | 6.6(2.55) | female |
| eye disorders | limbal swelling | 3 | 421.21(115.91, 1530.62) | 421.15(115.51, 1535.47) | 967.27 | 8.34(6.74) | 324.19(110.13) | female |
| investigations | mean cell haemoglobin concentration increased | 3 | 18.08(5.79, 56.46) | 18.08(5.8, 56.35) | 47.78 | 4.16(2.73) | 17.86(6.89) | female |
| eye disorders | corneal opacity | 3 | 7.84(2.52, 24.4) | 7.84(2.52, 24.44) | 17.81 | 2.96(1.55) | 7.8(3.02) | female |
| gastrointestinal disorders | dysbiosis | 17 | 97.49(59.62, 159.41) | 97.41(59.68, 159) | 1516.88 | 6.51(5.82) | 91.15(60.4) | female |
| respiratory, thoracic and mediastinal disorders | neonatal respiratory distress | 3 | 20.65(6.6, 64.56) | 20.64(6.62, 64.33) | 55.27 | 4.35(2.92) | 20.36(7.84) | female |
| injury, poisoning and procedural complications | wrong rate | 4 | 124.81(44.88, 347.06) | 124.79(45.03, 345.79) | 451.08 | 6.84(5.52) | 114.68(48.74) | female |
| renal and urinary disorders | kidney fibrosis | 8 | 17.69(8.81, 35.54) | 17.69(8.74, 35.82) | 124.38 | 4.13(3.18) | 17.48(9.75) | female |
| renal and urinary disorders | renal arteriosclerosis | 4 | 58.5(21.52, 159.08) | 58.49(21.53, 158.93) | 217 | 5.81(4.52) | 56.19(24.33) | female |
| renal and urinary disorders | renal tubular atrophy | 8 | 38.34(18.99, 77.41) | 38.33(18.93, 77.62) | 283.12 | 5.22(4.27) | 37.34(20.74) | female |
| blood and lymphatic system disorders | thrombotic microangiopathy | 20 | 7.74(4.98, 12.01) | 7.73(5.02, 11.9) | 116.57 | 2.94(2.32) | 7.69(5.33) | female |
| vascular disorders | necrosis ischaemic | 3 | 19.15(6.13, 59.83) | 19.14(6.14, 59.66) | 50.89 | 4.24(2.81) | 18.9(7.28) | female |
| renal and urinary disorders | crystal nephropathy | 7 | 40.12(18.93, 85.06) | 40.11(19.05, 84.47) | 259.53 | 5.29(4.27) | 39.02(20.81) | female |
| injury, poisoning and procedural complications | chemical peritonitis | 4 | 78.01(28.5, 213.52) | 77.99(28.7, 211.91) | 288.01 | 6.21(4.9) | 73.94(31.84) | female |
| infections and infestations | bacterial disease carrier | 5 | 16.96(7.02, 40.96) | 16.95(7.02, 40.95) | 74.17 | 4.07(2.9) | 16.76(8.01) | female |
| renal and urinary disorders | iga nephropathy | 9 | 32.41(16.73, 62.77) | 32.4(16.64, 63.09) | 267.67 | 4.99(4.08) | 31.69(18.23) | female |
| cardiac disorders | restrictive cardiomyopathy | 4 | 43.88(16.22, 118.71) | 43.87(16.15, 119.2) | 162.49 | 5.41(4.12) | 42.57(18.51) | female |
| eye disorders | haemorrhagic occlusive retinal vasculitis | 14 | 1966.67(873.46, 4428.1) | 1965.36(879.92, 4389.76) | 11452.97 | 9.68(8.75) | 819.49(415.53) | female |
| hepatobiliary disorders | hepatic cytolysis | 13 | 4.23(2.45, 7.29) | 4.23(2.44, 7.32) | 31.95 | 2.08(1.32) | 4.22(2.67) | female |
| infections and infestations | disseminated coccidioidomycosis | 3 | 150.43(45.73, 494.86) | 150.41(45.5, 497.19) | 402.16 | 7.09(5.59) | 135.95(50.2) | female |
| renal and urinary disorders | glomerulonephritis proliferative | 3 | 31.91(10.16, 100.22) | 31.91(10.24, 99.46) | 87.81 | 4.96(3.53) | 31.22(11.98) | female |
| infections and infestations | endophthalmitis | 6 | 4.73(2.12, 10.54) | 4.73(2.12, 10.56) | 17.59 | 2.24(1.17) | 4.72(2.41) | female |
| infections and infestations | weissella infection | 6 | 2808.46(702.33, 11230.49) | 2807.66(698.2, 11290.38) | 5611.33 | 9.87(8.48) | 936.55(293.68) | female |
| investigations | drug trough level | 3 | 421.21(115.91, 1530.62) | 421.15(115.51, 1535.47) | 967.27 | 8.34(6.74) | 324.19(110.13) | female |
| skin and subcutaneous tissue disorders | erythrosis | 4 | 80.23(29.29, 219.78) | 80.22(29.52, 217.97) | 296.01 | 6.25(4.94) | 75.94(32.68) | female |
| hepatobiliary disorders | acute on chronic liver failure | 5 | 87.76(35.55, 216.64) | 87.74(35.62, 216.15) | 403.53 | 6.37(5.17) | 82.64(38.8) | female |
| injury, poisoning and procedural complications | poor quality product administered | 10 | 3.68(1.98, 6.84) | 3.67(1.96, 6.87) | 19.42 | 1.87(1.02) | 3.67(2.18) | female |
| nervous system disorders | spinal stroke | 8 | 468.12(210.28, 1042.12) | 467.94(209.5, 1045.18) | 2795.68 | 8.46(7.39) | 351.21(179.78) | female |
| nervous system disorders | paraplegia | 7 | 8.07(3.84, 16.97) | 8.07(3.83, 17) | 43.1 | 3(2) | 8.03(4.31) | female |
| infections and infestations | overgrowth fungal | 3 | 102.73(31.81, 331.79) | 102.72(31.69, 332.95) | 281.58 | 6.58(5.11) | 95.78(35.91) | female |
| infections and infestations | rhinocerebral mucormycosis | 11 | 181.77(96.98, 340.67) | 181.67(97.03, 340.15) | 1749.99 | 7.33(6.47) | 160.97(95.16) | female |
| infections and infestations | pseudomonal sepsis | 3 | 7.77(2.5, 24.17) | 7.77(2.49, 24.22) | 17.6 | 2.95(1.53) | 7.73(2.99) | female |
| injury, poisoning and procedural complications | incision site swelling | 5 | 49.79(20.41, 121.5) | 49.78(20.21, 122.64) | 230.82 | 5.59(4.41) | 48.11(22.81) | female |
| general disorders and administration site conditions | implant site bruising | 5 | 12.38(5.13, 29.87) | 12.38(5.12, 29.91) | 51.85 | 3.62(2.46) | 12.28(5.88) | female |
| respiratory, thoracic and mediastinal disorders | eosinophilic pneumonia acute | 3 | 13.25(4.25, 41.29) | 13.24(4.25, 41.27) | 33.64 | 3.71(2.29) | 13.13(5.07) | female |
| investigations | allergy test positive | 5 | 32.21(13.27, 78.16) | 32.2(13.33, 77.79) | 147.76 | 4.98(3.81) | 31.5(15) | female |
| nervous system disorders | intraventricular haemorrhage neonatal | 3 | 32.4(10.31, 101.78) | 32.4(10.4, 100.98) | 89.22 | 4.99(3.55) | 31.69(12.16) | female |
| investigations | prothrombin time ratio decreased | 3 | 14.52(4.66, 45.3) | 14.52(4.66, 45.26) | 37.39 | 3.85(2.42) | 14.38(5.55) | female |
| general disorders and administration site conditions | facial discomfort | 4 | 19.57(7.29, 52.5) | 19.57(7.34, 52.14) | 69.5 | 4.27(3) | 19.31(8.46) | female |
| gastrointestinal disorders | dyschezia | 5 | 5.38(2.23, 12.94) | 5.37(2.22, 12.97) | 17.74 | 2.42(1.26) | 5.36(2.57) | female |
| infections and infestations | superinfection | 4 | 8.72(3.26, 23.31) | 8.72(3.27, 23.23) | 27.17 | 3.12(1.85) | 8.67(3.81) | female |
| respiratory, thoracic and mediastinal disorders | bronchial secretion retention | 4 | 8.97(3.36, 23.98) | 8.97(3.37, 23.9) | 28.15 | 3.16(1.89) | 8.92(3.92) | female |
| renal and urinary disorders | bladder sphincter atony | 3 | 51.37(16.23, 162.58) | 51.36(16.16, 163.25) | 142.91 | 5.63(4.19) | 49.58(18.91) | female |
| congenital, familial and genetic disorders | fanconi syndrome | 12 | 33.25(18.75, 58.94) | 33.23(18.82, 58.67) | 366.42 | 5.02(4.23) | 32.48(20.12) | female |
| renal and urinary disorders | fanconi syndrome acquired | 4 | 10.64(3.98, 28.45) | 10.64(3.99, 28.35) | 34.65 | 3.4(2.13) | 10.56(4.64) | female |
| hepatobiliary disorders | hepatic ischaemia | 3 | 29.87(9.52, 93.75) | 29.87(9.58, 93.1) | 81.96 | 4.87(3.44) | 29.27(11.24) | female |
| investigations | false positive investigation result | 3 | 10.03(3.22, 31.22) | 10.03(3.22, 31.26) | 24.21 | 3.32(1.9) | 9.96(3.85) | female |
| infections and infestations | septic pulmonary embolism | 3 | 59.33(18.68, 188.36) | 59.32(18.66, 188.55) | 165.03 | 5.83(4.38) | 56.95(21.66) | female |
| renal and urinary disorders | acute kidney injury | 1178 | 15.3(14.42, 16.23) | 14.61(13.78, 15.49) | 14692.18 | 3.84(3.76) | 14.34(13.65) | male |
| immune system disorders | drug hypersensitivity | 447 | 8.88(8.08, 9.75) | 8.73(7.92, 9.63) | 3030.69 | 3.11(2.98) | 8.64(7.99) | male |
| skin and subcutaneous tissue disorders | drug reaction with eosinophilia and systemic symptoms | 622 | 54.36(50.06, 59.03) | 52.99(48.99, 57.31) | 29606.62 | 5.63(5.51) | 49.49(46.19) | male |
| skin and subcutaneous tissue disorders | linear iga disease | 297 | 633.5(542.35, 739.97) | 625.77(534.96, 732) | 99958.04 | 8.4(8.2) | 338.09(296.88) | male |
| renal and urinary disorders | nephropathy toxic | 200 | 38.15(33.08, 44) | 37.84(32.99, 43.4) | 6823.53 | 5.17(4.97) | 36.04(31.98) | male |
| investigations | blood creatinine increased | 271 | 6.72(5.96, 7.58) | 6.65(5.91, 7.48) | 1292.34 | 2.72(2.55) | 6.6(5.97) | male |
| blood and lymphatic system disorders | thrombocytopenia | 272 | 4.7(4.17, 5.3) | 4.66(4.14, 5.24) | 779.27 | 2.21(2.04) | 4.64(4.2) | male |
| renal and urinary disorders | renal tubular necrosis | 288 | 50.89(45.14, 57.39) | 50.3(44.72, 56.58) | 13026.61 | 5.56(5.39) | 47.14(42.63) | male |
| investigations | drug level increased | 163 | 19.01(16.26, 22.22) | 18.89(16.15, 22.1) | 2692.83 | 4.2(3.98) | 18.44(16.18) | male |
| immune system disorders | anaphylactic reaction | 189 | 9.72(8.41, 11.22) | 9.65(8.41, 11.07) | 1447.78 | 3.25(3.05) | 9.54(8.46) | male |
| renal and urinary disorders | renal impairment | 201 | 4.35(3.79, 5) | 4.33(3.77, 4.97) | 511.84 | 2.11(1.91) | 4.31(3.83) | male |
| blood and lymphatic system disorders | eosinophilia | 188 | 19.04(16.46, 22.02) | 18.9(16.48, 21.68) | 3108.11 | 4.21(4) | 18.45(16.34) | male |
| general disorders and administration site conditions | treatment failure | 122 | 4.2(3.52, 5.02) | 4.19(3.51, 5) | 294.66 | 2.06(1.8) | 4.17(3.59) | male |
| blood and lymphatic system disorders | leukopenia | 159 | 6.97(5.96, 8.16) | 6.93(5.92, 8.11) | 800.65 | 2.78(2.56) | 6.88(6.03) | male |
| renal and urinary disorders | tubulointerstitial nephritis | 190 | 20.85(18.04, 24.1) | 20.7(18.05, 23.74) | 3465.22 | 4.33(4.12) | 20.16(17.86) | male |
| infections and infestations | clostridium difficile infection | 90 | 10.56(8.57, 13.01) | 10.53(8.49, 13.06) | 765.22 | 3.38(3.08) | 10.39(8.73) | male |
| skin and subcutaneous tissue disorders | rash maculo-papular | 186 | 16.8(14.52, 19.43) | 16.67(14.53, 19.12) | 2680.91 | 4.03(3.82) | 16.33(14.45) | male |
| skin and subcutaneous tissue disorders | rash erythematous | 111 | 6.35(5.26, 7.65) | 6.32(5.2, 7.69) | 493.45 | 2.65(2.38) | 6.28(5.37) | male |
| infections and infestations | septic shock | 123 | 5(4.19, 5.98) | 4.98(4.17, 5.94) | 389.47 | 2.31(2.05) | 4.96(4.27) | male |
| general disorders and administration site conditions | multiple organ dysfunction syndrome | 124 | 8.53(7.14, 10.18) | 8.49(7.12, 10.13) | 810.2 | 3.07(2.82) | 8.4(7.24) | male |
| skin and subcutaneous tissue disorders | toxic epidermal necrolysis | 116 | 16.65(13.84, 20.02) | 16.57(13.89, 19.77) | 1660.47 | 4.02(3.76) | 16.23(13.91) | male |
| skin and subcutaneous tissue disorders | drug eruption | 90 | 10.51(8.53, 12.95) | 10.47(8.44, 12.99) | 760.72 | 3.37(3.07) | 10.34(8.69) | male |
| skin and subcutaneous tissue disorders | acute generalised exanthematous pustulosis | 97 | 29.72(24.25, 36.43) | 29.61(24.34, 36.02) | 2577.71 | 4.83(4.54) | 28.5(24.04) | male |
| blood and lymphatic system disorders | pancytopenia | 117 | 3.86(3.22, 4.63) | 3.85(3.23, 4.59) | 245.42 | 1.94(1.68) | 3.83(3.29) | male |
| general disorders and administration site conditions | drug resistance | 126 | 10.17(8.52, 12.13) | 10.12(8.48, 12.07) | 1022.02 | 3.32(3.07) | 10(8.63) | male |
| infections and infestations | staphylococcal infection | 104 | 6.08(5.01, 7.38) | 6.06(4.98, 7.37) | 436.08 | 2.59(2.31) | 6.02(5.12) | male |
| eye disorders | retinal vasculitis | 40 | 115.2(82.53, 160.82) | 115.02(82.43, 160.5) | 3908.03 | 6.64(6.17) | 99.56(75.31) | male |
| hepatobiliary disorders | drug-induced liver injury | 132 | 11.2(9.43, 13.31) | 11.15(9.35, 13.3) | 1201.86 | 3.46(3.21) | 11(9.52) | male |
| skin and subcutaneous tissue disorders | stevens-johnson syndrome | 90 | 8.63(7.01, 10.63) | 8.6(6.93, 10.67) | 597.94 | 3.09(2.79) | 8.51(7.15) | male |
| infections and infestations | pathogen resistance | 72 | 17.13(13.56, 21.65) | 17.08(13.5, 21.61) | 1065.69 | 4.06(3.73) | 16.72(13.75) | male |
| skin and subcutaneous tissue disorders | vancomycin infusion reaction | 36 | 644.76(412, 1009.01) | 643.81(410.18, 1010.5) | 12302.8 | 8.42(7.86) | 343.27(235.99) | male |
| investigations | antibiotic level above therapeutic | 69 | 695.02(500, 966.1) | 693.05(496.66, 967.1) | 24513.03 | 8.48(8.07) | 356.77(270.84) | male |
| infections and infestations | enterococcal infection | 53 | 18.72(14.25, 24.6) | 18.68(14.2, 24.58) | 865.11 | 4.19(3.8) | 18.24(14.52) | male |
| skin and subcutaneous tissue disorders | toxic skin eruption | 65 | 12.55(9.82, 16.04) | 12.52(9.9, 15.84) | 677.3 | 3.62(3.27) | 12.32(10.03) | male |
| respiratory, thoracic and mediastinal disorders | hypoxia | 65 | 3.4(2.67, 4.34) | 3.4(2.69, 4.3) | 109.4 | 1.76(1.41) | 3.38(2.76) | male |
| general disorders and administration site conditions | face oedema | 50 | 7.3(5.52, 9.64) | 7.28(5.53, 9.58) | 268.48 | 2.85(2.45) | 7.22(5.72) | male |
| blood and lymphatic system disorders | leukocytosis | 48 | 4.73(3.56, 6.29) | 4.72(3.59, 6.21) | 140.08 | 2.23(1.83) | 4.7(3.71) | male |
| vascular disorders | shock | 57 | 4.76(3.67, 6.18) | 4.75(3.68, 6.13) | 168 | 2.24(1.87) | 4.73(3.8) | male |
| skin and subcutaneous tissue disorders | erythema multiforme | 52 | 11.12(8.46, 14.63) | 11.1(8.44, 14.6) | 471 | 3.45(3.06) | 10.95(8.71) | male |
| infections and infestations | candida infection | 57 | 9.48(7.3, 12.32) | 9.46(7.33, 12.21) | 425.97 | 3.23(2.85) | 9.35(7.52) | male |
| skin and subcutaneous tissue disorders | rash morbilliform | 61 | 45.15(34.86, 58.49) | 45.04(34.91, 58.11) | 2475.04 | 5.41(5.04) | 42.49(34.22) | male |
| investigations | blood urea increased | 50 | 4.57(3.46, 6.04) | 4.56(3.47, 6) | 138.24 | 2.18(1.78) | 4.54(3.6) | male |
| infections and infestations | clostridium difficile colitis | 33 | 6.59(4.68, 9.29) | 6.58(4.72, 9.18) | 154.87 | 2.71(2.22) | 6.53(4.9) | male |
| investigations | drug level above therapeutic | 25 | 12.8(8.62, 19.02) | 12.79(8.64, 18.93) | 267.11 | 3.65(3.09) | 12.59(9.04) | male |
| vascular disorders | haemodynamic instability | 44 | 11(8.16, 14.81) | 10.98(8.18, 14.73) | 393.18 | 3.44(3.01) | 10.83(8.44) | male |
| renal and urinary disorders | oliguria | 40 | 12.22(8.94, 16.7) | 12.2(8.92, 16.69) | 404.61 | 3.59(3.14) | 12.02(9.25) | male |
| cardiac disorders | kounis syndrome | 68 | 47.03(36.79, 60.12) | 46.9(37.07, 59.34) | 2871.46 | 5.46(5.11) | 44.14(35.95) | male |
| injury, poisoning and procedural complications | incorrect drug administration rate | 21 | 12.9(8.37, 19.86) | 12.89(8.38, 19.84) | 226.26 | 3.66(3.06) | 12.68(8.84) | male |
| blood and lymphatic system disorders | immune thrombocytopenia | 64 | 36.44(28.34, 46.85) | 36.35(28.17, 46.9) | 2096.34 | 5.12(4.76) | 34.68(28.1) | male |
| vascular disorders | circulatory collapse | 39 | 4.15(3.03, 5.69) | 4.14(3.03, 5.66) | 92.57 | 2.05(1.6) | 4.13(3.17) | male |
| renal and urinary disorders | anuria | 31 | 6.3(4.43, 8.98) | 6.3(4.43, 8.97) | 137.02 | 2.64(2.14) | 6.25(4.65) | male |
| infections and infestations | bacteraemia | 27 | 4.18(2.86, 6.1) | 4.18(2.88, 6.07) | 64.9 | 2.06(1.52) | 4.16(3.03) | male |
| skin and subcutaneous tissue disorders | purpura | 38 | 7.6(5.52, 10.46) | 7.59(5.55, 10.39) | 215.09 | 2.91(2.45) | 7.52(5.75) | male |
| infections and infestations | staphylococcal bacteraemia | 29 | 10.97(7.6, 15.84) | 10.96(7.55, 15.91) | 258.67 | 3.43(2.91) | 10.81(7.96) | male |
| infections and infestations | endocarditis | 27 | 8.49(5.81, 12.42) | 8.49(5.85, 12.32) | 176.28 | 3.07(2.53) | 8.4(6.11) | male |
| immune system disorders | type iv hypersensitivity reaction | 22 | 19.96(13.06, 30.49) | 19.94(12.96, 30.69) | 385.31 | 4.28(3.68) | 19.44(13.64) | male |
| eye disorders | retinal vascular occlusion | 4 | 14.45(5.37, 38.88) | 14.45(5.42, 38.5) | 49.1 | 3.83(2.55) | 14.19(6.2) | male |
| infections and infestations | systemic candida | 20 | 16.73(10.74, 26.07) | 16.72(10.65, 26.24) | 289.04 | 4.03(3.41) | 16.37(11.3) | male |
| ear and labyrinth disorders | ototoxicity | 34 | 41.61(29.44, 58.79) | 41.55(29.2, 59.13) | 1273.39 | 5.3(4.81) | 39.37(29.48) | male |
| eye disorders | retinal haemorrhage | 13 | 3.66(2.12, 6.31) | 3.66(2.11, 6.34) | 24.98 | 1.87(1.11) | 3.64(2.31) | male |
| infections and infestations | klebsiella infection | 26 | 9.59(6.51, 14.12) | 9.58(6.47, 14.18) | 197.24 | 3.24(2.7) | 9.47(6.85) | male |
| skin and subcutaneous tissue disorders | dermatitis exfoliative | 23 | 6.97(4.62, 10.51) | 6.96(4.61, 10.5) | 116.36 | 2.79(2.21) | 6.91(4.9) | male |
| skin and subcutaneous tissue disorders | henoch-schonlein purpura | 32 | 24.1(16.94, 34.27) | 24.06(16.91, 34.24) | 684.96 | 4.54(4.04) | 23.33(17.37) | male |
| vascular disorders | haemorrhagic vasculitis | 3 | 115.79(34.26, 391.3) | 115.77(34.34, 390.26) | 294.8 | 6.65(5.11) | 100.12(36.14) | male |
| investigations | eosinophil count increased | 34 | 7.54(5.38, 10.57) | 7.53(5.4, 10.51) | 190.67 | 2.9(2.42) | 7.47(5.63) | male |
| skin and subcutaneous tissue disorders | hypersensitivity vasculitis | 22 | 15.54(10.18, 23.71) | 15.53(10.09, 23.9) | 292.8 | 3.93(3.33) | 15.22(10.69) | male |
| investigations | drug level below therapeutic | 15 | 6.27(3.77, 10.43) | 6.27(3.77, 10.44) | 65.89 | 2.64(1.93) | 6.23(4.07) | male |
| skin and subcutaneous tissue disorders | cutaneous vasculitis | 25 | 17.71(11.91, 26.34) | 17.69(11.95, 26.18) | 384.5 | 4.11(3.55) | 17.3(12.41) | male |
| immune system disorders | anaphylactoid reaction | 14 | 7.91(4.67, 13.4) | 7.91(4.66, 13.43) | 83.59 | 2.97(2.24) | 7.83(5.04) | male |
| renal and urinary disorders | renal tubular disorder | 14 | 7.33(4.33, 12.41) | 7.33(4.32, 12.44) | 75.74 | 2.86(2.13) | 7.26(4.68) | male |
| respiratory, thoracic and mediastinal disorders | pulmonary haemorrhage | 15 | 3.11(1.87, 5.17) | 3.11(1.87, 5.18) | 21.42 | 1.63(0.93) | 3.1(2.03) | male |
| general disorders and administration site conditions | multiple-drug resistance | 20 | 12.65(8.13, 19.69) | 12.64(8.05, 19.84) | 210.8 | 3.64(3.01) | 12.44(8.6) | male |
| skin and subcutaneous tissue disorders | skin plaque | 13 | 3.29(1.91, 5.68) | 3.29(1.9, 5.7) | 20.64 | 1.71(0.96) | 3.28(2.08) | male |
| cardiac disorders | pulseless electrical activity | 13 | 5.74(3.33, 9.91) | 5.74(3.32, 9.94) | 50.48 | 2.51(1.75) | 5.7(3.61) | male |
| infections and infestations | pseudomonas infection | 17 | 3.68(2.29, 5.93) | 3.68(2.3, 5.89) | 33.02 | 1.87(1.21) | 3.67(2.46) | male |
| infections and infestations | acinetobacter infection | 9 | 16.14(8.34, 31.25) | 16.13(8.28, 31.41) | 125.02 | 3.98(3.08) | 15.81(9.1) | male |
| gastrointestinal disorders | noninfectious peritonitis | 9 | 66.68(33.7, 131.95) | 66.66(33.57, 132.37) | 533.55 | 5.94(5) | 61.19(34.56) | male |
| investigations | antibiotic level below therapeutic | 14 | 256.78(139.69, 472) | 256.63(139.78, 471.18) | 2640.65 | 7.57(6.75) | 190.35(114.38) | male |
| hepatobiliary disorders | hypertransaminasaemia | 13 | 4.48(2.6, 7.73) | 4.48(2.59, 7.76) | 34.92 | 2.16(1.4) | 4.46(2.82) | male |
| general disorders and administration site conditions | systemic inflammatory response syndrome | 10 | 4.83(2.59, 8.99) | 4.82(2.57, 9.02) | 30.12 | 2.26(1.41) | 4.8(2.85) | male |
| gastrointestinal disorders | megacolon | 14 | 15.47(9.11, 26.27) | 15.46(9.11, 26.24) | 185.44 | 3.92(3.18) | 15.16(9.73) | male |
| infections and infestations | stenotrophomonas infection | 10 | 12.18(6.52, 22.76) | 12.18(6.51, 22.81) | 100.95 | 3.58(2.72) | 12(7.11) | male |
| renal and urinary disorders | azotaemia | 9 | 3.64(1.89, 7.01) | 3.64(1.91, 6.95) | 17.15 | 1.86(0.96) | 3.63(2.1) | male |
| general disorders and administration site conditions | localised oedema | 8 | 3.83(1.91, 7.68) | 3.83(1.93, 7.61) | 16.67 | 1.93(0.99) | 3.82(2.14) | male |
| infections and infestations | mucormycosis | 10 | 5.61(3.01, 10.46) | 5.61(3, 10.5) | 37.6 | 2.48(1.62) | 5.57(3.31) | male |
| gastrointestinal disorders | enterocolitis haemorrhagic | 9 | 12.36(6.4, 23.89) | 12.36(6.35, 24.07) | 92.39 | 3.61(2.7) | 12.17(7.01) | male |
| infections and infestations | septic embolus | 8 | 15.4(7.65, 31.02) | 15.4(7.6, 31.19) | 105.47 | 3.92(2.96) | 15.1(8.4) | male |
| infections and infestations | enterobacter infection | 9 | 9.44(4.89, 18.23) | 9.44(4.85, 18.38) | 67.06 | 3.22(2.32) | 9.33(5.38) | male |
| infections and infestations | intervertebral discitis | 7 | 6.59(3.13, 13.87) | 6.59(3.13, 13.88) | 32.89 | 2.71(1.7) | 6.54(3.51) | male |
| investigations | clostridium test positive | 6 | 5.69(2.55, 12.71) | 5.69(2.55, 12.71) | 23.02 | 2.5(1.43) | 5.66(2.89) | male |
| infections and infestations | meningitis bacterial | 5 | 6.91(2.86, 16.66) | 6.9(2.86, 16.67) | 25.01 | 2.78(1.61) | 6.85(3.28) | male |
| skin and subcutaneous tissue disorders | urticaria | 167 | 3.24(2.78, 3.78) | 3.23(2.76, 3.78) | 255.99 | 1.69(1.47) | 3.22(2.83) | male |
| skin and subcutaneous tissue disorders | rash | 535 | 3.27(3, 3.56) | 3.22(2.98, 3.48) | 818.68 | 1.68(1.56) | 3.21(2.98) | male |
| blood and lymphatic system disorders | neutropenia | 216 | 3.59(3.14, 4.11) | 3.57(3.11, 4.09) | 398.51 | 1.83(1.64) | 3.56(3.18) | male |
| skin and subcutaneous tissue disorders | erythema | 222 | 3.06(2.68, 3.49) | 3.04(2.65, 3.49) | 303.64 | 1.6(1.41) | 3.03(2.71) | male |
| respiratory, thoracic and mediastinal disorders | respiratory distress | 57 | 3.75(2.89, 4.86) | 3.74(2.9, 4.83) | 113.93 | 1.9(1.53) | 3.73(3) | male |
| skin and subcutaneous tissue disorders | rash pruritic | 67 | 3.31(2.6, 4.21) | 3.3(2.61, 4.18) | 107.16 | 1.72(1.37) | 3.29(2.69) | male |
| infections and infestations | cardiac valve vegetation | 13 | 40.24(23.02, 70.34) | 40.22(23.23, 69.63) | 471.33 | 5.25(4.48) | 38.18(23.93) | male |
| blood and lymphatic system disorders | agranulocytosis | 55 | 5.71(4.38, 7.45) | 5.7(4.33, 7.5) | 211.5 | 2.5(2.12) | 5.66(4.53) | male |
| immune system disorders | anaphylactic shock | 59 | 5.57(4.31, 7.19) | 5.56(4.31, 7.17) | 218.87 | 2.47(2.1) | 5.52(4.46) | male |
| injury, poisoning and procedural complications | infusion related reaction | 110 | 5.06(4.2, 6.11) | 5.04(4.14, 6.13) | 354.57 | 2.33(2.06) | 5.02(4.29) | male |
| hepatobiliary disorders | cholestasis | 52 | 5.07(3.86, 6.67) | 5.06(3.85, 6.66) | 168.54 | 2.33(1.94) | 5.04(4.01) | male |
| skin and subcutaneous tissue disorders | petechiae | 37 | 7.75(5.61, 10.72) | 7.74(5.55, 10.8) | 215.02 | 2.94(2.48) | 7.67(5.85) | male |
| infections and infestations | clostridial infection | 13 | 7.43(4.3, 12.84) | 7.43(4.29, 12.86) | 71.61 | 2.88(2.12) | 7.36(4.66) | male |
| respiratory, thoracic and mediastinal disorders | acute respiratory distress syndrome | 59 | 5.81(4.49, 7.51) | 5.8(4.5, 7.48) | 232.4 | 2.53(2.16) | 5.76(4.65) | male |
| nervous system disorders | encephalopathy | 49 | 3.79(2.86, 5.03) | 3.79(2.88, 4.99) | 100.12 | 1.92(1.51) | 3.77(2.98) | male |
| nervous system disorders | metabolic encephalopathy | 9 | 5.91(3.07, 11.39) | 5.91(3.1, 11.28) | 36.4 | 2.55(1.65) | 5.87(3.39) | male |
| immune system disorders | type i hypersensitivity | 14 | 11.93(7.03, 20.23) | 11.92(7.02, 20.24) | 137.85 | 3.55(2.82) | 11.75(7.55) | male |
| skin and subcutaneous tissue disorders | dermatitis bullous | 42 | 11.57(8.53, 15.7) | 11.56(8.45, 15.82) | 398.74 | 3.51(3.07) | 11.39(8.83) | male |
| nervous system disorders | unresponsive to stimuli | 47 | 3.4(2.56, 4.53) | 3.4(2.53, 4.56) | 79.27 | 1.76(1.35) | 3.39(2.67) | male |
| investigations | pulse absent | 19 | 6.68(4.25, 10.49) | 6.67(4.25, 10.47) | 90.8 | 2.73(2.09) | 6.62(4.54) | male |
| skin and subcutaneous tissue disorders | exfoliative rash | 6 | 4.64(2.08, 10.35) | 4.64(2.08, 10.36) | 17 | 2.21(1.13) | 4.61(2.36) | male |
| investigations | transaminases increased | 49 | 4.46(3.36, 5.9) | 4.45(3.38, 5.86) | 130.32 | 2.15(1.75) | 4.43(3.5) | male |
| injury, poisoning and procedural complications | procedural hypotension | 3 | 6.79(2.18, 21.16) | 6.79(2.18, 21.16) | 14.67 | 2.75(1.33) | 6.74(2.6) | male |
| investigations | blood creatine increased | 13 | 4.85(2.81, 8.37) | 4.85(2.8, 8.4) | 39.45 | 2.27(1.51) | 4.82(3.06) | male |
| general disorders and administration site conditions | infusion site extravasation | 13 | 5.58(3.23, 9.63) | 5.58(3.22, 9.66) | 48.47 | 2.47(1.71) | 5.54(3.51) | male |
| respiratory, thoracic and mediastinal disorders | bronchospasm | 24 | 3.86(2.58, 5.76) | 3.85(2.6, 5.7) | 50.43 | 1.94(1.37) | 3.84(2.74) | male |
| infections and infestations | cytomegalovirus colitis | 6 | 5.12(2.29, 11.42) | 5.12(2.29, 11.44) | 19.73 | 2.35(1.27) | 5.09(2.6) | male |
| renal and urinary disorders | nephropathy | 79 | 14.24(11.4, 17.8) | 14.2(11.45, 17.62) | 951.23 | 3.8(3.48) | 13.95(11.58) | male |
| general disorders and administration site conditions | extravasation | 20 | 12.48(8.02, 19.42) | 12.47(7.94, 19.57) | 207.49 | 3.62(3) | 12.28(8.48) | male |
| infections and infestations | nosocomial infection | 7 | 5.94(2.82, 12.5) | 5.94(2.82, 12.51) | 28.53 | 2.56(1.56) | 5.9(3.17) | male |
| hepatobiliary disorders | hepatocellular injury | 53 | 7.18(5.48, 9.42) | 7.17(5.45, 9.43) | 278.83 | 2.83(2.44) | 7.11(5.67) | male |
| general disorders and administration site conditions | potentiating drug interaction | 10 | 5.61(3.01, 10.45) | 5.61(3, 10.5) | 37.56 | 2.48(1.62) | 5.57(3.31) | male |
| skin and subcutaneous tissue disorders | rash vesicular | 9 | 4.83(2.5, 9.29) | 4.82(2.52, 9.2) | 27.1 | 2.26(1.37) | 4.8(2.77) | male |
| hepatobiliary disorders | liver injury | 34 | 3.9(2.79, 5.47) | 3.9(2.79, 5.44) | 72.92 | 1.96(1.48) | 3.88(2.93) | male |
| investigations | creatinine renal clearance decreased | 7 | 3.98(1.89, 8.36) | 3.98(1.89, 8.38) | 15.51 | 1.99(0.98) | 3.96(2.13) | male |
| infections and infestations | pseudomembranous colitis | 6 | 4.87(2.18, 10.88) | 4.87(2.18, 10.88) | 18.34 | 2.28(1.2) | 4.85(2.48) | male |
| investigations | enterococcus test positive | 5 | 12.91(5.33, 31.26) | 12.91(5.34, 31.19) | 53.98 | 3.67(2.5) | 12.7(6.06) | male |
| respiratory, thoracic and mediastinal disorders | eosinophilic pneumonia | 18 | 10.13(6.36, 16.13) | 10.12(6.32, 16.2) | 145.96 | 3.32(2.67) | 10(6.77) | male |
| blood and lymphatic system disorders | haemolytic anaemia | 22 | 4.6(3.03, 7) | 4.6(3.05, 6.94) | 61.6 | 2.19(1.6) | 4.58(3.22) | male |
| gastrointestinal disorders | lip oedema | 12 | 6.18(3.5, 10.91) | 6.18(3.5, 10.91) | 51.66 | 2.62(1.83) | 6.14(3.81) | male |
| general disorders and administration site conditions | administration site extravasation | 7 | 16.61(7.85, 35.15) | 16.61(7.89, 34.98) | 100.42 | 4.02(3.01) | 16.26(8.69) | male |
| investigations | laboratory test interference | 5 | 11.11(4.59, 26.88) | 11.11(4.6, 26.84) | 45.31 | 3.45(2.29) | 10.96(5.23) | male |
| nervous system disorders | posterior reversible encephalopathy syndrome | 12 | 3.4(1.93, 5.99) | 3.39(1.92, 5.98) | 20.18 | 1.76(0.97) | 3.38(2.1) | male |
| infections and infestations | enterococcal bacteraemia | 18 | 30.15(18.82, 48.32) | 30.13(18.82, 48.23) | 486.98 | 4.86(4.19) | 28.98(19.53) | male |
| renal and urinary disorders | myeloma cast nephropathy | 15 | 203.8(115, 361.16) | 203.67(115.37, 359.57) | 2367.54 | 7.32(6.53) | 159.61(98.89) | male |
| infections and infestations | fungal sepsis | 4 | 9.62(3.59, 25.79) | 9.62(3.61, 25.63) | 30.48 | 3.25(1.97) | 9.5(4.16) | male |
| general disorders and administration site conditions | drug ineffective for unapproved indication | 144 | 6.91(5.86, 8.14) | 6.87(5.87, 8.04) | 716.28 | 2.77(2.53) | 6.82(5.94) | male |
| skin and subcutaneous tissue disorders | skin necrosis | 22 | 9.36(6.14, 14.25) | 9.35(6.2, 14.11) | 162.03 | 3.21(2.62) | 9.25(6.5) | male |
| hepatobiliary disorders | mixed liver injury | 5 | 4.23(1.76, 10.19) | 4.23(1.75, 10.22) | 12.25 | 2.07(0.92) | 4.21(2.02) | male |
| blood and lymphatic system disorders | bicytopenia | 8 | 8.81(4.39, 17.69) | 8.81(4.35, 17.84) | 54.71 | 3.12(2.17) | 8.71(4.86) | male |
| hepatobiliary disorders | venoocclusive liver disease | 15 | 5.65(3.4, 9.38) | 5.64(3.39, 9.39) | 56.87 | 2.49(1.78) | 5.61(3.67) | male |
| infections and infestations | liver abscess | 9 | 4.57(2.37, 8.8) | 4.57(2.39, 8.73) | 24.95 | 2.19(1.29) | 4.55(2.63) | male |
| renal and urinary disorders | crystalluria | 10 | 35.61(18.87, 67.18) | 35.59(19.01, 66.64) | 320.65 | 5.09(4.21) | 33.99(19.98) | male |
| skin and subcutaneous tissue disorders | vascular purpura | 9 | 10.75(5.57, 20.76) | 10.75(5.52, 20.93) | 78.42 | 3.41(2.51) | 10.61(6.11) | male |
| skin and subcutaneous tissue disorders | epidermal necrosis | 4 | 8.08(3.02, 21.65) | 8.08(3.03, 21.53) | 24.54 | 3(1.73) | 8(3.51) | male |
| gastrointestinal disorders | dysbiosis | 5 | 14.79(6.1, 35.84) | 14.78(6.12, 35.7) | 62.98 | 3.86(2.69) | 14.51(6.92) | male |
| injury, poisoning and procedural complications | wrong rate | 4 | 225.65(73.57, 692.08) | 225.61(73.82, 689.53) | 683.99 | 7.43(6) | 172.76(67.64) | male |
| renal and urinary disorders | kidney fibrosis | 6 | 5.61(2.51, 12.52) | 5.6(2.51, 12.51) | 22.53 | 2.48(1.4) | 5.57(2.84) | male |
| renal and urinary disorders | renal arteriosclerosis | 4 | 27.67(10.2, 75.1) | 27.67(10.18, 75.18) | 99.08 | 4.74(3.45) | 26.7(11.58) | male |
| renal and urinary disorders | renal tubular atrophy | 7 | 11.72(5.55, 24.74) | 11.72(5.56, 24.68) | 67.55 | 3.53(2.52) | 11.55(6.18) | male |
| injury, poisoning and procedural complications | chemical peritonitis | 3 | 32.35(10.18, 102.83) | 32.35(10.18, 102.82) | 87.29 | 4.96(3.5) | 31.02(11.79) | male |
| investigations | drug trough level | 6 | 488.94(174.02, 1373.78) | 488.82(172.98, 1381.32) | 1752.55 | 8.2(6.92) | 293.69(123.73) | male |
| respiratory, thoracic and mediastinal disorders | eosinophilic pneumonia acute | 6 | 12.98(5.79, 29.1) | 12.98(5.81, 28.99) | 65.17 | 3.67(2.59) | 12.77(6.5) | male |
| investigations | allergy test positive | 3 | 23.16(7.34, 73.09) | 23.15(7.28, 73.58) | 61.65 | 4.49(3.05) | 22.48(8.59) | male |
| renal and urinary disorders | nephritis | 37 | 22(15.86, 30.52) | 21.97(15.74, 30.66) | 718.98 | 4.42(3.95) | 21.36(16.24) | male |
| investigations | culture positive | 4 | 6.11(2.28, 16.35) | 6.11(2.29, 16.28) | 16.95 | 2.6(1.33) | 6.07(2.66) | male |
| investigations | blood culture positive | 10 | 5.13(2.75, 9.55) | 5.13(2.74, 9.61) | 33 | 2.35(1.49) | 5.1(3.03) | male |
| cardiac disorders | tachycardia | 130 | 3.33(2.8, 3.96) | 3.32(2.78, 3.96) | 209.71 | 1.73(1.48) | 3.31(2.86) | male |
| general disorders and administration site conditions | generalised oedema | 22 | 4.05(2.66, 6.16) | 4.05(2.68, 6.11) | 50.2 | 2.01(1.42) | 4.03(2.84) | male |
| respiratory, thoracic and mediastinal disorders | tachypnoea | 25 | 3.23(2.18, 4.78) | 3.23(2.18, 4.78) | 38.26 | 1.69(1.13) | 3.22(2.32) | male |
| ear and labyrinth disorders | deafness unilateral | 10 | 3.88(2.08, 7.22) | 3.88(2.07, 7.26) | 21.22 | 1.95(1.09) | 3.86(2.29) | male |
| infections and infestations | osteomyelitis | 30 | 3.1(2.16, 4.43) | 3.09(2.17, 4.4) | 42.34 | 1.63(1.12) | 3.08(2.28) | male |
| general disorders and administration site conditions | mucosal erosion | 7 | 15.51(7.34, 32.79) | 15.51(7.36, 32.66) | 93.03 | 3.93(2.91) | 15.21(8.13) | male |
| infections and infestations | pneumonia staphylococcal | 11 | 9.55(5.27, 17.31) | 9.54(5.3, 17.18) | 83.07 | 3.24(2.42) | 9.44(5.74) | male |
| infections and infestations | endocarditis bacterial | 7 | 14.14(6.69, 29.88) | 14.14(6.71, 29.78) | 83.86 | 3.8(2.79) | 13.89(7.43) | male |
| infections and infestations | opportunistic infection | 4 | 4.69(1.75, 12.53) | 4.69(1.76, 12.5) | 11.52 | 2.22(0.95) | 4.66(2.05) | male |
| skin and subcutaneous tissue disorders | subcutaneous emphysema | 5 | 7.89(3.27, 19.04) | 7.88(3.26, 19.04) | 29.74 | 2.97(1.8) | 7.81(3.74) | male |
| general disorders and administration site conditions | therapy non-responder | 53 | 3.02(2.3, 3.95) | 3.01(2.29, 3.96) | 71.01 | 1.59(1.2) | 3(2.4) | male |
| investigations | blood pressure systolic decreased | 10 | 4.42(2.37, 8.23) | 4.42(2.36, 8.28) | 26.3 | 2.14(1.28) | 4.4(2.61) | male |
| respiratory, thoracic and mediastinal disorders | diffuse alveolar damage | 5 | 6.35(2.63, 15.33) | 6.35(2.63, 15.34) | 22.36 | 2.66(1.5) | 6.31(3.02) | male |
| ear and labyrinth disorders | deafness neurosensory | 17 | 10.7(6.63, 17.27) | 10.69(6.68, 17.11) | 147.19 | 3.4(2.73) | 10.55(7.07) | male |
| infections and infestations | pyuria | 3 | 6.71(2.15, 20.91) | 6.71(2.15, 20.91) | 14.43 | 2.73(1.31) | 6.65(2.57) | male |
| skin and subcutaneous tissue disorders | rash macular | 39 | 3.03(2.21, 4.15) | 3.02(2.21, 4.13) | 52.62 | 1.59(1.14) | 3.02(2.32) | male |
| neoplasms benign, malignant and unspecified (incl cysts and polyps) | marrow hyperplasia | 4 | 8.7(3.25, 23.33) | 8.7(3.27, 23.18) | 26.95 | 3.11(1.83) | 8.61(3.77) | male |
| metabolism and nutrition disorders | electrolyte imbalance | 16 | 3.09(1.89, 5.04) | 3.09(1.89, 5.04) | 22.47 | 1.62(0.93) | 3.08(2.04) | male |
| eye disorders | periorbital oedema | 17 | 7.64(4.74, 12.33) | 7.64(4.77, 12.23) | 97.07 | 2.92(2.25) | 7.57(5.07) | male |
| hepatobiliary disorders | hepatorenal syndrome | 7 | 5.86(2.79, 12.33) | 5.86(2.78, 12.34) | 27.99 | 2.54(1.54) | 5.82(3.12) | male |
| skin and subcutaneous tissue disorders | skin erosion | 9 | 6.36(3.3, 12.26) | 6.36(3.33, 12.14) | 40.29 | 2.66(1.76) | 6.31(3.64) | male |
| respiratory, thoracic and mediastinal disorders | respiratory alkalosis | 4 | 4.9(1.83, 13.09) | 4.9(1.84, 13.06) | 12.32 | 2.28(1.01) | 4.87(2.14) | male |
| infections and infestations | escherichia infection | 16 | 4.41(2.7, 7.21) | 4.41(2.7, 7.2) | 41.87 | 2.13(1.44) | 4.39(2.91) | male |
| infections and infestations | endocarditis staphylococcal | 10 | 33.49(17.77, 63.14) | 33.48(17.88, 62.69) | 301.35 | 5(4.13) | 32.06(18.86) | male |
| metabolism and nutrition disorders | hypervolaemia | 9 | 4.78(2.48, 9.2) | 4.77(2.5, 9.11) | 26.69 | 2.25(1.35) | 4.75(2.74) | male |
| investigations | procalcitonin increased | 7 | 11.11(5.27, 23.44) | 11.11(5.28, 23.4) | 63.44 | 3.45(2.45) | 10.96(5.87) | male |
| respiratory, thoracic and mediastinal disorders | stridor | 6 | 4.25(1.91, 9.49) | 4.25(1.9, 9.49) | 14.83 | 2.08(1.01) | 4.23(2.16) | male |
| infections and infestations | lactobacillus infection | 4 | 39.64(14.49, 108.43) | 39.63(14.58, 107.68) | 142.91 | 5.23(3.93) | 37.65(16.22) | male |
| investigations | enterobacter test positive | 3 | 22.68(7.19, 71.56) | 22.68(7.14, 72.09) | 60.3 | 4.46(3.02) | 22.03(8.42) | male |
| ear and labyrinth disorders | vestibular disorder | 19 | 33.51(21.16, 53.09) | 33.49(21.34, 52.56) | 572.71 | 5(4.36) | 32.07(21.82) | male |
| skin and subcutaneous tissue disorders | livedo reticularis | 6 | 6.05(2.71, 13.52) | 6.05(2.71, 13.51) | 25.09 | 2.59(1.51) | 6.01(3.07) | male |
| renal and urinary disorders | leukocyturia | 3 | 8(2.56, 24.96) | 8(2.57, 24.93) | 18.17 | 2.99(1.56) | 7.92(3.06) | male |
| immune system disorders | serum sickness-like reaction | 3 | 14.01(4.47, 43.92) | 14.01(4.5, 43.67) | 35.57 | 3.78(2.35) | 13.77(5.29) | male |
| investigations | pseudomonas test positive | 4 | 8.53(3.18, 22.85) | 8.53(3.2, 22.73) | 26.27 | 3.08(1.8) | 8.44(3.7) | male |
| infections and infestations | fungal endocarditis | 11 | 61.13(33.04, 113.09) | 61.1(33.28, 112.18) | 600.29 | 5.82(4.97) | 56.48(33.75) | male |
| infections and infestations | endocarditis candida | 7 | 138.76(61.85, 311.28) | 138.72(62.11, 309.84) | 804.82 | 6.87(5.78) | 116.81(59.41) | male |
| renal and urinary disorders | nephritis allergic | 5 | 19.4(7.98, 47.16) | 19.4(8.03, 46.87) | 85 | 4.24(3.07) | 18.92(9) | male |
| skin and subcutaneous tissue disorders | papule | 10 | 3.47(1.87, 6.46) | 3.47(1.85, 6.5) | 17.52 | 1.79(0.94) | 3.46(2.06) | male |
| investigations | candida test positive | 3 | 14.47(4.62, 45.38) | 14.47(4.64, 45.1) | 36.89 | 3.83(2.4) | 14.21(5.46) | male |
| investigations | staphylococcus test positive | 6 | 4.83(2.16, 10.78) | 4.83(2.16, 10.79) | 18.1 | 2.26(1.19) | 4.8(2.45) | male |
| infections and infestations | trichosporon infection | 7 | 23.77(11.2, 50.46) | 23.76(11.28, 50.04) | 147.84 | 4.53(3.51) | 23.05(12.28) | male |
| infections and infestations | fungaemia | 9 | 10.53(5.45, 20.33) | 10.52(5.4, 20.48) | 76.48 | 3.38(2.48) | 10.39(5.99) | male |
| gastrointestinal disorders | peritoneal cloudy effluent | 5 | 6.34(2.63, 15.3) | 6.34(2.62, 15.32) | 22.31 | 2.65(1.49) | 6.3(3.01) | male |
| infections and infestations | ependymitis | 3 | 439.99(105.14, 1841.23) | 439.94(105.2, 1839.85) | 821.13 | 8.11(6.41) | 275.33(83.12) | male |
| vascular disorders | vasculitis necrotising | 5 | 12.39(5.12, 29.99) | 12.39(5.13, 29.93) | 51.46 | 3.61(2.44) | 12.2(5.82) | male |
| infections and infestations | infective thrombosis | 4 | 77.19(27.55, 216.31) | 77.18(27.31, 218.1) | 272.13 | 6.13(4.79) | 69.93(29.53) | male |
| injury, poisoning and procedural complications | traumatic lung injury | 6 | 6.22(2.78, 13.88) | 6.21(2.78, 13.87) | 26.03 | 2.63(1.55) | 6.17(3.15) | male |
| immune system disorders | cross sensitivity reaction | 5 | 20.72(8.52, 50.39) | 20.71(8.57, 50.03) | 91.23 | 4.33(3.16) | 20.17(9.59) | male |
| skin and subcutaneous tissue disorders | skin oedema | 5 | 12.02(4.97, 29.09) | 12.02(4.98, 29.04) | 49.7 | 3.57(2.4) | 11.84(5.65) | male |
| respiratory, thoracic and mediastinal disorders | hypercapnia | 9 | 5.55(2.88, 10.7) | 5.55(2.91, 10.6) | 33.32 | 2.46(1.57) | 5.52(3.19) | male |
| infections and infestations | cns ventriculitis | 8 | 43.79(21.45, 89.37) | 43.77(21.61, 88.64) | 315.54 | 5.37(4.4) | 41.36(22.77) | male |
| investigations | urinary casts | 6 | 26.83(11.88, 60.6) | 26.83(11.78, 61.11) | 143.91 | 4.7(3.61) | 25.91(13.11) | male |
| infections and infestations | meningitis enterococcal | 3 | 53.66(16.61, 173.29) | 53.65(16.55, 173.9) | 144.44 | 5.65(4.17) | 50.06(18.77) | male |
| infections and infestations | tetanus | 7 | 98.73(44.84, 217.37) | 98.7(45.06, 216.17) | 596.68 | 6.44(5.38) | 87.11(45.01) | male |
| investigations | acinetobacter test positive | 4 | 33.72(12.37, 91.87) | 33.71(12.41, 91.6) | 121.38 | 5.01(3.71) | 32.27(13.95) | male |
| infections and infestations | mediastinitis | 3 | 7.43(2.38, 23.18) | 7.43(2.38, 23.16) | 16.53 | 2.88(1.46) | 7.37(2.84) | male |
| investigations | inflammatory marker increased | 21 | 10.5(6.82, 16.15) | 10.49(6.82, 16.15) | 177.74 | 3.37(2.76) | 10.36(7.22) | male |
| infections and infestations | pneumonia necrotising | 4 | 10.37(3.86, 27.81) | 10.36(3.89, 27.6) | 33.37 | 3.36(2.08) | 10.23(4.48) | male |
| infections and infestations | human herpesvirus 6 infection | 17 | 19.7(12.17, 31.9) | 19.69(12.06, 32.14) | 293.74 | 4.26(3.59) | 19.2(12.83) | male |
| nervous system disorders | meningitis eosinophilic | 9 | 600.13(248.67, 1448.38) | 599.91(248.34, 1449.22) | 2959.68 | 8.37(7.28) | 330.4(158.08) | male |
| infections and infestations | device related sepsis | 7 | 6.62(3.15, 13.94) | 6.62(3.14, 13.94) | 33.12 | 2.72(1.71) | 6.57(3.53) | male |
| general disorders and administration site conditions | mucosal ulceration | 3 | 5.95(1.91, 18.52) | 5.95(1.91, 18.54) | 12.24 | 2.56(1.14) | 5.91(2.28) | male |
| skin and subcutaneous tissue disorders | neutrophilic dermatosis | 4 | 21.89(8.1, 59.18) | 21.89(8.06, 59.48) | 77.42 | 4.41(3.12) | 21.28(9.26) | male |
| investigations | stenotrophomonas test positive | 4 | 40.74(14.88, 111.52) | 40.73(14.99, 110.67) | 146.88 | 5.27(3.97) | 38.64(16.64) | male |
| infections and infestations | clostridium bacteraemia | 6 | 67.7(29.33, 156.25) | 67.68(29.14, 157.21) | 360.87 | 5.96(4.84) | 62.05(30.82) | male |
| nervous system disorders | haemorrhagic cerebral infarction | 3 | 6.73(2.16, 20.97) | 6.73(2.16, 20.98) | 14.49 | 2.74(1.32) | 6.67(2.58) | male |
| infections and infestations | fusarium infection | 4 | 11.03(4.11, 29.6) | 11.03(4.14, 29.39) | 35.93 | 3.44(2.17) | 10.88(4.76) | male |
| injury, poisoning and procedural complications | documented hypersensitivity to administered product | 7 | 27.31(12.84, 58.08) | 27.3(12.96, 57.49) | 171 | 4.72(3.7) | 26.36(14.02) | male |
| blood and lymphatic system disorders | febrile bone marrow aplasia | 9 | 4.07(2.11, 7.84) | 4.07(2.13, 7.77) | 20.72 | 2.02(1.12) | 4.05(2.34) | male |
| injury, poisoning and procedural complications | drug monitoring procedure not performed | 5 | 10.51(4.35, 25.4) | 10.5(4.35, 25.37) | 42.39 | 3.37(2.21) | 10.37(4.95) | male |
| hepatobiliary disorders | ischaemic hepatitis | 4 | 5.29(1.98, 14.13) | 5.28(1.98, 14.07) | 13.8 | 2.39(1.12) | 5.25(2.31) | male |
| infections and infestations | aspergillus infection | 21 | 6.07(3.95, 9.33) | 6.07(3.94, 9.34) | 88.15 | 2.59(1.99) | 6.03(4.21) | male |
| investigations | mast cell degranulation present | 4 | 488.9(137.95, 1732.62) | 488.82(136.73, 1747.59) | 1168.37 | 8.2(6.67) | 293.69(101.89) | male |
| blood and lymphatic system disorders | autoimmune haemolytic anaemia | 14 | 8.81(5.2, 14.92) | 8.8(5.18, 14.94) | 95.7 | 3.12(2.39) | 8.71(5.6) | male |
| skin and subcutaneous tissue disorders | palpable purpura | 7 | 47.1(21.93, 101.15) | 47.09(21.93, 101.14) | 296.71 | 5.47(4.44) | 44.31(23.37) | male |
| infections and infestations | arthritis bacterial | 9 | 4.15(2.16, 7.99) | 4.15(2.17, 7.92) | 21.4 | 2.05(1.15) | 4.13(2.39) | male |
| infections and infestations | corynebacterium infection | 4 | 12.7(4.73, 34.12) | 12.7(4.77, 33.84) | 42.37 | 3.64(2.37) | 12.5(5.47) | male |
| metabolism and nutrition disorders | vitamin k deficiency | 7 | 33.78(15.83, 72.06) | 33.77(15.72, 72.53) | 212.78 | 5.01(3.99) | 32.32(17.15) | male |
| vascular disorders | distributive shock | 6 | 7.23(3.23, 16.15) | 7.22(3.23, 16.13) | 31.86 | 2.84(1.77) | 7.16(3.65) | male |
| infections and infestations | muscle abscess | 3 | 7.61(2.44, 23.74) | 7.61(2.44, 23.72) | 17.05 | 2.92(1.49) | 7.54(2.91) | male |
| infections and infestations | infective aneurysm | 4 | 11.28(4.2, 30.29) | 11.28(4.23, 30.06) | 36.91 | 3.48(2.2) | 11.12(4.87) | male |
| vascular disorders | haemorrhagic infarction | 3 | 8.18(2.62, 25.52) | 8.18(2.62, 25.5) | 18.69 | 3.02(1.59) | 8.1(3.13) | male |
| infections and infestations | meningitis candida | 4 | 146.67(50.13, 429.14) | 146.65(49.9, 430.97) | 482.17 | 6.94(5.55) | 122.37(49.84) | male |
| hepatobiliary disorders | hepatotoxicity | 37 | 4.12(2.98, 5.69) | 4.11(3, 5.62) | 86.77 | 2.03(1.57) | 4.1(3.13) | male |
| investigations | nutritional condition abnormal | 5 | 16.44(6.78, 39.9) | 16.44(6.81, 39.71) | 70.92 | 4.01(2.84) | 16.1(7.67) | male |
| cardiac disorders | pulmonary valve disease | 3 | 32.35(10.18, 102.83) | 32.35(10.18, 102.82) | 87.29 | 4.96(3.5) | 31.02(11.79) | male |
| infections and infestations | geotrichum infection | 6 | 46.32(20.3, 105.71) | 46.31(20.33, 105.48) | 250.18 | 5.45(4.34) | 43.62(21.87) | male |
| cardiac disorders | hypersensitivity myocarditis | 3 | 56.41(17.43, 182.55) | 56.4(17.4, 182.81) | 151.6 | 5.71(4.23) | 52.44(19.63) | male |
| cardiac disorders | eosinophilic myocarditis | 8 | 25.4(12.55, 51.41) | 25.39(12.54, 51.42) | 181.19 | 4.62(3.66) | 24.58(13.63) | male |
| renal and urinary disorders | renal tubular injury | 21 | 35.59(22.96, 55.16) | 35.56(23.1, 54.73) | 672.75 | 5.09(4.47) | 33.96(23.54) | male |
| respiratory, thoracic and mediastinal disorders | alveolar lung disease | 3 | 25.58(8.09, 80.89) | 25.58(8.05, 81.31) | 68.46 | 4.63(3.18) | 24.75(9.45) | male |
| respiratory, thoracic and mediastinal disorders | pulmonary interstitial emphysema syndrome | 3 | 28.2(8.9, 89.36) | 28.2(8.87, 89.63) | 75.79 | 4.77(3.32) | 27.19(10.36) | male |
| respiratory, thoracic and mediastinal disorders | respiratory gas exchange disorder | 5 | 25.29(10.37, 61.68) | 25.28(10.46, 61.07) | 112.73 | 4.61(3.44) | 24.47(11.61) | male |
| metabolism and nutrition disorders | hyponatraemic syndrome | 3 | 27.5(8.68, 87.08) | 27.5(8.65, 87.41) | 73.83 | 4.73(3.28) | 26.54(10.12) | male |
| eye disorders | eye oedema | 6 | 6.33(2.83, 14.14) | 6.33(2.83, 14.14) | 26.7 | 2.65(1.58) | 6.28(3.21) | male |
| infections and infestations | wound infection bacterial | 4 | 20.51(7.59, 55.41) | 20.51(7.55, 55.73) | 72.21 | 4.32(3.03) | 19.98(8.7) | male |
| infections and infestations | parvovirus b19 infection | 4 | 9.52(3.55, 25.54) | 9.52(3.57, 25.37) | 30.12 | 3.23(1.96) | 9.41(4.12) | male |
| immune system disorders | immune-mediated adverse reaction | 4 | 6.48(2.42, 17.33) | 6.47(2.43, 17.24) | 18.35 | 2.68(1.41) | 6.43(2.82) | male |
| immune system disorders | haemophagocytic lymphohistiocytosis | 32 | 9.45(6.66, 13.39) | 9.43(6.63, 13.42) | 238.26 | 3.22(2.73) | 9.33(6.97) | male |
| general disorders and administration site conditions | catheter site extravasation | 3 | 18.33(5.83, 57.65) | 18.33(5.88, 57.13) | 47.96 | 4.16(2.73) | 17.91(6.87) | male |
| blood and lymphatic system disorders | bandaemia | 4 | 56.41(20.4, 155.98) | 56.4(20.35, 156.28) | 202.13 | 5.71(4.39) | 52.44(22.39) | male |
| neoplasms benign, malignant and unspecified (incl cysts and polyps) | metastases to bone marrow | 3 | 12.36(3.95, 38.69) | 12.36(3.97, 38.52) | 30.8 | 3.61(2.18) | 12.17(4.68) | male |
| psychiatric disorders | tangentiality | 3 | 23.66(7.49, 74.69) | 23.65(7.44, 75.17) | 63.05 | 4.52(3.08) | 22.94(8.77) | male |
| vascular disorders | phlebitis superficial | 3 | 16.67(5.31, 52.34) | 16.66(5.35, 51.93) | 43.19 | 4.03(2.59) | 16.32(6.26) | male |
| musculoskeletal and connective tissue disorders | soft tissue necrosis | 4 | 12.7(4.73, 34.12) | 12.7(4.77, 33.84) | 42.37 | 3.64(2.37) | 12.5(5.47) | male |
| infections and infestations | haemorrhagic pneumonia | 3 | 73.33(22.38, 240.3) | 73.32(22.18, 242.36) | 194.55 | 6.06(4.57) | 66.75(24.72) | male |
| investigations | lymphocyte morphology abnormal | 7 | 21.13(9.96, 44.79) | 21.12(10.03, 44.48) | 130.43 | 4.36(3.35) | 20.56(10.96) | male |
| infections and infestations | disseminated cryptococcosis | 3 | 5.91(1.9, 18.42) | 5.91(1.9, 18.42) | 12.15 | 2.55(1.13) | 5.87(2.27) | male |
| infections and infestations | escherichia bacteraemia | 5 | 4.28(1.78, 10.32) | 4.28(1.77, 10.34) | 12.51 | 2.09(0.93) | 4.26(2.04) | male |
| gastrointestinal disorders | angular cheilitis | 3 | 13.66(4.36, 42.82) | 13.66(4.38, 42.58) | 34.56 | 3.75(2.32) | 13.43(5.16) | male |
| general disorders and administration site conditions | therapeutic product effect increased | 3 | 5.91(1.9, 18.42) | 5.91(1.9, 18.42) | 12.15 | 2.55(1.13) | 5.87(2.27) | male |
| skin and subcutaneous tissue disorders | fixed eruption | 10 | 10.29(5.51, 19.21) | 10.28(5.49, 19.25) | 82.65 | 3.34(2.48) | 10.16(6.02) | male |
| skin and subcutaneous tissue disorders | target skin lesion | 3 | 115.79(34.26, 391.3) | 115.77(34.34, 390.26) | 294.8 | 6.65(5.11) | 100.12(36.14) | male |
| investigations | drug level abnormal | 4 | 17.36(6.44, 46.79) | 17.35(6.39, 47.14) | 60.22 | 4.09(2.8) | 16.98(7.4) | male |
| cardiac disorders | arteriospasm coronary | 10 | 4.68(2.51, 8.71) | 4.68(2.5, 8.76) | 28.72 | 2.22(1.36) | 4.65(2.77) | male |
| investigations | blood electrolytes abnormal | 5 | 9.01(3.73, 21.76) | 9.01(3.73, 21.77) | 35.16 | 3.16(1.99) | 8.91(4.26) | male |
| infections and infestations | toxic shock syndrome | 5 | 20.95(8.61, 50.98) | 20.95(8.67, 50.61) | 92.35 | 4.35(3.18) | 20.4(9.69) | male |
| infections and infestations | lymphangitis | 8 | 20.66(10.23, 41.72) | 20.65(10.2, 41.82) | 145.52 | 4.33(3.37) | 20.12(11.17) | male |
| infections and infestations | fungal peritonitis | 14 | 13.3(7.84, 22.57) | 13.3(7.83, 22.58) | 156.38 | 3.71(2.97) | 13.08(8.4) | male |
| congenital, familial and genetic disorders | epidermolysis bullosa | 3 | 20.18(6.41, 63.56) | 20.18(6.35, 64.14) | 53.23 | 4.3(2.86) | 19.67(7.53) | male |
| renal and urinary disorders | kidney congestion | 3 | 27.16(8.58, 85.99) | 27.16(8.54, 86.33) | 72.88 | 4.71(3.27) | 26.22(10) | male |
| nervous system disorders | cerebral cyst | 3 | 9.02(2.89, 28.15) | 9.02(2.89, 28.11) | 21.12 | 3.16(1.73) | 8.92(3.44) | male |
| infections and infestations | urinary tract infection fungal | 5 | 21.07(8.66, 51.27) | 21.07(8.72, 50.9) | 92.92 | 4.36(3.18) | 20.51(9.75) | male |
| investigations | antinuclear antibody negative | 3 | 314.28(81.26, 1215.45) | 314.24(81.27, 1215.07) | 655.71 | 7.78(6.13) | 220.27(71.03) | male |
| investigations | interleukin-2 receptor increased | 3 | 35.48(11.14, 113.04) | 35.48(11.16, 112.77) | 95.88 | 5.08(3.63) | 33.89(12.85) | male |
| investigations | red blood cell sedimentation rate decreased | 3 | 31.88(10.03, 101.3) | 31.88(10.03, 101.33) | 85.99 | 4.94(3.48) | 30.59(11.63) | male |
| investigations | rheumatoid factor negative | 3 | 27.5(8.68, 87.08) | 27.5(8.65, 87.41) | 73.83 | 4.73(3.28) | 26.54(10.12) | male |
| musculoskeletal and connective tissue disorders | still's disease | 3 | 9.95(3.19, 31.1) | 9.95(3.19, 31.01) | 23.84 | 3.3(1.87) | 9.83(3.79) | male |
| infections and infestations | viral pharyngitis | 3 | 20.56(6.53, 64.77) | 20.56(6.47, 65.35) | 54.3 | 4.32(2.88) | 20.02(7.67) | male |
| infections and infestations | dermo-hypodermitis | 4 | 13.33(4.96, 35.85) | 13.33(5, 35.52) | 44.81 | 3.71(2.43) | 13.11(5.73) | male |
| gastrointestinal disorders | peritoneal disorder | 3 | 8.7(2.79, 27.14) | 8.69(2.79, 27.08) | 20.19 | 3.11(1.68) | 8.6(3.32) | male |
| metabolism and nutrition disorders | cell death | 8 | 7.41(3.69, 14.87) | 7.41(3.66, 15.01) | 43.89 | 2.88(1.93) | 7.34(4.1) | male |
| nervous system disorders | encephalomalacia | 4 | 12.48(4.65, 33.54) | 12.48(4.68, 33.25) | 41.54 | 3.62(2.34) | 12.29(5.37) | male |
| investigations | procalcitonin abnormal | 3 | 55(17.01, 177.8) | 54.99(16.96, 178.24) | 147.94 | 5.68(4.2) | 51.23(19.19) | male |
| hepatobiliary disorders | cholestatic liver injury | 8 | 7.97(3.97, 16) | 7.97(3.94, 16.14) | 48.24 | 2.98(2.03) | 7.89(4.41) | male |
| investigations | antimicrobial susceptibility test resistant | 6 | 34.11(15.04, 77.35) | 34.1(14.97, 77.67) | 184.23 | 5.03(3.93) | 32.63(16.45) | male |
| metabolism and nutrition disorders | calciphylaxis | 4 | 6.74(2.52, 18.05) | 6.74(2.53, 17.96) | 19.38 | 2.74(1.47) | 6.69(2.94) | male |
| skin and subcutaneous tissue disorders | cutaneous calcification | 3 | 68.75(21.05, 224.52) | 68.74(21.21, 222.81) | 183.1 | 5.98(4.49) | 62.93(23.38) | male |
| skin and subcutaneous tissue disorders | sjs-ten overlap | 4 | 19.95(7.39, 53.88) | 19.95(7.34, 54.21) | 70.1 | 4.28(3) | 19.45(8.47) | male |
| vascular disorders | arteritis | 4 | 12.92(4.81, 34.73) | 12.92(4.85, 34.42) | 43.23 | 3.67(2.39) | 12.71(5.56) | male |
| hepatobiliary disorders | neonatal cholestasis | 4 | 86.28(30.61, 243.15) | 86.26(30.53, 243.76) | 301.61 | 6.27(4.93) | 77.29(32.48) | male |
| nervous system disorders | pleocytosis | 4 | 13.9(5.17, 37.39) | 13.9(5.22, 37.04) | 47 | 3.77(2.49) | 13.66(5.97) | male |
| vascular disorders | superficial vein thrombosis | 9 | 28.33(14.56, 55.14) | 28.32(14.54, 55.15) | 228.4 | 4.77(3.86) | 27.31(15.64) | male |
| infections and infestations | gastroenteritis staphylococcal | 3 | 27.16(8.58, 85.99) | 27.16(8.54, 86.33) | 72.88 | 4.71(3.27) | 26.22(10) | male |
| general disorders and administration site conditions | pneumatosis | 3 | 12.5(3.99, 39.13) | 12.5(4.01, 38.96) | 31.2 | 3.62(2.19) | 12.31(4.74) | male |
| eye disorders | choroidal detachment | 3 | 9.4(3.01, 29.36) | 9.4(3.02, 29.3) | 22.24 | 3.22(1.79) | 9.29(3.58) | male |
| blood and lymphatic system disorders | myeloid maturation arrest | 10 | 236.62(116, 482.69) | 236.52(116.8, 478.97) | 1773.27 | 7.48(6.53) | 179.08(98.62) | male |
| neoplasms benign, malignant and unspecified (incl cysts and polyps) | blast cell proliferation | 3 | 88(26.57, 291.48) | 87.99(26.62, 290.85) | 230.35 | 6.3(4.79) | 78.67(28.88) | male |
| infections and infestations | klebsiella bacteraemia | 3 | 6.18(1.98, 19.25) | 6.18(1.98, 19.26) | 12.91 | 2.62(1.2) | 6.14(2.37) | male |
| immune system disorders | jarisch-herxheimer reaction | 8 | 16.44(8.16, 33.12) | 16.43(8.11, 33.27) | 113.39 | 4.01(3.05) | 16.09(8.95) | male |
| injury, poisoning and procedural complications | drug dispensed to wrong patient | 3 | 9.73(3.12, 30.41) | 9.73(3.12, 30.33) | 23.2 | 3.27(1.84) | 9.62(3.71) | male |
| investigations | protein urine | 5 | 10.19(4.21, 24.62) | 10.18(4.21, 24.59) | 40.84 | 3.33(2.17) | 10.06(4.81) | male |
| investigations | double stranded dna antibody positive | 5 | 57.29(23.06, 142.37) | 57.28(23.25, 141.11) | 256.47 | 5.73(4.53) | 53.2(24.84) | male |
| infections and infestations | cutibacterium acnes infection | 4 | 127.54(44.1, 368.82) | 127.52(44.25, 367.48) | 427.72 | 6.77(5.39) | 108.77(44.74) | male |
| nervous system disorders | neurodevelopmental delay | 3 | 314.28(81.26, 1215.45) | 314.24(81.27, 1215.07) | 655.71 | 7.78(6.13) | 220.27(71.03) | male |
| infections and infestations | urinary tract candidiasis | 3 | 43.14(13.46, 138.22) | 43.13(13.57, 137.09) | 116.6 | 5.35(3.89) | 40.79(15.4) | male |
| injury, poisoning and procedural complications | sternal fracture | 5 | 11.18(4.62, 27.04) | 11.18(4.63, 27.01) | 45.64 | 3.46(2.3) | 11.02(5.26) | male |
| immune system disorders | multiple drug hypersensitivity | 3 | 314.28(81.26, 1215.45) | 314.24(81.27, 1215.07) | 655.71 | 7.78(6.13) | 220.27(71.03) | male |
| renal and urinary disorders | acute kidney injury | 464 | 14.36(13.06, 15.79) | 13.58(12.31, 14.98) | 5320.12 | 3.74(3.6) | 13.32(12.31) | unknown |
| immune system disorders | drug hypersensitivity | 157 | 4.98(4.25, 5.83) | 4.9(4.19, 5.73) | 485.33 | 2.28(2.06) | 4.87(4.26) | unknown |
| skin and subcutaneous tissue disorders | drug reaction with eosinophilia and systemic symptoms | 199 | 51.74(44.71, 59.87) | 50.47(44, 57.89) | 8956.46 | 5.55(5.34) | 46.89(41.5) | unknown |
| skin and subcutaneous tissue disorders | linear iga disease | 86 | 618.59(460.29, 831.34) | 611.91(456.04, 821.05) | 26967.9 | 8.3(7.93) | 315.08(246.04) | unknown |
| renal and urinary disorders | nephropathy toxic | 302 | 92.19(81.57, 104.18) | 88.72(78.88, 99.79) | 23047.7 | 6.29(6.11) | 78.15(70.55) | unknown |
| investigations | blood creatinine increased | 80 | 9(7.21, 11.24) | 8.92(7.19, 11.07) | 555.8 | 3.14(2.82) | 8.82(7.32) | unknown |
| blood and lymphatic system disorders | thrombocytopenia | 82 | 3.31(2.66, 4.11) | 3.28(2.64, 4.07) | 129.94 | 1.71(1.4) | 3.27(2.73) | unknown |
| renal and urinary disorders | renal tubular necrosis | 47 | 47.16(35.05, 63.47) | 46.89(34.95, 62.92) | 1968.57 | 5.45(5.03) | 43.79(34.16) | unknown |
| investigations | drug level increased | 94 | 34.67(28.14, 42.71) | 34.27(27.62, 42.52) | 2884.55 | 5.03(4.73) | 32.6(27.38) | unknown |
| immune system disorders | anaphylactic reaction | 42 | 4.24(3.13, 5.75) | 4.22(3.15, 5.66) | 102.84 | 2.07(1.64) | 4.2(3.26) | unknown |
| renal and urinary disorders | renal impairment | 65 | 4.56(3.57, 5.83) | 4.53(3.58, 5.73) | 177.93 | 2.17(1.82) | 4.51(3.67) | unknown |
| blood and lymphatic system disorders | eosinophilia | 30 | 14.46(10.06, 20.78) | 14.41(10.13, 20.51) | 366.33 | 3.82(3.3) | 14.12(10.42) | unknown |
| general disorders and administration site conditions | treatment failure | 273 | 15.75(13.94, 17.8) | 15.24(13.55, 17.14) | 3558.34 | 3.9(3.72) | 14.92(13.47) | unknown |
| blood and lymphatic system disorders | leukopenia | 39 | 4.27(3.11, 5.85) | 4.25(3.11, 5.82) | 96.55 | 2.08(1.63) | 4.23(3.25) | unknown |
| renal and urinary disorders | tubulointerstitial nephritis | 32 | 6.19(4.37, 8.78) | 6.17(4.34, 8.78) | 137.47 | 2.61(2.12) | 6.12(4.57) | unknown |
| infections and infestations | clostridium difficile infection | 62 | 19.77(15.34, 25.47) | 19.62(15.21, 25.31) | 1063.86 | 4.25(3.89) | 19.07(15.43) | unknown |
| skin and subcutaneous tissue disorders | rash maculo-papular | 22 | 9.11(5.98, 13.89) | 9.09(6.02, 13.72) | 156.26 | 3.17(2.57) | 8.98(6.31) | unknown |
| skin and subcutaneous tissue disorders | rash erythematous | 30 | 7.68(5.35, 11.01) | 7.65(5.38, 10.89) | 171.58 | 2.92(2.41) | 7.58(5.6) | unknown |
| infections and infestations | septic shock | 37 | 6.18(4.47, 8.55) | 6.16(4.5, 8.43) | 158.38 | 2.61(2.15) | 6.11(4.65) | unknown |
| general disorders and administration site conditions | multiple organ dysfunction syndrome | 29 | 6.12(4.24, 8.83) | 6.1(4.2, 8.85) | 122.7 | 2.6(2.08) | 6.06(4.46) | unknown |
| skin and subcutaneous tissue disorders | toxic epidermal necrolysis | 21 | 8.78(5.71, 13.52) | 8.76(5.69, 13.48) | 142.5 | 3.11(2.51) | 8.66(6.04) | unknown |
| skin and subcutaneous tissue disorders | drug eruption | 55 | 25.18(19.21, 32.99) | 25.01(19.01, 32.91) | 1220.86 | 4.59(4.21) | 24.12(19.23) | unknown |
| skin and subcutaneous tissue disorders | acute generalised exanthematous pustulosis | 19 | 19.06(12.07, 30.09) | 19.01(12.11, 29.84) | 315.06 | 4.21(3.57) | 18.5(12.62) | unknown |
| blood and lymphatic system disorders | pancytopenia | 24 | 3.49(2.33, 5.21) | 3.48(2.35, 5.15) | 42.22 | 1.79(1.23) | 3.47(2.48) | unknown |
| general disorders and administration site conditions | drug resistance | 24 | 3.04(2.04, 4.54) | 3.04(2.05, 4.5) | 32.63 | 1.6(1.03) | 3.03(2.16) | unknown |
| infections and infestations | staphylococcal infection | 26 | 8.93(6.06, 13.16) | 8.91(6.02, 13.19) | 180.1 | 3.14(2.59) | 8.8(6.36) | unknown |
| eye disorders | retinal vasculitis | 65 | 279.16(208.61, 373.57) | 276.88(206.35, 371.51) | 12516.18 | 7.6(7.2) | 194.25(152.23) | unknown |
| hepatobiliary disorders | drug-induced liver injury | 27 | 4.02(2.75, 5.87) | 4.01(2.76, 5.82) | 60.69 | 2(1.46) | 3.99(2.91) | unknown |
| skin and subcutaneous tissue disorders | stevens-johnson syndrome | 17 | 4.87(3.02, 7.85) | 4.86(3.04, 7.78) | 51.71 | 2.27(1.6) | 4.83(3.24) | unknown |
| infections and infestations | pathogen resistance | 30 | 7.25(5.05, 10.39) | 7.22(5.07, 10.27) | 159.17 | 2.84(2.33) | 7.16(5.29) | unknown |
| skin and subcutaneous tissue disorders | vancomycin infusion reaction | 33 | 932.87(547.53, 1589.4) | 929(547.25, 1577.05) | 12564.3 | 8.58(7.96) | 382.14(244.68) | unknown |
| investigations | antibiotic level above therapeutic | 15 | 374.25(198.16, 706.85) | 373.55(199.51, 699.42) | 3534.3 | 7.89(7.06) | 237.25(139.36) | unknown |
| infections and infestations | enterococcal infection | 24 | 41.9(27.71, 63.34) | 41.77(27.68, 63.04) | 897.31 | 5.3(4.71) | 39.3(27.81) | unknown |
| skin and subcutaneous tissue disorders | toxic skin eruption | 7 | 7.22(3.43, 15.22) | 7.22(3.43, 15.21) | 37.08 | 2.84(1.83) | 7.15(3.83) | unknown |
| respiratory, thoracic and mediastinal disorders | hypoxia | 13 | 3.25(1.88, 5.61) | 3.25(1.88, 5.63) | 20.13 | 1.69(0.94) | 3.24(2.05) | unknown |
| general disorders and administration site conditions | face oedema | 13 | 8.08(4.67, 13.97) | 8.07(4.66, 13.97) | 79.55 | 3(2.24) | 7.98(5.05) | unknown |
| blood and lymphatic system disorders | leukocytosis | 16 | 8.17(4.99, 13.39) | 8.16(5, 13.32) | 99.25 | 3.01(2.32) | 8.07(5.34) | unknown |
| vascular disorders | shock | 13 | 4.17(2.41, 7.19) | 4.16(2.4, 7.2) | 31.05 | 2.05(1.29) | 4.14(2.62) | unknown |
| skin and subcutaneous tissue disorders | erythema multiforme | 8 | 7.42(3.69, 14.89) | 7.41(3.66, 15.01) | 43.87 | 2.88(1.93) | 7.34(4.09) | unknown |
| infections and infestations | candida infection | 12 | 5.38(3.05, 9.5) | 5.37(3.04, 9.48) | 42.37 | 2.42(1.63) | 5.34(3.32) | unknown |
| skin and subcutaneous tissue disorders | rash morbilliform | 10 | 37.69(19.91, 71.33) | 37.64(19.71, 71.87) | 337.12 | 5.16(4.28) | 35.63(20.89) | unknown |
| investigations | blood urea increased | 10 | 7.04(3.77, 13.13) | 7.03(3.75, 13.16) | 51.18 | 2.8(1.94) | 6.97(4.13) | unknown |
| infections and infestations | clostridium difficile colitis | 11 | 6.91(3.81, 12.52) | 6.9(3.83, 12.42) | 54.94 | 2.77(1.95) | 6.84(4.16) | unknown |
| investigations | drug level above therapeutic | 9 | 6.04(3.13, 11.64) | 6.03(3.16, 11.51) | 37.44 | 2.58(1.68) | 5.99(3.46) | unknown |
| vascular disorders | haemodynamic instability | 12 | 11.48(6.48, 20.32) | 11.46(6.49, 20.23) | 112.59 | 3.5(2.7) | 11.28(6.99) | unknown |
| renal and urinary disorders | oliguria | 10 | 13.02(6.96, 24.35) | 13(6.94, 24.34) | 108.62 | 3.67(2.81) | 12.77(7.56) | unknown |
| cardiac disorders | kounis syndrome | 10 | 45.98(24.2, 87.35) | 45.92(24.05, 87.68) | 410.34 | 5.42(4.54) | 42.95(25.1) | unknown |
| injury, poisoning and procedural complications | incorrect drug administration rate | 20 | 25.26(16.15, 39.5) | 25.19(16.05, 39.54) | 447.32 | 4.6(3.97) | 24.29(16.71) | unknown |
| blood and lymphatic system disorders | immune thrombocytopenia | 8 | 8.63(4.29, 17.34) | 8.62(4.26, 17.46) | 53.18 | 3.09(2.14) | 8.52(4.75) | unknown |
| vascular disorders | circulatory collapse | 7 | 3.75(1.78, 7.88) | 3.75(1.78, 7.9) | 14.01 | 1.9(0.9) | 3.73(2) | unknown |
| renal and urinary disorders | anuria | 8 | 7.6(3.78, 15.27) | 7.6(3.75, 15.39) | 45.29 | 2.91(1.96) | 7.52(4.19) | unknown |
| infections and infestations | bacteraemia | 10 | 4.78(2.57, 8.91) | 4.78(2.55, 8.95) | 29.67 | 2.25(1.39) | 4.75(2.82) | unknown |
| skin and subcutaneous tissue disorders | purpura | 9 | 10.45(5.41, 20.21) | 10.44(5.36, 20.33) | 75.64 | 3.36(2.46) | 10.29(5.93) | unknown |
| infections and infestations | staphylococcal bacteraemia | 20 | 35.18(22.42, 55.2) | 35.09(22.36, 55.08) | 628.44 | 5.06(4.42) | 33.34(22.87) | unknown |
| infections and infestations | endocarditis | 14 | 19.53(11.47, 33.24) | 19.49(11.48, 33.09) | 238.48 | 4.24(3.5) | 18.95(12.14) | unknown |
| immune system disorders | type iv hypersensitivity reaction | 9 | 22.27(11.45, 43.29) | 22.24(11.42, 43.31) | 176.53 | 4.43(3.52) | 21.54(12.35) | unknown |
| eye disorders | retinal vascular occlusion | 49 | 476.47(329.41, 689.17) | 473.53(326.3, 687.19) | 13345.57 | 8.1(7.62) | 273.93(201.15) | unknown |
| infections and infestations | systemic candida | 17 | 33.32(20.46, 54.28) | 33.25(20.37, 54.27) | 505.89 | 4.99(4.3) | 31.68(21.06) | unknown |
| ear and labyrinth disorders | ototoxicity | 6 | 10.95(4.89, 24.55) | 10.94(4.9, 24.44) | 53.31 | 3.43(2.35) | 10.78(5.49) | unknown |
| eye disorders | retinal haemorrhage | 14 | 10.51(6.19, 17.83) | 10.49(6.18, 17.81) | 118.32 | 3.37(2.63) | 10.34(6.64) | unknown |
| infections and infestations | klebsiella infection | 9 | 13.53(6.99, 26.2) | 13.52(6.94, 26.33) | 102.22 | 3.73(2.82) | 13.26(7.63) | unknown |
| skin and subcutaneous tissue disorders | dermatitis exfoliative | 4 | 7.36(2.75, 19.73) | 7.36(2.76, 19.61) | 21.73 | 2.87(1.59) | 7.29(3.19) | unknown |
| skin and subcutaneous tissue disorders | henoch-schonlein purpura | 5 | 23.3(9.55, 56.89) | 23.29(9.45, 57.38) | 102.97 | 4.49(3.31) | 22.52(10.67) | unknown |
| vascular disorders | haemorrhagic vasculitis | 20 | 1622.79(714.57, 3685.37) | 1618.71(710.65, 3687.06) | 9238.4 | 8.86(8.04) | 463.2(233.19) | unknown |
| investigations | eosinophil count increased | 5 | 4.64(1.92, 11.19) | 4.64(1.92, 11.21) | 14.17 | 2.21(1.05) | 4.61(2.21) | unknown |
| skin and subcutaneous tissue disorders | hypersensitivity vasculitis | 5 | 13.01(5.37, 31.54) | 13(5.38, 31.4) | 54.31 | 3.67(2.51) | 12.77(6.08) | unknown |
| investigations | drug level below therapeutic | 18 | 7.7(4.84, 12.27) | 7.69(4.8, 12.31) | 103.52 | 2.93(2.27) | 7.61(5.16) | unknown |
| skin and subcutaneous tissue disorders | cutaneous vasculitis | 3 | 6.59(2.11, 20.54) | 6.58(2.11, 20.51) | 14.07 | 2.71(1.28) | 6.53(2.52) | unknown |
| immune system disorders | anaphylactoid reaction | 7 | 11.4(5.4, 24.07) | 11.39(5.41, 23.99) | 65.19 | 3.49(2.48) | 11.21(6) | unknown |
| renal and urinary disorders | renal tubular disorder | 19 | 45.5(28.56, 72.49) | 45.4(28.36, 72.67) | 770.93 | 5.41(4.75) | 42.49(28.78) | unknown |
| respiratory, thoracic and mediastinal disorders | pulmonary haemorrhage | 8 | 4.99(2.49, 10.01) | 4.99(2.51, 9.91) | 25.3 | 2.31(1.36) | 4.95(2.77) | unknown |
| general disorders and administration site conditions | multiple-drug resistance | 7 | 14.59(6.89, 30.86) | 14.57(6.92, 30.68) | 86.55 | 3.84(2.82) | 14.27(7.62) | unknown |
| skin and subcutaneous tissue disorders | skin plaque | 7 | 9.88(4.68, 20.85) | 9.87(4.69, 20.79) | 55 | 3.28(2.28) | 9.74(5.21) | unknown |
| cardiac disorders | pulseless electrical activity | 5 | 11.09(4.58, 26.86) | 11.09(4.59, 26.79) | 45.12 | 3.45(2.28) | 10.92(5.21) | unknown |
| infections and infestations | pseudomonas infection | 5 | 4.52(1.88, 10.9) | 4.52(1.87, 10.92) | 13.62 | 2.17(1.01) | 4.5(2.15) | unknown |
| infections and infestations | acinetobacter infection | 6 | 26.63(11.77, 60.26) | 26.61(11.68, 60.61) | 142.05 | 4.68(3.58) | 25.6(12.92) | unknown |
| gastrointestinal disorders | noninfectious peritonitis | 3 | 161.93(45.69, 573.95) | 161.87(45.28, 578.71) | 383.71 | 7.02(5.44) | 129.7(44.99) | unknown |
| investigations | antibiotic level below therapeutic | 3 | 277.6(71.77, 1073.72) | 277.49(71.76, 1072.97) | 578.54 | 7.6(5.95) | 194.55(62.73) | unknown |
| hepatobiliary disorders | hypertransaminasaemia | 4 | 4.74(1.77, 12.67) | 4.73(1.78, 12.6) | 11.7 | 2.24(0.96) | 4.71(2.07) | unknown |
| general disorders and administration site conditions | systemic inflammatory response syndrome | 4 | 6.29(2.35, 16.84) | 6.29(2.36, 16.76) | 17.61 | 2.64(1.37) | 6.24(2.73) | unknown |
| gastrointestinal disorders | megacolon | 7 | 42(19.55, 90.24) | 41.97(19.54, 90.14) | 262.9 | 5.3(4.27) | 39.47(20.82) | unknown |
| infections and infestations | stenotrophomonas infection | 3 | 15.67(4.98, 49.26) | 15.66(5.02, 48.81) | 40.21 | 3.94(2.5) | 15.32(5.87) | unknown |
| renal and urinary disorders | azotaemia | 9 | 20.47(10.54, 39.76) | 20.45(10.5, 39.82) | 161.38 | 4.31(3.4) | 19.85(11.39) | unknown |
| general disorders and administration site conditions | localised oedema | 3 | 6.46(2.07, 20.13) | 6.45(2.07, 20.1) | 13.69 | 2.68(1.25) | 6.4(2.47) | unknown |
| infections and infestations | mucormycosis | 3 | 6.99(2.24, 21.81) | 6.99(2.24, 21.79) | 15.23 | 2.79(1.37) | 6.92(2.67) | unknown |
| gastrointestinal disorders | enterocolitis haemorrhagic | 4 | 20.9(7.72, 56.57) | 20.89(7.69, 56.76) | 73.37 | 4.34(3.05) | 20.27(8.81) | unknown |
| infections and infestations | septic embolus | 8 | 78.56(37.7, 163.69) | 78.48(38, 162.07) | 545.81 | 6.13(5.13) | 70.11(37.93) | unknown |
| infections and infestations | enterobacter infection | 5 | 14.66(6.04, 35.57) | 14.65(6.06, 35.39) | 62.18 | 3.84(2.67) | 14.35(6.83) | unknown |
| infections and infestations | intervertebral discitis | 3 | 12.46(3.97, 39.05) | 12.45(3.99, 38.8) | 31 | 3.61(2.18) | 12.24(4.7) | unknown |
| investigations | clostridium test positive | 3 | 9.48(3.03, 29.64) | 9.48(3.04, 29.55) | 22.42 | 3.23(1.8) | 9.35(3.6) | unknown |
| infections and infestations | meningitis bacterial | 4 | 17.99(6.66, 48.61) | 17.99(6.62, 48.88) | 62.43 | 4.13(2.85) | 17.53(7.63) | unknown |
| blood and lymphatic system disorders | disseminated intravascular coagulation | 11 | 6.79(3.75, 12.31) | 6.78(3.77, 12.21) | 53.69 | 2.75(1.93) | 6.72(4.09) | unknown |
| skin and subcutaneous tissue disorders | blister | 21 | 3.8(2.48, 5.85) | 3.8(2.47, 5.85) | 43.05 | 1.92(1.31) | 3.78(2.64) | unknown |
| general disorders and administration site conditions | necrosis | 4 | 5.88(2.19, 15.73) | 5.87(2.2, 15.64) | 16.03 | 2.54(1.27) | 5.83(2.56) | unknown |
| infections and infestations | staphylococcal sepsis | 7 | 11.37(5.38, 24.01) | 11.36(5.39, 23.92) | 65 | 3.48(2.47) | 11.18(5.98) | unknown |
| investigations | urine output decreased | 7 | 8.86(4.2, 18.68) | 8.85(4.2, 18.64) | 48.1 | 3.13(2.12) | 8.75(4.68) | unknown |
| skin and subcutaneous tissue disorders | nikolsky's sign | 4 | 51.82(18.71, 143.53) | 51.8(18.69, 143.54) | 184.51 | 5.59(4.26) | 48.04(20.48) | unknown |
| infections and infestations | rash pustular | 6 | 8.36(3.74, 18.71) | 8.35(3.74, 18.65) | 38.35 | 3.05(1.97) | 8.26(4.21) | unknown |
| infections and infestations | leuconostoc infection | 4 | 863.75(193.28, 3859.98) | 863.31(194.64, 3829.06) | 1476.54 | 8.53(6.92) | 370.56(105.88) | unknown |
| eye disorders | retinal ischaemia | 8 | 71.03(34.22, 147.43) | 70.96(34.36, 146.54) | 497.28 | 6(5.01) | 64.05(34.76) | unknown |
| ear and labyrinth disorders | deafness bilateral | 6 | 32.95(14.5, 74.85) | 32.92(14.45, 74.98) | 176.74 | 4.97(3.87) | 31.38(15.79) | unknown |
| injury, poisoning and procedural complications | therapeutic drug monitoring analysis incorrectly performed | 3 | 102.27(30.26, 345.68) | 102.23(30.33, 344.61) | 259.72 | 6.47(4.93) | 88.43(31.92) | unknown |
| infections and infestations | septic pulmonary embolism | 4 | 431.87(121.85, 1530.73) | 431.66(120.74, 1543.24) | 1031.18 | 8.02(6.49) | 259.39(89.98) | unknown |
| respiratory, thoracic and mediastinal disorders | tachypnoea | 6 | 4.48(2.01, 10) | 4.48(2.01, 10.01) | 16.09 | 2.15(1.08) | 4.45(2.27) | unknown |
| ear and labyrinth disorders | deafness unilateral | 3 | 6.72(2.16, 20.97) | 6.72(2.16, 20.94) | 14.46 | 2.74(1.31) | 6.66(2.57) | unknown |
| infections and infestations | osteomyelitis | 13 | 9.53(5.51, 16.48) | 9.51(5.49, 16.46) | 97.59 | 3.23(2.47) | 9.39(5.93) | unknown |
| infections and infestations | pneumonia staphylococcal | 5 | 26.99(11.03, 66.06) | 26.98(10.95, 66.47) | 120.08 | 4.7(3.52) | 25.94(12.27) | unknown |
| ear and labyrinth disorders | deafness neurosensory | 3 | 5.46(1.75, 17.01) | 5.46(1.75, 17.02) | 10.83 | 2.44(1.02) | 5.42(2.09) | unknown |
| skin and subcutaneous tissue disorders | skin erosion | 6 | 14.84(6.6, 33.34) | 14.83(6.64, 33.12) | 75.64 | 3.86(2.78) | 14.52(7.37) | unknown |
| skin and subcutaneous tissue disorders | papule | 5 | 7.5(3.1, 18.11) | 7.49(3.1, 18.09) | 27.82 | 2.89(1.73) | 7.42(3.55) | unknown |
| infections and infestations | fungaemia | 7 | 29.27(13.72, 62.43) | 29.24(13.61, 62.8) | 182.68 | 4.81(3.78) | 28.02(14.86) | unknown |
| gastrointestinal disorders | peritoneal cloudy effluent | 3 | 46.27(14.34, 149.3) | 46.25(14.27, 149.91) | 123.96 | 5.43(3.96) | 43.23(16.22) | unknown |
| infections and infestations | cns ventriculitis | 5 | 59.99(23.99, 150) | 59.95(23.86, 150.61) | 265.28 | 5.78(4.57) | 54.96(25.53) | unknown |
| investigations | inflammatory marker increased | 3 | 7.04(2.26, 21.97) | 7.04(2.26, 21.94) | 15.37 | 2.8(1.38) | 6.97(2.69) | unknown |
| injury, poisoning and procedural complications | drug monitoring procedure not performed | 11 | 17.1(9.39, 31.13) | 17.08(9.3, 31.36) | 162.26 | 4.06(3.23) | 16.67(10.1) | unknown |
| infections and infestations | arthritis bacterial | 5 | 9.2(3.81, 22.25) | 9.2(3.81, 22.22) | 36.02 | 3.18(2.02) | 9.08(4.34) | unknown |
| infections and infestations | infective aneurysm | 5 | 107.98(41.89, 278.38) | 107.91(42.12, 276.47) | 453.96 | 6.53(5.29) | 92.64(41.94) | unknown |
| infections and infestations | meningitis candida | 3 | 485.8(108.71, 2170.96) | 485.61(109.49, 2153.84) | 829.06 | 8.12(6.39) | 277.92(79.41) | unknown |
| renal and urinary disorders | renal tubular injury | 5 | 49.08(19.77, 121.86) | 49.05(19.91, 120.84) | 218.79 | 5.51(4.31) | 45.67(21.34) | unknown |
| investigations | blood electrolytes abnormal | 7 | 63.89(29.38, 138.93) | 63.84(29.15, 139.82) | 394.12 | 5.86(4.81) | 58.2(30.38) | unknown |
| nervous system disorders | pleocytosis | 3 | 35.98(11.25, 115.11) | 35.97(11.32, 114.33) | 96.63 | 5.09(3.63) | 34.13(12.9) | unknown |
| general disorders and administration site conditions | pyrexia | 141 | 3.7(3.13, 4.37) | 3.65(3.12, 4.27) | 271.02 | 1.86(1.62) | 3.63(3.16) | unknown |
| general disorders and administration site conditions | drug interaction | 90 | 3.73(3.02, 4.59) | 3.69(2.97, 4.58) | 176.43 | 1.88(1.58) | 3.68(3.09) | unknown |
| nervous system disorders | serotonin syndrome | 25 | 8.11(5.46, 12.04) | 8.09(5.47, 11.97) | 153.37 | 3(2.44) | 8(5.75) | unknown |
| investigations | renal function test abnormal | 3 | 5.49(1.76, 17.1) | 5.49(1.76, 17.11) | 10.92 | 2.45(1.02) | 5.45(2.11) | unknown |
| injury, poisoning and procedural complications | maternal exposure during pregnancy | 29 | 3.06(2.13, 4.42) | 3.06(2.11, 4.44) | 40 | 1.61(1.09) | 3.05(2.24) | unknown |
| investigations | forced expiratory volume abnormal | 6 | 431.98(153.72, 1213.94) | 431.66(152.76, 1219.8) | 1546.78 | 8.02(6.74) | 259.39(109.27) | unknown |
| blood and lymphatic system disorders | splenic infarction | 5 | 20.9(8.58, 50.93) | 20.89(8.65, 50.46) | 91.72 | 4.34(3.16) | 20.27(9.62) | unknown |
| cardiac disorders | cardiopulmonary failure | 3 | 6.65(2.13, 20.76) | 6.65(2.13, 20.73) | 14.26 | 2.72(1.3) | 6.59(2.55) | unknown |
| injury, poisoning and procedural complications | product label confusion | 19 | 6.73(4.28, 10.59) | 6.72(4.28, 10.55) | 91.57 | 2.74(2.1) | 6.66(4.56) | unknown |
| hepatobiliary disorders | hepatosplenomegaly | 3 | 9.3(2.97, 29.07) | 9.29(2.98, 28.95) | 21.89 | 3.2(1.77) | 9.18(3.54) | unknown |
| respiratory, thoracic and mediastinal disorders | hypoventilation | 3 | 11.04(3.53, 34.57) | 11.04(3.54, 34.41) | 26.92 | 3.44(2.01) | 10.87(4.18) | unknown |
| infections and infestations | neonatal candida infection | 3 | 149.48(42.59, 524.65) | 149.42(42.62, 523.83) | 359.35 | 6.93(5.35) | 121.59(42.52) | unknown |
| eye disorders | retinal artery occlusion | 4 | 9.32(3.47, 25.02) | 9.32(3.5, 24.83) | 29.28 | 3.2(1.93) | 9.2(4.03) | unknown |
| gastrointestinal disorders | oral mucosal exfoliation | 4 | 23.77(8.76, 64.49) | 23.76(8.74, 64.56) | 84.13 | 4.52(3.23) | 22.96(9.96) | unknown |
| gastrointestinal disorders | oral mucosa erosion | 4 | 27.57(10.13, 75) | 27.55(10.14, 74.86) | 98.18 | 4.73(3.43) | 26.47(11.46) | unknown |
| infections and infestations | cavernous sinus thrombosis | 3 | 77.73(23.46, 257.49) | 77.7(23.51, 256.84) | 202.8 | 6.12(4.61) | 69.48(25.5) | unknown |
| hepatobiliary disorders | hepatomegaly | 7 | 6.22(2.95, 13.1) | 6.22(2.95, 13.1) | 30.36 | 2.62(1.62) | 6.17(3.31) | unknown |
| injury, poisoning and procedural complications | exposure during pregnancy | 41 | 4.26(3.13, 5.79) | 4.24(3.1, 5.8) | 100.92 | 2.08(1.64) | 4.22(3.26) | unknown |
| infections and infestations | infectious pleural effusion | 3 | 17.2(5.46, 54.13) | 17.19(5.52, 53.58) | 44.56 | 4.07(2.63) | 16.77(6.42) | unknown |
| blood and lymphatic system disorders | lymphadenopathy | 32 | 8.66(6.11, 12.28) | 8.63(6.06, 12.28) | 213.11 | 3.09(2.6) | 8.53(6.37) | unknown |
| eye disorders | glaucoma | 27 | 12.29(8.39, 18) | 12.25(8.44, 17.78) | 273.88 | 3.59(3.05) | 12.04(8.75) | unknown |
| injury, poisoning and procedural complications | transcription medication error | 8 | 4.82(2.4, 9.66) | 4.81(2.42, 9.55) | 24 | 2.26(1.31) | 4.79(2.67) | unknown |
| blood and lymphatic system disorders | leukaemoid reaction | 3 | 92.53(27.59, 310.29) | 92.5(27.44, 311.81) | 237.59 | 6.34(4.82) | 81.06(29.45) | unknown |
| infections and infestations | bone abscess | 3 | 60.72(18.59, 198.35) | 60.7(18.73, 196.75) | 161.06 | 5.8(4.31) | 55.58(20.65) | unknown |
| injury, poisoning and procedural complications | vascular pseudoaneurysm | 3 | 11.1(3.55, 34.77) | 11.1(3.56, 34.6) | 27.11 | 3.45(2.02) | 10.93(4.21) | unknown |
| skin and subcutaneous tissue disorders | skin reaction | 9 | 3.63(1.88, 6.99) | 3.63(1.9, 6.93) | 17.03 | 1.85(0.96) | 3.61(2.09) | unknown |
| infections and infestations | arthritis infective | 3 | 7.53(2.41, 23.51) | 7.53(2.42, 23.47) | 16.79 | 2.9(1.47) | 7.45(2.88) | unknown |
| blood and lymphatic system disorders | haemorrhagic disorder | 19 | 104.5(64.34, 169.73) | 104.26(63.87, 170.19) | 1673.62 | 6.49(5.81) | 89.94(59.94) | unknown |
| eye disorders | pupillary reflex impaired | 3 | 20.89(6.62, 65.98) | 20.89(6.57, 66.4) | 55.03 | 4.34(2.9) | 20.27(7.74) | unknown |
| immune system disorders | type iii immune complex mediated reaction | 21 | 227.22(138.17, 373.66) | 226.62(138.83, 369.92) | 3494.2 | 7.39(6.71) | 168.13(110.88) | unknown |
| eye disorders | corneal oedema | 6 | 11.93(5.32, 26.75) | 11.92(5.34, 26.62) | 58.92 | 3.55(2.47) | 11.72(5.96) | unknown |
| injury, poisoning and procedural complications | transplant failure | 8 | 9.05(4.5, 18.19) | 9.04(4.46, 18.31) | 56.42 | 3.16(2.21) | 8.93(4.98) | unknown |
| skin and subcutaneous tissue disorders | skin warm | 5 | 10.42(4.3, 25.21) | 10.41(4.31, 25.15) | 41.86 | 3.36(2.19) | 10.26(4.9) | unknown |
| infections and infestations | sepsis neonatal | 15 | 42.12(24.97, 71.04) | 42.04(24.76, 71.37) | 564.4 | 5.31(4.58) | 39.54(25.53) | unknown |
| blood and lymphatic system disorders | thrombocytopenia neonatal | 3 | 18.68(5.93, 58.89) | 18.68(5.88, 59.37) | 48.79 | 4.18(2.74) | 18.18(6.96) | unknown |
| infections and infestations | abscess limb | 5 | 12.32(5.08, 29.85) | 12.31(5.1, 29.74) | 50.99 | 3.6(2.43) | 12.1(5.77) | unknown |
| injury, poisoning and procedural complications | product appearance confusion | 6 | 8.45(3.78, 18.92) | 8.45(3.78, 18.87) | 38.88 | 3.06(1.98) | 8.35(4.25) | unknown |
| investigations | peritoneal effluent leukocyte count increased | 4 | 863.75(193.28, 3859.98) | 863.31(194.64, 3829.06) | 1476.54 | 8.53(6.92) | 370.56(105.88) | unknown |
| infections and infestations | mycobacterial infection | 3 | 13.13(4.19, 41.18) | 13.12(4.21, 40.89) | 32.94 | 3.69(2.25) | 12.88(4.95) | unknown |
| investigations | apgar score low | 5 | 8.16(3.38, 19.72) | 8.15(3.37, 19.69) | 31 | 3.01(1.85) | 8.07(3.85) | unknown |
| infections and infestations | alpha haemolytic streptococcal infection | 5 | 42.62(17.24, 105.38) | 42.6(17.29, 104.95) | 190.57 | 5.32(4.13) | 40.03(18.77) | unknown |
| skin and subcutaneous tissue disorders | palmar erythema | 4 | 28.47(10.46, 77.52) | 28.46(10.47, 77.33) | 101.52 | 4.77(3.47) | 27.3(11.81) | unknown |
| injury, poisoning and procedural complications | product communication issue | 7 | 8.17(3.88, 17.23) | 8.17(3.88, 17.21) | 43.48 | 3.01(2.01) | 8.08(4.33) | unknown |
| general disorders and administration site conditions | brain death | 3 | 8.23(2.64, 25.72) | 8.23(2.64, 25.65) | 18.82 | 3.03(1.6) | 8.14(3.14) | unknown |
| respiratory, thoracic and mediastinal disorders | pulmonary cavitation | 3 | 23.13(7.31, 73.19) | 23.12(7.27, 73.49) | 61.31 | 4.48(3.04) | 22.36(8.53) | unknown |

**Abbreviations:** SOC = system organ classe , ROR = Reporting Odds Ratio, PRR = Proportional Reporting Ratio,EBGM = Empirical Bayes Geometric Mean ,IC=Information Component, PT = preferred term
