# Supplementary material for: Evaluating adverse reaction signals of vancomycin in pediatric patients: A FAERS database analysis
Source: Medicine (Baltimore). 2026 Jun 5;105(23):e49064. doi: 10.1097/MD.0000000000049064 (PMC13246103; doi:10.1097/MD.0000000000049064)
Supplement: Supplementary file 7 [file medi-105-e49064-s008.docx]

**Table S8:**Signal Strength of Adverse Events Associated with Vancomycin at the PT Level by Age.

| **SOC** | **PT** | **Case Reports** | **ROR(95% CI)** | **PRR(95% CI)** | **chisq** | **IC(IC025)** | **EBGM(EBGM05)** | **group** |
| --- | --- | --- | --- | --- | --- | --- | --- | --- |
| renal and urinary disorders | acute kidney injury | 36 | 30.01(21.1, 42.68) | 28.96(20.75, 40.41) | 865.65 | 4.69(4.19) | 25.87(19.27) | <1 |
| skin and subcutaneous tissue disorders | drug reaction with eosinophilia and systemic symptoms | 6 | 33.52(14.22, 79.04) | 33.33(14.07, 78.95) | 164.65 | 4.87(3.73) | 29.28(14.29) | <1 |
| renal and urinary disorders | nephropathy toxic | 23 | 177.17(102.93, 304.96) | 173.08(101.96, 293.82) | 2259.43 | 6.64(5.94) | 99.78(63.34) | <1 |
| investigations | drug level increased | 12 | 20.08(11.1, 36.33) | 19.85(11.03, 35.74) | 198.12 | 4.2(3.38) | 18.37(11.19) | <1 |
| renal and urinary disorders | renal impairment | 12 | 17.27(9.58, 31.13) | 17.07(9.48, 30.73) | 169.28 | 4(3.18) | 15.97(9.75) | <1 |
| skin and subcutaneous tissue disorders | rash maculo-papular | 3 | 7.31(2.31, 23.1) | 7.29(2.29, 23.17) | 15.79 | 2.83(1.38) | 7.1(2.71) | <1 |
| general disorders and administration site conditions | extravasation | 3 | 13.76(4.29, 44.16) | 13.72(4.32, 43.61) | 33.43 | 3.7(2.24) | 13.02(4.91) | <1 |
| general disorders and administration site conditions | condition aggravated | 16 | 4.3(2.61, 7.09) | 4.25(2.6, 6.94) | 39.22 | 2.07(1.37) | 4.19(2.76) | <1 |
| blood and lymphatic system disorders | disseminated intravascular coagulation | 5 | 5.4(2.22, 13.13) | 5.38(2.23, 13) | 17.42 | 2.4(1.23) | 5.28(2.51) | <1 |
| renal and urinary disorders | renal tubular disorder | 6 | 32.74(13.9, 77.1) | 32.55(14.01, 75.61) | 161.05 | 4.84(3.7) | 28.69(14.01) | <1 |
| investigations | c-reactive protein increased | 4 | 6.69(2.47, 18.1) | 6.67(2.45, 18.12) | 18.74 | 2.7(1.41) | 6.51(2.83) | <1 |
| vascular disorders | shock | 4 | 5.09(1.89, 13.73) | 5.07(1.9, 13.51) | 12.81 | 2.32(1.03) | 4.98(2.17) | <1 |
| general disorders and administration site conditions | drug ineffective | 60 | 5.29(4.06, 6.88) | 5.03(3.9, 6.49) | 191.85 | 2.31(1.93) | 4.94(3.96) | <1 |
| injury, poisoning and procedural complications | accidental overdose | 13 | 4.85(2.79, 8.43) | 4.8(2.77, 8.31) | 38.41 | 2.24(1.47) | 4.72(2.97) | <1 |
| investigations | blood urea increased | 5 | 14.83(5.99, 36.71) | 14.76(5.99, 36.36) | 60.36 | 3.8(2.6) | 13.94(6.53) | <1 |
| investigations | blood creatinine increased | 14 | 21.78(12.55, 37.79) | 21.49(12.41, 37.2) | 250.57 | 4.3(3.54) | 19.76(12.46) | <1 |
| general disorders and administration site conditions | drug interaction | 13 | 8.34(4.78, 14.55) | 8.24(4.76, 14.26) | 80.02 | 3(2.22) | 7.99(5.02) | <1 |
| metabolism and nutrition disorders | electrolyte imbalance | 4 | 11.86(4.33, 32.43) | 11.81(4.35, 32.09) | 37.69 | 3.5(2.19) | 11.29(4.86) | <1 |
| renal and urinary disorders | nephrocalcinosis | 4 | 8.07(2.97, 21.92) | 8.04(2.96, 21.85) | 23.86 | 2.97(1.67) | 7.81(3.39) | <1 |
| immune system disorders | drug hypersensitivity | 3 | 5.44(1.73, 17.11) | 5.43(1.74, 16.92) | 10.59 | 2.41(0.97) | 5.32(2.04) | <1 |
| injury, poisoning and procedural complications | toxicity to various agents | 16 | 4.06(2.47, 6.69) | 4.01(2.46, 6.55) | 35.73 | 1.99(1.29) | 3.96(2.61) | <1 |
| skin and subcutaneous tissue disorders | erythema | 8 | 4.68(2.32, 9.46) | 4.65(2.3, 9.42) | 22.54 | 2.2(1.24) | 4.58(2.55) | <1 |
| blood and lymphatic system disorders | lymphadenopathy | 6 | 16.96(7.39, 38.93) | 16.86(7.4, 38.4) | 83.53 | 3.98(2.87) | 15.79(7.88) | <1 |
| general disorders and administration site conditions | paradoxical drug reaction | 3 | 13.76(4.29, 44.16) | 13.72(4.32, 43.61) | 33.43 | 3.7(2.24) | 13.02(4.91) | <1 |
| respiratory, thoracic and mediastinal disorders | acute respiratory distress syndrome | 4 | 5.41(2, 14.61) | 5.39(2.02, 14.36) | 14 | 2.4(1.12) | 5.29(2.31) | <1 |
| infections and infestations | pathogen resistance | 4 | 18.37(6.63, 50.91) | 18.3(6.6, 50.71) | 60.66 | 4.09(2.77) | 17.04(7.26) | <1 |
| renal and urinary disorders | renal failure neonatal | 3 | 6.95(2.2, 21.94) | 6.93(2.22, 21.6) | 14.79 | 2.76(1.31) | 6.76(2.58) | <1 |
| general disorders and administration site conditions | necrosis | 3 | 10.63(3.34, 33.87) | 10.6(3.33, 33.69) | 24.97 | 3.35(1.89) | 10.19(3.86) | <1 |
| gastrointestinal disorders | necrotising enterocolitis neonatal | 6 | 9.14(4.03, 20.71) | 9.09(4.07, 20.3) | 41.6 | 3.14(2.04) | 8.79(4.43) | <1 |
| eye disorders | retinopathy | 4 | 34.7(12.12, 99.34) | 34.56(12.23, 97.66) | 113.55 | 4.92(3.56) | 30.23(12.54) | <1 |
| respiratory, thoracic and mediastinal disorders | hypoxia | 9 | 5.04(2.59, 9.77) | 5(2.57, 9.74) | 28.24 | 2.3(1.39) | 4.91(2.82) | <1 |
| renal and urinary disorders | proteinuria | 4 | 13.77(5.01, 37.83) | 13.72(5.05, 37.28) | 44.57 | 3.7(2.39) | 13.02(5.59) | <1 |
| skin and subcutaneous tissue disorders | toxic epidermal necrolysis | 4 | 13.19(4.81, 36.19) | 13.14(4.84, 35.7) | 42.49 | 3.64(2.33) | 12.49(5.37) | <1 |
| skin and subcutaneous tissue disorders | dermatitis | 3 | 10.96(3.44, 34.96) | 10.93(3.44, 34.74) | 25.87 | 3.39(1.93) | 10.49(3.98) | <1 |
| general disorders and administration site conditions | infusion site extravasation | 3 | 13.76(4.29, 44.16) | 13.72(4.32, 43.61) | 33.43 | 3.7(2.24) | 13.02(4.91) | <1 |
| skin and subcutaneous tissue disorders | skin discolouration | 6 | 5.89(2.61, 13.26) | 5.86(2.62, 13.09) | 23.6 | 2.52(1.43) | 5.74(2.91) | <1 |
| renal and urinary disorders | neonatal anuria | 3 | 21.27(6.51, 69.46) | 21.21(6.54, 68.75) | 52.96 | 4.29(2.8) | 19.52(7.25) | <1 |
| respiratory, thoracic and mediastinal disorders | pulmonary necrosis | 3 | 350.97(58.58, 2102.81) | 349.91(58.8, 2082.4) | 417.51 | 7.14(5.31) | 140.57(31.43) | <1 |
| investigations | antibiotic level above therapeutic | 3 | 701.95(72.95, 6754.34) | 699.83(73.47, 6666.41) | 523.38 | 7.46(5.56) | 175.71(26.43) | <1 |
| infections and infestations | device related infection | 3 | 7.63(2.41, 24.13) | 7.61(2.39, 24.19) | 16.68 | 2.89(1.44) | 7.4(2.82) | <1 |
| infections and infestations | enterococcal infection | 4 | 19.51(7.02, 54.22) | 19.44(7.02, 53.87) | 64.6 | 4.17(2.85) | 18.02(7.66) | <1 |
| immune system disorders | haemophagocytic lymphohistiocytosis | 8 | 40.89(19.25, 86.87) | 40.57(19.26, 85.44) | 263.07 | 5.12(4.1) | 34.71(18.48) | <1 |
| nervous system disorders | generalised tonic-clonic seizure | 5 | 8.19(3.35, 20.03) | 8.16(3.38, 19.71) | 30.35 | 2.98(1.8) | 7.91(3.75) | <1 |
| blood and lymphatic system disorders | methaemoglobinaemia | 3 | 7.71(2.44, 24.4) | 7.69(2.42, 24.44) | 16.91 | 2.9(1.46) | 7.48(2.85) | <1 |
| infections and infestations | empyema | 3 | 46.79(13.53, 161.89) | 46.66(13.57, 160.4) | 111.7 | 5.29(3.73) | 39.05(13.82) | <1 |
| hepatobiliary disorders | hepatosplenomegaly | 3 | 10.02(3.15, 31.89) | 10(3.15, 31.78) | 23.3 | 3.27(1.81) | 9.63(3.66) | <1 |
| general disorders and administration site conditions | therapeutic response decreased | 3 | 9.36(2.95, 29.72) | 9.33(2.94, 29.66) | 21.46 | 3.17(1.72) | 9.01(3.43) | <1 |
| skin and subcutaneous tissue disorders | skin necrosis | 4 | 18.37(6.63, 50.91) | 18.3(6.6, 50.71) | 60.66 | 4.09(2.77) | 17.04(7.26) | <1 |
| infections and infestations | klebsiella infection | 3 | 11.7(3.66, 37.36) | 11.66(3.67, 37.06) | 27.86 | 3.48(2.02) | 11.16(4.22) | <1 |
| investigations | transaminases increased | 4 | 5.03(1.87, 13.58) | 5.02(1.88, 13.38) | 12.6 | 2.3(1.02) | 4.93(2.15) | <1 |
| injury, poisoning and procedural complications | product administration error | 3 | 6.21(1.97, 19.57) | 6.19(1.99, 19.29) | 12.73 | 2.6(1.16) | 6.06(2.32) | <1 |
| injury, poisoning and procedural complications | product preparation error | 4 | 13.19(4.81, 36.19) | 13.14(4.84, 35.7) | 42.49 | 3.64(2.33) | 12.49(5.37) | <1 |
| infections and infestations | candida infection | 18 | 45.97(27.64, 76.44) | 45.15(27.12, 75.16) | 651.4 | 5.25(4.54) | 37.99(24.82) | <1 |
| infections and infestations | systemic candida | 10 | 67.32(33.24, 136.33) | 66.65(32.91, 134.97) | 503.05 | 5.7(4.75) | 52.06(28.85) | <1 |
| immune system disorders | anaphylactic reaction | 6 | 9.57(4.22, 21.71) | 9.52(4.18, 21.68) | 43.99 | 3.2(2.1) | 9.19(4.63) | <1 |
| injury, poisoning and procedural complications | wrong product administered | 5 | 15.02(6.07, 37.19) | 14.95(6.07, 36.83) | 61.2 | 3.82(2.62) | 14.11(6.61) | <1 |
| infections and infestations | endocarditis | 4 | 55.11(18.51, 164.07) | 54.89(18.68, 161.31) | 171.33 | 5.48(4.08) | 44.62(17.91) | <1 |
| eye disorders | retinal haemorrhage | 4 | 27.55(9.76, 77.79) | 27.44(9.71, 77.54) | 91.21 | 4.62(3.28) | 24.66(10.35) | <1 |
| blood and lymphatic system disorders | eosinophilia | 8 | 30.34(14.49, 63.52) | 30.1(14.58, 62.16) | 199.4 | 4.74(3.74) | 26.77(14.43) | <1 |
| investigations | drug level above therapeutic | 4 | 39.03(13.52, 112.71) | 38.88(13.49, 112.04) | 126.55 | 5.06(3.69) | 33.47(13.78) | <1 |
| general disorders and administration site conditions | drug ineffective for unapproved indication | 9 | 7.08(3.64, 13.78) | 7.02(3.61, 13.67) | 45.18 | 2.78(1.86) | 6.85(3.92) | <1 |
| infections and infestations | neonatal candida infection | 6 | 281.63(85.81, 924.35) | 279.93(86.36, 907.36) | 758.04 | 7(5.65) | 127.79(47.27) | <1 |
| vascular disorders | haemodynamic instability | 4 | 9.86(3.62, 26.86) | 9.82(3.61, 26.68) | 30.43 | 3.24(1.94) | 9.47(4.09) | <1 |
| metabolism and nutrition disorders | acidosis | 4 | 5.64(2.09, 15.23) | 5.62(2.11, 14.97) | 14.85 | 2.46(1.18) | 5.51(2.4) | <1 |
| nervous system disorders | serotonin syndrome | 3 | 18(5.55, 58.33) | 17.94(5.53, 58.15) | 44.58 | 4.06(2.58) | 16.73(6.26) | <1 |
| skin and subcutaneous tissue disorders | palmar-plantar erythrodysaesthesia syndrome | 3 | 350.97(58.58, 2102.81) | 349.91(58.8, 2082.4) | 417.51 | 7.14(5.31) | 140.57(31.43) | <1 |
| skin and subcutaneous tissue disorders | angioedema | 3 | 13.24(4.13, 42.45) | 13.2(4.15, 41.96) | 32.03 | 3.65(2.18) | 12.55(4.74) | <1 |
| infections and infestations | cns ventriculitis | 3 | 63.81(17.77, 229.08) | 63.62(17.8, 227.45) | 145.29 | 5.65(4.06) | 50.2(17.23) | <1 |
| skin and subcutaneous tissue disorders | dermatitis exfoliative | 68 | 44.62(34.43, 57.82) | 41.8(33.04, 52.88) | 2422.97 | 5.23(4.86) | 37.44(30.14) | 1~6 |
| general disorders and administration site conditions | generalised oedema | 34 | 30.26(21.21, 43.17) | 29.32(20.6, 41.72) | 858.88 | 4.76(4.26) | 27.12(20.15) | 1~6 |
| general disorders and administration site conditions | face oedema | 10 | 18.55(9.79, 35.13) | 18.38(9.82, 34.41) | 156.2 | 4.13(3.25) | 17.51(10.26) | 1~6 |
| blood and lymphatic system disorders | pancytopenia | 6 | 7.24(3.22, 16.27) | 7.2(3.22, 16.08) | 31.41 | 2.82(1.74) | 7.07(3.59) | 1~6 |
| cardiac disorders | cardiac failure congestive | 17 | 17.22(10.54, 28.13) | 16.96(10.39, 27.68) | 243.75 | 4.02(3.33) | 16.22(10.76) | 1~6 |
| cardiac disorders | myocarditis | 10 | 8(4.26, 15.02) | 7.94(4.24, 14.87) | 59.35 | 2.96(2.09) | 7.78(4.59) | 1~6 |
| metabolism and nutrition disorders | hypoalbuminaemia | 6 | 33.98(14.67, 78.73) | 33.79(14.55, 78.49) | 174.12 | 4.95(3.82) | 30.9(15.3) | 1~6 |
| blood and lymphatic system disorders | leukocytosis | 36 | 14.08(10.04, 19.76) | 13.63(9.77, 19.02) | 406.7 | 3.72(3.24) | 13.16(9.91) | 1~6 |
| immune system disorders | anaphylactoid reaction | 25 | 5.6(3.75, 8.35) | 5.49(3.71, 8.12) | 90.71 | 2.44(1.87) | 5.42(3.88) | 1~6 |
| skin and subcutaneous tissue disorders | cold sweat | 5 | 5.05(2.08, 12.24) | 5.03(2.08, 12.15) | 15.94 | 2.31(1.15) | 4.97(2.37) | 1~6 |
| eye disorders | periorbital oedema | 3 | 7.72(2.46, 24.28) | 7.7(2.47, 24) | 17.13 | 2.92(1.48) | 7.56(2.9) | 1~6 |
| infections and infestations | cellulitis | 5 | 10.08(4.13, 24.57) | 10.03(4.15, 24.23) | 39.55 | 3.29(2.11) | 9.78(4.64) | 1~6 |
| metabolism and nutrition disorders | hyperkalaemia | 3 | 6.78(2.16, 21.27) | 6.76(2.17, 21.07) | 14.45 | 2.73(1.3) | 6.65(2.55) | 1~6 |
| blood and lymphatic system disorders | agranulocytosis | 7 | 4.03(1.91, 8.5) | 4.01(1.9, 8.45) | 15.64 | 1.99(0.98) | 3.97(2.13) | 1~6 |
| infections and infestations | stenotrophomonas infection | 3 | 19.82(6.18, 63.52) | 19.77(6.22, 62.84) | 50.59 | 4.23(2.76) | 18.76(7.08) | 1~6 |
| general disorders and administration site conditions | potentiating drug interaction | 3 | 9.82(3.11, 30.97) | 9.79(3.14, 30.51) | 23.04 | 3.26(1.81) | 9.55(3.65) | 1~6 |
| renal and urinary disorders | renal tubular necrosis | 5 | 6.47(2.67, 15.7) | 6.44(2.67, 15.56) | 22.59 | 2.67(1.5) | 6.34(3.02) | 1~6 |
| infections and infestations | clostridium difficile infection | 11 | 6.51(3.58, 11.86) | 6.46(3.59, 11.63) | 49.88 | 2.67(1.84) | 6.36(3.85) | 1~6 |
| general disorders and administration site conditions | drug chemical incompatibility | 3 | 12.97(4.09, 41.12) | 12.93(4.07, 41.1) | 31.86 | 3.64(2.2) | 12.51(4.76) | 1~6 |
| investigations | antibiotic level below therapeutic | 7 | 50.21(22.69, 111.11) | 49.88(22.77, 109.25) | 293.46 | 5.45(4.38) | 43.77(22.52) | 1~6 |
| investigations | eosinophil count increased | 4 | 6.46(2.4, 17.39) | 6.44(2.42, 17.16) | 18.05 | 2.66(1.38) | 6.34(2.77) | 1~6 |
| investigations | liver function test increased | 4 | 8.49(3.14, 22.94) | 8.47(3.12, 23.01) | 25.72 | 3.05(1.77) | 8.29(3.61) | 1~6 |
| immune system disorders | type iv hypersensitivity reaction | 3 | 14.79(4.65, 47.04) | 14.75(4.64, 46.88) | 36.92 | 3.83(2.37) | 14.2(5.39) | 1~6 |
| blood and lymphatic system disorders | autoimmune haemolytic anaemia | 3 | 16.67(5.23, 53.18) | 16.63(5.23, 52.86) | 42.07 | 3.99(2.53) | 15.92(6.03) | 1~6 |
| injury, poisoning and procedural complications | wrong rate | 6 | 21.28(9.31, 48.62) | 21.16(9.29, 48.2) | 108.71 | 4.32(3.22) | 20.01(10.02) | 1~6 |
| skin and subcutaneous tissue disorders | linear iga disease | 4 | 5.65(2.1, 15.2) | 5.63(2.11, 15) | 15.01 | 2.47(1.19) | 5.56(2.43) | 1~6 |
| investigations | drug level below therapeutic | 5 | 7.72(3.18, 18.77) | 7.69(3.18, 18.58) | 28.5 | 2.92(1.74) | 7.55(3.59) | 1~6 |
| injury, poisoning and procedural complications | poor quality product administered | 9 | 16.16(8.26, 31.62) | 16.03(8.23, 31.21) | 121.37 | 3.94(3.02) | 15.37(8.77) | 1~6 |
| skin and subcutaneous tissue disorders | pruritus | 3 | 21.01(6.54, 67.46) | 20.95(6.59, 66.59) | 53.78 | 4.31(2.84) | 19.82(7.47) | 1~6 |
| injury, poisoning and procedural complications | infusion related reaction | 5 | 48.73(19.08, 124.41) | 48.5(18.93, 124.26) | 204.26 | 5.42(4.18) | 42.71(19.49) | 1~6 |
| renal and urinary disorders | renal failure | 3 | 14(4.41, 44.48) | 13.97(4.4, 44.4) | 34.74 | 3.75(2.3) | 13.47(5.12) | 1~6 |
| infections and infestations | lower respiratory tract infection | 5 | 8.77(3.6, 21.34) | 8.73(3.61, 21.09) | 33.41 | 3.09(1.92) | 8.54(4.06) | 1~6 |
| cardiac disorders | cardiac arrest | 4 | 1402.1(156.58, 12555.41) | 1396.78(155.51, 12545.59) | 1115.84 | 8.13(6.42) | 280.16(44.75) | 1~6 |
| blood and lymphatic system disorders | leukopenia | 6 | 2107.16(253.45, 17518.36) | 2095.17(252.29, 17399.38) | 1794.18 | 8.23(6.76) | 300.17(51.02) | 1~6 |
| blood and lymphatic system disorders | neutropenia | 4 | 19.2(7.01, 52.63) | 19.13(7.04, 51.98) | 65.19 | 4.19(2.88) | 18.19(7.82) | 1~6 |
| skin and subcutaneous tissue disorders | rash morbilliform | 4 | 9.6(3.55, 25.97) | 9.57(3.52, 26) | 29.88 | 3.22(1.93) | 9.34(4.06) | 1~6 |
| renal and urinary disorders | tubulointerstitial nephritis | 3 | 17.51(5.48, 55.91) | 17.46(5.49, 55.5) | 44.34 | 4.06(2.6) | 16.68(6.31) | 1~6 |
| injury, poisoning and procedural complications | incorrect drug administration rate | 4 | 12.63(4.65, 34.3) | 12.58(4.63, 34.18) | 41.18 | 3.61(2.31) | 12.18(5.28) | 1~6 |
| skin and subcutaneous tissue disorders | drug eruption | 3 | 1050.57(109.19, 10108.3) | 1047.58(109.97, 9979) | 784.2 | 8.04(6.14) | 262.65(39.5) | 1~6 |
| skin and subcutaneous tissue disorders | toxic skin eruption | 3 | 19.45(6.07, 62.31) | 19.4(6.1, 61.66) | 49.61 | 4.2(2.74) | 18.43(6.96) | 1~6 |
| general disorders and administration site conditions | multiple organ dysfunction syndrome | 3 | 22.35(6.95, 71.92) | 22.29(7.01, 70.85) | 57.35 | 4.39(2.92) | 21.01(7.9) | 1~6 |
| general disorders and administration site conditions | hyperthermia | 3 | 11.06(3.5, 34.95) | 11.03(3.47, 35.06) | 26.52 | 3.42(1.98) | 10.72(4.09) | 1~6 |
| gastrointestinal disorders | lip erythema | 64 | 51.8(39.73, 67.53) | 48.6(37.67, 62.7) | 2705.85 | 5.46(5.09) | 44.11(35.33) | 6~12 |
| gastrointestinal disorders | lip swelling | 29 | 24.65(16.88, 35.99) | 23.97(16.52, 34.79) | 607.98 | 4.51(3.98) | 22.85(16.65) | 6~12 |
| infections and infestations | meningitis | 8 | 23.49(11.52, 47.91) | 23.31(11.51, 47.2) | 162.76 | 4.48(3.5) | 22.25(12.26) | 6~12 |
| general disorders and administration site conditions | localised oedema | 7 | 10.77(5.08, 22.84) | 10.7(5.08, 22.53) | 60.22 | 3.39(2.37) | 10.48(5.59) | 6~12 |
| nervous system disorders | posterior reversible encephalopathy syndrome | 5 | 4.76(1.97, 11.5) | 4.74(1.96, 11.45) | 14.61 | 2.23(1.07) | 4.7(2.25) | 6~12 |
| gastrointestinal disorders | enterocolitis haemorrhagic | 5 | 5.46(2.25, 13.2) | 5.43(2.25, 13.12) | 17.9 | 2.43(1.26) | 5.38(2.57) | 6~12 |
| musculoskeletal and connective tissue disorders | myositis | 4 | 55.06(19.5, 155.46) | 54.85(19.41, 155) | 189.24 | 5.62(4.27) | 49.18(20.64) | 6~12 |
| infections and infestations | subperiosteal abscess | 22 | 6.37(4.16, 9.75) | 6.25(4.14, 9.43) | 96.17 | 2.63(2.03) | 6.18(4.33) | 6~12 |
| eye disorders | eyelid oedema | 12 | 5.91(3.33, 10.48) | 5.85(3.31, 10.33) | 47.78 | 2.53(1.74) | 5.79(3.59) | 6~12 |
| metabolism and nutrition disorders | hypokalaemia | 5 | 4.48(1.85, 10.84) | 4.47(1.85, 10.8) | 13.34 | 2.15(0.98) | 4.43(2.12) | 6~12 |
| investigations | antimicrobial susceptibility test resistant | 5 | 156.18(56.65, 430.53) | 155.41(56.08, 430.64) | 575.35 | 6.87(5.56) | 116.81(50) | 6~12 |
| general disorders and administration site conditions | therapy non-responder | 8 | 10.26(5.08, 20.74) | 10.19(5.03, 20.64) | 64.95 | 3.32(2.36) | 9.99(5.55) | 6~12 |
| general disorders and administration site conditions | impaired healing | 3 | 7.54(2.41, 23.63) | 7.52(2.41, 23.44) | 16.7 | 2.89(1.46) | 7.42(2.85) | 6~12 |
| injury, poisoning and procedural complications | incorrect dosage administered | 10 | 5.01(2.68, 9.37) | 4.97(2.65, 9.31) | 31.45 | 2.3(1.44) | 4.93(2.92) | 6~12 |
| infections and infestations | overgrowth fungal | 3 | 10.09(3.21, 31.72) | 10.06(3.23, 31.35) | 23.98 | 3.3(1.87) | 9.87(3.79) | 6~12 |
| infections and infestations | mucormycosis | 3 | 32.62(10.1, 105.32) | 32.53(10.04, 105.44) | 85.71 | 4.93(3.45) | 30.47(11.43) | 6~12 |
| infections and infestations | rhinocerebral mucormycosis | 17 | 10.23(6.3, 16.6) | 10.07(6.29, 16.12) | 136.19 | 3.3(2.62) | 9.88(6.59) | 6~12 |
| infections and infestations | pseudomonal sepsis | 8 | 6.06(3.01, 12.2) | 6.02(3.03, 11.95) | 33.08 | 2.57(1.62) | 5.95(3.31) | 6~12 |
| skin and subcutaneous tissue disorders | vancomycin infusion reaction | 3 | 127.53(35.53, 457.82) | 127.16(35.57, 454.61) | 295.04 | 6.65(5.05) | 100.12(34.36) | 6~12 |
| injury, poisoning and procedural complications | wrong patient received product | 4 | 22.83(8.35, 62.4) | 22.74(8.37, 61.79) | 79.3 | 4.44(3.14) | 21.73(9.37) | 6~12 |
| injury, poisoning and procedural complications | drug dispensed to wrong patient | 4 | 12.56(4.64, 33.98) | 12.52(4.61, 34.02) | 41.29 | 3.61(2.32) | 12.22(5.31) | 6~12 |
| injury, poisoning and procedural complications | product dispensing error | 4 | 23.4(8.56, 64) | 23.31(8.58, 63.34) | 81.37 | 4.48(3.17) | 22.25(9.59) | 6~12 |
| general disorders and administration site conditions | pyrexia | 3 | 42.51(13.02, 138.83) | 42.39(13.08, 137.4) | 111.13 | 5.28(3.79) | 38.94(14.46) | 6~12 |
| skin and subcutaneous tissue disorders | skin disorder | 16 | 78.12(45.87, 133.05) | 76.91(45.31, 130.56) | 1029.22 | 6.05(5.31) | 66.16(42.37) | 6~12 |
| blood and lymphatic system disorders | coagulopathy | 22 | 3.55(2.32, 5.42) | 3.49(2.31, 5.27) | 39.11 | 1.8(1.2) | 3.48(2.44) | 6~12 |
| investigations | prothrombin time prolonged | 11 | 6.66(3.66, 12.12) | 6.6(3.67, 11.88) | 51.63 | 2.71(1.88) | 6.52(3.95) | 6~12 |
| skin and subcutaneous tissue disorders | rash erythematous | 9 | 7.56(3.9, 14.66) | 7.51(3.93, 14.34) | 50.01 | 2.89(1.98) | 7.4(4.26) | 6~12 |
| general disorders and administration site conditions | infusion site pain | 9 | 24.46(12.48, 47.95) | 24.26(12.46, 47.24) | 190.82 | 4.53(3.61) | 23.11(13.16) | 6~12 |
| vascular disorders | hypotension | 8 | 6(2.98, 12.08) | 5.96(3, 11.84) | 32.64 | 2.56(1.61) | 5.9(3.28) | 6~12 |
| metabolism and nutrition disorders | lactic acidosis | 8 | 6.97(3.46, 14.04) | 6.92(3.48, 13.74) | 39.97 | 2.77(1.82) | 6.83(3.8) | 6~12 |
| skin and subcutaneous tissue disorders | petechiae | 12 | 4(2.26, 7.08) | 3.96(2.24, 6.99) | 26.43 | 1.98(1.18) | 3.94(2.44) | 6~12 |
| cardiac disorders | tachycardia | 4 | 37.44(13.5, 103.87) | 37.3(13.46, 103.36) | 130.85 | 5.11(3.79) | 34.61(14.74) | 6~12 |
| vascular disorders | flushing | 3 | 7.15(2.28, 22.41) | 7.14(2.29, 22.25) | 15.6 | 2.82(1.39) | 7.04(2.71) | 6~12 |
| investigations | blood lactate dehydrogenase increased | 8 | 49.46(23.81, 102.75) | 49.08(23.77, 101.36) | 340.96 | 5.48(4.48) | 44.5(24.14) | 6~12 |
| hepatobiliary disorders | acute hepatic failure | 3 | 6.98(2.23, 21.85) | 6.96(2.23, 21.69) | 15.09 | 2.78(1.35) | 6.87(2.64) | 6~12 |
| nervous system disorders | hepatic encephalopathy | 6 | 41.38(17.92, 95.56) | 41.14(18.06, 93.71) | 215.96 | 5.24(4.12) | 37.88(18.81) | 6~12 |
| skin and subcutaneous tissue disorders | rash pruritic | 8 | 9.18(4.55, 18.54) | 9.12(4.5, 18.47) | 56.77 | 3.16(2.21) | 8.96(4.98) | 6~12 |
| skin and subcutaneous tissue disorders | skin lesion | 4 | 14.29(5.27, 38.72) | 14.24(5.24, 38.69) | 47.78 | 3.79(2.5) | 13.84(6.01) | 6~12 |
| vascular disorders | pallor | 4 | 98.54(33.46, 290.17) | 98.16(33.4, 288.47) | 317.78 | 6.34(4.95) | 81.26(32.92) | 6~12 |
| general disorders and administration site conditions | treatment failure | 5 | 4.94(2.04, 11.94) | 4.92(2.04, 11.89) | 15.46 | 2.29(1.12) | 4.88(2.33) | 6~12 |
| infections and infestations | septic shock | 3 | 13.11(4.15, 41.36) | 13.07(4.19, 40.74) | 32.54 | 3.67(2.23) | 12.74(4.87) | 6~12 |
| hepatobiliary disorders | cholestasis | 5 | 65.07(25.48, 166.16) | 64.76(25.28, 165.92) | 275.61 | 5.83(4.6) | 56.98(26.01) | 6~12 |
| hepatobiliary disorders | hepatic lesion | 11 | 5.8(3.19, 10.54) | 5.74(3.19, 10.33) | 42.65 | 2.51(1.68) | 5.69(3.45) | 6~12 |
| immune system disorders | anaphylactic shock | 4 | 98.54(33.46, 290.17) | 98.16(33.4, 288.47) | 317.78 | 6.34(4.95) | 81.26(32.92) | 6~12 |
| respiratory, thoracic and mediastinal disorders | bronchospasm | 4 | 27.13(9.88, 74.48) | 27.03(9.95, 73.45) | 94.78 | 4.68(3.37) | 25.6(11) | 6~12 |
| vascular disorders | hyperaemia | 4 | 1872.34(209.08, 16766.84) | 1864.97(207.64, 16750.78) | 1490.39 | 8.55(6.83) | 373.79(59.7) | 6~12 |
| general disorders and administration site conditions | chills | 3 | 6.43(2.06, 20.13) | 6.42(2.06, 20.01) | 13.54 | 2.67(1.24) | 6.34(2.44) | 6~12 |
| renal and urinary disorders | oliguria | 5 | 5.12(2.12, 12.39) | 5.1(2.11, 12.32) | 16.32 | 2.34(1.17) | 5.06(2.41) | 6~12 |
| eye disorders | blindness | 6 | 76.05(32.03, 180.59) | 75.61(31.92, 179.11) | 380.12 | 6.03(4.87) | 65.2(31.62) | 6~12 |
| skin and subcutaneous tissue disorders | purpura | 8 | 7.19(3.57, 14.5) | 7.15(3.6, 14.2) | 41.69 | 2.82(1.86) | 7.05(3.92) | 6~12 |
| gastrointestinal disorders | lip oedema | 3 | 13.75(4.35, 43.42) | 13.71(4.4, 42.73) | 34.36 | 3.74(2.3) | 13.35(5.1) | 6~12 |
| investigations | blood electrolytes abnormal | 3 | 20.94(6.57, 66.67) | 20.88(6.57, 66.37) | 54.35 | 4.32(2.87) | 20.02(7.6) | 6~12 |
| skin and subcutaneous tissue disorders | acute generalised exanthematous pustulosis | 3 | 233.81(58.39, 936.18) | 233.12(57.97, 937.44) | 462.26 | 7.28(5.61) | 155.75(48.79) | 6~12 |
| infections and infestations | toxic shock syndrome | 6 | 40.78(17.67, 94.13) | 40.54(17.8, 92.34) | 212.93 | 5.22(4.1) | 37.38(18.56) | 6~12 |
| infections and infestations | meningitis bacterial | 3 | 127.53(35.53, 457.82) | 127.16(35.57, 454.61) | 295.04 | 6.65(5.05) | 100.12(34.36) | 6~12 |
| hepatobiliary disorders | drug-induced liver injury | 3 | 28.05(8.74, 90.09) | 27.97(8.8, 88.9) | 73.62 | 4.73(3.26) | 26.45(9.96) | 6~12 |
| skin and subcutaneous tissue disorders | erythema multiforme | 6 | 562.81(171.49, 1847.12) | 559.49(172.61, 1813.52) | 1520.47 | 7.99(6.65) | 254.86(94.28) | 6~12 |
| infections and infestations | alpha haemolytic streptococcal infection | 3 | 28.63(8.91, 91.99) | 28.55(8.98, 90.75) | 75.15 | 4.75(3.28) | 26.96(10.15) | 6~12 |
| general disorders and administration site conditions | infusion site necrosis | 3 | 38.97(11.98, 126.74) | 38.85(11.99, 125.93) | 102.14 | 5.17(3.68) | 35.94(13.4) | 6~12 |
| cardiac disorders | cardiac dysfunction | 3 | 5.27(1.69, 16.48) | 5.26(1.69, 16.39) | 10.24 | 2.38(0.95) | 5.21(2.01) | 6~12 |
| cardiac disorders | ventricular tachycardia | 39 | 13.05(9.46, 18) | 12.7(9.28, 17.38) | 413.34 | 3.64(3.18) | 12.48(9.53) | 12~18 |
| infections and infestations | urosepsis | 65 | 44.7(34.57, 57.79) | 42.61(33.03, 54.98) | 2479.71 | 5.32(4.96) | 40.02(32.28) | 12~18 |
| ear and labyrinth disorders | deafness neurosensory | 18 | 34.45(21.38, 55.51) | 34.01(21.25, 54.44) | 547.92 | 5.02(4.35) | 32.35(21.7) | 12~18 |
| hepatobiliary disorders | liver disorder | 10 | 10.38(5.54, 19.43) | 10.31(5.51, 19.3) | 82.81 | 3.35(2.48) | 10.16(6.01) | 12~18 |
| skin and subcutaneous tissue disorders | stevens-johnson syndrome | 11 | 9.85(5.41, 17.9) | 9.77(5.43, 17.59) | 85.41 | 3.27(2.44) | 9.64(5.85) | 12~18 |
| respiratory, thoracic and mediastinal disorders | tachypnoea | 6 | 8.13(3.63, 18.22) | 8.1(3.63, 18.09) | 36.88 | 3(1.92) | 8.01(4.08) | 12~18 |
| infections and infestations | rash pustular | 6 | 73.04(31.35, 170.2) | 72.72(31.31, 168.92) | 381.29 | 6.03(4.9) | 65.43(32.24) | 12~18 |
| blood and lymphatic system disorders | thrombocytopenia | 6 | 4.78(2.14, 10.68) | 4.76(2.13, 10.63) | 17.7 | 2.24(1.17) | 4.73(2.41) | 12~18 |
| infections and infestations | clostridium colitis | 26 | 12.68(8.57, 18.76) | 12.46(8.42, 18.44) | 269.13 | 3.61(3.06) | 12.24(8.82) | 12~18 |
| renal and urinary disorders | anuria | 4 | 32.21(11.78, 88.05) | 32.12(11.82, 87.28) | 114.87 | 4.94(3.63) | 30.64(13.21) | 12~18 |
| infections and infestations | clostridial infection | 9 | 9.74(5.03, 18.86) | 9.68(5.07, 18.48) | 69.1 | 3.26(2.35) | 9.56(5.5) | 12~18 |
| infections and infestations | enterobacter infection | 8 | 27.35(13.45, 55.59) | 27.19(13.43, 55.06) | 193.67 | 4.71(3.74) | 26.13(14.43) | 12~18 |
| skin and subcutaneous tissue disorders | dermatitis bullous | 3 | 26.1(8.22, 82.88) | 26.04(8.19, 82.77) | 69.44 | 4.65(3.2) | 25.07(9.53) | 12~18 |
| respiratory, thoracic and mediastinal disorders | apnoea | 20 | 7.98(5.11, 12.43) | 7.87(5.11, 12.11) | 118.76 | 2.96(2.34) | 7.79(5.37) | 12~18 |
| renal and urinary disorders | nephritis allergic | 4 | 85.9(30.22, 244.17) | 85.65(30.31, 242.03) | 295.3 | 6.24(4.89) | 75.69(31.58) | 12~18 |
| nervous system disorders | encephalopathy | 4 | 8.34(3.1, 22.39) | 8.32(3.12, 22.17) | 25.42 | 3.04(1.76) | 8.22(3.6) | 12~18 |
| skin and subcutaneous tissue disorders | ecchymosis | 7 | 3.84(1.83, 8.1) | 3.83(1.82, 8.07) | 14.57 | 1.93(0.93) | 3.81(2.04) | 12~18 |
| psychiatric disorders | mental status changes | 6 | 4.42(1.98, 9.88) | 4.4(1.97, 9.83) | 15.7 | 2.13(1.06) | 4.38(2.23) | 12~18 |
| ear and labyrinth disorders | ototoxicity | 7 | 10.86(5.14, 22.97) | 10.81(5.13, 22.77) | 61.31 | 3.41(2.4) | 10.65(5.69) | 12~18 |
| cardiac disorders | cardiac tamponade | 4 | 22.41(8.26, 60.81) | 22.34(8.22, 60.7) | 78.82 | 4.43(3.14) | 21.63(9.38) | 12~18 |
| general disorders and administration site conditions | brain death | 4 | 8.28(3.09, 22.24) | 8.26(3.1, 22.01) | 25.22 | 3.03(1.75) | 8.17(3.58) | 12~18 |
| investigations | drug level decreased | 3 | 49.52(15.29, 160.44) | 49.41(15.24, 160.16) | 132.15 | 5.52(4.04) | 45.96(17.19) | 12~18 |
| nervous system disorders | unresponsive to stimuli | 5 | 14.01(5.77, 34.04) | 13.97(5.78, 33.75) | 58.92 | 3.77(2.6) | 13.69(6.51) | 12~18 |
| infections and infestations | cardiac valve vegetation | 4 | 2577.15(287.85, 23073.32) | 2569.57(286.09, 23079.36) | 2054.07 | 9.01(7.29) | 514.71(82.23) | 12~18 |
| general disorders and administration site conditions | drug resistance | 7 | 48.6(22.5, 104.96) | 48.35(22.51, 103.84) | 301.92 | 5.49(4.45) | 45.04(23.65) | 12~18 |
| investigations | blood creatine increased | 3 | 10.91(3.48, 34.19) | 10.89(3.49, 33.94) | 26.5 | 3.42(1.99) | 10.72(4.12) | 12~18 |
| gastrointestinal disorders | enterocolitis | 35 | 3.57(2.55, 5) | 3.5(2.51, 4.88) | 62.71 | 1.8(1.32) | 3.49(2.63) | 12~18 |
| infections and infestations | osteomyelitis | 9 | 5.09(2.64, 9.83) | 5.06(2.65, 9.66) | 29.15 | 2.33(1.43) | 5.03(2.9) | 12~18 |
| infections and infestations | clostridium difficile colitis | 6 | 47.79(20.82, 109.71) | 47.58(20.89, 108.38) | 254.79 | 5.47(4.36) | 44.37(22.14) | 12~18 |
| general disorders and administration site conditions | disease recurrence | 5 | 6.28(2.6, 15.18) | 6.26(2.59, 15.12) | 21.9 | 2.63(1.47) | 6.21(2.97) | 12~18 |
| investigations | pulse absent | 9 | 42.48(21.59, 83.55) | 42.2(21.67, 82.17) | 339.76 | 5.31(4.38) | 39.66(22.52) | 12~18 |
| investigations | urine output decreased | 9 | 23.37(11.99, 45.54) | 23.22(11.92, 45.21) | 184.74 | 4.49(3.57) | 22.44(12.84) | 12~18 |
| hepatobiliary disorders | jaundice | 4 | 25.77(9.47, 70.1) | 25.7(9.46, 69.83) | 91.3 | 4.63(3.33) | 24.75(10.71) | 12~18 |
| blood and lymphatic system disorders | granulocytopenia | 12 | 3888.66(869.48, 17391.68) | 3854.36(869.01, 17095.32) | 6604.14 | 9.11(8.03) | 551.48(157.47) | 12~18 |
| pregnancy, puerperium and perinatal conditions | premature baby | 37 | 3.2(2.3, 4.43) | 3.14(2.29, 4.3) | 54.03 | 1.64(1.18) | 3.13(2.38) | 12~18 |
| infections and infestations | staphylococcal sepsis | 3 | 5.66(1.81, 17.66) | 5.65(1.81, 17.61) | 11.39 | 2.49(1.06) | 5.61(2.17) | 12~18 |
| skin and subcutaneous tissue disorders | skin erosion | 4 | 5.28(1.97, 14.14) | 5.27(1.98, 14.04) | 13.72 | 2.39(1.11) | 5.23(2.29) | 12~18 |
| investigations | forced expiratory volume abnormal | 7 | 23.66(11.11, 50.4) | 23.54(11.18, 49.58) | 145.79 | 4.51(3.49) | 22.75(12.08) | 12~18 |
| pregnancy, puerperium and perinatal conditions | low birth weight baby | 17 | 6.6(4.08, 10.67) | 6.53(4.08, 10.45) | 78.93 | 2.69(2.02) | 6.47(4.33) | 12~18 |
| infections and infestations | staphylococcal infection | 3 | 9.8(3.13, 30.69) | 9.78(3.14, 30.48) | 23.31 | 3.27(1.84) | 9.65(3.71) | 12~18 |
| cardiac disorders | cardiopulmonary failure | 35 | 6.19(4.42, 8.67) | 6.06(4.34, 8.46) | 146.97 | 2.59(2.11) | 6.01(4.53) | 12~18 |
| injury, poisoning and procedural complications | product label confusion | 6 | 4.71(2.1, 10.52) | 4.69(2.1, 10.48) | 17.3 | 2.22(1.15) | 4.66(2.38) | 12~18 |
| injury, poisoning and procedural complications | product packaging confusion | 3 | 8.08(2.58, 25.26) | 8.06(2.59, 25.12) | 18.34 | 3(1.57) | 7.98(3.07) | 12~18 |
| infections and infestations | arthritis bacterial | 22 | 4.72(3.09, 7.2) | 4.66(3.09, 7.03) | 63 | 2.21(1.62) | 4.63(3.25) | 12~18 |
| hepatobiliary disorders | liver injury | 8 | 5.21(2.59, 10.47) | 5.19(2.61, 10.31) | 26.84 | 2.37(1.42) | 5.15(2.87) | 12~18 |
| respiratory, thoracic and mediastinal disorders | hypoventilation | 7 | 14.03(6.62, 29.73) | 13.97(6.63, 29.42) | 82.49 | 3.77(2.76) | 13.69(7.3) | 12~18 |
| gastrointestinal disorders | megacolon | 9 | 13.85(7.14, 26.86) | 13.77(7.07, 26.81) | 104.36 | 3.75(2.85) | 13.5(7.75) | 12~18 |
| infections and infestations | staphylococcal bacteraemia | 3 | 22.99(7.26, 72.82) | 22.94(7.22, 72.91) | 60.79 | 4.47(3.02) | 22.19(8.46) | 12~18 |
| eye disorders | retinal artery occlusion | 5 | 4.85(2.01, 11.71) | 4.84(2, 11.69) | 15.12 | 2.27(1.1) | 4.81(2.3) | 12~18 |
| gastrointestinal disorders | oral mucosal exfoliation | 4 | 7.02(2.62, 18.83) | 7(2.63, 18.65) | 20.36 | 2.79(1.52) | 6.94(3.04) | 12~18 |
| metabolism and nutrition disorders | hypernatraemia | 8 | 5.26(2.62, 10.58) | 5.24(2.64, 10.41) | 27.25 | 2.38(1.43) | 5.2(2.9) | 12~18 |
| gastrointestinal disorders | oral mucosa erosion | 6 | 3.99(1.79, 8.93) | 3.98(1.78, 8.89) | 13.33 | 1.99(0.91) | 3.96(2.02) | 12~18 |
| infections and infestations | bacteraemia | 5 | 4.7(1.95, 11.34) | 4.68(1.94, 11.31) | 14.39 | 2.22(1.06) | 4.66(2.23) | 12~18 |
| infections and infestations | leuconostoc infection | 5 | 9.48(3.91, 22.96) | 9.45(3.91, 22.83) | 37.23 | 3.22(2.05) | 9.32(4.45) | 12~18 |
| infections and infestations | pneumonia staphylococcal | 5 | 65.79(26.17, 165.37) | 65.55(26.09, 164.68) | 288.42 | 5.9(4.68) | 59.57(27.55) | 12~18 |
| infections and infestations | cavernous sinus thrombosis | 15 | 21.45(12.79, 35.98) | 21.22(12.75, 35.32) | 279.98 | 4.36(3.64) | 20.58(13.35) | 12~18 |
| hepatobiliary disorders | hepatomegaly | 7 | 13.78(6.5, 29.18) | 13.71(6.51, 28.87) | 80.79 | 3.75(2.73) | 13.44(7.17) | 12~18 |
| investigations | inflammatory marker increased | 3 | 35.77(11.17, 114.53) | 35.69(11.23, 113.44) | 95.83 | 5.08(3.62) | 33.86(12.79) | 12~18 |
| skin and subcutaneous tissue disorders | skin plaque | 8 | 4.66(2.32, 9.36) | 4.64(2.34, 9.21) | 22.7 | 2.21(1.26) | 4.61(2.57) | 12~18 |
| skin and subcutaneous tissue disorders | hypersensitivity vasculitis | 5 | 17.42(7.16, 42.42) | 17.36(7.19, 41.94) | 75.09 | 4.08(2.91) | 16.93(8.04) | 12~18 |
| injury, poisoning and procedural complications | exposure during pregnancy | 3 | 5.71(1.83, 17.82) | 5.7(1.83, 17.77) | 11.53 | 2.5(1.08) | 5.66(2.18) | 12~18 |
| blood and lymphatic system disorders | neutrophilia | 3 | 13.7(4.36, 43.03) | 13.67(4.39, 42.61) | 34.5 | 3.74(2.31) | 13.4(5.14) | 12~18 |
| renal and urinary disorders | azotaemia | 3 | 11.92(3.8, 37.39) | 11.9(3.82, 37.09) | 29.4 | 3.55(2.12) | 11.7(4.49) | 12~18 |
| gastrointestinal disorders | noninfectious peritonitis | 5 | 153.51(57.8, 407.71) | 152.95(57.4, 407.53) | 609.65 | 6.95(5.67) | 123.73(54.64) | 12~18 |
| infections and infestations | infectious pleural effusion | 5 | 32.56(13.24, 80.08) | 32.44(13.17, 79.92) | 145.07 | 4.95(3.76) | 30.93(14.57) | 12~18 |
| eye disorders | retinal vein occlusion | 4 | 73.63(26.13, 207.45) | 73.42(25.98, 207.47) | 256.43 | 6.04(4.7) | 65.99(27.74) | 12~18 |
| gastrointestinal disorders | peritoneal cloudy effluent | 4 | 78.09(27.63, 220.73) | 77.87(27.56, 220.05) | 270.71 | 6.12(4.77) | 69.56(29.16) | 12~18 |
| eye disorders | glaucoma | 12 | 14.26(8.03, 25.33) | 14.14(8.01, 24.96) | 143.51 | 3.79(3) | 13.86(8.57) | 12~18 |
| eye disorders | retinal vasculitis | 3 | 10.79(3.44, 33.81) | 10.77(3.46, 33.57) | 26.15 | 3.41(1.98) | 10.61(4.08) | 12~18 |
| injury, poisoning and procedural complications | transcription medication error | 4 | 61.36(21.97, 171.35) | 61.18(22.08, 169.53) | 216.21 | 5.81(4.47) | 55.95(23.69) | 12~18 |
| blood and lymphatic system disorders | leukaemoid reaction | 3 | 643.81(129.82, 3192.7) | 642.39(128.77, 3204.77) | 960.6 | 8.33(6.56) | 321.7(84.25) | 12~18 |
| metabolism and nutrition disorders | metabolic acidosis | 3 | 44.91(13.92, 144.95) | 44.82(13.83, 145.28) | 120.15 | 5.39(3.92) | 41.96(15.74) | 12~18 |
| infections and infestations | bone abscess | 3 | 5.85(1.87, 18.26) | 5.84(1.87, 18.2) | 11.93 | 2.54(1.11) | 5.8(2.24) | 12~18 |
| infections and infestations | intervertebral discitis | 3 | 55.18(16.95, 179.64) | 55.06(16.99, 178.47) | 146.68 | 5.67(4.18) | 50.79(18.92) | 12~18 |
| injury, poisoning and procedural complications | vascular pseudoaneurysm | 3 | 18.75(5.94, 59.17) | 18.71(5.89, 59.47) | 48.87 | 4.19(2.74) | 18.21(6.96) | 12~18 |
| eye disorders | vitritis | 8 | 12.95(6.42, 26.13) | 12.88(6.36, 26.08) | 85.98 | 3.66(2.7) | 12.65(7.03) | 12~18 |
| infections and infestations | infective aneurysm | 506 | 27.45(25.09, 30.03) | 26.26(24.28, 28.4) | 12125.09 | 4.69(4.56) | 25.87(23.99) | unknow |
| infections and infestations | acinetobacter infection | 223 | 121.35(105.79, 139.19) | 118.97(103.72, 136.47) | 24332.23 | 6.79(6.6) | 111.02(98.98) | unknow |
| hepatobiliary disorders | hypertransaminasaemia | 298 | 165.41(146.65, 186.58) | 161.07(143.2, 181.17) | 43187.39 | 7.2(7.02) | 146.8(132.73) | unknow |
| skin and subcutaneous tissue disorders | henoch-schonlein purpura | 98 | 48(39.23, 58.73) | 47.59(39.12, 57.89) | 4345.39 | 5.53(5.24) | 46.28(39.1) | unknow |
| infections and infestations | septic embolus | 71 | 4.99(3.95, 6.3) | 4.96(3.92, 6.28) | 224.35 | 2.31(1.97) | 4.95(4.07) | unknow |
| skin and subcutaneous tissue disorders | nikolsky's sign | 27 | 14.58(9.98, 21.31) | 14.55(10.03, 21.12) | 337.8 | 3.85(3.31) | 14.43(10.51) | unknow |
| injury, poisoning and procedural complications | foetal exposure during delivery | 7 | 7.24(3.45, 15.22) | 7.24(3.44, 15.25) | 37.46 | 2.85(1.85) | 7.21(3.87) | unknow |
| cardiac disorders | pulseless electrical activity | 11 | 8.57(4.74, 15.5) | 8.56(4.75, 15.41) | 73.07 | 3.09(2.27) | 8.52(5.19) | unknow |
| respiratory, thoracic and mediastinal disorders | infantile apnoea | 20 | 67.85(43.37, 106.15) | 67.73(43.15, 106.31) | 1262.97 | 6.02(5.39) | 65.09(44.76) | unknow |
| injury, poisoning and procedural complications | dose calculation error | 20 | 4.61(2.97, 7.15) | 4.6(2.99, 7.08) | 56.21 | 2.2(1.58) | 4.59(3.18) | unknow |
| infections and infestations | human herpesvirus 6 infection | 17 | 6.99(4.34, 11.26) | 6.98(4.36, 11.17) | 86.73 | 2.8(2.13) | 6.95(4.67) | unknow |
| investigations | clostridium test positive | 11 | 5.85(3.23, 10.58) | 5.84(3.24, 10.51) | 44.02 | 2.54(1.72) | 5.83(3.55) | unknow |
| blood and lymphatic system disorders | haemorrhagic disorder | 92 | 9.73(7.92, 11.95) | 9.66(7.94, 11.75) | 710.67 | 3.26(2.97) | 9.61(8.09) | unknow |
| infections and infestations | fungaemia | 94 | 4.61(3.76, 5.65) | 4.58(3.76, 5.57) | 263.05 | 2.19(1.9) | 4.57(3.86) | unknow |
| eye disorders | pupillary reflex impaired | 602 | 14.04(12.93, 15.24) | 13.34(12.33, 14.43) | 6844.36 | 3.73(3.61) | 13.24(12.36) | unknow |
| eye disorders | retinal ischaemia | 34 | 6.01(4.29, 8.43) | 6(4.3, 8.37) | 141.19 | 2.58(2.1) | 5.98(4.51) | unknow |
| eye disorders | retinal vascular occlusion | 8 | 4.29(2.14, 8.6) | 4.29(2.16, 8.52) | 20.15 | 2.1(1.15) | 4.28(2.4) | unknow |
| immune system disorders | type iii immune complex mediated reaction | 40 | 18.97(13.88, 25.92) | 18.91(13.82, 25.88) | 670.82 | 4.23(3.78) | 18.7(14.4) | unknow |
| vascular disorders | haemorrhagic vasculitis | 16 | 4.18(2.56, 6.83) | 4.18(2.56, 6.82) | 38.58 | 2.06(1.37) | 4.17(2.76) | unknow |
| eye disorders | conjunctival hyperaemia | 33 | 23.57(16.71, 33.25) | 23.5(16.51, 33.44) | 701.05 | 4.54(4.05) | 23.19(17.38) | unknow |
| eye disorders | corneal oedema | 6 | 3.96(1.78, 8.83) | 3.96(1.77, 8.84) | 13.25 | 1.98(0.91) | 3.95(2.02) | unknow |
| eye disorders | iridocyclitis | 19 | 894.97(511.81, 1564.97) | 893.47(516.11, 1546.75) | 10978.27 | 9.18(8.44) | 579.45(363.03) | unknow |
| skin and subcutaneous tissue disorders | papule | 26 | 51.74(35, 76.47) | 51.62(34.88, 76.39) | 1251.41 | 5.65(5.09) | 50.08(36.11) | unknow |
| renal and urinary disorders | renal tubular injury | 5 | 7.71(3.2, 18.56) | 7.71(3.19, 18.63) | 29.04 | 2.94(1.78) | 7.67(3.68) | unknow |
| general disorders and administration site conditions | multiple-drug resistance | 3 | 9.61(3.09, 29.9) | 9.61(3.08, 29.95) | 23 | 3.26(1.84) | 9.56(3.7) | unknow |
| respiratory, thoracic and mediastinal disorders | pulmonary haemorrhage | 4 | 6.37(2.39, 17) | 6.37(2.39, 16.97) | 18.03 | 2.67(1.4) | 6.35(2.79) | unknow |
| injury, poisoning and procedural complications | transplant failure | 8 | 16.05(8, 32.21) | 16.04(7.92, 32.48) | 111.72 | 3.99(3.04) | 15.89(8.87) | unknow |
| infections and infestations | pseudomonas infection | 21 | 4.31(2.81, 6.61) | 4.3(2.79, 6.62) | 53.11 | 2.1(1.5) | 4.29(3) | unknow |
| infections and infestations | meningitis candida | 15 | 5.07(3.05, 8.42) | 5.07(3.05, 8.44) | 48.8 | 2.34(1.63) | 5.05(3.31) | unknow |
| nervous system disorders | hydrocephalus | 19 | 65.53(41.41, 103.69) | 65.42(41.68, 102.68) | 1159.24 | 5.98(5.33) | 62.96(42.88) | unknow |
| investigations | pulse abnormal | 76 | 8.49(6.77, 10.64) | 8.44(6.8, 10.47) | 496.07 | 3.07(2.75) | 8.4(6.95) | unknow |
| skin and subcutaneous tissue disorders | skin warm | 11 | 14.6(8.06, 26.44) | 14.59(8.1, 26.27) | 138.01 | 3.85(3.03) | 14.47(8.8) | unknow |
| cardiac disorders | kounis syndrome | 14 | 10.91(6.45, 18.46) | 10.9(6.42, 18.5) | 125.07 | 3.44(2.7) | 10.83(6.98) | unknow |
| blood and lymphatic system disorders | splenic infarction | 31 | 21.48(15.07, 30.63) | 21.43(15.06, 30.5) | 596.05 | 4.4(3.9) | 21.17(15.73) | unknow |
| skin and subcutaneous tissue disorders | subcutaneous emphysema | 8 | 11.12(5.55, 22.29) | 11.11(5.59, 22.06) | 73.12 | 3.47(2.52) | 11.04(6.17) | unknow |
| infections and infestations | sepsis neonatal | 13 | 20.25(11.71, 34.99) | 20.22(11.68, 35) | 234.67 | 4.32(3.56) | 19.99(12.65) | unknow |
| blood and lymphatic system disorders | thrombocytopenia neonatal | 23 | 10.24(6.79, 15.44) | 10.22(6.77, 15.42) | 190.23 | 3.35(2.77) | 10.17(7.21) | unknow |
| nervous system disorders | myasthenia gravis crisis | 18 | 3.58(2.25, 5.68) | 3.57(2.23, 5.71) | 33.32 | 1.84(1.19) | 3.57(2.42) | unknow |
| general disorders and administration site conditions | systemic inflammatory response syndrome | 6 | 147.47(63.96, 340.03) | 147.39(63.45, 342.37) | 800.68 | 7.08(5.96) | 135.36(67.28) | unknow |
| infections and infestations | corynebacterium infection | 5 | 9.21(3.82, 22.18) | 9.21(3.81, 22.25) | 36.37 | 3.2(2.04) | 9.16(4.39) | unknow |
| injury, poisoning and procedural complications | product prescribing error | 13 | 7.05(4.09, 12.17) | 7.05(4.07, 12.2) | 67.19 | 2.81(2.05) | 7.02(4.45) | unknow |
| injury, poisoning and procedural complications | product appearance confusion | 35 | 5.59(4.01, 7.79) | 5.57(3.99, 7.77) | 130.92 | 2.47(2) | 5.56(4.21) | unknow |
| hepatobiliary disorders | venoocclusive liver disease | 20 | 10.31(6.64, 16.01) | 10.29(6.69, 15.84) | 166.79 | 3.36(2.74) | 10.24(7.08) | unknow |
| injury, poisoning and procedural complications | therapeutic drug monitoring analysis incorrectly performed | 5 | 9.2(3.82, 22.16) | 9.19(3.8, 22.2) | 36.32 | 3.19(2.04) | 9.15(4.38) | unknow |
| infections and infestations | mycobacterial infection | 4 | 28.88(10.75, 77.64) | 28.87(10.84, 76.92) | 105.78 | 4.83(3.55) | 28.39(12.41) | unknow |
| injury, poisoning and procedural complications | drug monitoring procedure not performed | 48 | 61.55(46.11, 82.15) | 61.29(45.68, 82.24) | 2744.64 | 5.89(5.47) | 59.12(46.44) | unknow |
| infections and infestations | escherichia bacteraemia | 93 | 26.79(21.81, 32.91) | 26.58(21.85, 32.34) | 2253.61 | 4.71(4.41) | 26.17(22.03) | unknow |
| injury, poisoning and procedural complications | product name confusion | 3 | 109.75(34.1, 353.22) | 109.72(33.85, 355.64) | 303 | 6.69(5.21) | 102.93(38.71) | unknow |
| immune system disorders | type i hypersensitivity | 7 | 6.17(2.94, 12.97) | 6.17(2.93, 12.99) | 30.22 | 2.62(1.62) | 6.15(3.31) | unknow |
| injury, poisoning and procedural complications | product preparation issue | 10 | 35.89(19.18, 67.17) | 35.86(19.15, 67.14) | 331.64 | 5.13(4.27) | 35.11(20.78) | unknow |
| infections and infestations | fungal sepsis | 93 | 1498.4(1131.36, 1984.52) | 1486.06(1129.45, 1955.27) | 72530.68 | 9.61(9.25) | 781.42(617.7) | unknow |
| blood and lymphatic system disorders | immune thrombocytopenia | 20 | 15.75(10.13, 24.47) | 15.72(10.21, 24.19) | 273.09 | 3.96(3.34) | 15.58(10.78) | unknow |
| investigations | apgar score low | 10 | 3.96(2.13, 7.38) | 3.96(2.11, 7.41) | 22.1 | 1.98(1.13) | 3.95(2.35) | unknow |
| blood and lymphatic system disorders | pseudolymphoma | 41 | 3.77(2.77, 5.13) | 3.76(2.75, 5.14) | 83.03 | 1.91(1.47) | 3.76(2.9) | unknow |
| gastrointestinal disorders | dysbiosis | 83 | 3.19(2.57, 3.96) | 3.17(2.56, 3.93) | 123.6 | 1.66(1.35) | 3.17(2.64) | unknow |
| respiratory, thoracic and mediastinal disorders | neonatal respiratory distress | 28 | 3.27(2.26, 4.74) | 3.26(2.25, 4.73) | 43.92 | 1.7(1.18) | 3.26(2.39) | unknow |
| skin and subcutaneous tissue disorders | palmar erythema | 59 | 8.09(6.26, 10.46) | 8.06(6.25, 10.4) | 363.04 | 3(2.64) | 8.02(6.47) | unknow |
| nervous system disorders | toxic encephalopathy | 13 | 55.78(32.09, 96.98) | 55.72(32.19, 96.46) | 675.72 | 5.75(4.98) | 53.93(33.95) | unknow |
| respiratory, thoracic and mediastinal disorders | bronchopulmonary dysplasia | 33 | 11.82(8.39, 16.66) | 11.79(8.45, 16.45) | 323.69 | 3.55(3.06) | 11.72(8.79) | unknow |
| eye disorders | retinopathy of prematurity | 18 | 21.47(13.48, 34.19) | 21.44(13.39, 34.32) | 346.22 | 4.4(3.75) | 21.17(14.34) | unknow |
| injury, poisoning and procedural complications | intercepted product dispensing error | 65 | 34.95(27.32, 44.72) | 34.76(27.47, 43.98) | 2087.33 | 5.09(4.74) | 34.06(27.71) | unknow |
| injury, poisoning and procedural complications | product selection error | 11 | 13.67(7.55, 24.74) | 13.65(7.58, 24.58) | 127.94 | 3.76(2.94) | 13.55(8.24) | unknow |
| respiratory, thoracic and mediastinal disorders | neonatal respiratory failure | 35 | 12.06(8.64, 16.82) | 12.02(8.61, 16.77) | 351.18 | 3.58(3.1) | 11.94(9.04) | unknow |
| nervous system disorders | pleocytosis | 4 | 6.08(2.28, 16.22) | 6.07(2.28, 16.17) | 16.89 | 2.6(1.33) | 6.05(2.66) | unknow |
| infections and infestations | fungal endocarditis | 4 | 22.4(8.35, 60.09) | 22.39(8.4, 59.66) | 80.65 | 4.47(3.19) | 22.11(9.68) | unknow |
| injury, poisoning and procedural complications | product communication issue | 4 | 18.04(6.73, 48.34) | 18.04(6.77, 48.07) | 63.67 | 4.16(2.88) | 17.85(7.83) | unknow |
| skin and subcutaneous tissue disorders | cutaneous vasculitis | 4 | 235.21(82.49, 670.68) | 235.12(83.2, 664.41) | 815.94 | 7.69(6.33) | 205.86(85.66) | unknow |
| hepatobiliary disorders | mixed liver injury | 47 | 2353.89(1507.65, 3675.15) | 2344.1(1493.47, 3679.22) | 45407.65 | 9.92(9.4) | 967.53(666.45) | unknow |
| respiratory, thoracic and mediastinal disorders | pulmonary cavitation | 21 | 3.71(2.42, 5.7) | 3.71(2.41, 5.71) | 41.44 | 1.89(1.28) | 3.7(2.59) | unknow |
| infections and infestations | septic pulmonary embolism | 189 | 3.91(3.39, 4.52) | 3.86(3.37, 4.43) | 402 | 1.95(1.74) | 3.86(3.42) | unknow |
| nervous system disorders | intraventricular haemorrhage neonatal | 48 | 8.05(6.06, 10.69) | 8.02(6.1, 10.55) | 293.58 | 3(2.59) | 7.98(6.29) | unknow |
| investigations | procalcitonin increased | 6 | 4.35(1.95, 9.69) | 4.35(1.95, 9.72) | 15.42 | 2.12(1.05) | 4.34(2.22) | unknow |
| nervous system disorders | neurodevelopmental delay | 39 | 3.55(2.59, 4.87) | 3.55(2.59, 4.86) | 71.18 | 1.82(1.38) | 3.54(2.72) | unknow |

**Abbreviations:** SOC = system organ classe , ROR = Reporting Odds Ratio, PRR = Proportional Reporting Ratio,EBGM = Empirical Bayes Geometric Mean ,IC=Information Component, PT = preferred term
