# Supplementary material for: Evaluating adverse reaction signals of vancomycin in pediatric patients: A FAERS database analysis
Source: Medicine (Baltimore). 2026 Jun 5;105(23):e49064. doi: 10.1097/MD.0000000000049064 (PMC13246103; doi:10.1097/MD.0000000000049064)
Supplement: Supplementary file 8 [file medi-105-e49064-s009.docx]

**Table S9:**Signal Strength of Adverse Events Associated with Vancomycin at the PT Level by Weight.

| **SOC** | **PT** | **Case Reports** | **ROR(95% CI)** | **PRR(95% CI)** | **chisq** | **IC(IC025)** | **EBGM(EBGM05)** | **group** |
| --- | --- | --- | --- | --- | --- | --- | --- | --- |
| renal and urinary disorders | acute kidney injury | 23 | 33.24(21.51, 51.36) | 32.19(20.91, 49.54) | 632.76 | 4.88(4.26) | 29.36(20.4) | <10 |
| skin and subcutaneous tissue disorders | drug reaction with eosinophilia and systemic symptoms | 3 | 74.91(21.3, 263.46) | 74.6(21.28, 261.53) | 177 | 5.93(4.35) | 60.8(21.23) | <10 |
| investigations | blood creatinine increased | 7 | 13.93(6.51, 29.78) | 13.8(6.55, 29.06) | 79.75 | 3.73(2.7) | 13.27(7.03) | <10 |
| renal and urinary disorders | nephropathy toxic | 10 | 99.35(48.78, 202.37) | 97.96(48.37, 198.38) | 736.63 | 6.24(5.28) | 75.41(41.58) | <10 |
| investigations | drug level increased | 3 | 9.93(3.14, 31.41) | 9.9(3.18, 30.86) | 23.29 | 3.27(1.82) | 9.63(3.68) | <10 |
| immune system disorders | anaphylactic reaction | 7 | 26.88(12.39, 58.3) | 26.62(12.39, 57.17) | 159.54 | 4.62(3.58) | 24.67(12.91) | <10 |
| renal and urinary disorders | renal impairment | 3 | 6.16(1.96, 19.35) | 6.14(1.97, 19.14) | 12.67 | 2.6(1.16) | 6.04(2.32) | <10 |
| blood and lymphatic system disorders | leukopenia | 7 | 23.31(10.79, 50.37) | 23.09(10.75, 49.59) | 138.13 | 4.43(3.39) | 21.62(11.35) | <10 |
| blood and lymphatic system disorders | eosinophilia | 3 | 17.7(5.53, 56.72) | 17.63(5.55, 56.04) | 44.64 | 4.07(2.6) | 16.77(6.33) | <10 |
| infections and infestations | pathogen resistance | 5 | 81.38(30.46, 217.46) | 80.81(30.33, 215.31) | 315.34 | 6.02(4.73) | 64.85(28.5) | <10 |
| investigations | drug level above therapeutic | 5 | 65.11(24.85, 170.56) | 64.65(24.74, 168.91) | 261.15 | 5.76(4.49) | 54.04(24.14) | <10 |
| general disorders and administration site conditions | condition aggravated | 11 | 5.79(3.18, 10.56) | 5.72(3.18, 10.3) | 42.18 | 2.49(1.66) | 5.63(3.41) | <10 |
| renal and urinary disorders | renal tubular disorder | 6 | 45.48(19.3, 107.21) | 45.11(19.42, 104.78) | 227.13 | 5.31(4.17) | 39.7(19.38) | <10 |
| investigations | c-reactive protein increased | 11 | 25.79(13.9, 47.85) | 25.4(13.83, 46.63) | 239.2 | 4.56(3.71) | 23.62(14.08) | <10 |
| renal and urinary disorders | renal failure | 5 | 5.19(2.14, 12.6) | 5.16(2.14, 12.47) | 16.55 | 2.35(1.18) | 5.1(2.43) | <10 |
| investigations | white blood cell count increased | 3 | 7.55(2.4, 23.76) | 7.52(2.41, 23.44) | 16.58 | 2.88(1.44) | 7.37(2.82) | <10 |
| general disorders and administration site conditions | drug ineffective | 27 | 6.7(4.55, 9.88) | 6.48(4.47, 9.4) | 123.52 | 2.67(2.12) | 6.38(4.61) | <10 |
| general disorders and administration site conditions | drug interaction | 6 | 7.24(3.21, 16.31) | 7.18(3.21, 16.04) | 31.28 | 2.82(1.73) | 7.05(3.57) | <10 |
| metabolism and nutrition disorders | electrolyte imbalance | 4 | 21.31(7.73, 58.77) | 21.2(7.8, 57.6) | 72.26 | 4.32(3) | 19.95(8.54) | <10 |
| renal and urinary disorders | nephrocalcinosis | 4 | 16.25(5.94, 44.48) | 16.16(5.95, 43.91) | 54.21 | 3.95(2.64) | 15.44(6.65) | <10 |
| skin and subcutaneous tissue disorders | erythema | 6 | 5.99(2.66, 13.48) | 5.95(2.66, 13.29) | 24.29 | 2.55(1.47) | 5.86(2.97) | <10 |
| injury, poisoning and procedural complications | accidental overdose | 8 | 6.48(3.21, 13.1) | 6.42(3.17, 13) | 35.94 | 2.66(1.7) | 6.31(3.5) | <10 |
| vascular disorders | flushing | 7 | 20.77(9.64, 44.75) | 20.57(9.58, 44.18) | 122.6 | 4.28(3.24) | 19.4(10.21) | <10 |
| cardiac disorders | tachycardia | 8 | 5.83(2.88, 11.77) | 5.77(2.91, 11.46) | 31.07 | 2.51(1.55) | 5.69(3.16) | <10 |
| skin and subcutaneous tissue disorders | toxic epidermal necrolysis | 3 | 44.26(13.22, 148.23) | 44.08(13.34, 145.71) | 111.16 | 5.28(3.76) | 38.91(14.15) | <10 |
| skin and subcutaneous tissue disorders | skin necrosis | 4 | 59.1(20.31, 171.96) | 58.77(20.39, 169.36) | 192.24 | 5.64(4.26) | 49.89(20.41) | <10 |
| infections and infestations | septic shock | 5 | 13.34(5.44, 32.72) | 13.25(5.48, 32.01) | 54.42 | 3.67(2.49) | 12.77(6.02) | <10 |
| respiratory, thoracic and mediastinal disorders | aspiration | 4 | 8.9(3.29, 24.1) | 8.86(3.26, 24.07) | 27.15 | 3.11(1.82) | 8.65(3.76) | <10 |
| injury, poisoning and procedural complications | toxicity to various agents | 8 | 6.53(3.23, 13.2) | 6.47(3.19, 13.1) | 36.3 | 2.67(1.71) | 6.36(3.53) | <10 |
| infections and infestations | systemic candida | 10 | 93.68(46.21, 189.91) | 92.36(45.61, 187.03) | 703.01 | 6.17(5.22) | 72.06(39.89) | <10 |
| vascular disorders | hypotension | 13 | 7.51(4.31, 13.07) | 7.39(4.27, 12.79) | 70.35 | 2.86(2.08) | 7.24(4.55) | <10 |
| vascular disorders | neonatal hypotension | 3 | 5.41(1.72, 16.96) | 5.39(1.73, 16.8) | 10.55 | 2.41(0.98) | 5.32(2.04) | <10 |
| renal and urinary disorders | renal failure neonatal | 3 | 9.27(2.94, 29.28) | 9.24(2.96, 28.8) | 21.43 | 3.17(1.73) | 9.01(3.44) | <10 |
| skin and subcutaneous tissue disorders | pruritus | 4 | 7.1(2.63, 19.17) | 7.07(2.65, 18.84) | 20.4 | 2.79(1.51) | 6.94(3.02) | <10 |
| respiratory, thoracic and mediastinal disorders | hypoxia | 6 | 4.93(2.19, 11.08) | 4.9(2.19, 10.94) | 18.37 | 2.27(1.19) | 4.84(2.46) | <10 |
| skin and subcutaneous tissue disorders | dermatitis | 3 | 26.32(8.1, 85.56) | 26.21(8.09, 84.96) | 67.3 | 4.6(3.12) | 24.32(9.07) | <10 |
| renal and urinary disorders | neonatal anuria | 3 | 19.09(5.94, 61.32) | 19.02(5.98, 60.45) | 48.37 | 4.17(2.7) | 18.01(6.79) | <10 |
| respiratory, thoracic and mediastinal disorders | pulmonary necrosis | 3 | 486.95(81.24, 2918.81) | 484.88(81.48, 2885.64) | 579.48 | 7.6(5.78) | 194.55(43.48) | <10 |
| general disorders and administration site conditions | drug chemical incompatibility | 4 | 1300.41(145.16, 11649.68) | 1293.02(143.96, 11613.64) | 1032.84 | 8.02(6.3) | 259.4(41.42) | <10 |
| general disorders and administration site conditions | generalised oedema | 3 | 11.45(3.61, 36.31) | 11.41(3.59, 36.27) | 27.53 | 3.47(2.02) | 11.05(4.21) | <10 |
| renal and urinary disorders | oliguria | 3 | 8.46(2.68, 26.7) | 8.43(2.7, 26.27) | 19.16 | 3.04(1.6) | 8.24(3.15) | <10 |
| respiratory, thoracic and mediastinal disorders | pleural effusion | 3 | 5.38(1.71, 16.87) | 5.36(1.72, 16.71) | 10.47 | 2.4(0.97) | 5.29(2.03) | <10 |
| general disorders and administration site conditions | treatment failure | 3 | 14.75(4.63, 47.03) | 14.69(4.62, 46.69) | 36.63 | 3.82(2.36) | 14.1(5.34) | <10 |
| vascular disorders | haemodynamic instability | 5 | 21.99(8.86, 54.56) | 21.84(8.87, 53.8) | 93.18 | 4.36(3.16) | 20.52(9.59) | <10 |
| infections and infestations | candida infection | 9 | 49.94(24.67, 101.1) | 49.31(24.35, 99.86) | 369.71 | 5.42(4.46) | 42.92(23.79) | <10 |
| respiratory, thoracic and mediastinal disorders | bronchospasm | 3 | 11.32(3.57, 35.88) | 11.28(3.55, 35.85) | 27.16 | 3.45(2) | 10.93(4.16) | <10 |
| injury, poisoning and procedural complications | wrong product administered | 5 | 39.7(15.64, 100.75) | 39.42(15.69, 99.03) | 166.92 | 5.14(3.91) | 35.25(16.17) | <10 |
| infections and infestations | endocarditis | 3 | 51.25(15.13, 173.6) | 51.04(15.14, 172.05) | 127.12 | 5.47(3.93) | 44.22(15.93) | <10 |
| hepatobiliary disorders | liver disorder | 3 | 8.25(2.62, 26.01) | 8.22(2.64, 25.62) | 18.56 | 3.01(1.57) | 8.04(3.08) | <10 |
| infections and infestations | neonatal candida infection | 5 | 162.78(55.49, 477.46) | 161.63(55, 475) | 532.15 | 6.76(5.4) | 108.09(43.93) | <10 |
| metabolism and nutrition disorders | acidosis | 4 | 7.73(2.86, 20.91) | 7.7(2.89, 20.52) | 22.78 | 2.91(1.63) | 7.54(3.28) | <10 |
| nervous system disorders | pleocytosis | 4 | 433.46(96.83, 1940.35) | 431.01(97.18, 1911.67) | 735.47 | 7.53(5.92) | 185.29(52.87) | <10 |
| gastrointestinal disorders | necrotising enterocolitis neonatal | 3 | 6.81(2.16, 21.41) | 6.78(2.18, 21.13) | 14.49 | 2.74(1.3) | 6.66(2.55) | <10 |
| infections and infestations | cns ventriculitis | 3 | 194.78(46.46, 816.61) | 193.95(46.38, 811.11) | 359.93 | 6.93(5.22) | 121.6(36.65) | <10 |
| investigations | procalcitonin increased | 6 | 177.83(65.58, 482.18) | 176.32(64.89, 479.1) | 676.85 | 6.84(5.57) | 114.44(49.67) | <10 |
| nervous system disorders | neurodevelopmental delay | 3 | 973.91(101.18, 9374.51) | 969.77(101.8, 9237.8) | 725.84 | 7.93(6.03) | 243.19(36.57) | <10 |
| nervous system disorders | haemorrhage intracranial | 3 | 10.94(3.45, 34.65) | 10.9(3.43, 34.65) | 26.1 | 3.4(1.95) | 10.57(4.03) | <10 |
| renal and urinary disorders | acute kidney injury | 92 | 39.42(31.69, 49.04) | 36.64(30.12, 44.57) | 3005.18 | 5.11(4.8) | 34.51(28.75) | 10~45 |
| skin and subcutaneous tissue disorders | drug reaction with eosinophilia and systemic symptoms | 17 | 34.56(21.12, 56.55) | 34.11(20.9, 55.68) | 516.1 | 5.01(4.32) | 32.26(21.37) | 10~45 |
| investigations | blood creatinine increased | 8 | 4.12(2.05, 8.27) | 4.1(2.06, 8.14) | 18.61 | 2.03(1.08) | 4.07(2.27) | 10~45 |
| renal and urinary disorders | nephropathy toxic | 6 | 31.85(13.97, 72.58) | 31.7(13.92, 72.21) | 169.12 | 4.91(3.81) | 30.1(15.11) | 10~45 |
| investigations | drug level increased | 12 | 15.63(8.79, 27.81) | 15.5(8.78, 27.36) | 158.56 | 3.92(3.12) | 15.12(9.34) | 10~45 |
| immune system disorders | anaphylactic reaction | 9 | 5.46(2.83, 10.55) | 5.43(2.84, 10.37) | 32.25 | 2.43(1.53) | 5.39(3.1) | 10~45 |
| renal and urinary disorders | renal impairment | 17 | 7.68(4.74, 12.43) | 7.59(4.74, 12.15) | 96.17 | 2.91(2.23) | 7.5(5.01) | 10~45 |
| blood and lymphatic system disorders | leukopenia | 7 | 4.99(2.37, 10.53) | 4.97(2.36, 10.47) | 22.04 | 2.3(1.3) | 4.94(2.64) | 10~45 |
| blood and lymphatic system disorders | eosinophilia | 4 | 10.6(3.94, 28.53) | 10.57(3.97, 28.16) | 34.04 | 3.38(2.1) | 10.4(4.54) | 10~45 |
| infections and infestations | pathogen resistance | 3 | 26.64(8.36, 84.88) | 26.58(8.36, 84.48) | 70.61 | 4.67(3.21) | 25.45(9.65) | 10~45 |
| investigations | drug level above therapeutic | 6 | 58.84(25.36, 136.53) | 58.57(25.21, 136.05) | 308.21 | 5.73(4.61) | 53.26(26.33) | 10~45 |
| general disorders and administration site conditions | drug interaction | 54 | 18.46(14, 24.34) | 17.72(13.47, 23.31) | 828.6 | 4.11(3.71) | 17.22(13.66) | 10~45 |
| skin and subcutaneous tissue disorders | erythema | 21 | 5.13(3.33, 7.92) | 5.06(3.29, 7.79) | 68.13 | 2.33(1.72) | 5.03(3.5) | 10~45 |
| injury, poisoning and procedural complications | toxicity to various agents | 10 | 4.34(2.33, 8.11) | 4.32(2.31, 8.09) | 25.35 | 2.1(1.24) | 4.29(2.55) | 10~45 |
| skin and subcutaneous tissue disorders | pruritus | 36 | 5.66(4.06, 7.89) | 5.53(3.96, 7.72) | 132.87 | 2.45(1.98) | 5.48(4.15) | 10~45 |
| blood and lymphatic system disorders | agranulocytosis | 4 | 5.05(1.89, 13.54) | 5.04(1.89, 13.43) | 12.85 | 2.32(1.05) | 5.01(2.19) | 10~45 |
| skin and subcutaneous tissue disorders | rash | 37 | 4.24(3.05, 5.89) | 4.15(3.03, 5.68) | 88.31 | 2.04(1.58) | 4.12(3.13) | 10~45 |
| immune system disorders | drug hypersensitivity | 12 | 5.05(2.86, 8.94) | 5.02(2.84, 8.86) | 38.32 | 2.32(1.52) | 4.98(3.09) | 10~45 |
| skin and subcutaneous tissue disorders | urticaria | 24 | 5.11(3.41, 7.67) | 5.04(3.41, 7.46) | 77.24 | 2.32(1.75) | 5(3.56) | 10~45 |
| skin and subcutaneous tissue disorders | stevens-johnson syndrome | 8 | 6.82(3.39, 13.73) | 6.79(3.42, 13.48) | 39.04 | 2.75(1.8) | 6.72(3.74) | 10~45 |
| skin and subcutaneous tissue disorders | rash erythematous | 12 | 8.45(4.76, 14.98) | 8.38(4.75, 14.79) | 76.93 | 3.05(2.25) | 8.27(5.12) | 10~45 |
| general disorders and administration site conditions | infusion site extravasation | 3 | 10.96(3.49, 34.39) | 10.94(3.51, 34.1) | 26.58 | 3.43(1.99) | 10.75(4.13) | 10~45 |
| metabolism and nutrition disorders | hypernatraemia | 3 | 7.91(2.53, 24.74) | 7.89(2.53, 24.59) | 17.81 | 2.96(1.53) | 7.8(3) | 10~45 |
| renal and urinary disorders | tubulointerstitial nephritis | 3 | 8.7(2.78, 27.24) | 8.68(2.78, 27.05) | 20.1 | 3.1(1.67) | 8.57(3.3) | 10~45 |
| general disorders and administration site conditions | localised oedema | 6 | 35.79(15.66, 81.78) | 35.62(15.64, 81.13) | 190.18 | 5.07(3.96) | 33.61(16.83) | 10~45 |
| renal and urinary disorders | renal tubular necrosis | 4 | 14(5.19, 37.81) | 13.96(5.14, 37.93) | 47.01 | 3.77(2.49) | 13.66(5.95) | 10~45 |
| skin and subcutaneous tissue disorders | rash maculo-papular | 10 | 12.19(6.5, 22.85) | 12.1(6.46, 22.66) | 99.79 | 3.57(2.7) | 11.87(7.01) | 10~45 |
| general disorders and administration site conditions | swelling face | 9 | 5.27(2.73, 10.17) | 5.24(2.74, 10.01) | 30.6 | 2.38(1.48) | 5.2(3) | 10~45 |
| skin and subcutaneous tissue disorders | rash pruritic | 5 | 4.29(1.78, 10.36) | 4.28(1.77, 10.34) | 12.48 | 2.09(0.93) | 4.25(2.03) | 10~45 |
| immune system disorders | anaphylactoid reaction | 6 | 25.15(11.09, 57.07) | 25.04(10.99, 57.04) | 132.74 | 4.59(3.49) | 24.04(12.11) | 10~45 |
| investigations | antibiotic level above therapeutic | 4 | 165.07(54.26, 502.17) | 164.55(53.84, 502.91) | 505.75 | 7(5.58) | 128.21(50.54) | 10~45 |
| blood and lymphatic system disorders | lymphadenopathy | 5 | 5.15(2.13, 12.44) | 5.13(2.12, 12.39) | 16.51 | 2.35(1.19) | 5.1(2.44) | 10~45 |
| general disorders and administration site conditions | hyperthermia | 4 | 10(3.72, 26.92) | 9.97(3.74, 26.56) | 31.75 | 3.3(2.02) | 9.82(4.29) | 10~45 |
| gastrointestinal disorders | lip erythema | 4 | 110.04(37.72, 321.04) | 109.7(38.07, 316.13) | 361.92 | 6.53(5.15) | 92.31(37.69) | 10~45 |
| eye disorders | periorbital oedema | 3 | 20.14(6.36, 63.75) | 20.09(6.32, 63.86) | 52.59 | 4.28(2.83) | 19.45(7.41) | 10~45 |
| eye disorders | eyelid oedema | 5 | 8.14(3.36, 19.71) | 8.11(3.36, 19.59) | 30.76 | 3(1.84) | 8.01(3.82) | 10~45 |
| skin and subcutaneous tissue disorders | angioedema | 9 | 11.97(6.17, 23.21) | 11.89(6.11, 23.15) | 87.99 | 3.54(2.64) | 11.67(6.7) | 10~45 |
| immune system disorders | anaphylactic shock | 4 | 5.05(1.89, 13.54) | 5.04(1.89, 13.43) | 12.85 | 2.32(1.05) | 5.01(2.19) | 10~45 |
| investigations | antibiotic level below therapeutic | 5 | 2891.04(337.51, 24764.1) | 2879.68(333.43, 24870.42) | 2398.08 | 8.91(7.33) | 480.78(79.7) | 10~45 |
| injury, poisoning and procedural complications | incorrect drug administration rate | 3 | 14.8(4.7, 46.62) | 14.77(4.74, 46.03) | 37.55 | 3.85(2.41) | 14.42(5.52) | 10~45 |
| investigations | eosinophil count increased | 4 | 11.33(4.2, 30.51) | 11.29(4.24, 30.08) | 36.81 | 3.47(2.19) | 11.09(4.84) | 10~45 |
| investigations | liver function test increased | 6 | 17.01(7.54, 38.39) | 16.94(7.58, 37.84) | 87.44 | 4.04(2.95) | 16.48(8.34) | 10~45 |
| investigations | blood electrolytes abnormal | 5 | 65.7(26.01, 165.96) | 65.45(26.05, 164.43) | 284.95 | 5.88(4.66) | 58.87(27.11) | 10~45 |
| infections and infestations | dermo-hypodermitis | 4 | 770.33(172.23, 3445.46) | 767.91(173.14, 3405.93) | 1313.01 | 8.36(6.75) | 329.68(94.13) | 10~45 |
| general disorders and administration site conditions | extravasation | 7 | 48.25(22.27, 104.54) | 47.99(22.34, 103.07) | 297.37 | 5.47(4.43) | 44.38(23.24) | 10~45 |
| skin and subcutaneous tissue disorders | vancomycin infusion reaction | 8 | 927.32(302.95, 2838.51) | 921.5(301.51, 2816.37) | 2829.27 | 8.47(7.26) | 355.04(139.23) | 10~45 |
| renal and urinary disorders | acute kidney injury | 663 | 16.78(15.51, 18.15) | 16.04(14.83, 17.35) | 9222.08 | 3.98(3.87) | 15.79(14.79) | >=45 |
| skin and subcutaneous tissue disorders | drug reaction with eosinophilia and systemic symptoms | 146 | 40.94(34.66, 48.35) | 40.53(34.65, 47.41) | 5403.81 | 5.28(5.04) | 38.94(33.88) | >=45 |
| investigations | blood creatinine increased | 245 | 12.47(10.98, 14.16) | 12.27(10.91, 13.8) | 2508.8 | 3.6(3.42) | 12.13(10.91) | >=45 |
| renal and urinary disorders | nephropathy toxic | 111 | 75.43(62.14, 91.56) | 74.84(61.52, 91.04) | 7507.33 | 6.12(5.84) | 69.54(59.13) | >=45 |
| investigations | drug level increased | 114 | 35.97(29.81, 43.39) | 35.69(29.92, 42.58) | 3707.57 | 5.11(4.84) | 34.45(29.45) | >=45 |
| immune system disorders | anaphylactic reaction | 175 | 13.7(11.79, 15.91) | 13.54(11.58, 15.84) | 2005.83 | 3.74(3.52) | 13.36(11.79) | >=45 |
| renal and urinary disorders | renal impairment | 114 | 5.78(4.81, 6.96) | 5.75(4.82, 6.86) | 444.84 | 2.52(2.25) | 5.72(4.9) | >=45 |
| blood and lymphatic system disorders | leukopenia | 64 | 4.7(3.68, 6.01) | 4.69(3.64, 6.05) | 184.79 | 2.22(1.87) | 4.67(3.8) | >=45 |
| blood and lymphatic system disorders | eosinophilia | 146 | 37.37(31.65, 44.13) | 36.99(31.62, 43.27) | 4926.43 | 5.16(4.92) | 35.67(31.04) | >=45 |
| infections and infestations | pathogen resistance | 10 | 17.39(9.31, 32.51) | 17.38(9.28, 32.54) | 151.68 | 4.1(3.23) | 17.09(10.13) | >=45 |
| investigations | drug level above therapeutic | 43 | 38.74(28.55, 52.56) | 38.62(28.22, 52.85) | 1515.46 | 5.22(4.78) | 37.18(28.8) | >=45 |
| renal and urinary disorders | renal tubular disorder | 8 | 10.98(5.47, 22.04) | 10.97(5.42, 22.21) | 71.69 | 3.44(2.49) | 10.86(6.06) | >=45 |
| renal and urinary disorders | renal failure | 106 | 3.03(2.5, 3.66) | 3.01(2.47, 3.66) | 142.26 | 1.59(1.31) | 3(2.56) | >=45 |
| skin and subcutaneous tissue disorders | erythema | 237 | 5.18(4.56, 5.89) | 5.11(4.54, 5.75) | 782.17 | 2.35(2.16) | 5.09(4.57) | >=45 |
| cardiac disorders | tachycardia | 98 | 3.64(2.98, 4.44) | 3.62(2.98, 4.4) | 185.42 | 1.85(1.57) | 3.61(3.06) | >=45 |
| skin and subcutaneous tissue disorders | toxic epidermal necrolysis | 54 | 20.36(15.54, 26.67) | 20.29(15.42, 26.7) | 970.09 | 4.31(3.93) | 19.89(15.87) | >=45 |
| skin and subcutaneous tissue disorders | skin necrosis | 13 | 11.43(6.61, 19.75) | 11.42(6.6, 19.77) | 122.19 | 3.5(2.74) | 11.3(7.15) | >=45 |
| injury, poisoning and procedural complications | toxicity to various agents | 56 | 3.25(2.5, 4.23) | 3.24(2.51, 4.18) | 86.51 | 1.69(1.32) | 3.23(2.59) | >=45 |
| vascular disorders | hypotension | 189 | 3.36(2.91, 3.88) | 3.33(2.9, 3.82) | 308.33 | 1.73(1.53) | 3.32(2.95) | >=45 |
| skin and subcutaneous tissue disorders | pruritus | 412 | 4.55(4.13, 5.02) | 4.45(4.03, 4.91) | 1103.61 | 2.15(2.01) | 4.43(4.08) | >=45 |
| respiratory, thoracic and mediastinal disorders | hypoxia | 38 | 3.15(2.29, 4.34) | 3.15(2.3, 4.31) | 55.57 | 1.65(1.2) | 3.14(2.41) | >=45 |
| general disorders and administration site conditions | generalised oedema | 15 | 4.09(2.46, 6.79) | 4.09(2.46, 6.81) | 34.81 | 2.03(1.32) | 4.07(2.66) | >=45 |
| renal and urinary disorders | oliguria | 26 | 18.08(12.26, 26.65) | 18.05(12.2, 26.71) | 411 | 4.15(3.6) | 17.73(12.81) | >=45 |
| general disorders and administration site conditions | treatment failure | 28 | 4.35(3, 6.31) | 4.34(2.99, 6.3) | 71.78 | 2.11(1.59) | 4.33(3.17) | >=45 |
| respiratory, thoracic and mediastinal disorders | bronchospasm | 27 | 6.37(4.36, 9.3) | 6.36(4.38, 9.23) | 121.22 | 2.66(2.12) | 6.33(4.61) | >=45 |
| infections and infestations | endocarditis | 6 | 4.79(2.15, 10.68) | 4.79(2.14, 10.7) | 17.88 | 2.25(1.18) | 4.77(2.44) | >=45 |
| metabolism and nutrition disorders | acidosis | 12 | 6.2(3.51, 10.94) | 6.2(3.51, 10.95) | 51.97 | 2.62(1.84) | 6.16(3.83) | >=45 |
| blood and lymphatic system disorders | agranulocytosis | 36 | 6.93(4.99, 9.62) | 6.92(4.96, 9.66) | 180.95 | 2.78(2.31) | 6.87(5.22) | >=45 |
| skin and subcutaneous tissue disorders | rash | 478 | 5.24(4.78, 5.74) | 5.09(4.61, 5.61) | 1574.77 | 2.34(2.21) | 5.07(4.7) | >=45 |
| immune system disorders | drug hypersensitivity | 220 | 7.1(6.21, 8.11) | 7(6.1, 8.03) | 1126.72 | 2.8(2.61) | 6.96(6.22) | >=45 |
| skin and subcutaneous tissue disorders | urticaria | 187 | 4.52(3.91, 5.23) | 4.48(3.91, 5.14) | 504.1 | 2.16(1.95) | 4.46(3.95) | >=45 |
| skin and subcutaneous tissue disorders | stevens-johnson syndrome | 63 | 12.69(9.89, 16.28) | 12.64(9.8, 16.31) | 666.63 | 3.64(3.29) | 12.49(10.14) | >=45 |
| skin and subcutaneous tissue disorders | rash erythematous | 96 | 8.66(7.08, 10.6) | 8.61(7.08, 10.47) | 640.4 | 3.09(2.81) | 8.54(7.22) | >=45 |
| general disorders and administration site conditions | infusion site extravasation | 13 | 7.44(4.31, 12.84) | 7.43(4.29, 12.86) | 71.8 | 2.88(2.12) | 7.38(4.67) | >=45 |
| renal and urinary disorders | tubulointerstitial nephritis | 71 | 16.81(13.29, 21.27) | 16.73(13.22, 21.17) | 1032.53 | 4.04(3.7) | 16.46(13.52) | >=45 |
| general disorders and administration site conditions | localised oedema | 7 | 4.78(2.27, 10.05) | 4.78(2.27, 10.07) | 20.82 | 2.25(1.25) | 4.76(2.56) | >=45 |
| renal and urinary disorders | renal tubular necrosis | 148 | 52.96(44.85, 62.54) | 52.41(44.8, 61.31) | 7081.96 | 5.64(5.4) | 49.77(43.31) | >=45 |
| skin and subcutaneous tissue disorders | rash maculo-papular | 90 | 14.31(11.61, 17.63) | 14.22(11.46, 17.64) | 1090.79 | 3.81(3.51) | 14.03(11.78) | >=45 |
| skin and subcutaneous tissue disorders | rash pruritic | 75 | 5.12(4.08, 6.43) | 5.1(4.03, 6.45) | 246.05 | 2.34(2.02) | 5.08(4.2) | >=45 |
| immune system disorders | anaphylactoid reaction | 9 | 7.12(3.7, 13.73) | 7.12(3.73, 13.6) | 46.99 | 2.82(1.92) | 7.07(4.09) | >=45 |
| investigations | antibiotic level above therapeutic | 71 | 1079.53(769.73, 1514.02) | 1074.1(769.73, 1498.83) | 36086.34 | 8.99(8.58) | 509.73(384.08) | >=45 |
| general disorders and administration site conditions | hyperthermia | 13 | 6.52(3.78, 11.25) | 6.51(3.76, 11.27) | 60.29 | 2.7(1.94) | 6.48(4.1) | >=45 |
| immune system disorders | anaphylactic shock | 50 | 6.84(5.18, 9.04) | 6.82(5.18, 8.97) | 246.6 | 2.76(2.36) | 6.78(5.37) | >=45 |
| investigations | antibiotic level below therapeutic | 11 | 968.96(420.02, 2235.3) | 968.2(416.81, 2248.99) | 5314.11 | 8.92(7.91) | 484.6(240.78) | >=45 |
| injury, poisoning and procedural complications | incorrect drug administration rate | 23 | 17.87(11.83, 27) | 17.84(11.82, 26.92) | 359.1 | 4.13(3.55) | 17.54(12.42) | >=45 |
| investigations | eosinophil count increased | 20 | 11.32(7.28, 17.59) | 11.3(7.34, 17.39) | 185.71 | 3.48(2.86) | 11.19(7.73) | >=45 |
| general disorders and administration site conditions | extravasation | 5 | 6.66(2.76, 16.05) | 6.66(2.76, 16.09) | 23.88 | 2.73(1.57) | 6.62(3.17) | >=45 |
| skin and subcutaneous tissue disorders | vancomycin infusion reaction | 35 | 1258.2(761.37, 2079.21) | 1255.07(753.97, 2089.22) | 19099.36 | 9.1(8.5) | 547.13(359.38) | >=45 |
| investigations | eosinophils urine present | 3 | 126.31(37.92, 420.74) | 126.29(38.21, 417.46) | 329.86 | 6.81(5.29) | 111.83(40.86) | >=45 |
| blood and lymphatic system disorders | neutropenia | 112 | 3.58(2.97, 4.32) | 3.56(2.98, 4.25) | 206.18 | 1.83(1.56) | 3.55(3.04) | >=45 |
| investigations | blood urea increased | 46 | 7.21(5.39, 9.64) | 7.19(5.36, 9.65) | 243.46 | 2.84(2.42) | 7.14(5.6) | >=45 |
| injury, poisoning and procedural complications | medication error | 43 | 3.76(2.78, 5.07) | 3.75(2.79, 5.03) | 86.45 | 1.9(1.48) | 3.74(2.91) | >=45 |
| renal and urinary disorders | nephritis | 20 | 24.8(15.9, 38.66) | 24.76(15.78, 38.86) | 444.7 | 4.6(3.97) | 24.17(16.67) | >=45 |
| cardiac disorders | cardio-respiratory arrest | 41 | 4.57(3.36, 6.22) | 4.56(3.33, 6.24) | 113.6 | 2.18(1.75) | 4.55(3.52) | >=45 |
| respiratory, thoracic and mediastinal disorders | respiratory arrest | 26 | 5.85(3.98, 8.61) | 5.84(3.95, 8.64) | 103.75 | 2.54(1.99) | 5.81(4.21) | >=45 |
| respiratory, thoracic and mediastinal disorders | respiratory distress | 25 | 3.43(2.31, 5.08) | 3.42(2.31, 5.06) | 42.75 | 1.77(1.22) | 3.41(2.46) | >=45 |
| skin and subcutaneous tissue disorders | dermatitis exfoliative | 10 | 7.85(4.21, 14.63) | 7.85(4.19, 14.7) | 59.26 | 2.96(2.11) | 7.79(4.63) | >=45 |
| infections and infestations | staphylococcal infection | 42 | 4.73(3.49, 6.41) | 4.72(3.52, 6.33) | 122.71 | 2.23(1.8) | 4.7(3.65) | >=45 |
| general disorders and administration site conditions | drug resistance | 13 | 6.95(4.03, 11.99) | 6.94(4.01, 12.01) | 65.66 | 2.79(2.03) | 6.9(4.37) | >=45 |
| blood and lymphatic system disorders | leukocytosis | 19 | 3.9(2.49, 6.13) | 3.9(2.48, 6.12) | 40.82 | 1.96(1.33) | 3.89(2.67) | >=45 |
| investigations | urine output decreased | 14 | 4.84(2.86, 8.19) | 4.84(2.85, 8.22) | 42.46 | 2.27(1.54) | 4.82(3.11) | >=45 |
| investigations | blood pressure decreased | 61 | 3.04(2.36, 3.91) | 3.03(2.35, 3.91) | 82.82 | 1.6(1.24) | 3.02(2.45) | >=45 |
| respiratory, thoracic and mediastinal disorders | acute respiratory distress syndrome | 16 | 3.61(2.21, 5.91) | 3.61(2.21, 5.89) | 30.1 | 1.85(1.16) | 3.6(2.39) | >=45 |
| investigations | drug level below therapeutic | 13 | 8.37(4.85, 14.45) | 8.36(4.83, 14.47) | 83.56 | 3.05(2.29) | 8.3(5.26) | >=45 |
| hepatobiliary disorders | cholestasis | 46 | 8.14(6.09, 10.89) | 8.12(6.05, 10.9) | 284.97 | 3.01(2.6) | 8.06(6.32) | >=45 |
| vascular disorders | shock | 28 | 5.68(3.91, 8.23) | 5.67(3.91, 8.23) | 107.02 | 2.5(1.97) | 5.64(4.13) | >=45 |
| respiratory, thoracic and mediastinal disorders | tachypnoea | 18 | 4.4(2.77, 6.99) | 4.39(2.74, 7.03) | 46.99 | 2.13(1.48) | 4.38(2.97) | >=45 |
| general disorders and administration site conditions | face oedema | 27 | 6.06(4.15, 8.85) | 6.05(4.17, 8.78) | 113.17 | 2.59(2.05) | 6.02(4.38) | >=45 |
| ear and labyrinth disorders | deafness unilateral | 8 | 4.99(2.49, 10) | 4.99(2.51, 9.91) | 25.38 | 2.31(1.37) | 4.97(2.78) | >=45 |
| infections and infestations | osteomyelitis | 17 | 3.65(2.27, 5.89) | 3.65(2.28, 5.84) | 32.61 | 1.86(1.2) | 3.64(2.44) | >=45 |
| skin and subcutaneous tissue disorders | drug eruption | 40 | 8.69(6.36, 11.87) | 8.67(6.34, 11.86) | 269.01 | 3.1(2.66) | 8.6(6.63) | >=45 |
| ear and labyrinth disorders | deafness | 30 | 5.29(3.7, 7.58) | 5.28(3.71, 7.51) | 103.67 | 2.4(1.89) | 5.26(3.9) | >=45 |
| cardiac disorders | cardiac arrest | 62 | 3.61(2.81, 4.63) | 3.6(2.79, 4.64) | 116.02 | 1.84(1.49) | 3.59(2.91) | >=45 |
| investigations | lymphocyte stimulation test positive | 6 | 23.15(10.3, 52.04) | 23.14(10.36, 51.68) | 124.16 | 4.5(3.42) | 22.63(11.49) | >=45 |
| skin and subcutaneous tissue disorders | toxic skin eruption | 45 | 18.08(13.46, 24.29) | 18.03(13.44, 24.19) | 710.57 | 4.15(3.73) | 17.71(13.84) | >=45 |
| infections and infestations | clostridial infection | 9 | 7.86(4.08, 15.15) | 7.86(4.12, 15.01) | 53.43 | 2.96(2.07) | 7.8(4.51) | >=45 |
| blood and lymphatic system disorders | pancytopenia | 63 | 4.22(3.29, 5.4) | 4.2(3.26, 5.42) | 153.2 | 2.07(1.71) | 4.19(3.4) | >=45 |
| skin and subcutaneous tissue disorders | petechiae | 17 | 5.46(3.39, 8.8) | 5.45(3.4, 8.72) | 61.5 | 2.44(1.77) | 5.43(3.64) | >=45 |
| injury, poisoning and procedural complications | infusion related reaction | 157 | 6.26(5.35, 7.33) | 6.2(5.3, 7.25) | 682.26 | 2.63(2.4) | 6.17(5.41) | >=45 |
| skin and subcutaneous tissue disorders | linear iga disease | 22 | 263.38(164.36, 422.04) | 262.97(164.29, 420.92) | 4515.09 | 7.69(7.04) | 207.01(139.53) | >=45 |
| ear and labyrinth disorders | ear disorder | 6 | 4.56(2.05, 10.18) | 4.56(2.04, 10.19) | 16.62 | 2.18(1.11) | 4.55(2.32) | >=45 |
| respiratory, thoracic and mediastinal disorders | laryngeal oedema | 7 | 3.88(1.85, 8.15) | 3.88(1.84, 8.17) | 14.9 | 1.95(0.95) | 3.87(2.08) | >=45 |
| blood and lymphatic system disorders | thrombocytopenia | 129 | 4.44(3.73, 5.28) | 4.41(3.7, 5.26) | 339.11 | 2.14(1.89) | 4.39(3.8) | >=45 |
| skin and subcutaneous tissue disorders | rash morbilliform | 23 | 32.14(21.21, 48.7) | 32.09(21.26, 48.43) | 670.54 | 4.96(4.37) | 31.09(21.96) | >=45 |
| skin and subcutaneous tissue disorders | skin exfoliation | 60 | 4.23(3.28, 5.45) | 4.21(3.26, 5.43) | 146.44 | 2.07(1.71) | 4.2(3.39) | >=45 |
| investigations | sputum culture positive | 3 | 7.92(2.54, 24.66) | 7.91(2.54, 24.65) | 17.98 | 2.97(1.55) | 7.86(3.04) | >=45 |
| ear and labyrinth disorders | deafness neurosensory | 12 | 16.52(9.33, 29.23) | 16.5(9.35, 29.13) | 171.85 | 4.02(3.23) | 16.24(10.07) | >=45 |
| nervous system disorders | encephalopathy | 36 | 6.17(4.45, 8.57) | 6.16(4.41, 8.6) | 154.7 | 2.62(2.15) | 6.13(4.66) | >=45 |
| nervous system disorders | metabolic encephalopathy | 8 | 8.24(4.11, 16.54) | 8.24(4.15, 16.36) | 50.46 | 3.03(2.08) | 8.18(4.57) | >=45 |
| skin and subcutaneous tissue disorders | purpura | 14 | 5.82(3.44, 9.85) | 5.82(3.43, 9.88) | 55.53 | 2.53(1.8) | 5.79(3.73) | >=45 |
| vascular disorders | circulatory collapse | 34 | 7.29(5.2, 10.22) | 7.27(5.21, 10.14) | 182.65 | 2.85(2.37) | 7.23(5.45) | >=45 |
| respiratory, thoracic and mediastinal disorders | stridor | 4 | 4.85(1.82, 12.97) | 4.85(1.82, 12.92) | 12.18 | 2.27(1) | 4.83(2.12) | >=45 |
| skin and subcutaneous tissue disorders | erythema multiforme | 45 | 17.45(12.99, 23.44) | 17.39(12.96, 23.33) | 683.12 | 4.1(3.67) | 17.1(13.36) | >=45 |
| renal and urinary disorders | renal injury | 20 | 3.09(1.99, 4.8) | 3.09(2.01, 4.76) | 28.23 | 1.63(1.01) | 3.09(2.14) | >=45 |
| skin and subcutaneous tissue disorders | dermatitis bullous | 20 | 11.98(7.7, 18.62) | 11.96(7.77, 18.41) | 198.45 | 3.56(2.94) | 11.83(8.18) | >=45 |
| infections and infestations | clostridium difficile colitis | 22 | 7.01(4.61, 10.67) | 7(4.64, 10.56) | 112.45 | 2.8(2.21) | 6.96(4.9) | >=45 |
| renal and urinary disorders | azotaemia | 5 | 4.62(1.92, 11.12) | 4.61(1.91, 11.14) | 14.09 | 2.2(1.04) | 4.6(2.2) | >=45 |
| gastrointestinal disorders | lip pain | 5 | 6.03(2.5, 14.53) | 6.03(2.5, 14.57) | 20.84 | 2.58(1.43) | 6(2.87) | >=45 |
| vascular disorders | cyanosis | 17 | 3.99(2.48, 6.43) | 3.99(2.49, 6.39) | 37.9 | 1.99(1.32) | 3.98(2.67) | >=45 |
| nervous system disorders | unresponsive to stimuli | 61 | 7.97(6.19, 10.26) | 7.94(6.15, 10.24) | 367.36 | 2.98(2.62) | 7.89(6.38) | >=45 |
| general disorders and administration site conditions | infusion site rash | 4 | 7.32(2.74, 19.59) | 7.32(2.75, 19.5) | 21.67 | 2.86(1.59) | 7.27(3.19) | >=45 |
| investigations | laboratory test interference | 4 | 15.81(5.89, 42.47) | 15.81(5.93, 42.13) | 54.59 | 3.96(2.68) | 15.57(6.81) | >=45 |
| skin and subcutaneous tissue disorders | rash macular | 32 | 4.06(2.87, 5.74) | 4.05(2.85, 5.76) | 73.22 | 2.01(1.52) | 4.04(3.02) | >=45 |
| investigations | transaminases increased | 27 | 4.41(3.02, 6.44) | 4.41(3.04, 6.4) | 70.82 | 2.13(1.6) | 4.39(3.2) | >=45 |
| respiratory, thoracic and mediastinal disorders | respiratory depression | 14 | 6.57(3.88, 11.12) | 6.57(3.87, 11.15) | 65.64 | 2.71(1.97) | 6.53(4.21) | >=45 |
| investigations | pulse absent | 22 | 11.75(7.72, 17.9) | 11.74(7.78, 17.72) | 213.5 | 3.54(2.94) | 11.61(8.16) | >=45 |
| skin and subcutaneous tissue disorders | rash vesicular | 6 | 4.66(2.09, 10.39) | 4.66(2.09, 10.41) | 17.16 | 2.21(1.14) | 4.64(2.37) | >=45 |
| ear and labyrinth disorders | ototoxicity | 12 | 39.96(22.43, 71.2) | 39.93(22.62, 70.49) | 437.38 | 5.26(4.46) | 38.38(23.67) | >=45 |
| infections and infestations | enterococcal infection | 13 | 12.14(7.02, 20.98) | 12.13(7.01, 21) | 131.08 | 3.58(2.82) | 11.99(7.58) | >=45 |
| infections and infestations | staphylococcal bacteraemia | 8 | 6.31(3.15, 12.64) | 6.3(3.17, 12.51) | 35.46 | 2.65(1.7) | 6.27(3.5) | >=45 |
| investigations | anti-platelet antibody positive | 4 | 62.48(22.73, 171.77) | 62.46(22.54, 173.08) | 227.26 | 5.88(4.57) | 58.74(25.2) | >=45 |
| renal and urinary disorders | nephropathy | 16 | 7.34(4.49, 12) | 7.33(4.49, 11.96) | 86.84 | 2.86(2.18) | 7.28(4.83) | >=45 |
| ear and labyrinth disorders | vestibular disorder | 6 | 14.07(6.28, 31.51) | 14.07(6.3, 31.43) | 71.78 | 3.79(2.72) | 13.88(7.07) | >=45 |
| renal and urinary disorders | anuria | 19 | 9.23(5.87, 14.51) | 9.22(5.87, 14.47) | 137.97 | 3.19(2.56) | 9.14(6.26) | >=45 |
| infections and infestations | enterobacter infection | 3 | 7.83(2.51, 24.39) | 7.83(2.51, 24.4) | 17.73 | 2.96(1.54) | 7.77(3) | >=45 |
| respiratory, thoracic and mediastinal disorders | oropharyngeal blistering | 3 | 5.69(1.83, 17.69) | 5.68(1.82, 17.7) | 11.51 | 2.5(1.08) | 5.66(2.19) | >=45 |
| investigations | blood creatine increased | 7 | 5.8(2.76, 12.19) | 5.79(2.75, 12.19) | 27.59 | 2.53(1.52) | 5.76(3.1) | >=45 |
| gastrointestinal disorders | cheilitis | 7 | 4.37(2.08, 9.19) | 4.37(2.08, 9.2) | 18.13 | 2.12(1.12) | 4.36(2.34) | >=45 |
| infections and infestations | liver abscess | 5 | 6.68(2.77, 16.1) | 6.68(2.77, 16.14) | 23.97 | 2.73(1.57) | 6.64(3.18) | >=45 |
| investigations | blood pressure systolic decreased | 6 | 4.13(1.85, 9.21) | 4.13(1.85, 9.22) | 14.17 | 2.04(0.97) | 4.12(2.1) | >=45 |
| general disorders and administration site conditions | administration site pain | 4 | 14.4(5.36, 38.66) | 14.4(5.4, 38.37) | 49.14 | 3.83(2.55) | 14.2(6.22) | >=45 |
| cardiac disorders | pulseless electrical activity | 12 | 11.06(6.26, 19.55) | 11.05(6.26, 19.51) | 108.51 | 3.45(2.66) | 10.94(6.8) | >=45 |
| nervous system disorders | hypoxic-ischaemic encephalopathy | 4 | 6.57(2.46, 17.55) | 6.56(2.46, 17.48) | 18.74 | 2.71(1.44) | 6.53(2.87) | >=45 |
| general disorders and administration site conditions | no reaction on previous exposure to drug | 3 | 10.23(3.28, 31.91) | 10.23(3.28, 31.88) | 24.72 | 3.34(1.92) | 10.13(3.91) | >=45 |
| investigations | red blood cell count abnormal | 5 | 15.83(6.54, 38.3) | 15.82(6.55, 38.22) | 68.3 | 3.96(2.8) | 15.58(7.44) | >=45 |
| investigations | white blood cell count abnormal | 5 | 4.25(1.77, 10.24) | 4.25(1.76, 10.27) | 12.37 | 2.08(0.93) | 4.24(2.03) | >=45 |
| investigations | enterococcus test positive | 3 | 11.96(3.83, 37.33) | 11.95(3.83, 37.25) | 29.74 | 3.56(2.14) | 11.82(4.56) | >=45 |
| injury, poisoning and procedural complications | thermal burn | 7 | 3.85(1.83, 8.1) | 3.85(1.83, 8.11) | 14.73 | 1.94(0.94) | 3.84(2.06) | >=45 |
| hepatobiliary disorders | hepatitis | 23 | 3.51(2.33, 5.29) | 3.51(2.33, 5.3) | 41.16 | 1.81(1.23) | 3.5(2.48) | >=45 |
| blood and lymphatic system disorders | neutrophilia | 5 | 9.14(3.79, 22.05) | 9.13(3.78, 22.06) | 35.88 | 3.18(2.02) | 9.06(4.33) | >=45 |
| skin and subcutaneous tissue disorders | vascular purpura | 8 | 17.34(8.62, 34.89) | 17.33(8.56, 35.09) | 120.93 | 4.09(3.14) | 17.04(9.49) | >=45 |
| respiratory, thoracic and mediastinal disorders | hypercapnia | 5 | 6.58(2.73, 15.86) | 6.58(2.72, 15.9) | 23.49 | 2.71(1.55) | 6.54(3.13) | >=45 |
| respiratory, thoracic and mediastinal disorders | eosinophilic pneumonia | 6 | 8.69(3.89, 19.41) | 8.68(3.89, 19.39) | 40.43 | 3.11(2.03) | 8.62(4.4) | >=45 |
| infections and infestations | clostridium difficile infection | 34 | 6.81(4.86, 9.54) | 6.79(4.87, 9.47) | 166.86 | 2.76(2.28) | 6.75(5.09) | >=45 |
| infections and infestations | pseudomembranous colitis | 3 | 5.41(1.74, 16.83) | 5.41(1.74, 16.86) | 10.72 | 2.43(1.01) | 5.38(2.08) | >=45 |
| skin and subcutaneous tissue disorders | hypersensitivity vasculitis | 17 | 29.58(18.25, 47.95) | 29.55(18.1, 48.23) | 455.06 | 4.84(4.17) | 28.7(19.16) | >=45 |
| blood and lymphatic system disorders | allergic eosinophilia | 3 | 726.3(162.54, 3245.53) | 726.15(163.72, 3220.71) | 1241.4 | 8.7(6.97) | 415.37(118.69) | >=45 |
| general disorders and administration site conditions | catheter site pruritus | 3 | 6.04(1.94, 18.8) | 6.04(1.94, 18.83) | 12.54 | 2.59(1.17) | 6.01(2.32) | >=45 |
| general disorders and administration site conditions | foaming at mouth | 9 | 14.88(7.7, 28.75) | 14.87(7.64, 28.96) | 114.68 | 3.87(2.97) | 14.66(8.45) | >=45 |
| nervous system disorders | tongue biting | 4 | 6.02(2.25, 16.1) | 6.02(2.26, 16.04) | 16.65 | 2.58(1.31) | 5.99(2.63) | >=45 |
| general disorders and administration site conditions | mucosal erosion | 4 | 15.88(5.91, 42.65) | 15.87(5.96, 42.28) | 54.84 | 3.97(2.69) | 15.63(6.84) | >=45 |
| investigations | venous pressure jugular increased | 3 | 24.62(7.83, 77.45) | 24.62(7.9, 76.74) | 66.28 | 4.59(3.15) | 24.03(9.21) | >=45 |
| gastrointestinal disorders | lip oedema | 19 | 21.77(13.81, 34.32) | 21.74(13.85, 34.12) | 367.78 | 4.41(3.77) | 21.29(14.55) | >=45 |
| hepatobiliary disorders | hepatocellular injury | 38 | 7.85(5.7, 10.8) | 7.83(5.72, 10.71) | 224.64 | 2.96(2.5) | 7.77(5.95) | >=45 |
| skin and subcutaneous tissue disorders | acute generalised exanthematous pustulosis | 29 | 24.99(17.28, 36.14) | 24.94(17.19, 36.19) | 649.63 | 4.6(4.08) | 24.33(17.87) | >=45 |
| injury, poisoning and procedural complications | documented hypersensitivity to administered product | 6 | 19.63(8.75, 44.06) | 19.63(8.79, 43.84) | 103.95 | 4.27(3.19) | 19.26(9.79) | >=45 |
| investigations | glomerular filtration rate decreased | 12 | 4.1(2.32, 7.22) | 4.09(2.32, 7.22) | 27.93 | 2.03(1.24) | 4.08(2.54) | >=45 |
| investigations | mast cell degranulation present | 3 | 581.04(138.84, 2431.59) | 580.92(138.91, 2429.43) | 1085.48 | 8.51(6.81) | 363.45(109.71) | >=45 |
| hepatobiliary disorders | hepatitis fulminant | 8 | 10.09(5.03, 20.25) | 10.09(4.98, 20.43) | 64.81 | 3.32(2.37) | 9.99(5.58) | >=45 |
| neoplasms benign, malignant and unspecified (incl cysts and polyps) | neuroendocrine tumour of the lung metastatic | 4 | 184.47(63.31, 537.48) | 184.42(62.75, 541.97) | 612.95 | 7.28(5.9) | 155.07(63.38) | >=45 |
| general disorders and administration site conditions | administration site rash | 3 | 96.84(29.55, 317.35) | 96.82(29.29, 320.04) | 258.63 | 6.46(4.97) | 88.11(32.64) | >=45 |
| vascular disorders | hyperaemia | 12 | 22.23(12.54, 39.41) | 22.21(12.58, 39.21) | 237.68 | 4.44(3.65) | 21.74(13.46) | >=45 |
| injury, poisoning and procedural complications | dose calculation error | 3 | 30.26(9.59, 95.51) | 30.26(9.52, 96.18) | 82.3 | 4.88(3.43) | 29.37(11.23) | >=45 |
| nervous system disorders | tonic clonic movements | 6 | 14.86(6.64, 33.29) | 14.86(6.65, 33.19) | 76.38 | 3.87(2.79) | 14.65(7.46) | >=45 |
| investigations | creatinine renal clearance decreased | 11 | 10.99(6.06, 19.91) | 10.98(6.1, 19.77) | 98.67 | 3.44(2.62) | 10.87(6.61) | >=45 |
| skin and subcutaneous tissue disorders | skin toxicity | 6 | 7.74(3.46, 17.28) | 7.74(3.47, 17.29) | 34.91 | 2.94(1.87) | 7.68(3.92) | >=45 |
| nervous system disorders | myoclonus | 16 | 6.99(4.27, 11.44) | 6.98(4.28, 11.39) | 81.47 | 2.8(2.11) | 6.94(4.6) | >=45 |
| general disorders and administration site conditions | hyperpyrexia | 6 | 4.95(2.22, 11.04) | 4.95(2.22, 11.06) | 18.81 | 2.3(1.23) | 4.93(2.52) | >=45 |
| skin and subcutaneous tissue disorders | symmetrical drug-related intertriginous and flexural exanthema | 9 | 142.94(70.98, 287.86) | 142.85(70.54, 289.28) | 1104.72 | 6.96(6.01) | 124.61(69.37) | >=45 |
| respiratory, thoracic and mediastinal disorders | alveolar lung disease | 3 | 45.39(14.26, 144.51) | 45.38(14.28, 144.24) | 124.39 | 5.44(3.98) | 43.4(16.47) | >=45 |
| respiratory, thoracic and mediastinal disorders | diffuse alveolar damage | 3 | 12.69(4.06, 39.63) | 12.68(4.07, 39.52) | 31.87 | 3.65(2.22) | 12.53(4.83) | >=45 |
| respiratory, thoracic and mediastinal disorders | pulmonary interstitial emphysema syndrome | 3 | 121.05(36.45, 402.05) | 121.02(36.61, 400.04) | 317.42 | 6.75(5.24) | 107.69(39.44) | >=45 |
| investigations | drug clearance decreased | 6 | 22.7(10.1, 51.01) | 22.69(10.16, 50.68) | 121.57 | 4.47(3.39) | 22.2(11.27) | >=45 |
| eye disorders | eye oedema | 5 | 7.9(3.28, 19.05) | 7.9(3.27, 19.08) | 29.88 | 2.97(1.81) | 7.84(3.75) | >=45 |
| general disorders and administration site conditions | administration site erythema | 3 | 35.43(11.19, 112.14) | 35.42(11.14, 112.58) | 96.81 | 5.1(3.65) | 34.21(13.04) | >=45 |
| blood and lymphatic system disorders | haemolytic anaemia | 14 | 6.47(3.82, 10.94) | 6.46(3.81, 10.97) | 64.2 | 2.68(1.95) | 6.42(4.14) | >=45 |
| nervous system disorders | partial seizures | 5 | 4.49(1.87, 10.82) | 4.49(1.86, 10.85) | 13.51 | 2.16(1) | 4.47(2.15) | >=45 |
| metabolism and nutrition disorders | cell death | 6 | 9.12(4.08, 20.39) | 9.12(4.08, 20.37) | 42.97 | 3.18(2.1) | 9.04(4.61) | >=45 |
| hepatobiliary disorders | hypertransaminasaemia | 7 | 5.23(2.49, 10.99) | 5.23(2.48, 11.01) | 23.79 | 2.38(1.38) | 5.2(2.79) | >=45 |
| renal and urinary disorders | crystalluria | 10 | 72.85(38.3, 138.56) | 72.8(38.13, 139.01) | 658.6 | 6.08(5.2) | 67.78(39.58) | >=45 |
| investigations | drug trough level | 5 | 2421.36(469.72, 12481.72) | 2420.5(466.53, 12558.2) | 3455 | 9.44(7.91) | 692.29(175.53) | >=45 |
| blood and lymphatic system disorders | bicytopenia | 8 | 14.38(7.15, 28.9) | 14.37(7.1, 29.1) | 98.07 | 3.83(2.87) | 14.17(7.9) | >=45 |
| renal and urinary disorders | renal tubular injury | 5 | 49.92(20.32, 122.67) | 49.91(20.26, 122.96) | 227.89 | 5.57(4.38) | 47.51(22.39) | >=45 |
| investigations | haematocrit abnormal | 7 | 25.68(12.12, 54.41) | 25.67(12.19, 54.06) | 161.69 | 4.65(3.63) | 25.03(13.36) | >=45 |
| investigations | haemoglobin abnormal | 7 | 5.8(2.76, 12.19) | 5.79(2.75, 12.19) | 27.59 | 2.53(1.52) | 5.76(3.1) | >=45 |
| investigations | prothrombin time abnormal | 4 | 15.43(5.75, 41.45) | 15.43(5.79, 41.11) | 53.13 | 3.93(2.65) | 15.2(6.65) | >=45 |
| investigations | red cell distribution width abnormal | 7 | 188.35(83.81, 423.32) | 188.26(84.29, 420.49) | 1091.61 | 7.3(6.21) | 157.78(80.13) | >=45 |
| skin and subcutaneous tissue disorders | dermatitis exfoliative generalised | 15 | 16.37(9.82, 27.28) | 16.35(9.82, 27.22) | 212.66 | 4.01(3.3) | 16.1(10.5) | >=45 |
| investigations | lymphocyte morphology abnormal | 4 | 29.57(10.93, 79.98) | 29.56(10.88, 80.32) | 107.12 | 4.84(3.56) | 28.72(12.49) | >=45 |
| blood and lymphatic system disorders | anisocytosis | 7 | 34.95(16.44, 74.31) | 34.94(16.59, 73.58) | 222.71 | 5.08(4.06) | 33.75(17.96) | >=45 |
| injury, poisoning and procedural complications | central nervous system injury | 7 | 616.43(238.93, 1590.4) | 616.13(240.49, 1578.54) | 2627.11 | 8.56(7.37) | 376.91(170.54) | >=45 |
| metabolism and nutrition disorders | hyperchloraemia | 7 | 77.94(36.08, 168.37) | 77.9(36.27, 167.31) | 491.83 | 6.17(5.13) | 72.17(37.89) | >=45 |
| blood and lymphatic system disorders | hypochromasia | 7 | 111.16(50.84, 243.06) | 111.1(50.73, 243.33) | 685.17 | 6.64(5.58) | 99.77(51.85) | >=45 |
| blood and lymphatic system disorders | macrocytosis | 7 | 21.8(10.31, 46.12) | 21.79(10.35, 45.89) | 135.81 | 4.42(3.4) | 21.33(11.4) | >=45 |
| injury, poisoning and procedural complications | splenic injury | 7 | 102.74(47.13, 223.96) | 102.69(46.89, 224.91) | 637.29 | 6.54(5.49) | 92.94(48.42) | >=45 |
| cardiac disorders | tachyarrhythmia | 8 | 9.45(4.71, 18.97) | 9.45(4.67, 19.14) | 59.83 | 3.23(2.28) | 9.36(5.23) | >=45 |
| general disorders and administration site conditions | vessel puncture site inflammation | 4 | 430.43(132.54, 1397.89) | 430.31(132.76, 1394.8) | 1186.1 | 8.22(6.75) | 298.22(111.3) | >=45 |
| immune system disorders | type iv hypersensitivity reaction | 5 | 11.96(4.95, 28.89) | 11.95(4.95, 28.87) | 49.57 | 3.56(2.4) | 11.82(5.65) | >=45 |
| cardiac disorders | kounis syndrome | 7 | 62.78(29.23, 134.87) | 62.75(29.22, 134.77) | 399.5 | 5.88(4.85) | 58.99(31.11) | >=45 |
| investigations | bacterial test | 5 | 47.95(19.53, 117.71) | 47.93(19.46, 118.08) | 218.92 | 5.51(4.33) | 45.72(21.56) | >=45 |
| infections and infestations | systemic infection | 5 | 6.44(2.67, 15.52) | 6.44(2.67, 15.56) | 22.81 | 2.68(1.52) | 6.4(3.07) | >=45 |
| injury, poisoning and procedural complications | therapeutic drug monitoring analysis incorrectly performed | 5 | 127.44(50.15, 323.83) | 127.39(49.72, 326.38) | 554.11 | 6.82(5.59) | 112.7(51.65) | >=45 |
| investigations | peritoneal effluent leukocyte count increased | 4 | 1291.3(288.97, 5770.24) | 1290.93(291.06, 5725.69) | 2209.6 | 9.11(7.5) | 553.83(158.25) | >=45 |
| gastrointestinal disorders | peritoneal cloudy effluent | 4 | 21.52(7.99, 57.98) | 21.52(7.92, 58.47) | 76.55 | 4.4(3.12) | 21.07(9.19) | >=45 |
| infections and infestations | lymphangitis | 7 | 30(14.14, 63.67) | 29.99(14.24, 63.16) | 190.26 | 4.86(3.85) | 29.12(15.51) | >=45 |
| general disorders and administration site conditions | infusion site thrombosis | 3 | 30.58(9.69, 96.53) | 30.57(9.62, 97.17) | 83.2 | 4.89(3.45) | 29.67(11.34) | >=45 |
| nervous system disorders | toxic encephalopathy | 5 | 5.77(2.4, 13.91) | 5.77(2.39, 13.94) | 19.6 | 2.52(1.36) | 5.74(2.75) | >=45 |
| metabolism and nutrition disorders | hypervolaemia | 7 | 5.82(2.77, 12.25) | 5.82(2.76, 12.26) | 27.8 | 2.53(1.53) | 5.79(3.11) | >=45 |
| renal and urinary disorders | kidney congestion | 3 | 76.45(23.6, 247.7) | 76.44(23.58, 247.77) | 207.01 | 6.15(4.67) | 70.92(26.52) | >=45 |
| eye disorders | limbal swelling | 3 | 484.2(121.08, 1936.29) | 484.1(120.38, 1946.7) | 964.2 | 8.34(6.66) | 323.07(101.3) | >=45 |
| investigations | mean cell haemoglobin concentration increased | 3 | 17.19(5.49, 53.84) | 17.19(5.52, 53.58) | 44.94 | 4.08(2.65) | 16.9(6.5) | >=45 |
| vascular disorders | venoocclusive disease | 4 | 12.3(4.59, 32.98) | 12.29(4.61, 32.75) | 40.98 | 3.6(2.33) | 12.15(5.32) | >=45 |
| congenital, familial and genetic disorders | macroglossia | 3 | 27.41(8.7, 86.35) | 27.4(8.62, 87.09) | 74.22 | 4.74(3.3) | 26.68(10.21) | >=45 |
| hepatobiliary disorders | cholestatic liver injury | 6 | 13.42(6, 30.05) | 13.42(6.01, 29.97) | 68 | 3.73(2.65) | 13.25(6.75) | >=45 |
| blood and lymphatic system disorders | immune thrombocytopenia | 5 | 6.65(2.76, 16.03) | 6.65(2.75, 16.06) | 23.84 | 2.72(1.57) | 6.61(3.17) | >=45 |
| skin and subcutaneous tissue disorders | cutaneous vasculitis | 4 | 6.51(2.44, 17.41) | 6.51(2.44, 17.35) | 18.53 | 2.69(1.42) | 6.47(2.84) | >=45 |
| metabolism and nutrition disorders | calciphylaxis | 4 | 25.15(9.32, 67.89) | 25.15(9.26, 68.34) | 90.4 | 4.62(3.33) | 24.54(10.69) | >=45 |
| skin and subcutaneous tissue disorders | cutaneous calcification | 3 | 138.34(41.26, 463.86) | 138.31(41.03, 466.24) | 357.84 | 6.92(5.4) | 121.15(44.02) | >=45 |
| immune system disorders | infusion related hypersensitivity reaction | 3 | 18.86(6.02, 59.15) | 18.86(6.05, 58.78) | 49.77 | 4.21(2.78) | 18.52(7.12) | >=45 |
| skin and subcutaneous tissue disorders | erythrosis | 4 | 80.71(29.1, 223.85) | 80.68(29.12, 223.56) | 290.57 | 6.22(4.9) | 74.55(31.75) | >=45 |
| investigations | allergy test positive | 4 | 51.65(18.89, 141.24) | 51.64(19, 140.32) | 188.57 | 5.62(4.31) | 49.07(21.15) | >=45 |
| injury, poisoning and procedural complications | burns second degree | 3 | 6.51(2.09, 20.28) | 6.51(2.09, 20.29) | 13.91 | 2.7(1.27) | 6.48(2.5) | >=45 |
| gastrointestinal disorders | dyschezia | 5 | 6.13(2.54, 14.77) | 6.13(2.54, 14.81) | 21.32 | 2.61(1.45) | 6.1(2.92) | >=45 |
| investigations | false positive investigation result | 3 | 18.16(5.79, 56.9) | 18.15(5.82, 56.57) | 47.73 | 4.16(2.73) | 17.84(6.86) | >=45 |
| respiratory, thoracic and mediastinal disorders | respiratory acidosis | 5 | 5.78(2.4, 13.92) | 5.78(2.39, 13.96) | 19.63 | 2.52(1.36) | 5.75(2.75) | >=45 |
| injury, poisoning and procedural complications | sternal fracture | 5 | 14.54(6.01, 35.17) | 14.54(6.02, 35.12) | 62.1 | 3.84(2.68) | 14.34(6.85) | >=45 |
| renal and urinary disorders | acute kidney injury | 1676 | 19.76(18.8, 20.76) | 18.91(18.18, 19.67) | 27983.66 | 4.22(4.14) | 18.59(17.83) | unknow |
| skin and subcutaneous tissue disorders | drug reaction with eosinophilia and systemic symptoms | 1076 | 65.83(61.84, 70.07) | 63.96(60.31, 67.83) | 62774.2 | 5.91(5.82) | 60.24(57.17) | unknow |
| investigations | blood creatinine increased | 251 | 6.51(5.75, 7.37) | 6.47(5.75, 7.28) | 1154.62 | 2.69(2.51) | 6.44(5.8) | unknow |
| renal and urinary disorders | nephropathy toxic | 528 | 73.73(67.46, 80.58) | 72.7(67.22, 78.63) | 34857.15 | 6.09(5.96) | 67.92(63.06) | unknow |
| investigations | drug level increased | 288 | 26.43(23.5, 29.72) | 26.23(23.32, 29.5) | 6816.71 | 4.68(4.51) | 25.6(23.2) | unknow |
| immune system disorders | anaphylactic reaction | 238 | 7.21(6.35, 8.2) | 7.17(6.37, 8.06) | 1256.45 | 2.83(2.65) | 7.13(6.41) | unknow |
| renal and urinary disorders | renal impairment | 262 | 5(4.43, 5.65) | 4.97(4.42, 5.59) | 828.66 | 2.31(2.13) | 4.95(4.47) | unknow |
| blood and lymphatic system disorders | leukopenia | 224 | 7.43(6.51, 8.48) | 7.39(6.44, 8.48) | 1230.02 | 2.88(2.69) | 7.35(6.58) | unknow |
| blood and lymphatic system disorders | eosinophilia | 199 | 18.3(15.9, 21.06) | 18.21(15.88, 20.89) | 3180.09 | 4.16(3.96) | 17.9(15.92) | unknow |
| infections and infestations | pathogen resistance | 135 | 18.62(15.7, 22.08) | 18.56(15.56, 22.14) | 2202.6 | 4.19(3.94) | 18.24(15.82) | unknow |
| investigations | drug level above therapeutic | 33 | 12.28(8.71, 17.32) | 12.27(8.62, 17.46) | 337.64 | 3.6(3.11) | 12.14(9.11) | unknow |
| renal and urinary disorders | renal tubular disorder | 25 | 14.63(9.86, 21.71) | 14.62(9.88, 21.64) | 312.71 | 3.85(3.29) | 14.43(10.37) | unknow |
| renal and urinary disorders | renal failure | 281 | 3.19(2.84, 3.59) | 3.18(2.83, 3.58) | 418.4 | 1.66(1.49) | 3.17(2.87) | unknow |
| skin and subcutaneous tissue disorders | toxic epidermal necrolysis | 175 | 17.38(14.97, 20.19) | 17.31(14.8, 20.25) | 2644.62 | 4.09(3.87) | 17.03(15.03) | unknow |
| skin and subcutaneous tissue disorders | skin necrosis | 17 | 4.8(2.98, 7.74) | 4.8(3, 7.68) | 50.94 | 2.26(1.59) | 4.78(3.21) | unknow |
| infections and infestations | septic shock | 219 | 8.67(7.59, 9.91) | 8.62(7.51, 9.89) | 1464.69 | 3.1(2.91) | 8.56(7.66) | unknow |
| infections and infestations | systemic candida | 41 | 26.13(19.16, 35.63) | 26.1(19.07, 35.71) | 965.08 | 4.67(4.23) | 25.48(19.65) | unknow |
| respiratory, thoracic and mediastinal disorders | hypoxia | 73 | 4.08(3.24, 5.13) | 4.07(3.22, 5.15) | 168.54 | 2.02(1.69) | 4.06(3.35) | unknow |
| general disorders and administration site conditions | generalised oedema | 22 | 3.15(2.07, 4.79) | 3.15(2.09, 4.75) | 32.24 | 1.65(1.06) | 3.15(2.22) | unknow |
| renal and urinary disorders | oliguria | 51 | 13.09(9.93, 17.25) | 13.07(9.93, 17.2) | 561.29 | 3.69(3.3) | 12.92(10.25) | unknow |
| general disorders and administration site conditions | treatment failure | 449 | 7.2(6.56, 7.91) | 7.13(6.46, 7.86) | 2353.84 | 2.83(2.69) | 7.09(6.55) | unknow |
| vascular disorders | haemodynamic instability | 74 | 15.17(12.06, 19.09) | 15.14(11.97, 19.15) | 963.24 | 3.9(3.57) | 14.94(12.32) | unknow |
| infections and infestations | candida infection | 89 | 8.57(6.95, 10.56) | 8.55(6.89, 10.61) | 588.47 | 3.09(2.79) | 8.49(7.13) | unknow |
| infections and infestations | endocarditis | 51 | 15.8(11.98, 20.84) | 15.78(11.99, 20.76) | 695.34 | 3.96(3.56) | 15.56(12.34) | unknow |
| infections and infestations | cns ventriculitis | 11 | 48.73(26.61, 89.24) | 48.72(26.54, 89.45) | 490.65 | 5.54(4.7) | 46.54(28.05) | unknow |
| blood and lymphatic system disorders | agranulocytosis | 66 | 6.41(5.03, 8.17) | 6.4(5.06, 8.1) | 299.18 | 2.67(2.32) | 6.37(5.2) | unknow |
| immune system disorders | drug hypersensitivity | 1156 | 8.27(7.79, 8.77) | 8.04(7.58, 8.53) | 7097.85 | 3(2.91) | 7.98(7.6) | unknow |
| skin and subcutaneous tissue disorders | stevens-johnson syndrome | 113 | 7.78(6.46, 9.36) | 7.76(6.51, 9.26) | 660.62 | 2.95(2.68) | 7.71(6.6) | unknow |
| skin and subcutaneous tissue disorders | rash erythematous | 144 | 5.43(4.61, 6.39) | 5.41(4.62, 6.33) | 515.16 | 2.43(2.19) | 5.39(4.69) | unknow |
| general disorders and administration site conditions | infusion site extravasation | 12 | 3.24(1.84, 5.72) | 3.24(1.84, 5.72) | 18.57 | 1.69(0.91) | 3.24(2.01) | unknow |
| metabolism and nutrition disorders | hypernatraemia | 11 | 3.74(2.07, 6.76) | 3.74(2.08, 6.73) | 21.98 | 1.9(1.08) | 3.73(2.27) | unknow |
| renal and urinary disorders | tubulointerstitial nephritis | 242 | 20.36(17.92, 23.13) | 20.23(17.99, 22.75) | 4339.8 | 4.31(4.13) | 19.86(17.85) | unknow |
| renal and urinary disorders | renal tubular necrosis | 360 | 67.67(60.8, 75.32) | 67.03(60.77, 73.93) | 21973.04 | 5.98(5.82) | 62.95(57.55) | unknow |
| skin and subcutaneous tissue disorders | rash maculo-papular | 202 | 16.2(14.09, 18.62) | 16.11(14.04, 18.48) | 2819.75 | 3.99(3.79) | 15.88(14.13) | unknow |
| immune system disorders | anaphylactoid reaction | 25 | 10.82(7.3, 16.05) | 10.81(7.3, 16) | 220.32 | 3.42(2.86) | 10.71(7.7) | unknow |
| investigations | antibiotic level above therapeutic | 67 | 712.2(521.22, 973.15) | 710.92(519.55, 972.78) | 27974.29 | 8.71(8.3) | 419.11(322.77) | unknow |
| blood and lymphatic system disorders | lymphadenopathy | 83 | 3.76(3.03, 4.67) | 3.76(3.03, 4.66) | 167.5 | 1.91(1.6) | 3.75(3.13) | unknow |
| eye disorders | periorbital oedema | 20 | 5.74(3.7, 8.9) | 5.73(3.72, 8.82) | 77.74 | 2.51(1.89) | 5.71(3.95) | unknow |
| skin and subcutaneous tissue disorders | angioedema | 88 | 3.45(2.79, 4.25) | 3.44(2.77, 4.27) | 151.94 | 1.78(1.48) | 3.43(2.88) | unknow |
| immune system disorders | anaphylactic shock | 71 | 4.9(3.88, 6.19) | 4.89(3.87, 6.19) | 219.08 | 2.29(1.95) | 4.88(4.01) | unknow |
| investigations | antibiotic level below therapeutic | 9 | 158.1(78.33, 319.1) | 158.06(78.05, 320.08) | 1215.95 | 7.1(6.14) | 136.97(76.11) | unknow |
| injury, poisoning and procedural complications | incorrect drug administration rate | 47 | 22.08(16.53, 29.48) | 22.05(16.43, 29.59) | 924.63 | 4.43(4.02) | 21.61(16.96) | unknow |
| investigations | eosinophil count increased | 24 | 4.41(2.95, 6.59) | 4.41(2.98, 6.53) | 62.98 | 2.14(1.57) | 4.39(3.14) | unknow |
| investigations | blood electrolytes abnormal | 7 | 9.65(4.58, 20.32) | 9.65(4.58, 20.32) | 53.76 | 3.26(2.25) | 9.57(5.13) | unknow |
| general disorders and administration site conditions | extravasation | 24 | 8.37(5.6, 12.51) | 8.37(5.66, 12.39) | 154.41 | 3.05(2.49) | 8.31(5.93) | unknow |
| skin and subcutaneous tissue disorders | vancomycin infusion reaction | 108 | 1362.12(1020.99, 1817.23) | 1358.18(1012.22, 1822.38) | 62772.01 | 9.19(8.85) | 582.65(457.77) | unknow |
| investigations | blood urea increased | 41 | 4.63(3.41, 6.29) | 4.62(3.38, 6.32) | 115.97 | 2.2(1.77) | 4.61(3.56) | unknow |
| renal and urinary disorders | nephritis | 19 | 9.44(6.01, 14.83) | 9.43(6.01, 14.8) | 141.91 | 3.23(2.59) | 9.35(6.41) | unknow |
| respiratory, thoracic and mediastinal disorders | respiratory distress | 55 | 3.58(2.75, 4.67) | 3.58(2.77, 4.62) | 101.73 | 1.83(1.46) | 3.57(2.86) | unknow |
| skin and subcutaneous tissue disorders | dermatitis exfoliative | 39 | 11.42(8.33, 15.66) | 11.41(8.34, 15.61) | 366.31 | 3.5(3.05) | 11.29(8.67) | unknow |
| infections and infestations | staphylococcal infection | 149 | 7.2(6.12, 8.46) | 7.17(6.13, 8.39) | 786.43 | 2.83(2.6) | 7.13(6.23) | unknow |
| general disorders and administration site conditions | drug resistance | 194 | 9.62(8.35, 11.08) | 9.57(8.34, 10.98) | 1476.45 | 3.25(3.04) | 9.49(8.43) | unknow |
| blood and lymphatic system disorders | leukocytosis | 85 | 7.73(6.25, 9.57) | 7.72(6.22, 9.58) | 493.42 | 2.94(2.63) | 7.67(6.41) | unknow |
| investigations | urine output decreased | 17 | 3.55(2.2, 5.71) | 3.55(2.22, 5.68) | 31 | 1.82(1.16) | 3.54(2.38) | unknow |
| respiratory, thoracic and mediastinal disorders | acute respiratory distress syndrome | 65 | 5.93(4.65, 7.57) | 5.92(4.68, 7.49) | 264.5 | 2.56(2.21) | 5.89(4.81) | unknow |
| investigations | drug level below therapeutic | 29 | 9.19(6.38, 13.25) | 9.19(6.33, 13.34) | 209.66 | 3.19(2.67) | 9.11(6.71) | unknow |
| hepatobiliary disorders | cholestasis | 60 | 5.53(4.29, 7.13) | 5.52(4.28, 7.12) | 221.17 | 2.46(2.1) | 5.5(4.45) | unknow |
| vascular disorders | shock | 79 | 5.47(4.38, 6.82) | 5.46(4.4, 6.77) | 286.34 | 2.44(2.13) | 5.44(4.52) | unknow |
| general disorders and administration site conditions | face oedema | 83 | 7.89(6.36, 9.8) | 7.88(6.35, 9.78) | 494.55 | 2.97(2.66) | 7.82(6.53) | unknow |
| skin and subcutaneous tissue disorders | drug eruption | 163 | 15.95(13.66, 18.63) | 15.89(13.58, 18.59) | 2239.34 | 3.97(3.75) | 15.66(13.75) | unknow |
| skin and subcutaneous tissue disorders | toxic skin eruption | 95 | 15.01(12.25, 18.38) | 14.97(12.31, 18.21) | 1221.04 | 3.88(3.59) | 14.77(12.47) | unknow |
| infections and infestations | clostridial infection | 12 | 5.9(3.35, 10.41) | 5.9(3.34, 10.42) | 48.58 | 2.55(1.77) | 5.87(3.65) | unknow |
| blood and lymphatic system disorders | pancytopenia | 141 | 4.25(3.6, 5.02) | 4.24(3.62, 4.96) | 348.18 | 2.08(1.84) | 4.23(3.68) | unknow |
| skin and subcutaneous tissue disorders | petechiae | 43 | 7.34(5.44, 9.91) | 7.33(5.46, 9.84) | 233.6 | 2.87(2.44) | 7.29(5.67) | unknow |
| skin and subcutaneous tissue disorders | linear iga disease | 553 | 722.85(648.12, 806.19) | 712.14(633.13, 801.01) | 231133.94 | 8.71(8.57) | 419.54(382.93) | unknow |
| blood and lymphatic system disorders | thrombocytopenia | 355 | 5.24(4.72, 5.82) | 5.2(4.71, 5.74) | 1201.15 | 2.37(2.22) | 5.18(4.75) | unknow |
| skin and subcutaneous tissue disorders | rash morbilliform | 75 | 47.55(37.71, 59.95) | 47.45(37.51, 60.03) | 3258.81 | 5.5(5.17) | 45.38(37.38) | unknow |
| ear and labyrinth disorders | deafness neurosensory | 10 | 4.96(2.66, 9.23) | 4.96(2.65, 9.29) | 31.43 | 2.3(1.45) | 4.94(2.94) | unknow |
| nervous system disorders | encephalopathy | 60 | 3.94(3.06, 5.08) | 3.94(3.05, 5.08) | 131.05 | 1.97(1.61) | 3.93(3.18) | unknow |
| nervous system disorders | metabolic encephalopathy | 7 | 4.11(1.96, 8.63) | 4.11(1.95, 8.66) | 16.39 | 2.03(1.03) | 4.09(2.2) | unknow |
| skin and subcutaneous tissue disorders | purpura | 49 | 8.91(6.72, 11.8) | 8.9(6.76, 11.71) | 340.54 | 3.14(2.74) | 8.83(6.98) | unknow |
| skin and subcutaneous tissue disorders | erythema multiforme | 57 | 10.44(8.04, 13.56) | 10.43(8.08, 13.46) | 481.1 | 3.37(3) | 10.33(8.31) | unknow |
| skin and subcutaneous tissue disorders | dermatitis bullous | 43 | 9.4(6.96, 12.69) | 9.39(7, 12.6) | 319.33 | 3.22(2.79) | 9.31(7.24) | unknow |
| infections and infestations | clostridium difficile colitis | 63 | 10.27(8.01, 13.17) | 10.26(7.95, 13.24) | 521.13 | 3.35(2.99) | 10.16(8.26) | unknow |
| renal and urinary disorders | azotaemia | 17 | 7.18(4.45, 11.56) | 7.17(4.48, 11.48) | 89.69 | 2.83(2.16) | 7.13(4.78) | unknow |
| investigations | laboratory test interference | 7 | 14.7(6.97, 31.01) | 14.7(6.98, 30.96) | 88.12 | 3.86(2.85) | 14.51(7.77) | unknow |
| investigations | transaminases increased | 72 | 5.41(4.29, 6.82) | 5.4(4.27, 6.83) | 256.64 | 2.43(2.09) | 5.37(4.43) | unknow |
| skin and subcutaneous tissue disorders | rash vesicular | 10 | 3.58(1.93, 6.67) | 3.58(1.91, 6.7) | 18.57 | 1.84(0.98) | 3.58(2.13) | unknow |
| ear and labyrinth disorders | ototoxicity | 40 | 32.12(23.44, 44) | 32.08(23.44, 43.9) | 1167.82 | 4.96(4.51) | 31.13(23.92) | unknow |
| infections and infestations | enterococcal infection | 113 | 37.89(31.39, 45.73) | 37.78(31.06, 45.96) | 3901.41 | 5.19(4.92) | 36.46(31.15) | unknow |
| infections and infestations | staphylococcal bacteraemia | 55 | 23.55(18.02, 30.78) | 23.52(17.88, 30.95) | 1159.25 | 4.52(4.14) | 23.01(18.4) | unknow |
| renal and urinary disorders | nephropathy | 85 | 13.27(10.71, 16.44) | 13.24(10.67, 16.43) | 949.91 | 3.71(3.4) | 13.09(10.94) | unknow |
| ear and labyrinth disorders | vestibular disorder | 15 | 22.35(13.4, 37.28) | 22.34(13.42, 37.19) | 299.19 | 4.45(3.74) | 21.88(14.26) | unknow |
| renal and urinary disorders | anuria | 46 | 8.27(6.19, 11.06) | 8.26(6.16, 11.08) | 291.26 | 3.04(2.62) | 8.2(6.43) | unknow |
| infections and infestations | enterobacter infection | 16 | 15.32(9.35, 25.11) | 15.32(9.39, 25.01) | 210.96 | 3.92(3.23) | 15.11(9.99) | unknow |
| investigations | blood creatine increased | 15 | 5.47(3.29, 9.09) | 5.47(3.29, 9.11) | 54.51 | 2.45(1.74) | 5.45(3.56) | unknow |
| infections and infestations | liver abscess | 11 | 5.51(3.05, 9.97) | 5.51(3.06, 9.92) | 40.38 | 2.46(1.64) | 5.48(3.34) | unknow |
| cardiac disorders | pulseless electrical activity | 20 | 8.21(5.28, 12.74) | 8.2(5.33, 12.62) | 125.47 | 3.03(2.41) | 8.14(5.64) | unknow |
| blood and lymphatic system disorders | neutrophilia | 14 | 3.25(1.92, 5.5) | 3.25(1.91, 5.52) | 21.77 | 1.7(0.97) | 3.25(2.09) | unknow |
| respiratory, thoracic and mediastinal disorders | eosinophilic pneumonia | 21 | 10.86(7.06, 16.69) | 10.85(7.05, 16.7) | 185.88 | 3.43(2.82) | 10.75(7.5) | unknow |
| infections and infestations | clostridium difficile infection | 249 | 18.92(16.68, 21.45) | 18.8(16.71, 21.15) | 4121.04 | 4.21(4.03) | 18.47(16.63) | unknow |
| infections and infestations | pseudomembranous colitis | 18 | 12.54(7.88, 19.96) | 12.53(7.83, 20.06) | 188.71 | 3.63(2.98) | 12.39(8.4) | unknow |
| skin and subcutaneous tissue disorders | hypersensitivity vasculitis | 27 | 14.11(9.65, 20.64) | 14.1(9.72, 20.46) | 324.25 | 3.8(3.26) | 13.93(10.13) | unknow |
| hepatobiliary disorders | hepatocellular injury | 41 | 5.12(3.77, 6.96) | 5.12(3.74, 7.01) | 135.2 | 2.35(1.91) | 5.1(3.94) | unknow |
| skin and subcutaneous tissue disorders | acute generalised exanthematous pustulosis | 192 | 35.48(30.71, 40.99) | 35.3(30.77, 40.49) | 6186.15 | 5.09(4.89) | 34.15(30.27) | unknow |
| skin and subcutaneous tissue disorders | symmetrical drug-related intertriginous and flexural exanthema | 18 | 32.24(20.16, 51.55) | 32.22(20.13, 51.57) | 527.9 | 4.97(4.31) | 31.27(21.11) | unknow |
| blood and lymphatic system disorders | haemolytic anaemia | 38 | 6.7(4.87, 9.22) | 6.7(4.9, 9.17) | 182.98 | 2.74(2.28) | 6.66(5.1) | unknow |
| hepatobiliary disorders | hypertransaminasaemia | 17 | 4.55(2.82, 7.33) | 4.55(2.84, 7.28) | 46.84 | 2.18(1.51) | 4.53(3.04) | unknow |
| renal and urinary disorders | renal tubular injury | 23 | 30.93(20.42, 46.83) | 30.91(20.48, 46.65) | 646.05 | 4.91(4.32) | 30.03(21.22) | unknow |
| skin and subcutaneous tissue disorders | dermatitis exfoliative generalised | 21 | 11.74(7.63, 18.05) | 11.73(7.62, 18.05) | 203.87 | 3.54(2.93) | 11.61(8.1) | unknow |
| immune system disorders | type iv hypersensitivity reaction | 55 | 29.21(22.33, 38.19) | 29.16(22.16, 38.37) | 1454.37 | 4.83(4.44) | 28.38(22.67) | unknow |
| cardiac disorders | kounis syndrome | 82 | 48.81(39.1, 60.93) | 48.7(39.26, 60.42) | 3656.78 | 5.54(5.22) | 46.53(38.65) | unknow |
| blood and lymphatic system disorders | immune thrombocytopenia | 78 | 31.42(25.07, 39.37) | 31.35(25.27, 38.89) | 2223.88 | 4.93(4.61) | 30.45(25.21) | unknow |
| skin and subcutaneous tissue disorders | cutaneous vasculitis | 39 | 19.25(14.02, 26.43) | 19.23(14.05, 26.31) | 661.5 | 4.24(3.79) | 18.89(14.49) | unknow |
| general disorders and administration site conditions | pyrexia | 620 | 3.08(2.84, 3.33) | 3.04(2.81, 3.29) | 852.16 | 1.6(1.49) | 3.04(2.84) | unknow |
| blood and lymphatic system disorders | disseminated intravascular coagulation | 65 | 7.99(6.26, 10.2) | 7.98(6.31, 10.1) | 393.92 | 2.99(2.64) | 7.93(6.46) | unknow |
| hepatobiliary disorders | hepatorenal syndrome | 9 | 9.1(4.72, 17.54) | 9.09(4.76, 17.36) | 64.27 | 3.17(2.27) | 9.02(5.21) | unknow |
| infections and infestations | cardiac valve vegetation | 18 | 57.15(35.54, 91.88) | 57.12(35.69, 91.43) | 939.78 | 5.76(5.09) | 54.14(36.39) | unknow |
| infections and infestations | klebsiella infection | 45 | 14.16(10.55, 19.01) | 14.14(10.54, 18.97) | 542.15 | 3.8(3.38) | 13.96(10.91) | unknow |
| infections and infestations | pseudomonas infection | 27 | 5.1(3.49, 7.44) | 5.09(3.51, 7.39) | 88.36 | 2.34(1.81) | 5.07(3.7) | unknow |
| investigations | blood culture positive | 7 | 6.23(2.96, 13.1) | 6.23(2.96, 13.12) | 30.53 | 2.63(1.63) | 6.2(3.33) | unknow |
| psychiatric disorders | mental status changes | 58 | 3.68(2.85, 4.77) | 3.68(2.85, 4.75) | 112.82 | 1.88(1.51) | 3.67(2.96) | unknow |
| infections and infestations | acinetobacter infection | 28 | 38.62(26.48, 56.34) | 38.6(26.6, 56.02) | 987.97 | 5.22(4.68) | 37.22(27.14) | unknow |
| infections and infestations | abscess limb | 9 | 3.58(1.86, 6.88) | 3.58(1.87, 6.84) | 16.64 | 1.83(0.94) | 3.57(2.06) | unknow |
| investigations | drug level decreased | 24 | 3.75(2.51, 5.6) | 3.75(2.53, 5.55) | 48.14 | 1.9(1.33) | 3.74(2.67) | unknow |
| respiratory, thoracic and mediastinal disorders | respiratory failure | 150 | 3.35(2.85, 3.94) | 3.34(2.86, 3.91) | 245.72 | 1.74(1.51) | 3.33(2.92) | unknow |
| skin and subcutaneous tissue disorders | blister | 105 | 3.18(2.62, 3.85) | 3.17(2.61, 3.86) | 155.71 | 1.66(1.39) | 3.16(2.69) | unknow |
| general disorders and administration site conditions | multiple-drug resistance | 33 | 13.74(9.74, 19.37) | 13.73(9.65, 19.54) | 384.21 | 3.76(3.27) | 13.56(10.17) | unknow |
| infections and infestations | enterococcal bacteraemia | 22 | 33.57(21.95, 51.33) | 33.55(21.8, 51.64) | 672.57 | 5.02(4.42) | 32.51(22.78) | unknow |
| infections and infestations | rash pustular | 22 | 4.62(3.04, 7.02) | 4.61(3.05, 6.96) | 62.02 | 2.2(1.61) | 4.6(3.24) | unknow |
| infections and infestations | septic embolus | 19 | 37.97(24.01, 60.03) | 37.95(24.18, 59.57) | 658.99 | 5.19(4.55) | 36.62(24.96) | unknow |
| skin and subcutaneous tissue disorders | henoch-schonlein purpura | 49 | 40.7(30.58, 54.16) | 40.65(30.3, 54.54) | 1822.2 | 5.29(4.88) | 39.12(30.81) | unknow |
| skin and subcutaneous tissue disorders | exfoliative rash | 12 | 7.45(4.22, 13.14) | 7.44(4.21, 13.13) | 66.46 | 2.89(2.1) | 7.4(4.6) | unknow |
| cardiac disorders | aortic valve incompetence | 8 | 3.94(1.97, 7.9) | 3.94(1.98, 7.82) | 17.5 | 1.97(1.03) | 3.93(2.2) | unknow |
| general disorders and administration site conditions | infusion site erythema | 13 | 5(2.9, 8.62) | 5(2.89, 8.66) | 41.34 | 2.31(1.56) | 4.98(3.15) | unknow |
| skin and subcutaneous tissue disorders | skin plaque | 30 | 4.71(3.29, 6.74) | 4.7(3.3, 6.69) | 87.08 | 2.23(1.72) | 4.69(3.47) | unknow |
| gastrointestinal disorders | odynophagia | 12 | 3.67(2.08, 6.48) | 3.67(2.08, 6.48) | 23.27 | 1.87(1.09) | 3.66(2.28) | unknow |
| general disorders and administration site conditions | potentiating drug interaction | 15 | 7.23(4.35, 12.02) | 7.23(4.34, 12.04) | 79.93 | 2.84(2.14) | 7.18(4.7) | unknow |
| skin and subcutaneous tissue disorders | ecchymosis | 17 | 4.34(2.7, 7) | 4.34(2.71, 6.95) | 43.55 | 2.11(1.45) | 4.33(2.9) | unknow |
| general disorders and administration site conditions | necrosis | 14 | 3.84(2.27, 6.48) | 3.83(2.26, 6.5) | 29.23 | 1.94(1.2) | 3.82(2.46) | unknow |
| infections and infestations | bacteraemia | 57 | 8.46(6.52, 10.98) | 8.45(6.55, 10.9) | 371.4 | 3.07(2.7) | 8.39(6.74) | unknow |
| infections and infestations | endocarditis staphylococcal | 11 | 35.47(19.44, 64.71) | 35.46(19.31, 65.11) | 355.97 | 5.1(4.27) | 34.3(20.74) | unknow |
| hepatobiliary disorders | hepatomegaly | 20 | 3.38(2.18, 5.25) | 3.38(2.2, 5.2) | 33.44 | 1.75(1.14) | 3.37(2.34) | unknow |
| gastrointestinal disorders | megacolon | 22 | 22.47(14.72, 34.28) | 22.45(14.59, 34.55) | 441.27 | 4.46(3.86) | 21.99(15.44) | unknow |
| infections and infestations | intervertebral discitis | 12 | 10.49(5.94, 18.52) | 10.48(5.94, 18.5) | 101.9 | 3.38(2.59) | 10.39(6.45) | unknow |
| injury, poisoning and procedural complications | toxic anterior segment syndrome | 8 | 10.11(5.04, 20.29) | 10.11(4.99, 20.47) | 65.03 | 3.32(2.38) | 10.02(5.6) | unknow |
| infections and infestations | lactobacillus infection | 7 | 69.92(32.51, 150.39) | 69.91(32.55, 150.15) | 444.91 | 6.03(5) | 65.48(34.5) | unknow |
| blood and lymphatic system disorders | haemolysis | 25 | 4.73(3.19, 7.01) | 4.73(3.2, 7) | 73.18 | 2.24(1.68) | 4.71(3.39) | unknow |
| blood and lymphatic system disorders | haemorrhagic disorder | 21 | 29.48(19.1, 45.5) | 29.46(19.14, 45.34) | 561.25 | 4.84(4.23) | 28.66(19.94) | unknow |
| respiratory, thoracic and mediastinal disorders | pleurisy | 12 | 3.31(1.88, 5.83) | 3.3(1.87, 5.83) | 19.23 | 1.72(0.94) | 3.3(2.05) | unknow |
| infections and infestations | staphylococcal sepsis | 17 | 5.82(3.61, 9.37) | 5.81(3.63, 9.3) | 67.4 | 2.53(1.86) | 5.79(3.88) | unknow |
| infections and infestations | cytomegalovirus colitis | 11 | 7.18(3.97, 12.99) | 7.18(3.99, 12.93) | 58.08 | 2.83(2.02) | 7.13(4.34) | unknow |
| infections and infestations | pneumonia klebsiella | 9 | 8.25(4.28, 15.89) | 8.24(4.32, 15.73) | 56.83 | 3.03(2.13) | 8.19(4.73) | unknow |
| infections and infestations | stenotrophomonas infection | 24 | 29.12(19.41, 43.7) | 29.1(19.28, 43.92) | 633.24 | 4.82(4.25) | 28.32(20.17) | unknow |
| immune system disorders | type i hypersensitivity | 22 | 9.19(6.04, 13.99) | 9.19(6.09, 13.87) | 159.11 | 3.19(2.6) | 9.11(6.41) | unknow |
| hepatobiliary disorders | liver injury | 59 | 4.36(3.38, 5.64) | 4.36(3.38, 5.63) | 152.08 | 2.12(1.75) | 4.34(3.51) | unknow |
| skin and subcutaneous tissue disorders | skin erosion | 17 | 8.53(5.29, 13.75) | 8.53(5.33, 13.65) | 112.01 | 3.08(2.41) | 8.46(5.68) | unknow |
| infections and infestations | pneumonia staphylococcal | 14 | 13(7.68, 22.03) | 13(7.66, 22.07) | 153.13 | 3.68(2.95) | 12.85(8.27) | unknow |
| vascular disorders | vasculitis necrotising | 7 | 13.01(6.17, 27.43) | 13.01(6.18, 27.4) | 76.64 | 3.68(2.68) | 12.86(6.89) | unknow |
| infections and infestations | device related sepsis | 7 | 6.37(3.03, 13.39) | 6.37(3.02, 13.42) | 31.47 | 2.66(1.66) | 6.33(3.4) | unknow |
| infections and infestations | fungal endocarditis | 13 | 63.08(36.02, 110.46) | 63.06(35.72, 111.33) | 747.68 | 5.89(5.11) | 59.44(37.2) | unknow |
| infections and infestations | endocarditis candida | 8 | 143(68.22, 299.73) | 142.97(67.89, 301.1) | 988.98 | 6.97(5.97) | 125.49(67.56) | unknow |
| infections and infestations | brain abscess | 11 | 6.07(3.35, 10.98) | 6.07(3.37, 10.93) | 46.27 | 2.59(1.77) | 6.04(3.68) | unknow |
| vascular disorders | arteritis | 8 | 19.64(9.76, 39.54) | 19.64(9.7, 39.77) | 138.82 | 4.27(3.32) | 19.28(10.74) | unknow |
| general disorders and administration site conditions | brain death | 9 | 4.35(2.26, 8.36) | 4.34(2.27, 8.29) | 23.08 | 2.11(1.22) | 4.33(2.5) | unknow |
| infections and infestations | trichosporon infection | 10 | 33.74(17.97, 63.35) | 33.73(18.01, 63.15) | 307.42 | 5.03(4.16) | 32.68(19.29) | unknow |
| infections and infestations | fungaemia | 18 | 18.22(11.43, 29.04) | 18.21(11.38, 29.15) | 287.6 | 4.16(3.51) | 17.91(12.12) | unknow |
| infections and infestations | arthritis bacterial | 15 | 5.85(3.52, 9.73) | 5.85(3.51, 9.74) | 60 | 2.54(1.83) | 5.82(3.81) | unknow |
| infections and infestations | meningitis bacterial | 12 | 13.65(7.72, 24.12) | 13.64(7.73, 24.08) | 138.73 | 3.75(2.96) | 13.48(8.37) | unknow |
| blood and lymphatic system disorders | splenic infarction | 7 | 6.84(3.25, 14.39) | 6.84(3.25, 14.41) | 34.69 | 2.77(1.76) | 6.8(3.65) | unknow |
| general disorders and administration site conditions | systemic inflammatory response syndrome | 19 | 8.68(5.53, 13.64) | 8.68(5.53, 13.62) | 128.01 | 3.11(2.47) | 8.61(5.9) | unknow |
| injury, poisoning and procedural complications | traumatic lung injury | 8 | 6.2(3.1, 12.43) | 6.2(3.12, 12.31) | 34.69 | 2.63(1.68) | 6.17(3.45) | unknow |
| cardiac disorders | eosinophilic myocarditis | 11 | 21.27(11.7, 38.64) | 21.26(11.81, 38.28) | 208.06 | 4.38(3.56) | 20.85(12.65) | unknow |
| infections and infestations | nosocomial infection | 12 | 8.34(4.73, 14.72) | 8.34(4.72, 14.72) | 76.87 | 3.05(2.26) | 8.28(5.15) | unknow |
| infections and infestations | escherichia infection | 22 | 4.47(2.94, 6.8) | 4.47(2.96, 6.75) | 58.98 | 2.15(1.56) | 4.45(3.14) | unknow |
| infections and infestations | tetanus | 7 | 101.88(46.84, 221.6) | 101.86(46.51, 223.1) | 635.56 | 6.53(5.48) | 92.69(48.38) | unknow |
| investigations | inflammatory marker increased | 23 | 7.59(5.03, 11.44) | 7.58(5.02, 11.44) | 130.52 | 2.91(2.33) | 7.54(5.35) | unknow |
| infections and infestations | human herpesvirus 6 infection | 19 | 12.14(7.72, 19.09) | 12.13(7.73, 19.04) | 191.84 | 3.59(2.95) | 12(8.22) | unknow |
| nervous system disorders | meningitis eosinophilic | 9 | 611.33(267.51, 1397.03) | 611.18(268.32, 1392.13) | 3426.65 | 8.58(7.52) | 382.36(191.49) | unknow |
| infections and infestations | aspergillus infection | 26 | 5.37(3.65, 7.9) | 5.37(3.63, 7.95) | 91.99 | 2.42(1.87) | 5.35(3.87) | unknow |
| cardiac disorders | cardiopulmonary failure | 10 | 3.75(2.01, 6.97) | 3.74(2, 7) | 20.05 | 1.9(1.05) | 3.73(2.22) | unknow |
| hepatobiliary disorders | mixed liver injury | 11 | 8.35(4.61, 15.11) | 8.34(4.63, 15.02) | 70.52 | 3.05(2.23) | 8.28(5.04) | unknow |
| investigations | renal function test abnormal | 11 | 3.76(2.08, 6.8) | 3.76(2.09, 6.77) | 22.23 | 1.91(1.09) | 3.75(2.29) | unknow |
| skin and subcutaneous tissue disorders | nikolsky's sign | 11 | 27.74(15.24, 50.5) | 27.74(15.11, 50.93) | 275.97 | 4.76(3.93) | 27.03(16.37) | unknow |
| respiratory, thoracic and mediastinal disorders | pulmonary haemorrhage | 32 | 6.53(4.61, 9.24) | 6.52(4.58, 9.28) | 148.63 | 2.7(2.2) | 6.49(4.85) | unknow |
| renal and urinary disorders | iga nephropathy | 11 | 10.7(5.91, 19.39) | 10.7(5.94, 19.26) | 95.75 | 3.41(2.59) | 10.6(6.45) | unknow |
| respiratory, thoracic and mediastinal disorders | acute respiratory failure | 36 | 3.42(2.47, 4.75) | 3.42(2.45, 4.77) | 61.51 | 1.77(1.31) | 3.41(2.6) | unknow |
| injury, poisoning and procedural complications | product label confusion | 19 | 4.59(2.93, 7.21) | 4.59(2.92, 7.2) | 53.11 | 2.19(1.56) | 4.57(3.14) | unknow |
| eye disorders | retinal vasculitis | 195 | 264.47(225.87, 309.67) | 263.09(224.91, 307.75) | 40463.8 | 7.71(7.49) | 209.29(183.41) | unknow |
| eye disorders | retinal haemorrhage | 50 | 10.31(7.8, 13.62) | 10.29(7.82, 13.54) | 415.38 | 3.35(2.95) | 10.2(8.08) | unknow |
| eye disorders | retinal ischaemia | 19 | 45.67(28.84, 72.32) | 45.65(29.08, 71.65) | 794.12 | 5.45(4.8) | 43.73(29.77) | unknow |
| hepatobiliary disorders | drug-induced liver injury | 185 | 9.96(8.61, 11.52) | 9.92(8.65, 11.38) | 1469.59 | 3.3(3.09) | 9.83(8.71) | unknow |
| hepatobiliary disorders | hepatosplenomegaly | 7 | 4.16(1.98, 8.74) | 4.16(1.98, 8.76) | 16.72 | 2.05(1.05) | 4.14(2.23) | unknow |
| eye disorders | retinal artery occlusion | 10 | 6.06(3.25, 11.29) | 6.06(3.24, 11.35) | 42 | 2.59(1.74) | 6.03(3.58) | unknow |
| injury, poisoning and procedural complications | seroma | 8 | 11.35(5.65, 22.79) | 11.35(5.6, 22.98) | 74.67 | 3.49(2.54) | 11.24(6.27) | unknow |
| injury, poisoning and procedural complications | drug monitoring procedure not performed | 15 | 12.55(7.54, 20.88) | 12.54(7.53, 20.87) | 157.43 | 3.63(2.92) | 12.4(8.1) | unknow |
| eye disorders | glaucoma | 56 | 4.46(3.43, 5.8) | 4.45(3.45, 5.74) | 149.25 | 2.15(1.77) | 4.44(3.56) | unknow |
| eye disorders | vitreous haemorrhage | 9 | 4.74(2.46, 9.13) | 4.74(2.48, 9.05) | 26.46 | 2.24(1.34) | 4.73(2.73) | unknow |
| vascular disorders | haemorrhagic vasculitis | 49 | 999.57(673.98, 1482.45) | 998.26(674.53, 1477.36) | 24655.03 | 8.98(8.49) | 504.67(362.9) | unknow |
| infections and infestations | leuconostoc infection | 11 | 700.52(325.08, 1509.55) | 700.31(326.07, 1504.06) | 4551.96 | 8.7(7.72) | 415.41(218.51) | unknow |
| immune system disorders | graft versus host disease in gastrointestinal tract | 7 | 4.14(1.97, 8.7) | 4.14(1.97, 8.72) | 16.59 | 2.04(1.04) | 4.13(2.22) | unknow |
| injury, poisoning and procedural complications | transplant failure | 9 | 5.01(2.6, 9.64) | 5(2.62, 9.55) | 28.7 | 2.32(1.42) | 4.98(2.88) | unknow |
| blood and lymphatic system disorders | thrombotic microangiopathy | 28 | 4.15(2.86, 6.01) | 4.15(2.86, 6.02) | 66.6 | 2.05(1.52) | 4.13(3.03) | unknow |
| general disorders and administration site conditions | multiple organ dysfunction syndrome | 221 | 13.02(11.4, 14.87) | 12.95(11.29, 14.85) | 2406.68 | 3.68(3.49) | 12.8(11.45) | unknow |
| injury, poisoning and procedural complications | drug monitoring procedure incorrectly performed | 7 | 15.67(7.43, 33.07) | 15.67(7.44, 33) | 94.69 | 3.95(2.94) | 15.45(8.27) | unknow |
| infections and infestations | mucormycosis | 22 | 10.88(7.15, 16.57) | 10.88(7.21, 16.42) | 195.28 | 3.43(2.84) | 10.77(7.58) | unknow |
| gastrointestinal disorders | noninfectious peritonitis | 29 | 205.3(137.75, 305.98) | 205.14(138.61, 303.59) | 4903.72 | 7.42(6.86) | 170.92(122.4) | unknow |
| investigations | clostridium test positive | 10 | 10.18(5.46, 18.98) | 10.18(5.44, 19.06) | 81.93 | 3.33(2.48) | 10.09(5.99) | unknow |
| gastrointestinal disorders | enterocolitis haemorrhagic | 22 | 21.64(14.19, 33.02) | 21.63(14.05, 33.29) | 423.91 | 4.41(3.81) | 21.2(14.89) | unknow |
| renal and urinary disorders | renal tubular atrophy | 17 | 26.61(16.44, 43.08) | 26.6(16.3, 43.42) | 408.19 | 4.7(4.02) | 25.95(17.34) | unknow |
| nervous system disorders | posterior reversible encephalopathy syndrome | 29 | 4.34(3.01, 6.25) | 4.34(2.99, 6.3) | 74.2 | 2.11(1.59) | 4.32(3.19) | unknow |
| eye disorders | retinal vascular occlusion | 68 | 149.88(116.17, 193.36) | 149.6(115.95, 193.01) | 8752.29 | 7.03(6.67) | 130.57(105.51) | unknow |
| injury, poisoning and procedural complications | transcription medication error | 8 | 5.91(2.95, 11.85) | 5.91(2.98, 11.74) | 32.47 | 2.56(1.61) | 5.89(3.29) | unknow |
| eye disorders | optic atrophy | 8 | 7.09(3.54, 14.21) | 7.09(3.57, 14.08) | 41.53 | 2.82(1.87) | 7.04(3.94) | unknow |
| blood and lymphatic system disorders | autoimmune haemolytic anaemia | 17 | 7.82(4.85, 12.61) | 7.82(4.89, 12.52) | 100.32 | 2.96(2.29) | 7.77(5.21) | unknow |
| skin and subcutaneous tissue disorders | palpable purpura | 10 | 50.95(26.99, 96.15) | 50.93(27.2, 95.36) | 466.21 | 5.6(4.73) | 48.55(28.54) | unknow |
| injury, poisoning and procedural complications | vascular pseudoaneurysm | 7 | 6.74(3.21, 14.17) | 6.74(3.2, 14.19) | 33.99 | 2.74(1.74) | 6.7(3.6) | unknow |
| infections and infestations | lung abscess | 9 | 7.12(3.7, 13.72) | 7.12(3.73, 13.6) | 47 | 2.82(1.93) | 7.08(4.09) | unknow |
| infections and infestations | infective aneurysm | 11 | 30.46(16.72, 55.49) | 30.45(16.58, 55.91) | 304.2 | 4.89(4.06) | 29.59(17.92) | unknow |
| infections and infestations | fungal peritonitis | 15 | 12.92(7.76, 21.5) | 12.92(7.76, 21.51) | 162.84 | 3.67(2.96) | 12.77(8.34) | unknow |
| hepatobiliary disorders | hepatotoxicity | 62 | 3.92(3.05, 5.03) | 3.91(3.03, 5.04) | 133.91 | 1.96(1.61) | 3.9(3.17) | unknow |
| renal and urinary disorders | myeloma cast nephropathy | 22 | 276.83(172.79, 443.51) | 276.67(172.85, 442.85) | 4752.06 | 7.77(7.12) | 217.79(146.81) | unknow |
| immune system disorders | type iii immune complex mediated reaction | 22 | 61.77(40.16, 95) | 61.74(40.11, 95.02) | 1239.43 | 5.86(5.26) | 58.26(40.64) | unknow |
| eye disorders | corneal oedema | 11 | 6.36(3.52, 11.51) | 6.36(3.53, 11.45) | 49.41 | 2.66(1.84) | 6.33(3.85) | unknow |
| general disorders and administration site conditions | drug ineffective for unapproved indication | 260 | 6.97(6.16, 7.87) | 6.93(6.16, 7.79) | 1310.51 | 2.78(2.61) | 6.89(6.21) | unknow |
| blood and lymphatic system disorders | thrombotic thrombocytopenic purpura | 13 | 5.41(3.14, 9.34) | 5.41(3.13, 9.37) | 46.51 | 2.43(1.67) | 5.39(3.41) | unknow |
| vascular disorders | distributive shock | 8 | 6.39(3.19, 12.81) | 6.39(3.22, 12.69) | 36.16 | 2.67(1.72) | 6.36(3.55) | unknow |
| infections and infestations | meningitis candida | 8 | 239.73(110.97, 517.89) | 239.68(111.6, 514.76) | 1539.28 | 7.6(6.56) | 194.22(101.95) | unknow |
| ear and labyrinth disorders | deafness bilateral | 16 | 16.22(9.9, 26.59) | 16.22(9.94, 26.48) | 224.89 | 4(3.31) | 15.98(10.57) | unknow |
| general disorders and administration site conditions | administration site extravasation | 8 | 13.96(6.95, 28.04) | 13.95(6.89, 28.25) | 94.91 | 3.78(2.83) | 13.78(7.69) | unknow |
| infections and infestations | geotrichum infection | 8 | 44.78(22.06, 90.92) | 44.78(22.11, 90.68) | 327.97 | 5.42(4.46) | 42.93(23.74) | unknow |
| infections and infestations | sepsis neonatal | 15 | 36.39(21.74, 60.92) | 36.38(21.85, 60.56) | 498.32 | 5.14(4.42) | 35.16(22.85) | unknow |
| nervous system disorders | myasthenia gravis crisis | 13 | 14(8.1, 24.21) | 14(8.09, 24.24) | 154.78 | 3.79(3.03) | 13.82(8.74) | unknow |
| neoplasms benign, malignant and unspecified (incl cysts and polyps) | langerhans' cell histiocytosis | 7 | 31.42(14.81, 66.66) | 31.41(14.91, 66.15) | 199.94 | 4.93(3.91) | 30.5(16.25) | unknow |
| hepatobiliary disorders | venoocclusive liver disease | 29 | 8.57(5.95, 12.35) | 8.56(5.9, 12.42) | 192.17 | 3.09(2.57) | 8.5(6.26) | unknow |
| respiratory, thoracic and mediastinal disorders | eosinophilic pneumonia acute | 9 | 14.44(7.48, 27.88) | 14.44(7.42, 28.12) | 110.99 | 3.83(2.93) | 14.25(8.22) | unknow |
| injury, poisoning and procedural complications | therapeutic drug monitoring analysis not performed | 7 | 48.52(22.73, 103.56) | 48.51(22.59, 104.19) | 310.89 | 5.53(4.51) | 46.35(24.57) | unknow |
| infections and infestations | empyema | 8 | 6.36(3.17, 12.75) | 6.36(3.2, 12.63) | 35.93 | 2.66(1.72) | 6.33(3.54) | unknow |
| infections and infestations | parvovirus b19 infection | 8 | 14.98(7.45, 30.12) | 14.98(7.4, 30.34) | 102.86 | 3.89(2.93) | 14.78(8.24) | unknow |
| injury, poisoning and procedural complications | product preparation issue | 12 | 3.26(1.85, 5.75) | 3.26(1.85, 5.76) | 18.79 | 1.7(0.92) | 3.26(2.03) | unknow |
| immune system disorders | haemophagocytic lymphohistiocytosis | 37 | 8.81(6.37, 12.18) | 8.8(6.31, 12.28) | 253.78 | 3.13(2.67) | 8.74(6.66) | unknow |
| skin and subcutaneous tissue disorders | fixed eruption | 14 | 11.86(7, 20.09) | 11.85(6.98, 20.12) | 137.55 | 3.55(2.82) | 11.73(7.55) | unknow |
| cardiac disorders | arteriospasm coronary | 11 | 4.42(2.45, 8) | 4.42(2.46, 7.96) | 29 | 2.14(1.32) | 4.41(2.68) | unknow |
| renal and urinary disorders | kidney fibrosis | 16 | 13.06(7.98, 21.39) | 13.06(8, 21.32) | 175.93 | 3.69(3) | 12.91(8.54) | unknow |
| gastrointestinal disorders | dysbiosis | 19 | 55.32(34.86, 87.8) | 55.3(34.55, 88.52) | 960.84 | 5.71(5.06) | 52.5(35.67) | unknow |
| renal and urinary disorders | renal arteriosclerosis | 9 | 59.16(30.21, 115.86) | 59.15(30.38, 115.18) | 486.24 | 5.81(4.89) | 55.96(31.89) | unknow |
| renal and urinary disorders | crystal nephropathy | 8 | 18.28(9.08, 36.77) | 18.27(9.02, 37) | 128.31 | 4.17(3.22) | 17.97(10.01) | unknow |
| investigations | antimicrobial susceptibility test resistant | 7 | 29.84(14.07, 63.28) | 29.83(14.16, 62.82) | 189.53 | 4.86(3.84) | 29.01(15.47) | unknow |
| eye disorders | haemorrhagic occlusive retinal vasculitis | 14 | 620.27(319.16, 1205.48) | 620.04(318.42, 1207.35) | 5378.62 | 8.59(7.73) | 385.81(221.26) | unknow |
| hepatobiliary disorders | hepatic cytolysis | 16 | 3.97(2.43, 6.48) | 3.96(2.43, 6.46) | 35.33 | 1.98(1.3) | 3.95(2.62) | unknow |
| vascular disorders | superficial vein thrombosis | 9 | 28.57(14.73, 55.41) | 28.56(14.67, 55.61) | 232.83 | 4.8(3.89) | 27.81(15.97) | unknow |
| blood and lymphatic system disorders | myeloid maturation arrest | 11 | 186.8(98.21, 355.31) | 186.75(97.8, 356.58) | 1717.44 | 7.3(6.42) | 157.97(92.24) | unknow |
| nervous system disorders | spinal stroke | 9 | 247.84(119.61, 513.53) | 247.78(119.98, 511.7) | 1779.23 | 7.64(6.66) | 199.49(108.44) | unknow |
| nervous system disorders | paraplegia | 8 | 3.99(1.99, 7.98) | 3.99(2.01, 7.92) | 17.83 | 1.99(1.05) | 3.98(2.22) | unknow |
| infections and infestations | rhinocerebral mucormycosis | 13 | 50.18(28.75, 87.57) | 50.16(28.97, 86.84) | 596.95 | 5.58(4.81) | 47.85(30.03) | unknow |
| infections and infestations | septic pulmonary embolism | 8 | 78.37(38.18, 160.88) | 78.36(37.94, 161.82) | 567.32 | 6.19(5.21) | 72.83(39.9) | unknow |
| immune system disorders | jarisch-herxheimer reaction | 8 | 13.77(6.85, 27.66) | 13.77(6.8, 27.89) | 93.44 | 3.77(2.82) | 13.6(7.58) | unknow |
| congenital, familial and genetic disorders | fanconi syndrome | 13 | 12.97(7.51, 22.42) | 12.97(7.49, 22.45) | 141.81 | 3.68(2.92) | 12.82(8.11) | unknow |

**Abbreviations:** SOC = system organ classe , ROR = Reporting Odds Ratio, PRR = Proportional Reporting Ratio,EBGM = Empirical Bayes Geometric Mean ,IC=Information Component, PT = preferred term
