# Supplementary material for: Evaluating adverse reaction signals of vancomycin in pediatric patients: A FAERS database analysis
Source: Medicine (Baltimore). 2026 Jun 5;105(23):e49064. doi: 10.1097/MD.0000000000049064 (PMC13246103; doi:10.1097/MD.0000000000049064)
Supplement: Supplementary file 9 [file medi-105-e49064-s010.docx]

**Table S2** Signal Detection Algorithms: Formulae and Thresholds

| Algorithms | Calculation formula | Criteria |
| --- | --- | --- |
| ROR | 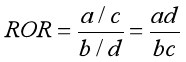  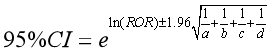 | (1) a ≥ 3  (2) ROR ≥ 2  (3) 95%CI > 1 |
| PRR | 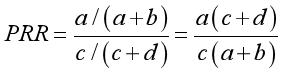  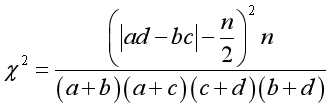  n= a + b + c + d | (1)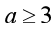  (2) PRR ≥ 2  (3)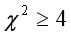 |
| BCPNN | 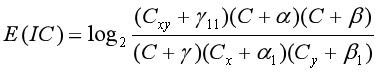  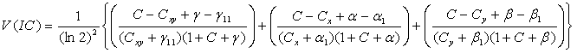  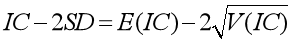  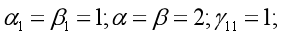 | (1) a ≥ 3  (2) IC-2SD > 0 |
| EBGM | EBGM=a(a+b+c+d)/[(a+c)(a+b)] | (1) a > 0  (2) 95%CI > 2 |

**Abbreviations:** ROR = Reporting Odds Ratio, PRR = Proportional Reporting Ratio,EBGM = Empirical Bayes Geometric Mean
